# Supplementary material for: Transcriptomic and biochemical insights into key gene networks driving bulbil development of Pinellia ternata (Thunb.) Breit
Source: PLoS One. 2025 Feb 11;20(2):e0314396. doi: 10.1371/journal.pone.0314396 (PMC11813136; doi:10.1371/journal.pone.0314396)
Supplement: S1 Fig — (PDF) [file pone.0314396.s008.pdf]

钦诚生物
QCHENG BIO
TEL :021-67652295 www .shqcsw .com

This kit can only be used for scientific research and not for medical diagnosis

Plant (Plant) auxin (IAA) ELISA test kit

operating instruction

Detection principle

The kit used double antibody one-step sandwich enzyme-linked immunosorbent assay (ELISA). Add the specimen, standard, and HRP-labeled auxin (IAA) antibody to the well, warm and wash thoroughly. Colorcolor with the substrate TMB, TMB is converted to blue catalyzed by peroxidase and to the final yellow with the acid. The depth of the color was positively correlated with the auxin (IAA) in the sample. Measure the absorbance (OD) at 450nm to calculate the sample concentration.

Sample collection, processing, and preservation methods

1. The sample should not contain sodium azide (NaN 3) because sodium azide (NaN 3) is an inhibitor of horseradish peroxidase (HRP).

2. Extract the specimens as soon as possible after specimen collection, and the extraction is carried out according to the relevant literature.

3. Plant extract or other related samples: please centrifuge at 1000 xg for 20 minutes, and remove the supernatant to detect.

4. Storage: If the sample is not tested in time after collection, please divide the samples according to one dosage and freeze at-20℃ to avoid repeated freezing and thawing, thaw at room temperature and ensure that the samples are thawed evenly and fully.

Self-provided items

1. Microplate reader (450nm)

2. High-precision sampler and gun head: 0.5-10 uL, 2-20 uL, 20-200 uL, 200-1000 uL

A 3. 37℃ incubator

Operational considerations

1 The kit was stored at 2 - 8℃ and equilibrated at room temperature for 20 min before use. The concentrated washing liquid taken out from the refrigerator will be crystallized, which is a normal phenomenon, and the water bath heat makes the crystallization completely dissolved before use.

2. The slats not used in the experiment should be immediately put back into the self-sealing bag and sealed (dry at low temperature) for preservation.

3. S 0 standard with concentration of 0 can be regarded as negative control or blank; the sample has been diluted 5 times according to the instructions, and the final result is multiplied by 5.

4. Warm the operation in strict accordance with the time, quantity and order indicated in the instructions.

5. Shake all the liquid components well before use.

Kit composition

name	The 96-well configuration	48 Hole configuration	remarks
Micropore plate	12 wells and 8 bars	12 wells and 4 bars	not have
good merchantable quality	0.3 mL * 6 tube	0.3 mL * 6 tube	not have
Sample dilutions	6mL	3mL	not have
Detection of the antibody-HRP	10mL	5mL	not have
20 in the washing buffer	25mL	15m L	Dilute according to the instructions
substrate A	6mL	3mL	not have
substrate B	6mL	3mL	not have
stop buffer	6mL	3mL	not have
Seal plate film	2 Zhang	2 Zhang	not have
instructions	A	A	not have
valve bag	One	One	not have

Note: The concentration of the standard product (S 0-S 5) is successively: 0,3,6,12,24 and 48 nmol / L

Preparation of reagents

20 Wash buffer dilution: distilled water press 1:20, dilution, namely 1 part of 20 washing buffer plus 19 parts of distilled water.

Plate washing method

1. Hand wash the plate: shake the liquid in the hole, fill the washing liquid with each well, stand for 1min before shake the liquid in the hole, pat dry on the absorbent paper, and wash the board 5 times.

2. Automatic plate washing machine: inject 350 μ L of lotion into each well, soak for 1min, and wash the plate for 5 times.

operating steps

1. Remove the required strip from the aluminum foil bag balanced 20min at room temperature and seal the remaining strip with a self-sealing bag for 4℃.

2. Set up standard holes and sample holes, with 50 μ L of different concentrations of standard holes;

3. Add 10 μ L of sample to be tested, and then 40 μ L of sample dilutions; no blank hole.

4. 100 μ L of horseradish peroxidase (HRP) -labeled detection antibody was added to each well of the standard well except the blank, the reaction well was sealed with a sealing plate film, and the 37℃ water bath or incubator was incubated for 60min.

5. discard the liquid, pat dry on the absorbent paper, fill the well with the washing liquid, stand 1min, shake the washing liquid, pat dry on the absorbent paper, and wash the plate 5 times (you can also wash the plate by washing plate machine).

6. 50 μL of substrates A and B were added to each well and incubated for 37℃ protected from light for 15min.

7. 50 μL of the termination solution was added to each well for 15min, and the OD value of each well was measured at the 450nm wavelength.

Results judgment

Draw the standard curve: in the Excel worksheet, take the standard

concentration as the abscissa and the corresponding OD value as the ordinate

Standard linear regression curve, the concentration value of each sample was calculated by the curve equation.

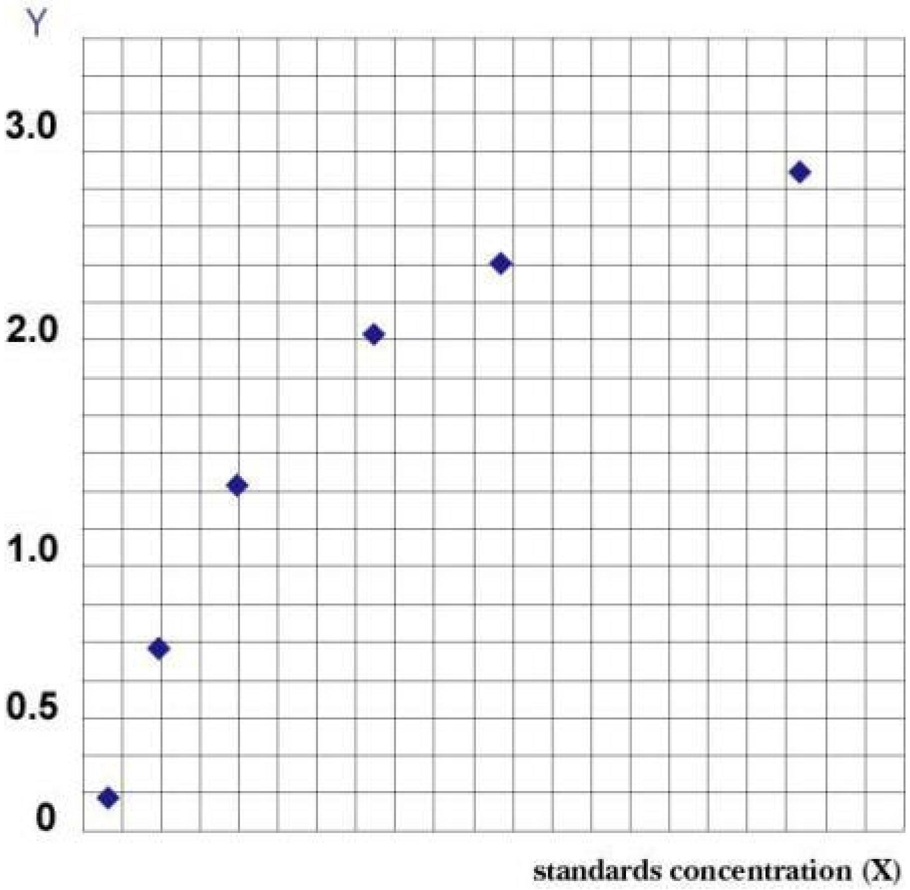

Kit performance

1. Accuracy: R value of standard linear regression and expected concentration correlation coefficient, greater than or equal to 0.9900.

2. Sensitivity: the minimum detection concentration is less than 1.0 nmol / L.

3. Specificity: it does not cross-react with other soluble structural analogues.

4. Repeatability: the coefficient of variation within and between plates is less than 15%.

5. Storage: 2-8℃, protected from light and moisture.

6. Expiry date: 6 months

disclaimer

1. The kit is only for research use and shall not be used in clinical experiments or human experiments, otherwise, all the consequences arising therefrom shall be borne by the experimenter, and the company shall not be responsible.

2. Operate in strict accordance with the instructions. If the experimenter violates the instructions, the consequences shall be borne by the experimenter.
