# Supplementary material for: Transcriptomic and biochemical insights into key gene networks driving bulbil development of Pinellia ternata (Thunb.) Breit
Source: PLoS One. 2025 Feb 11;20(2):e0314396. doi: 10.1371/journal.pone.0314396 (PMC11813136; doi:10.1371/journal.pone.0314396)
Supplement: S1 Text — (DOCX) [file pone.0314396.s014.docx]

>TRINITY_DN16871_c0_g1_i7_3
GATTTCATCATCACCAGTACCTACCAAGAGATCGCCGGAAGCAAGGACACGGTGGGGCAGTACGAGAGCCACACCGCCTTCACCATGCCGGGGCTCTACCGCGTGGTCCACGGCATCGACGTGTTCGACCCCAAGTTCAACATCGTCTCTCCGGGCGCGGACATGTCCATCTACTACCCTTACTCGGAGGAGAGCAAGAGGCTCACCGCCCTCCACTCTGAGATCGAGGAGCTCCTCTTCAGTGACGTGCAAAACGGAGACCACATGTGAGCAGCACAGCACTTGAGCTCAGCTAGCTAGCCAGCAATCCAGCTGCTATATACAATTAATACATACAAATATATATATATGGTGTCTTCAACTTTTGATGATGCTAGCTACATCTCAATATAATTACTTGCATGGGCATATCTATATATGTATGTATGTGCAGATGTGTGCTCAAGGACAGGAGCAAGCCCATCCTCTTCTCCATGGCCAGGCTGGACCGCGTGAAGAACATGACGGGGCTCGTGGAGCTGTACGGGAAGTCCCCCAAGCTGCAGCAGCTGGTCAACCTGGTCGTGGTCTGCGGAGACCACGGGAAGGAGTCCAAGGATTTGGAGGAGCAGGCCGAGTTCAAGAAGATGCACAGGCTCATCAAGGAGCACAACCTCGACGGCCACATCCGCTGGATCTCTGCCCAGATGAACCGAGTCCGCAACGGCGAGCTCTACCGTTACATTGCCGACACCAAGGGCGCATTCGTGCAGCCCGCCTTCTATGAGGCGTTTGGTCTCACCGTGGTGGAAGCCATGACTTGCGGTCTGCCGACGTTTGCCACCCTGCACGGAGGGCCTGCGGAGATCATAGTGGACGGCGTCTCTGGCTACCACATCGACCCATACCAGGGCGACAAGGTCGCCGACATCCTGGTCAACTTCTTCGACAAGTGCAAGGCGGATCCAACCCACTGGGACAGCATCTCCCAGGGCGGCCTCAAGCGTATCTATGAGAAGTACACATGGCAGCTCTACTCAGAGAGGTTGATGACGCTGTCCGGGGTGTATGGGTTCTGGAAGTATGTGTCCAACCTGGAGAGGCGCGAAACCCGTCGTTACCTGGAGATGTTCTACGCCCTCAAATACCGCAACCTGGTAAAGCTACTTAAATTAATTTCAGCTTCTTCCACCTCTCTCTCTCTCTCTATATGCTCATATATCTGTGTTTTCTTGAAAGAAAAAGGGTAAGTAATTACTCGCTTGTGAAACCCGGCCCCCATTATCACCATACCCTGTTTGCCTCTGATATAATAGATATATATATATAGGCTCATTATAAGTTTCCAAACATCTAATTAATTATTACATGTCATACGATATATATACATCATGTTCAGATCATAACCCCAAATATTGTCTTGTTCCTCCATTTTTGAACAATTATTAAAGAAGAGAAAAGGTTCTTGCAATATCTTTGCAATAAAGTGTAGTGGGACTGCAAGGAATAAAGTAATGGCCCATCATGCATGATTGGAGAGCCCTGACGACGACAAGTGCCATACCTTTTGGTACATAACATCAGCCAATTAATAAAACTACTTCCCTTTGCAATCCAATTCAAGTACCTTATAGTAGTTCTCTCTCTCTTTTTTTTCCTGTTGAGTACATCTATTTAAATTGAGCTACGTACTTATAGACCAAACTAGAAGAGCAGAGCGCTCTTAATTTGTAGAAATCTCAAGTACTGCTTTTTGGACATTAAAATGAAGAGAGCCTCTTGTATCATCACTGACTATATATGGTGATCTTATAATGTGCTGTTTTCTTTTTCTTTTTTTGGCAGGCAAAATCTGTTCCGCTTGCTATTGAAGGTGAGACTGCAGTTATCAATGGCACCAAGTAGCTAGCTAGGAGGGCTCGATCGAGGGGGAGTTCGTACGTTTCGATCGGCAGCTTCCCCATAGATCCCCACACCCACTTTTTCCAAATTAGCAGCTTTTAATTATTTCCCATATGATCTTGTCCTGATCAAGATGTTTGTCTTTTAATCTGCAAGTGA

>TRINITY_DN18118_c0_g1_i1_5
CATGCCGGGGCTCTACCGCGTGGTCCACGGCATCGACGTGTTCGACCCCAAGTTCAACATCGTCTCTCCGGGCGCGGACATGTCCATCTACTACCCTTACTCGGAGGAGAGCAAGAGGCTCACCGCCCTCCACTCTGAGATCGAGGAGCTCCTCTTCAGTGAGGTGCAAAACGGAGACCACATATGTGTGCTCAAGGACAGGAGCAAGCCCATCCTCTTCTCCATGGCCAGGCTGGACCGCGTGAAGAACATGACGGGGCTGGTGGAGCTGTACGGGAAGTCCCCCAAGCTGCAGCAGCTGGTCAACCTGGTCGTGGTCTGCGGAGACCACGGGAAGGAGTCCAAGGATTTGGAGGAGCAGGCCGAGTTCAAGAAGATGCACAGGCTCATCAAGGAGCACAACCTCGACGGCCACATCCGCTGGATCTCTGCCCAGATGAACCGAGTCCGCAACGGCGAGCTCTACCGTTACATTGCCGACACCAAGGGCGCATTCGTGCAGCCCGCCTTCTATGAGGCGTTTGGTCTCACCGTGGTGGAAGCCATGACTTGCGGTCTGCCGACGTTTGCCACCCTGCACGGAGGGCCTGCGGAGATCATAGTGGACGGCGTCTCTGGCTACCACATCGACCCATACCAGGGCGACAAGGTCGCCGACATCCTGGTCAACTTCTTCGACAAGTGCAAGGCGGATCCAACCCACTGGGACAGCATCTCCCAGGGCGGCCTCAAGCGTATCTATGAGAAGTACACATGGCAGCTCTACTCAGAGAGGTTGATGACGCTGTCCGGGGTGTATGGGTTCTGGAAGTATGTGTCCAACCTGGAGAGGCGCGAAACCCGTCGTTACCTGGAGATGTTCTACGCCCTCAAATACCGCAACCTGGCAAAATCTGTTCCGCTTGCTATTGAAGGTGAGACTGCAGTTATCAATGGCACCAAGTAGCTAGCTAGGAGGGGCGCAGATCGAAGGGGAGTTCGTACGTTTCGATCGGCAGCTTCCCCATAGATCCCCAGTACCCCCACTTTTTCCAAATTAGCAGCTTTTAATTATTTCCCATATGATCTTGTCCTGAACAAGATGTTTGTCTTTAAATCTGCAAGTGAGAGTAGTACTGTCACCCTTTTAATTGGTCGACCATCGACCGACACGAATATTCCCCTTTTTGCTCTCTGCAGCGAGTGGTATTCTCCTCGATCGTTATAGACGGACGGGTGATGTATGTATGATCTCGCGGGTATGAATAATACAAGGCTCCTTGCCAATGTTATGCACCAA

>TRINITY_DN30678_c1_g4_i2_1
ATATATATATATATATATATCTCAGAATTTATGTAGTCGACCCAATTTAAATGAGATAAGACTTTGATAACGATGATGATGATGACATCTCCCAGATCGCTAACTAGCTAGCCCTCTATATTTCTGTGCACGCATGTGTGTCCGTCCAATTAATATTCCGTTATATATGCATGCATGCTCTCGAAGAAGCCTCTCTATATATATTATTATTAATTAACCGGCCAGGAGGCCCGTGCAGACGATGATGCTCAACAACAGAATACGCAGCCTCAGCGCTCTCCGAGCCGCACTGAGGAAGGCGGAAGAACACCTGCTGAATGTGCCATCTGACACCCCATACTATGAGTTCGACCACAGGTTCCAAGAGCTCGGGTTGGAGAAGGG

>TRINITY_DN30825_c0_g1_i1_4
AGAGAGAGAGAGAGAGAGAGAGAGAGAGAGAGAGAGAGTTGGAAGGCTTCTTCTTGGAGGTGAAACCATGCCGGAACGCCAGCTGACCCGTCTGCACAGCCTCAGGGAGCGCCTCGCCGACACCCTCGCCGCCCACCCAAATGAGCTGCTGGCCCTCTTCTCCAGGTTTGTTCACCAGGGGAAGGGGATGCTGCAGCCGCACCAGCTGCTGGCGGAGTTCGAAGCTGTTTTCCCCGATGGGGACAAGCAGAGGCTGAAAGATACCGTGTTTGGGGAAGTCTTGAGGGCGGCCCAGGAAGCCATTGTGTTACCACCCTGGGTTGCTCTGGCCGTCCGTCCAAGGCCTGGTGTCTGGGAGTACATTCGTGTGAACGTGAACGAGCTGGTTGTGGAGGAGCTGCGTGTGCCCGAGTACCTGCAGTTCAAGGAAGAACTTATCGATGGAGGCTGCCACAGCAACTTTGTTCTTGAGCTGGACTTTGAGCCCTTCAACGCATCCTTCCCTCGGCCCTCCCTGTCCAAATCCATCGGTAATGGCGTGCAGTTCCTGAACCGGCACCTCTCCTCCAAGATGTTCCACGACAAGGAGAGCATGGAGCCCATGCTCAACTTTCTCCGCAAGCATAACTACAACGGCATGACGATGATGCTCAACAACAGAATACGCAGCCTCAGCGCTCTCCGAGCCGCACTGAGGAAGGCGGAAGAACACCTGCTGAATGTGCCATCTGACACCCCATACTATGAGTTCGACCACAGGTTCCAAGAGCTCGGGTTGGAGAAGGGGTGGGGTGACTGCGCCGAGCGCGTGCACGAGAACATCCACCTGCTCCTGGACCTTCTCGAGGCTCCCGACCCCTGCACCCTGGAGAGGTTCTTGGGCACCATCCCCATGGTCTTCAACGTGGTCATCCTCTCCCCCCACGGCTACTTCGCCCAGGCCAACGTCCTGGGTTATCCCGACACCGGCGGCCAGGTGGTGTACATCCTGGACCAAGTTCGCGCCTTGGAACATGAGATGCTGGTGCGGATAAAGCGCCAGGGGCTGGACATTACTCCGAGGATCCTCATCGTGACGCGGCTGCTGCCTGACGCAGTCGGAACCACCTGCGGTCAGCGTCTGGAGAAGGTCCTCGGCACCGAGCACACCCACATCCTGCGCGTTCCTTTCAGAACAGAGCACGGCATCGTCCGTAAATGGATTTCCCGCTTCGAGGTCTGGCCTTACCTGGAGACCTACGCCGAGGATGTTGCGAATGAGGTTGCAGGGGAGCTGCAGGCCAAACCCGACCTGATTATTGGCAATTACAGCGACGGGAACCTCGTCGCGTCTCTGATGGCGCATAAACTAGGAGTCACTCAGTGCACCATTGCCCATGCGTTGGAGAAGACCAAGTACCCCAGCTCGGACATATACTGGAAGAAGTTCGAAAACCAGTACCACTTCTCGTGCCAGTTCACTGCCGACCTGATTGCCATGAACCACGCCGATTTCATCATCACCAGTACCTACCAAGAGATCGCCGGAAGGTACGTACGCATCATCATAATTAGAGAGTACTGCACAGATCGAGCCCATACAATATTTGTATACCTTCTTGTACTTTGTACGACCAATCATTTCTCCTGTGTAATGTTTAATCGATCGATATATCTTTTTTTTTTTTTTTGACAAAGCAAGTAGTAGATTCTATTAAAGATAACACATTACACATATGAGGGATGCCCATAGCCCTAAACAAGAGACGACAACATCGGGGCTAGCCATAACATGGGCAGGGCAAGATGATAACAGACACATCACAGGGGATGATAACAACATCCAAAGCAAAGGGAATGCATACGAACTAGATAACCACTGGAACCGCC

>TRINITY_DN23254_c2_g2_i4_3
GGGAGGGAGGATTCTTGGACGTCGAGGGAGTCGTCGCCGAGGGACTCCTCGTCGGCGCCCATGTCGTCCAGCGGCGTGTCCATCTGCCACTGCGGGTGCCGCATCCGGCAGGCCGCCACCCTGGTAAGGTAGGTGCGGCAGTGCTCCAGCTAGGAGAAGTGGTGGATGCCGCTCCTCTTCCAGTAGTCGTAACAGTACTTGGAGAACACTCACGGCAGATGCTCGAGCCCAATCGCCTCAGCCCAGCGACGCCACTGTCATCACCAAGTGGACCGGTCTTCAGAGCCGGCAGAGCCGACTGCTGCTGTGGCCACCTTGGGCACCCGACGACGGTCCTCCTGCAATTTCTCATTCAGGCCGGTAATTTCTTACATGAGTCCCACGAATTTCAGTTTTCTCGCCCGTCTCCCAAATAGGAAGGTTATTAAGATCGTTCTGGATGAAGAGCAACAAGACAGTTATATTCCTCAGAAGATTGTCGATCTCCTATTACAAAGAGGTGAAATAATTGTTGAAGGTAATGAAGCTCTTATTGAGTTCGACCGTCACTTTAGAGACGAAGCTTCTTTACAAGCTACTTATTTTTATGATGGAGCTTGGTGCAAAATTGATTCTTCAAGATCTTATCTCACTTTGGGGCGAAAGTGGATGGAGGAGCGACGTGTTATGATAGACTCGGAACAGTCTATTTGTACCATCCGGCCACCACATATAGGGAGCAACTTTCATTTTCCGAAGTATGTCTCTAAAAGGCTCAAACCAGTGCTTCCAGAGGAAAGGGTGACAGAAGAGAAACCATTAGAGTTAGCAACTCTTGCTGACCGTGTATGTGTGGAGTCCGTAGTTGCTGAAGTTTGGGACCAAGAGTTGTTTGTTGAGTCTGGACTGACATTAGTGGATGATTCCATTTTCATATTTGATTCAGCATTTGAGATTGCGACAACAATCAAAGTTGAAGACACATGGGATCAAGCGTTGTTTATACGATCTGGGTTGAGCCGTATTTTTCCAGATATTTGCACGGCTGCTCCTCTATATGTGCTCCCCCCAGTTGAAGTCCTTACTCCAAGTGAAGCGCTACCAGTTTATGAACCTACTGATCTGATCAAGTGGATGCAACCCTCAGCTGATTTAATATGGTTGACGTTTTGTGACTCACAGTTGGCACATCCAGTGGAGACAGTCATTATCAAGCCTTGCTCAGAAGCTTCCGTGGTTTATCTCGTCACTCGCTTGCTTATATTAGCACGTTTCAAAACTCGAGGACGAGTTTTCTCGAGCCAGGGGAGTATGATGGGAATCGAAGAGCTAGAAAAGATATTTTCATGTTTATTTTTATATTTTGTTTGGCCTATAAGAGGC

>TRINITY_DN24836_c0_g1_i1_5
GGGGAGGCAGGAGAAGGGAGCGATGGCCGGGAACGAGTGGCTAAACGGGTACCTGGAAGCCATCCTCGACGCCGGCGGGCCGGGAAAGGGCCTCGAAGGCGGCGGCAGCGGCAGAGGGGAGAACGAGCCGCCGTGGGCGGCGACGTCGCTGGTGCCCCACCTCCAGTTCAGCCCCACCAAGTACTTCGTGGAGGAGGTCGTCAACAGCTTCGACGAGGCGGACCTGTACAAGACATGGGTCAAGGTGATCGCCATGAGGAATACGCGGGAGCGCAACAACAGGCTCGAGAATATGTGCTGGAGAATATGGCATCTTGCCCGTAAAAAGAAGAAGATAGAGAGGGAGGATGCGCGCCGCCTCTCGAAGAGGCGCCTGGAGCGCGAGCAAGGCCACAGGGACGCGGCGGAGGACCTGTCGGAGCTGTCCGAGGGCGAGAAGGAGAAAGAGAACGGCGGCCAGGCGGCGGGACCGCCCAAGGAGAAGATGCTGCGAATCAACTCCGACCTGCGGCTTTGGTCGGACGTCGACGAGACGAAGCGCCTCTACATCGTCTTGATCAGCTTGCATGGGTTGGTACGTGGAGAAAACATGGAACTGGGACGAGACTCGGATACGGGAGGGCAGGTGAAGTATGTGGTGGAGCTGGCCCGGGCCTTGGCAGCCACCAAGGGGGTCCACCGTGTGGACCTCCTCACTCGCCAGATCTCCACACCGGACGTGGACTGGAGCTACGGCGAACCCGTCGAAATGCTATCACGGCCGTCCGATGTCGAGTCCAACGGTGATGGCTGCGGTGCGTACATCATCCGACTCCCATGTGGACCTCGGGACAGGTATCTGGCAAAGGAATCTCTGTGGGCCCACATCCCTGAGTTCGTGGATCGAGCTTTGGCCCACATAACCAACGTGGCGCGCTCCCTCGGTGACCAGACCGAGGACGGGAAGCCCACATGGCCCTATGTCATCCACGGCCACTACGCCGACGCCGGCGAGGTGGCCGCCCGGCTCTCCGGCTCCCTCAACGTGCCCATGGTGATGACAGGCCACTCGCTGGGGCGCAACAAGTTCGAGCAGCTGCTGAAGCAGGGGAGGCTCAGCCGGGAGGACATCAACTCCACGTACCACATCATGCGCCGGATCGAGGCGGAGGAGCTCGGCCTGGACGCGGCGGAGATGGTGGTCACCAGCACCCGGCAGGAGATAGAGGAGCAGTGGGGCCTCTACGACGGGTTCGACGTGAAGATAGAGAGGAAGCTCAGGGTGCGGCGGCGGAGGGGGGTGAGCTGCCTCGGCCGCCACATGCCCCGCATGGTGGTCATTCCGCCGGGGATGGATTTCAGCTACGTGACGATGGATTCGCCGGACGGAGAAGGTGACCTGCAGTCTTTGATCGGTCCCGACAGGGCCCAGACCAGAAGAAATCTGCCCCCAATATGGTCCGAGGTGATGAGGTTCTTTACGAACCCCCACAAGCCCATGATCCTAGCACTCTCCCGTCCAGACCCGAAGAAGAACGTCACCACGCTGCTGAAAGCCTTCGGGGAAAGCCGGTCTCTCCGGGAGCTCGCTAACCTGACGCTCATATTAGGGAACAGGGACGACGTCGAGGAGATGCCGGCCAGTAGCTCCACCGTCCTCACCACCGTCCTCAAGCTCATCGATAAGTACGACCTCTATGGGCACGTGGCGTACCCCAAGCATCACAAGCAGTCTGAGGTTCCACACATATATCGTCTTGCTGCCAAGACCAAGGGCGTGTTCATCAATCCAGCCTTGGTGGAACCCTTCGGCCTCACCCTCATAGAGGCAGCTGCTTATGGTTTGCCCGTCGTTGCCACCAAAAATGGTGGACCAGTGGATATCCTCAAGACGTTGAACAATGGCTTGCTGGTGGACCCCCACGATCAGAACGCCATATCCGACGCCCTCCTGAAGTTGGTGGCCGACAAAGGCTTGTGGCTAGAATGCAGGAAGAATAGCCTCAAGAACATCCACCGTTTCTCCTGGCCCGAGCACTGCCGGAACTACCTCTCCCACGTCGAGCACTGCCGCAGCCGCCACCCGGGCACCCATCTCGACGTGGCGCCGCGGGCAGAGGAACCTATGAGCGACTCCCTCAAGGACGTCGACGACCTCTCCATCCGGCTCTCGGTGGAAGGAGGCAGCGACCTCAAGGCCAACGGCGACATCGCCACCGTCCTCGACGCCCTACGCCGTCCCCGACCCGAGCACACGCACCACCACCCCGCCGCCGACGGCAACTCCCCCACGGGGTACTACCCCGGGAGGCGGCGACGGCTTTTCGTGATCGCTGCCGACTGCTACGATTCGGAGGGGCGGATCGCCGTCGGCGTGCTGAAAGGAATCGTCGAGAAGGTGGCGGCCGCCGCTGGTCCAGACGGGCGAACGGGGCTCGTCATCTCGACGGGTTCCACGGTGGCAGAGACGATAGAGGCCTTAAACCTGTGCCGCATAGCGCCGAGCGACTTCGACGCACTGATCTGTAGCAGCGGCAGCGAAGTCTGCTACCCGTGGAAGGACCTGGCACCAGATGCAGACTACTCGGCGTACGTGGACTACCGGTGGCCGGGCGAGCACATCCGATCCGCAGTGCCGCGGCTCGCGAAGCTTGGCGGCACCGGGAAGGAGGACGCTGAGGCGGAGGTAGATGCCATGGAACTCGACGAGAAAGCCTGCGGCTCTCACTGCTACGCATACTCCCTGAAGGTGGGAGCCAAGGCTCGGAAGACCGACGAGCTTCGGCAGAGGCTGCGGATGCGGGGATTCCGCTGCAACCAGGTTTACACCCGTGCTTGCACGCGCCTGAACGTGGTGCCTCTGTTCGCATCGAGGGCGCAGGCCCTGAGGTACCTGTGGATCCGGTGGGGCGTGGAGCTCTCGGAGATGGTGACGTTCGTGGGGGAGAAGGGGGACACGGACGCGGAGGAGCTCCACCTCGGCCTCCACAGCGCCGTGGTCCTCGGCGGCTCCGTGCAGCACGGGAGCGAGAGGCTGCTGCGGAGCGAGGAGAGCTACAAGAGAGAGGACGTGGTGCCGTCGCAGTGCCCCAACGTGGCGACCGTCGCCGGCGACTGCAGCGTTCGGGACATCCTCGACGCGCTCGCGGGTCTCGGCGTGAAGTGATGCGGGCGGCGCCAAAGAACGGCCTGCCGTCTATTTATACGGTTTTTTTACCAACTACCAGCTGTTTTGTTTTTT

>TRINITY_DN29370_c0_g2_i3_1
TCTAATGCGGCTGCACGGAGGAGAGAGTAGAAGAGCCGACCGCCATTGCTAGCGGGGCAATGGAGGACGAGAGTTGCCGACCTCTCATCTCTGCTTCTCGTTTATCTCGAGCATAGGCGTCGGCAATCTGCCCTTCTCCTGTTCGTTCTACTCCTCTTCTCTCTCTCTCTCATATCGAATCAAATTACGGAGTAAAGATCCCGTTTTTTCCTGTCTCTGTGGGCATCTTCTGGACCGAAGTTGCAGACTGGAAGGTGCCCAGATGAGGAGAATCCCCCGTTTACGCGCATTCCTCCTGCCCGCAGCGCATTCGCCGCCACCGCATCAGCAACGGGACTGCAAGTGCTAGTTTCACCGCCTATCGCGTCGCTCTTGCGCTGCTCTGTACGCAAGGGGGGGACGGAGGGCAGATTCCCATCTCTATTCTCTACCTGTGTGTTCTTTTGTAGAGCAGAGCAATGGCGGGGAACGAGTGGATTAATGGGTACCTGGAGGCCATATTGGACAGCGGCGCGTCGCCGATTTCGGGTGAGGACCAGCGAGGGGTGGTGTCGCCTGCGGAGCTGAAGGAGACACCCACCGCCGGCGGGAGGGCCGGCGCCGTCGTCCACTTCAACCCCACGAGGTACTTCGTGGAGGAGGTGGTGATGGGTGTGGACGAGACGGACCTCCACCGGACATGGATCAAGGTTGTCGCCACCCGCAACACACGCGAGCGCAGCTCCCGGCTGGAGAACATGTGCTGGCGCATCTGGCACCTCGCTCGAAAGACGAAGCAGATAGAATGGGAGAACTTTCAAAGGGCTTCAAGGAGGAGGATGGAGCGGGAACAAGGGAGGAGGGAAGCTGCAGAGGACATGTCAGAGGACTTGTCAGAAGGAGAGAAGGGGGACACAGTTGGAGAACTTGTACAGCCTGAGACACCAGGGACAAAGATTCAGAGAAATGTATCTGATGTTCAAATATGGTCTGATGACATTAAGGGGAAGAAACTCTACATTGTGCTGATTAGTTTGCATGGCTTAGTCCGTGGGGATAACATGGAGCTAGGCAGAGACTCTGATACAGGTGGTCAGGTGAAATATGTGGTGGAACTTGCTCGAGCACTTGCCATGATGCCTGGGGTATACAGGGTTGACCTCTTTACTCGCCAGATATCAAGTCCTGAGGTGGACTGGAGCTATGGAGAGCCAACAGAAATGCTAACTGCAGGTTCAGAAGATGCAGAAGAAAATGATGCTGGAGAGAGTGCTGGGGCATACATCATCCGCATCCCATGTGGACCAAGGGACAAGTACCTGCGCAAGGAATTGCTGTGGCCTCATATCCAGGAGTTTGTAGATGGCGCATTAGCTCATATCCTTAACATGTCGAGGGTGCTGGGGGAACAGATCGGTGGCGGTCAGCCTGTGTGGCCATATGTTATACATGGCCATTATGCAGATGCAGGAGACTGTGCTGCTCTTCTTTCTGGTGCTCTGAATGTTCCAATGGTCCTTACTGGCCACTCACTTGGGAGGAACAAATTAGAGCAACTTCTCAAACAGGGCCGTCAATCAAAAGAGGACATAAATGCCACTTACAAAATCATGAGGAGGATAGAAGCAGAGGAGCTCTCCCTGGATGCTGCTGAACTTGTAATCACGAGTACCAAGCAGGAGATTGTAGAACAATGGGGTTTGTATGATGGTTTTGATGTCAAGCTTGAGAAGGTCTTGAGGGCGCGGTCTAGAAGGGGAGTCAAGTGCCATGGCCGTTACATGCCAAGGATGGTGGTTATTCCACCTGGAATGGATTTCAGCAGTGTTGTAGTCCAGGAAGAGGCAGCTGAAGCAGATGGAGAAGTTGCTGCACTCATAGGTATAGATGGGACCTCTCCTAGGGCACTGCCACCAATATGGTCTGAAGTAATGCGCTTTCTGACAAATCCTCACAAGCCGATGATCCTAGCCTTATCAAGACCTGACCCCAAAAAGAACATCACTACCCTTTTAAAAGCATTTGGAGAATGCCGTCCTTTGAGGGACCTTGCAAATATGACACTGATAATGGGGAACAGAGATGACATAGAGGAGATGTCCTCAGGAAATGCAAGTGTACTCACCACAGTTTTGAAGCTGATTGACAAGTACGACCTGTACGGACTCGTTGCTTACCCAAAGCATCACAAGCAGAGTGATGTTCCAGATATTTACAGGCTGGCAGCTCATACGAAGGGAGTCTTCATCAACCCAGCCTTGGTTGAGCCATTTGGCCTTACGCTAATTGAGGCTGCTGCACATGGGCTTCCGATGGTGGCCACCAAGAATGGAGGTCCAGTTGACATTCATCGGGCTCTGAACAACGGGTTGCTGGTTGACCCACACGACCAGAAAGCCATAGCCGACGCACTTCTGAAACTTGTAGCAGACAAGAACTTGTGGCACGAATGCAGGAAGAATGGGTGGAAGAATATACACCTCTTCTCATGGCCTGAGCACTGCCGGACCTACCTCACAAGGGTAGCAGCCTGCAGGATGAGACACCCACAGTGGCAGATGGACACACCATTAGATGACATGGCTGCAGAGGAGTCCCTAGGAGACTCCTTGAAGGATGTCCAGGAATCATCTCTCAGGCTTTCTGTTGATGGGGAGAAAAACTCCCTTAATGACTCCTATGCAGATGATTTAGAGAGAGCTGCAGCACAGGAGGGTGACCCTGAGCCACAAGATCAAGTAAGGAAGATACTTAACAAGATCCGGAGGCCATCTTCTGACGAACAGACTGCAGATAAGAAGGGCGAGATCACACCGCTCGCTTTCAGCAAGTACCCACTCCTCAGGAGACGACGGCGGCTGTTTGTGATAGCTCTTGATTGCTATGATGATGCGGGCCGGCCCATTAGGAAGATGCTTCAGGTAATACAAGAAGTCTTTAGGGCTGTCAAGTTGGATTCTCAGCTGGCTAGGATATCAGGATTTGCTCTTTCCACGGCTATGCCCATCTCAGAAACACTGGACCTATTGAAGTCGGGTAGAATACAGCCTACAGAGTTCGACGCTATCATCTGCAGCAGTGGAAGTGAAGTGTACTACCCTGGGACATATAAATGCACCCAAGACGGCGAGTTCTGCCCGGATCCTGACTATGCCTCACATATAGAGTATCGCTGGGGTTATGATGGTGTGAGGACGATTATATCCAAACTGATGAACTCAGATGACGGTCCATTGGATGGTAAACCTGAGAACTCTTCTAATGTTGTTGAAGAAGATGCAGCGTCGAGTGGTGATCATTGTGTCTCGTTCTTGATCAAAGACTCTTCGAGGGCAAAGAAAGTGGATGATCTGAGGCAGAAGCTAAGGATGCGTGGGCTCCGCTGTCATCTGATGTACTGCAGGAACTCCACCCGGCTGCAAGTTATTCCTCTTCTTGCATCGCGGTCTCAGGCTCTCAGGTACCTTTTCGTCCGCTGGGGCCTCAACATCGCAAACGTATTTGTCGTCCTCGGCGAGAAGGGCGACACCGATCATGAAGGAATGATCGCCGGGTCTCACAAGACGATCATCCTGAAAGGGGTTGTGGAGAAGGGTTCGGAAGAGCTGCTGAGGACATCCGGCAGCTACCACGGGGACGACGTCATC

>TRINITY_DN25039_c0_g2_i1_2
TTCTGATATCTTTAAGTAGGCATGTATGCAGAAATTTTTTTAAATATTAATTTTTTCTTCTGTTCATTTGAGCCTTGTGTTAATGTGAACTTTTTCAGTATGCTTTCGTTCACTGTTGAGTTTTTCTACAGGAAAAGTATGTGTTTCAGCACCCACAACCAAAGAGACCCAAATCACTGCGGATTTATGAATCACATATTGGAATGAGTAGTACGGTATGCAACCACCTTTTGTGTACTTTTTATGCTGTAGCTCTGATTAGGAGGATCAAAGTGGTTACACTTGTTGCTGAGTGTTCTGTCTTCTCTAACCAGATTGGAAATCCTAGATTGTTGTATACTTGTATGAATAGTGAATTTCAATCTTCATGTGACTCATTAAAGCAAGTATAGATTTATGCTGAACTGATATCTTTCCATTTTTTTATTCTTTGTATATTGTACTGTAACATATTAAGTTCTGCACATCATGGTTATTTTTCTTGTTCATCCCAGATATCTTATTAGAATTGACAAAAGGTCATGCTGACCTTGTATCACCTTTTTGGTTATATGGTTGACACTATGGAGGAAGGCATCTGCCTAATCATTAGTTTTTTCTGCACAGAGATCATATTGATTCTTTCTATAAATTTTCTCTACTTATATCTTAATTTGGGGTTTTTTGGCTGTACCCTGATTCTAGTGACGGTGCCTTTTGTCTTGATATTCATTGTGCATGCAGGAGCCAAAGATCAACACTTACGCCAGTTTCAGGGATGATGTGCTTCCTCGGATTAAGAAACTAGGATATAATGCTGTACAAATTATGGCTATTCAAGAGCACTCATATTATGCTAGCTTTGGGTA

>TRINITY_DN25890_c0_g3_i1_3
TGTTTGTTCACAAGTTTCATTATTCCTGCTTACTAGTCTATGTAGCATGCAGTAGAACTTTACTAATGATTGCAGATGTCAATTACTAGAAGATGCAAAACATCATCTCTCAAGTAATTAGCGATATTTTCTCAGAGCCACAACGTTTCAATGGAAGGGTTTGAATCCTCTTCTTAACTGTTCTATCTTATATTCAGGGAGATGCAGAGTATTTGAGGTATCGAGGGATGCAAGAGTTTGATCAGGCTATGCAGCACCTTGAAGAGAAATATGGTGTACGTTTCTGGACTGCACTTGCCTGGCATATTCCTTTTTTTCCCCATCATTTGAGAAATGGAAGAGACCTCATATGAACATCTTGCAGAAGTTGTGGTTGATGGAGACTTGTAGTTTTTTCTTCCATGTGGTGGATCTGTTTGTAAATATTGACATTTGGCAAATTAACAGTTATTATTTTTAGATTTCCTTTATCATGTTGTTTTATCCTTTATATGGAACATTTAGAGTGCTGACAAATGGCCTTATCCTTTTTCTCCTTTCATGTATTATGGATACTTGTGCAGTTTATGACGTCAGAGCACCAATATGTATCTCGTAAAGATGAAGGAGACAAAGTGATTGTTTTTGAACGAGCAGACTTAGTGTTCGTCTTCAACTTT

>TRINITY_DN27715_c0_g1_i15_1
GTAGGAATATTAGGGTATGCCTTTTTTCAAATAATTATATATATTCTAATTCATTTTAACAAGAAGGGAGTAATCTACGCCGGTAAGGACCATTAGGATTACACTGTGTACGTGAGGATCAGATTGGATCAGCTGATCCAGATTGGCCAGCCATCTGCCAATCCATTCCCATTTCCTGATTCCTCCTTTCTTCAATGGACTTAGCTAGGACTTGGACACATGTCCAACATACACTTGTAACTCACCTCAATACTTGAACTCCCCTTGCCCACATGCCTATGGCCTAACACTTTCATTGAAACTTAATATTCTGTCCCTAATTTAATAGTTTAATGATAATCACTGACCTGATATCTCTATCTTGAACTGTACACCTTGTGTCCAGGAATATACCTACAATGACTAAAATATTCTTAAATTTTAAGACTTAATATAAGGCAAATAAATCACAGAAGAGGTATGGGGTATGCTATATTAAGACAAGGTCAAGTTCTGGCTTCGTATCTGTTGGGAATTTGAAGATCTTTCTTTTGTGTGGGTAGAATTTAATGGATGACAATGGGTTGATAGGTTACCTTTTCTGTTGGTTCACGGTGGGGTTAATAGTTTAGGCTTTTTCTCTTGTGAATATTTCTTTTGGTGATACCAATGCTGTATAATTGCCAGAAATCTATTGTATTCCCATTTTTGTGTATGAATCTGATAAGTTGAGAACCAATTTTCGAGATAAAAAATATTTGTTGATACACTCGTATCTTCATCTTCCTTTCTGTACACACCTTTAGTGGTACTACACTTTAGAAATAAGTGGAGAAAAACGTTCTCCACCAGATGTCTTTGTGGTTACATTTAATGGCTATTTGAAAATTAAGTTTTCTTATTGAAACTATAAAACCTTATTCTGAACTCTGTTAACGTATGCATATGATTACTTCATCATGTTCCATTTTCTGCCTCCCTTTCTTTCTCTCTCTGGAGTGGGTGGCAAGGCATCCTTTTCCATTAAAACGTAATGCATAAAATTGTAATCTAAGCTTCCTACATTTGGGAACTTGACTGATGCTGAATATTGGGCTAAGGTGCCTTCAGGCATCTTTTCAGGAGAGCCCGTTGATGCCCAGGATATTTGTCCAAGTTTTATTCTTGTCACCAGTTCCAAGCCTCCTTCCTTCTTAGAAGGTTTACGAAGATGAAACGTGTGGCATATGCTATATGCATATAATTTCTTCACCATGTTCCATTTTCTACCACCCTTTCTTTCTCTTTCTGGAATGGGGTGGCAAGTCATTCTTTTCCATTAAAACATAATGCATAAAGTTGTAATCTAAGCTTCCCACATTTGGGAACTTTACTGATGCTGAATATTGGGTTAGGATGCCTTCGGGCATCTTTTCAGGAGGACCTATTGATCCCCAGGATATTTGTCCAAGTTTTATTCTTGTTACCCGTTCCAAGCCTCCTTCCTCCTGAGAAGGTTTACGAAGATGAAACGTGTGGGGATATAAAAGTGGGGTGATTGGGGGAAGTAGATTCTAAGAAGGGAATGGAAGAAACTCTCGCCTATTCTATTATTCAATTGAGAATTGCATAAGGAATTGGGCAAGGTCTATTACCATGAGAAGAAAAGGAATTTGACTCATCATACCTGACATTTAGATTGCAAGGAAAATGAGGCATGAAATGATCTTCAATTTGGCAGGTTTTTTAGGACAGATTAAAAGCTGTTTTCCAGTGTTCTGGTAATCTCTTCTTCTGATTCTGACTGGTAATTCATGCTTAAAGTTGCTAATCAAGGAATCTTCTTTAGAGCCCAGTGAAGCAAATTGCTTTTCTGTGAACTAGAAACTGACCAATAGAGTTACTCAGTTACATGAGGTTCTAATTTTTCTAGGCCATGTTACATATCTAGGATGGCAATTGAAAAGTTTGTCGTTTATATAAAGACATGGGTTTTTCAGTGTGTATTATTGGACTATAGTTCTAGTAATTATTTTACCTTATCTTTATCCATTGGCTGCCATTGAAAGGGGGGACAATGCATCAAAATGCCTCATTCATGTGTTTAAATTACTAGGCTCACTGTGATTGTCTCAATGCTCACTTTCAAAAAGGTGATTGTCTCAATGCTGATTGGCAGATACACATGGAGACACCTACTGGAATAAAGGATTCTATTCCAGCATGGATCAAGTTCTCTGTACAGGCTCCTGGGGAGATTCCATATAATGGGATCTACTATGATCCCCCCGAGGAGGAAAAGTATGTGTTTCAGCACCCACAACCAAAGAGACCCAAATCACTGCGGATTTATGAATCACATATTGGAATGAGTAGTACGGAGCCAAAGATCAACACTTACGCCAGTTTCAGGGATGATGTGCTTCCTCGGATTAAGAAACTAGGATATAATGCTGTACAAATTATGGCTATTCAAGAGCACTCATATTATGCTAGCTTTGGGTACCATGTCACAAATTTTTTTGCACCTAGTAGCCGTTTTGGAACTCCTGAAGATCTCAAGTCATTAATTGACAGGGCTCATGAGCTTGGTCTTCTTGTTCTCATGGATATTGTTCACAGCCATGCATCAAACAATGTTTTGGATGGGCTGAACATGTTTGACGGAACAGACGCACACTATTTTCACTCTGGTTCTCGTGGGTATCACTGGATGTGGGATTCTCGTCTATTCAACTATGGAAATTGGGAAGTTTTGCGGTTTCTTCTATCGAATGCAAGATGGTGGCTTGAGGAATACAAATTTGATGGGTTCAGATTTGACGGGGTGACCTCCATGATGTATACTCACCATGGGTTGCAGGTAGCTTTTACTGGGAATTACAACGAATACTTTGGATTTGCTACTGATGTTGATGCAGTGATATATTTAATGCTAGTAAATGATCTCATTAAAGGGATATTTCCAGAAGCTGTTTCTGTCGGTGAAGATGTTAGTGGAATGCCAGCATTTTGCATTCCAGTTAAAGATGGTGGTGTTGGGTTTGACTATCGTCTTCATATGGCCATTGCTGACAAATGGATTGAACTTCTTAAGAATAGTGATGAGCATTGGGGAATGGGTGACATTGTTCACACACTTACGAACAGAAGATGGCTAGAGAAATGTATCGCTTATGCTGAAAGCCATGACCAAGCTCTTGTTGGTGATAAGACAATTGCATTTTGGCTGATGGACAAGGACATGTATGATTTCATGGCTTTAGATAGGCCATCAACTCCTCGCATCGACCGTGGAATAGCATTGCATAAAATGATCAGGCTAATTACGATGGGTTTAGGTGGAGATGGATACCTGAACTTCATGGGAAATGAATTTGGGCATCCCGAATGGATAGATTTTCCAAGATGTGAACACCAACTTTCAACTGGCGCAGTTATTCCAGGGAATAATAACAGTTACGACAAATGCCGCCGTAGATTTGATCTTGTAAGTTTCAAAAAGAAAGTTCCTAGGGCCAATTTAAATAAGGTGCCTTGTTGATTTGTTTCGTAACTTCCAAGTACTAATAGCACATTAGCACTGATCCTGTACTGGATGCTTAAACAATGCTTTAAAGTCTGTCAAATCTAGAACAATAATATATGTTGATTTACTCATCTGGATTTCTAGCATATGAAAGCCTTTTATATGAAAGTTCAGTTCCCAGTTCTAGTACATCGTGAAGTTGTGACTGATGTTCAAAGATATTTAATCATTTGGTAGAAACCACCTAATTTTCATGCTAAGAAGATTCTGAGTTCTGAAATATTGGAAAATAATAGATATAGAAGATTTATTATTCGGGTAGGGATGGAAGCTACATGACGGGGGAATCAAGTTTCCAGATTGCTTATTTGGAAGTGATTCTTTGTTTGTTGAAATGTTCCGTACAGCTGGTAATCGCCCCTGGAAAAATATAATATGCGCCAAGAAAATTATTTTCTTGAAATTGGTAATACTCCTGTTTTCATCAAAAATGAAAAGGAATGTGTCAGCATTGAGGAAACAACAAAAGCTCCCCTTTACAAATTGTAAAGCAAAAACCTTGTGGTAGCCTCAGAACAAAGCGAAAAGAGAGGATTTCCACCTGCCTACTTACAGAAGTGTAGACGCACATTTCCATCACTCACATACAAATCCGACAATCTTTTCTGAGTCACTGGCTTCTTTCTTAAAATCCAGTGCCAAGAGAAGCAATGAATGTAATGGATATAGAATTTCCTTTTTCTCTTGATCAACCTTAGCTGAATGATTGTAGAAAATCCGATTGTGTGTAGTGGTGGGATCTCTTGACTGCTTTTCAGTGACATGTTTAGGAAATAGAAATCCGCTTCCAGGAAGAGGGGAGGAAGTGGTCGACACCAGTTCCTCTGCAGCTCTTCCCCAAGTGCTGCATTCGCGGGAATGGTTCCTTGAGCATGAGTTAGCTGTTCAGGAAGCAGAAACCTACAGAATGCTGACTATTCATTTACACCTGAATTTTGACCAAAATACTTCCCCAAGTGCTGCATTCGCAGGAATGGTTCCTTGAGCATCAGTTAACTGTTCAGGAAGCAGAAACCTACAGAATGCTGACTATTCATTTACACCTGAA

>TRINITY_DN27715_c0_g2_i2_1
ATTTGTTTTACAATTACATTTCAAGGTTCACATTTTTTTTGAGAAGTTCAAGGTTCGCATTTATGCCCCTGATTTCATGAAGTTTATATTTTTCTCATCGTTGAAAATTTAGATCTACAGCCCAATCTATCAGAATTGTTTTGATGCCCCAGTCAGCTCTATTGCATCATCAATCTTAAAGTTCATAGTTATTATCCACAATTATTTATCATTGAAGACAGGGTTTCATTATGATGGAAATAGGTGTGAAGATAGAAGAAAGATAAGTGGTTAAGAAAATTCACCCCAAAGCATGTGCTTGAACACAACTTTATATTTTCATCAATTAACTAAAAAATTCTGGTACATTGGATGGTTTCTTGAACTTATTATTATTATTATTATTATTATTATTATTATTATTATTTTCATAAGCTGTTTTTTTATGTTATAGAAAGTAGAAACCTCAATAAGTACAATCTTGTCTGTGGTTTTTTGTGAGAATCAAGTTTATTTGCTTGAATGATTAGCAGAACTATTAGGAATTATTTGATTCTGATTCCTTTTAGTCATTTGCAGTGAAACAGGCATCACTTACAGAGAATGGGCTCCTGGAGCAATGTCGGCTGCATTGATAGGAGACTTCAATAATTGGAATCCAAATGCAGATGTTATGACACAGGTATGTTCTATGGTTCTTTCATATTCGCTTTTTGACACCCAAACCTGTGGCCCACTTAACATCAGAGATGACTCATAGTCATTCATACTGATTTTCAATTTTCTCTTCCCATGCATAACTTCAATTTACTCCAAGGATCTTACTTTTGTCATTGTCACAATGTAAAGTACCTGCAAAGAAGCTCACTTCTCAAAAAAAAAGTACCTGCAAAGAAGCTGTAGAAATATGGTCTAAAATATGTCACATCCATTCTTCAGAATGAAGAGTAGTGACAGCAGAACCCACATCAACATGAACTATCTCAGACATATTGTAACAAATTTAATTTTCTAAACATATGATAACCATGATTGCAG

>TRINITY_DN27415_c0_g1_i2_3
TTGGGTTGCTGTCTCCCATTTTGATCTCTATCGTCCAGCCGATCCCCACAAGGGAATCCGTGGCTTCCGGCATTCTTGCTGACATCTGCCTCAGAGCTTACCTCCCTACTTGTTCCTGCTCCTCGCCCCCCTTCGTGGGTCCCCCGCCCCCCTTCTCTCTGTGCTCATCAGTGTCCTCCAATTTTCCTTCTCCTCCTCCCTGCGGCAGGGCAGGCACACCACGGCGAGATTTCTCTGCATTCCCCCTCCTTCAAAGGGGGTTCCTTTCTGCCCGGCAGTCGGGGGAGCAGTACATGACACCCTTTTGATTTGTTTTCCTGGGAGGCCCAGCTGAAGGTAAGGAGGAAAAGAGGAAGAAAGAAAGATGGCATCTTTGGGGCGAGCTGGGGCGGCTGTTTTCTTCCTGGATCCGTCAAATGAGAAGTTTTTTGGCCTCGGCAGGAGCGGCGGGCGGCCGCGGGCTGCTGGGTTCCGTGTGCCTGTCCGGTGCAGGTCGAATTGTCTTGGTCTCCATTCGGGCGCTGGGGAATTCACGTTTCTTAGGGTGGGCAATGGCGAAGGGCACATGGGTTTTACATGGAGGGACAGGGGAACGGCTAGGGTTGTGCCGGCTGTGGGAAGAAAAGGTGCTGGCAAGGGAGCTGAGGAAGGCGGCGAGGACGATATGGGCGATGTGGATGTGGATGGCGACGAGGTGCTTAAGGCGACTATCGCAAAGACCAAGAAGGCGCTGGCCGTGCAGAAGACCCTGCTTGAACAGATTATTGAAAGGAGAAAACTTGTTTCCTCCACAAAAGAGAGTGTCATCAAGACGGGAAATGATGCAGTCTCCTCTACTAAAAGTGATGCCTCTTTCCCAAGATTGAGCTCAGATACCACAAGTGATAGGGTTGGTAATAAATATAAGAACATTTCTTACCCCACTTCTGAAACAGCTGAAACTCTGTCTGCCAGTACCAATGGGTCTGCTATAAAAAGGAAATTCCAGAATGAACAAGTGGAAGGCTCTGCCGATGCTAAAGAGGTTTCCATGATTAGTTCAAAAGTAGAAAGCTCAGATGATAAAGAAAAGGAAAATGAAGATGGCTTACCTGCAGAAAGCTTGATAGCTGAATCTCTTGGACTGCTAAGAGAACCCAAATCAAAAGTTTCTCCGTCTAAGACAGTGATGCCCTCTTTACTTCCAAATGTGCCTGAAGCTCTCACAGCCAAAAATGAACAACTTGAGGAATTGGAAGAACACAAGAAAGATGGTCTAGATGATCAAACTGATGTAGCTCCCAAGGAAGAAAATGTAAAACCTCCTCCATTGGCTGGAGTAAATGTTATGAATGTTATCTTGGTAGCTGCAGAATGTGCTCCTTGGTCTAAAACTGGTGGGCTCGGAGATGTTGCTGGAGCTTTACCAAAGGCCTTGGCCAGACGTGGGCATAGAGTCATGGTTGTGGCTCCCCGATATAGCAACTATTCTGAACCTCAAGCAATAGGTGTGATCAAGCGTTACAAGGTGGATGGACAGGATATGGAGGTAACATACTACCATACCTACATTGATGGTGTGGATTTCGTTTTCATCGATAGTCCTATTTTTCGCCATCGTGATAACGACGTATATGGTGGGAAACATGTGGATATTTTAAAACGAATGGTCCTTTTATGCAAGGCAGCTGTTGAGGTTCCATGGCACGTTCCATGTGGTGGAGCCATCTATGGTGATGGAAACCTTGTTTTCATTGCAAATGATTGGCACACAGCACTGCTTCCGGTTTATCTAAAAGCATATTACCGTGACAATGGATTGATGCAATACACCCGGTCTGTCTTGGTTATACACAATATAGCTCACCAGGGTCGTGGGCCAGTAGATGATTTTAGCATTGTGGATTTACCTGGTCATTATGTAGATCTCTTTAGGATGTATGACCCAGTTGGTGGTGAGCACTTAAACATCTTTGCTGCGGGCTTGAAAACTGCTGATCGTGTAGTCACAGTGAGCCATGGCTATGCATGGGAGCTTAAAACAATTGAAGGTGGCTGGGGACTACACCAGATTATCAATGAGAGCGACTGGAAACTTCGAGGTATTGTGAATGGAATCGACACAGAAGAGTGGAACCCGAAAGTTGATGTGCACTTAAAATCTGATGGATATACCAACTACTCATTGGAGACAATTAATATGGGTAAGCCTCAGTGCAAGGCTGCACTGCAGAAAGAACTTGGCCTTCCTGTTCGACCTGAAGTTCCTCTTGTTGGCTTTATTGGGAGGCTAGATGGTCAAAAAGGAGTGGATATCATTGCAGAGGCCATTCCCTGGATGGTCAGTCAGGATATGCAGTTGGTCATGCTCGGTACTGGAAGGCCAGATCTGGAGGACTTGCTCCGAAGTTTTCAGGGCAACTACCCTGACAAGGTCAGGGGTTGGGTTGGTTTTTCTGTTAAAATGGCGCATAGGATAACTGCAGGTGCAGACGTCCTGTTAATGCCATCCAGGTTCGAGCCGTGCGGCCTGAACCAGCTGTATGCTATGATGTATGGAACAGTTCCTCTTGTTCATGCGGTTGGCGGACTGAGAGACACTGTCCTGCCATTCAATCCGTTCGAGGAGACTGGATTTGGGTGGACATTTGACAGAGCAGAGACAAATAAGCTGATTAACGCCATGGGCAACTGTTTCAACACTTACTGGAATTACAAGAAGAGCTGGGAGGGGTTGCAGAGCCGCGGTATGTCCCAGGACCTTAGCTGGGATCATGCTGCTCAGCTATATGAGGAAGTCCTTGTTGCTGCCAAGTTCCAGTGGTGAAAGCTTTCAGCAAATGACCAGTTCACCTGGGGCCGATCAAATTGGCCATCCAGTGATCCTTTTTGGTAGGTCTTTGCTAGGGCGTAGCTGCTAGAGAAAGCCGCCACCACCTCCTGATGTGTACTGCTTGTTCCTTATTTTGCTACTTTCGTAATCTTCCTCCATTTTTACTAGGTTCACCACGGATCGTGAGTACTGCGAGTCTCGCATCCGTGTAGTATGGCAAATTTCTCCCTCCTTTGTAGTACTCTGAATCATTAGTTTTGTTGGCTTTTTTGCCTGGCCATTGTTTCTAAGAGATGTACATATCTGTCTCCTAGCAGCACTATTAATACGTAGCAGGCACCTTAAACCCTAAATCCTAAACCAACAGGGGCAAATGCAGTAAATATTTGCTGCACCTTGT

>TRINITY_DN30069_c0_g1_i1_1
TTGAACGAGAATATAGCACTTGATAGAAATGCATGGCGGAACGGGATTCATGTAGCCGACCCCATTTAATGGGGTAAGGCTTTGATGATGATGATCATGATAATGTTTTCTAAAGGGTACAAATTTTTCTTAAAAATTGAAGGAGCAACCACCAAACGCGGTAAAGTGTCTTATATGTTCCAAATTTTAAATTTAAGAGAAAAGAGGAAAAAAGTGCAACCAAACACCCTTTAAACCTTGCACGATTTTGCACATTCAAAATTTTGCCCTACCCATGTCTGCAAACAGTCTCTACTTTGTTGTCTTTGTTTGCTGAGCATTGCACAAAAGTATGAAGCTTTTCTTTGCTATATTTAAGATGTTTGAAAATCAGAAGTTTTAGCTAGTCATATTCAGTTGAGATTATTCTTGCTTGCAACAGTTGAGAAACATTATTGAAGAGAATGAGCTATTCTGTACAGGTTCCATGGCACGTTCCATGTGGTGGAGCCATCTATGGTGATGGAAACCTTGTTTTCATTGCAAATGATTGGCACACAGCACTGCTTCCGGTTTATCTAAAAGCATATTACCGTGACAAT

>TRINITY_DN30069_c0_g2_i4_1
CCTCCCCCTCTCTCTGTGCTCATCAGTGTTCTCCAATTTTCCTTCTCCTCCTCGCTGCGGCAGGCCAGGCACACCACGGCGAGGTTTCTCTGCATTCCCCCTCCTTCAAAGGGGGTTCCTTTCTGCCCGGCAGTCGGGGGAGCAGTACATGACACCCTTTTGATTTGTTTTCCTTGGAGGCCCAGCTTAAGGTAAGGAGGAAAAGAGGAAGAGAGAAAGATGGCATCTTTGGGGCGAGCTGGGGCGGCTGTTTTCTTCCTGGATCCGTCAAATGAGAAGTTTTTTGGCCTCGGCAGGAGCGGCGGGCGGCCGCGGGCTGCTGGGTTCCGTGTGCCTGTCCGGTGCAGGTCGAATTGTCTTGGTCTCCATTCGGGCGCTGGGGAATTCACGTTTCTTAGGGTGGGCAATGGCGAAGGGCACATGGGTTTTACATGGAGGGACAGGGGAACGGCTAGGGTTGTGCCGGCTGTGGGAAGAAAAGGTGCTGGCAAGGGAGCTGAGGAAGGCGGCGAGGACGATATGGGCGATGTGGATGTGGATGGCGACGAGGTGCTTAAGGCGACTATCGCAAAGACCAAGAAGGCGCTGGCCGTGCAGAAGACCCTGCTTGAACAGATTATTGAAAGGAGAAAACTTGTTTCCTCCACAAAAGAGAGTGTCATCAAGACGGGAAATGATGCAGTCTCCTCTACTAAAAGTGATGCCTCTTTCCCAAGATTGAGCTCAGATACCACAAGTGATAGGGTTGGTAATAAATATAAGAACATTTCTTACCCCACTTCTGAAACAGCTGAAACTCTGTCTGCCAGTACCAATGGGTCTGCTATAAAAAGGAAATTCCAGAATGAACAAGTGGAAGGCTCTGCCGATGCTAAAGAGGTTTCCATGATTAGTTCAAAAGTAGAAAGCTCAGATGATAAAGAAAAGGAAAATGAAGATGGCTTACCTGCAGAAAGCTTGATAGCTGAATCTCTTGGACTGCTAAGAGAACCCAAATCAAAAGTTTCTCCGTCTAAGACAGTGATGCCCTCTTTACTTCCAAATGTGCCTGAAGCTCTCACAGCCAAAAATGAACAACTTGAGGAATTGGAAGAACACAAGAAAGATGGTCTAGATGATCAAACTGATGTAGCTCCCAAGGAAGAAAATGTAAAACCTCCTCCATTGGCTGGAGTAAATGTTATGAATGTTATCTTGGTAGCTGCAGAATGTGCTCCTTGGTCTAAAACTGGTGGGCTCGGAGATGTTGCTGGAGCTTTACCAAAGGCCTTGGCCAGACGTGGGCATAGAGTCATGGTACTCATTTCTTCCTTTGCTTTAACCTTTAAATGCCAAATATTTGTTCTCAGATGTATAGATAACCCATTGTTCTTATGTGCTGTGTCACCCAAAAAGTTCTCTGCTCTGGTGGTAAAATGTTTCTGATCTAGCAAGTCAATGAAAATTTGCTTTGCAGATGCTGGTTATCTGAGTTTTTGCTTTCATAGTTCTGGATCTTGATTTGCCGAATTTTGTTAAATTTCTTGAAATTCCTGAAAGCTGTACAATGTATTTTCCCCCCCTCCTTTCCCTCCCCTTCCCTTGAAAGGTTGTGGCTCCCCGATATAGCAACTATTCTGAACCTCAAGCAATAGGTGTGATCAAGCGTTACAAGGTGGATGGACAGGATATGGAGGTAACATACTACCATACCTACATTGATGGTGTGGATTTCGTTTTCATCGATAGTCCTATTTTTCGCCATCGTGATAACGACGTATATGGTGGGAAACATGTGGATATTTTAAAACGAATGGTCCTTTTATGCAAGGCAGCTGTTGAGGTTCCATGGCACGTTCCATGTGGTGGAGCCATCTATGGTGATGGAAACCTTGTTTTCATTGCAAATGATTGGCACACAGCACTGCTTCCGGTTTATCTAAAAGCATATTACCGTGACAATGGATTGATGCAATACACCCGGTCTGTCTTGGTTATACACAATATAGCTCACCAGGGTCGTGGGCCAGTAGATGATTTCAGCATTGTGGATTTACCTGGTCATTATGTAGATCTCTTTAGGATGTATGACCCAGTTGGTGGTGAGCACTTAAACATCTTTGCTGCGGGCTTGAAAACTGCTGATCGTGTAGTCACAGTGAGCCATGGCTATGCATGGGAGCTTAAAACAATTGAAGGTGGCTGGGGACTACACCAGATTATCAATGAGAGCGACTGGAAACTTCGAGGTATTGTGAATGGAATCGACACAGAAGAATGGAACCCGAAAGTTGATGTGCACTTAAAATCTGATGGATATACCAACTACTCATTGGAGACAATTAATATGGGTAAGCCTCAGTGCAAGGCTGCACTGCAGAAAGAACTTGGCCTTCCTGTTCGACCTGAAGTTCCTCTTGTTGGCTTTATTGGGAGGCTAGATGGTCAAAAAGGAGTGGATATCATTGCAGAGGCCATTCCCTGGATGGTCAGTCAGGATATGCAGTTGGTCATGCTCGGTACTGGAAGGCCAGATCTGGAGGACTTGCTCCGAAGTTTTCAGGGCAACTACCCTGACAAGGTCAGGGGTTGGGTTGGTTTTTCTGTTAAAATGGCGCATAGGATAACTGCAGGTGCAGACGTCCTGTTAATGCCATCCAGGTTCGAGCCGTGCGGCCTGAACCAGCTGTATGCTATGATGTATGGAACAGTTCCTCTTGTTCATGCGGTTGGCGGACTGAGAGACACTGTCCTGCCATTCAATCCGTTCGAGGAGACTGGATTTGGGTGGACATTTGACAGAGCAGAGACAAATAAGCTGATTAACGCCATGGGCAACTGTTTCAACACTTACTGGAATTACAAGAAGAGCTGGGAGGGGTTGCAGAGCCGCGGTATGTCCCAGGACCTTAGCTGGGATCATGCTGCTCAGCTATATGAGGAAGTCCTTGTTGCTGCCAAGTTCCAGTGGTGAAAGCTTTCAGCAAATGACCAGTTCACCTGGGGCCGATCAAATTGGCCATCCAGTGATCCTTTTTGGTAGGTCTTTGCTAGGGCGTAGCTGCTAGAGAAAGCCACCACCACCTCCTGATGTGTACTGCTTGTTCCTTATTTTGCTACTTTCGTAATCTTCCTCCATTTTTACTGGGTTCACCACGGATCGTGAGTACTGCGAGTCTCGCAATCCGTGTAGTATGGCAAATTTCTCCCTCTTTTGTAGTACTCTGAATCATTAGTTTTGTTGGCTTTTTTTGCCTGGCCATTGTTTCTAAGAGATGTACATATCTGTCTCCTAGCAGCACTATTAATACGTAGCAGGCACCTTAAACCCTAAATCCTAAACCAACAGGGGCAAATGCAGTAAATATTTGCTGCACCTTGCTAGAGTGCAGCAACGTATAC

>TRINITY_DN27891_c0_g1_i2_5
GTGCGCCGAGCTTGGAGTTGGGACGGGAGCGCTCTTCCTCGCCGCCTGCTTCCGGAGAGGCTCTCTCGCGATCTCCCCTGCCTGCGGTGAAAGATCAGAGTAAAAGTCCTGCAAAATGGCGACGGTGACTGCTTCAACCATCATCTCAAGGAGTGCTCATGGTGGAGTGTCTGACGTGGAACCAAGAGTATCAGGAAGCAGAAAGGCTGCTCGCTTGAGAAACCAAGGCGCTACTTTTCGTGGGCTGACGTCTCTGAACAAAGTAGATTTTGCTACGGGGAAAGTGAGATCCACAAATGCCCTAAGGACTGGCATCCCGGCTGGGAAAGCTAGATCCTCTGGGGTCGTCGTTTGCGAAAAAAGTTTGGTCCCTGTTGAAGCAGTTCCTTGTGAAGATCCCATGAACATTCTCTTTGTATCAACAGAAGTCGCACCATGGTGCAAAACTGGGGGTCTGGGTGATGTTGTTGGAGGTTTGCCTCCTGCCTTGGCGGCGCTTGGGCACCGTGTCATGAGCGTGTCACCTCGCTATGATCAATATAAGGAAGCATGGGATACCGGTGTTCTTCTTGAGATCAAATTTGCAGACACTGTGTACACTGTCCGTTTCTTCCACTGCTATAAGAACGGTGTTGATCGTGTGTTTGTCGACCATCCTGTATTCCTGGAAAAGGTATGGGGGAAGACTGGTGGAAAATTGTATGGCCCTGTCGCTGGGGAAGATTACACAGACAACCAGATAAGATTCAGTCTGCTATGTCTTGTGAGTGCCCTATCTTACAACAACTTTTCTTTTGAACTTCTGTCTGTCTTTGATTGCTCATTCTGTAGACTTTCCAATTGTTCCCTGAAAACAATTATGTGAAATCTCTTTTACCTTTTAATTGATCAATCAATCAAGTGAGAATTTCCCATCAGGCAGATAGGCTTTTGGACTTGCAGGTGGAGTTTCTTCACAGACTCAGCTTGGACAAGAGTAGTGACTGATTACCAATTTGTCATTATTATTCAAAGTATGAATTTGATTACAATTTCTATGCAAGCAGTGGCCTACCATGAGCAATGTGCCAGAAACCTTGCTGATCGGGTTCTCATAGGATGAATTACCAGATGATATTCTCTAGTTGATTGTAGATGTTACAAAGAAAATTTCCTCCCCCCAATAGAAAGATTTATTCTTTGTTTAGGTCCGTTAGATCTTGAAATCCGCAGTTTCTCTCTTTTTCTGCTTTTCCTGCAAGCACAAATGGTCATTAAGAGTTTTTTGAACATCATTGCAGTCTGTTAAACCTTACTGTTTTTTTGCGACTGTGCTCTTCAGGCTGCTTTGGAAGCACCTCGCATTCTTAATTTGAATAACAGCAAATCCTTTTCTGGGCCTTATGGAGAAGATGTTGTATTTGTTTGTAATGATTGGCACACTGGAATTCTCCCATGCTACTTGAAGAGCATGTACAAATCGCATGGGCTCTACGAGAATGCAAGAGTAGCCTTTTGCATTCACAATATTGCATATCAAGGCAGATTTGCCTTCTCAGACTTCTCGTCTCTTAATCTCCCAGACACATTTAAGAGCTCGTTTGATTTTATTGATGGATATCTCAAGCCAGTGAAAGGGAGGAAAATTAACTGGATGAAAGCTGGAATCCTCGAATCTGACAAAGTCTTCACAGTGAGTCCATACTACGCTGAAGAGGTCGTCGCAGGTGTTTCTAAAGGTGTCGAGCTGGATAATATACTCCGTAAGACTGGTGTTACTGGAATTGTGAATGGAATGGATGTTGATATATGGAATCCAGTGACAGACAAATATATCTGTGCAAATTATGATTCTACAACTGTCTTTGATGCAAAGCGCCTTAACAAGGAGGATCTCCAAGCGGCAGTCGGGTTACCAGTGGATCCAAGCATCCCTCTCATAGCATTTGTTGGTAGACTTGAGGAACAGAAAGGTTCAGACATCTTAGTTGCCGCCATTCCGAAGTTCATTCATGAAAACATTCAGATAGTGGTCCTCGGAACTGGAAAGAAAAACATGGAGGAGGAAGTTACGAAGCTCGAGGAAGATTATCCAGACAAGGCAAGAGGAATAGCGAAATTTGATGCTGTCTTGGCTCACAAGCTTTTTGCTGGAGCTGACCTTATGATAATTACCAGTAGGTTTGAGCCATGTGGGCTTATTCAGCTGCAGGCAATGCAATATGGGACGCCATCTATCTGTACCAGCACCGGTGGGCTAGTAAACACTGTCGTTGACGGCGTCACGGGTTTTCATATGGGAGCCTTTAATATCGATTGTGACGCTGTAGACCCGAAGGATGTAGAGGCAGTTGCCACATCTGTGCATAGAGCAATTGGGGTCTTTGGGACAGATCAATTCTCTCAGATGATCCAGAACTGCATGGCTCAAGATCATTCCTGGGAGGAGCCTTCCAAGAAGTGGGAAGCGGAGCTACGAGGCCTCCTTTGTGCCCCAAGCCAAGTTGAGGCGCCCGAGATTGAGATAGAGGAATCTCTTGTTGCTGAGAAAGTCGCTGTCTGAAGGGATGAAGAAGAGCCAGCTTTTCTAAAGCTTTTCAGTTTTCATGTACATCGCCACTTTGGAGGGAATCCGCAAGCTGAAAGTAGTTGTCGCCTGCTCCCTTGTTTTGTAATTTCTAATTTCTATTTTACGCTCTCTACCCGTAGGATGAAGATGGAGGCTGAGGTGCCGTCGAGGCTGCTTGTTCTTGAAAGCCTCCTCTTTTGGACTTTGAAATAATGAAGGATTTTACATCTCAGTAAGAGTGTTTAACTATGGTTGCACCAAGTTGGTAGCCACATCGTGCAATGACTTGCCATCGGACCTGTTCTCAAGTCTTCAAGAACGTTTGTTATTAGGACTTGGTGCCCTCCAAGGCGGGTAATAAAACCCTT

>TRINITY_DN27951_c1_g1_i1_3
GCGCCGAGCTTGGAGTTGGGACGGGAGCGCTCTTCCTCGCCGCCTGCTTCCGGAGAGGCTCTCTCGCGATCTCCCCTGCCTGCGGTGAAAGATCAGAGTAAAAGTCCTGCAAAATGGCGACGGTGACTGCTTCAACCATCATCTCAAGGAGTGCTCATGGTGGAGTGTCTGACGTGGAACCAAGAGTATCAGGAAGCAGAAAGGCTGCTCGCTTGAGAAACCAAGGCGCTACTTTTCGTGGGCTGACGTCTCTGAACAAAGTAGATTTTGCTACGGGGAAAGTGAGATCCACAAATGCCCTAAGGACTGGCATCCCGGCTGGGAAAGCTAGATCCTCTGGGGTCGTCGTTTGCGAAAAAAGTTTGGTCCCTGTTGAAGCAGTTCCTTGTGAAGATCCCATGAACATTCTCTTTGTATCAACAGAAGTCGCACCATGGTGCAAAACTGGGGGTCTGGGTGATGTTGTTGGAGGTTTGCCTCCTGCCTTGGCGGCGCTTGGGCACCGTGTCATGAGCGTGTCACCTCGCTATGATCAATATAAGGAAGCATGGGATACCGGTGTTCTTCTTGAGATCAAATTTGCAGACACTGTGTACACTGTCCGTTTCTTCCACTGCTATAAGAACGGTGTTGATCGTGTGTTTGTCGACCATCCTGTATTCCTGGAAAAGGTATGGGGGAAGACTGGTGGAAAATTGTATGGCCCTGTCGCTGGGGAAGATTACACAGACAACCAGATAAGATTCAGTCTGCTATGTCTTGCTGCTTTGGAAGCACCTCGCATTCTTAATTTGAATAACAGCAAATCCTTTTCTGGGCCTTATGGAGAAGATGTTGTATTTGTTTGTAATGATTGGCACACTGGAATTCTCCCATGCTACTTGAAGAGCATGTACAAATCGCATGGGCTCTACGAGAATGCAAGAGTAGCCTTTTGCATTCACAATATTGCATATCAAGGCAGATTTGCCTTCTCAGACTTCTCGTCTCTTAATCTCCCAGACACATTTAAGAGCTCGTTTGATTTTATTGATGGATATCTCAAGCCAGTGAAAGGGAGGAAAATTAACTGGATGAAAGCTGGAATCCTCGAATCTGACAAAGTCTTCACAGTGAGTCCATACTACGCTGAAGAGGTCGTCGCAGGTGTTTCTAAAGGTGTCGAGCTGGATAATATACTCCGTAAGACTGGTGTTACTGGAATTGTGAATGGAATGGATGTTGATATATGGAATCCAGTGACAGACAAATATATCTGTGCAAATTATGATTCTACAACTGTGGGTTCCTCACTGTCACTTTTGCTCAAGCTTCTCTTTTGGAATCCTTTTACCCTCATTCATAGGCCTGTATGTTAATCTGTGTTTACGAATTGGCATGGTCAGGTCTTTGATGCAAAGCGCCTTAACAAGGAGGATCTCCAAGCGGCAGTCGGGTTACCAGTGGATCCAAGCATCCCTCTCATAGCATTTGTTGGTAGACTTGAGGAACAGAAAGGTTCAGACATCTTAGTTGCCGCCATTCCGAAGTTCATTCATGAAAACATTCAGATAGTGGTCCTCGGAACTGGAAAGAAAAACATGGAGGAGGAAGTTACGAAGCTCGAGGAAGATTATCCAGACAAGGCAAGAGGAATAGCGAAATTTGATGCTGTCTTGGCTCACAAGCTTTTTGCTGGAGCTGACCTTATGATAATTACCAGTAGGTTTGAGCCATGTGGGCTTATTCAGCTGCAGGCAATGCAATATGGGACGGTGCGTTTTCTTGACTAGGCACATAATATGCATGGTATATAATAAAGGTCATTTTGCAAGCTGTCATGAATAGTATGCTTGGGGAGGACCATCAGCTTTTGGGTTTCCATAGTTGGCACTTCATGTGACCCCTTGAATATCGATTTAAAGGTAACAAAACTGATCTGTGTTATTTTGCAGCCATCTATCTGTACCAGCACCGGTGGGCTAGTAAACACTGTCGTTGACGGCGTCACGGGTTTTCATATGGGAGCCTTTAATATCGATGTAATGCTTGCCTTCTTAGCTCTTTCCAATTTGAATTTATTTTGTTTTCTTCTACAAGACCCAGCATGACACGGAACATCTCTGTTTTGGTCTTCCACAGTGTGACGCTGTAGACCCGAAGGATGTAGAGGCAGTTGCCACATCTGTGCATAGAGCAATTGGGGTCTATGGGACAGATCAATTCTCTGAGATGATCCAGAACTGCATGGCTCAAGATCATTCCTGGGAGGTACACACCACTATATTTTAAGCCCCTTTCATTCGATTACACCCAAGAAACTTTTGAACCAAATGCTCATTAATTACCTCTGCAACTTGCGTGCCCCAGGAGCCTTCCAAGAAGTGGGAAACGGAGCTACGAGGCCTCCTTTGTGCCCCAAGCCAAGTTGAGGCGCCCGAGATTGAGATAGAGGAATCTCTTGTTGCTGAGAAAGTCGCTGTCTGAAGGG

>TRINITY_DN29934_c1_g2_i2_1
CGCCGAGCTTGGAGTTGGGACGGGAGCGCTCTTCCTCGCCGCCTGCTTCCGGAGAGGCTCTCTCGCGATCTCCCCTGCCTGCGGTGAAAGATCAGAGTAAAAGTCCTGCAAAATGGCGACGGTGACTGCTTCAACCATCATCTCAAGGAGTGCTCATGGTGGAGTGTCTGACGTGGAACCAAGAGTATCAGGAAGCAGAAAGGCTGCTCGCTTGAGAAACCAAGGCGCTACTTTTCGTGGGCTGACGTCTCTGAACAAAGTAGATTTTGCTACGGGGAAAGTGAGATCCACAAATGCCCTAAGGACTGGCATCCCGGCTGGGAAAGCTAGATCCTCTGGGGTCGTCGTTTGCGAAAAAAGTTTGGTCCCTGTTGAAGCAGTTCCTTGTGAAGATCCCATGAACATTCTCTTTGTATCAACAGAAGTCGCACCATGGTGCAAAACTGGGGGTCTGGGTGATGTTGTTGGAGGTTTGCCTCCTGCCTTGGCGGTAAGCAGTAATTCTCAGCCGATTTTGCTAATTATGGTCAAGTGCGTATGATGAAAAATGGCTCTTTTTCCGCATTTCTTCCTTCAGTCTTTTATATCCTTTGATTTGCTTGCTTGAAATACCAGTGGATGCTTTACATTACAGTTTACGTTGCTGAGTCGTCCGTTGCTAATTATTCTGTAGGCGCTTGGGCACCGTGTCATGAGCGTGTCACCTCGCTATGATCAATATAAGGAAGCATGGGATACCGGTGTTCTTCTTGAGGTACAAACATCTTACTGGTCATGTCTGTCTGTACTCTATTTAAATAGTTGCGAACTTATTTGGTTTTTTTGTAGGTCACTCCTTTCTTATGGTGATCGCTCATAGAACTTTAGTCTATTTGACGTTGTAAACTTGAAAATTTCTGTGTAGATCAAATTTGCAGACACTGTGTACACTGTCCGTTTCTTCCACTGCTATAAGAACGGTGTTGATCGTGTGTTTGTCGACCATCCTGTATTCCTGGAAAAGGTATGGGGGAAGACTGGTGGAAAATTGTATGGCCCTGTCGCTGGGGAAGATTACACAGACAACCAGATAAGATTCAGTCTGCTATGTCTTGCTGCTTTGGAAGCACCTCGCATTCTTAATCTGAATAACAGCAAATCCTTTTCTGGGCCTTATGGAGAAGATGTTGTATTTGTTTGTAATGATTGGCACACTGGAATTCTCCCATGCTACTTGAAGAGCATGTACAAATCGCATGGGCTCTACGAGAATGCAAGAGTAGCCTTTTGCATTCACAATATTGCATATCAAGGCAGATTTGCCTTCTCAGACTTCTCGTCTCTTAATCTCCCAGACACATTTAAGAGCTCGTTTGATTTTATTGATGGATATCTCAAGCCAGTGAAAGGGAGGAAAATTAACTGGATGAAAGCTGGAATCCTCGAATCTGACAAAGTCTTCACAGTGAGTCCATACTACGCTGAAGAGGTCGTCGCAGGTGTTTCTAAAGGTGTCGAGCTGGATAATATACTCCGTAAGACTGGTGTTACTGGAATTGTGAATGGAATGGATGTTGATATATGGAATCCAGTGACAGACAAATATATCTGTGCAAATTATGATTCTACAACTGTCTTTGATGCAAAGCGCCTTAACAAGGAGGATCTCCAAGCGGCAGTCGGGTTACCAGTGGATCCAAGCATCCCTCTCATAGCATTTGTTGGTAGACTTGAGGAACAGAAAGGTTCAGACATCTTAGTTGCCGCCATTCCGAAGTTCATTCATGAAAACATTCAGATAGTGGTCCTCGGAACTGGAAAGAAAAACATGGAGGAGGAAGTTACGAAGCTCGAGGAAGATTATCCAGACAAGGCAAGAGGAATAGCGAAATTTGATGCTGTCTTGGCTCACAAGCTTTTTGCTGGAGCTGACCTTATGATAATTACCAGTAGGTTTGAGCCATGTGGGCTTATTCAGCTGCAGGCAATGCAATATGGGACGCCATCTATCTGTACCAGCACCGGTGGGCTAGTAAACACTGTCGTTGACGGCGTCACGGGTTTTCATATGGGAGCCTTTAATATCGATTGTGACGCTGTAGACCCGAAGGATGTAGAGGCAGTTGCCACATCTGTGCATAGAGCAATTGGGGTCTTTGGGACAGATCAATTCTCTCAGATGATCCAGAACTGCATGGCTCAAGATCATTCCTGGGAGGAGCCTTCCAAGAAGTGGGAAACGGAGCTACGAGGCCTCCTTTGTGCCCCAAGCCAAGTTGAGGCGCCCGAGATTGAGATAGAGGAATCTCTTGTTGCTGAGAAAGTCGCTGTCTGAAGGG

>TRINITY_DN18482_c0_g1_i4_2
GGCCGACGGCGAGCGGGAGTTCATGTTCTACCGCAACCCCAGCGCCGACATGCTCCTCACCGAGGCCGAGCTCAACCTCGACCTTATCCGAAGGGCCAGGATCTTCCACTACGGGTCGATAAGCTTGATCACGGAGCCCTGCCGGTCGGCGCACCTGAGGGCCATCGAGGTGGCCAAGGAAGCCGGCGCCCTGCTCTCGTATGATCCCAACCTCCGGCTGCCGCTGTGGCCGTCGGCGGAGGAGGCGAAGACGCAGATCATGAGCATATGGAACAAGGCGGACATCATCAAGGTCAGCGACGTCGAGTTCGAGTTCCTGACGGGCAAGGAGACCGTCGAGGACGACGTCGCCATGTCGCTGTTTCACCCGGAGCTCAGGCTCCTCCTCGTCACCCTCGGCGAGAAGGGCTGCAAGTACTACACCAAGGAGTTTAAAGGGTCCGTGGTGGGCTTCTCGGTGAAGACGGTGGACACCACCGGCGCCGGCGACGCGTTCATCGGTGCGGCGCTGCGCAAGCTCGCCGTCGACCTGTCGGCGGTGCAGGTACGCCATTTCTGTCTGGCATCATGGCCCCCACTTTCCTCCGACGGTGGTTGGTTGATCTCTACGCCTAAAACTGCGCCGTGAAAGCCGTTCGATTAAGGCACCGCGGACCGCAGAGCCGTCTGATGGCTGCGTTCTCGCAATCGACCCTGTGTGGACCGCATCTTTTTTTTTTTACAATAATAGAAACCCTATTATTCCTTGATAAAACCATGTGCGGATTTGAACGTAACATCTCTATACACACATACATATTCGTATCACATATATATTTAAGTATGTCTTTTTTTGACAATTATTTAAGTATGTCTACCTACCTATATATATACACACATACCTCTTTTAAAAAAATATTTACATAAATATGCCTGGATAAAAAATATTTAACAAACTATGCTCCTGTGAAAAATACTTACAAAACTATGCATCTATCACTTACACTATTTGAATCTAGTTTACACTTTTTTATTTTCTTTTACACTATTTGTCTTCATGTTATATTATTTGTCTTTAGATTATGCTATTTGATCTGAGTAAATAGTATAATATGAAGATAAATAGTGTAATCTAGAGACAAATAGTGTAAGTTTGGGTTAAATAGTGTAACTATCGGAGCATAATTTTGTAAATATTTTCAACTTAGGTATATTTATGAAAATAATTTTTAAAAGAGGCATATTTTAATAAAAATTCCTATAAATAAATATCCCCTTGGCTTTTGACAAGCATCAAACCCTAATAGACATGAATTGTCATAAACGCCCTTTGTTTGATGCAATACCCAGCTGAGGAATTGACGAATAATATATTTATATTTGTGTATGGATGTGAATTTGCAGGACGAGAGAAAGCTGAGGGAACTGCTGGAATTTGCAAATGCATGTGGGGCAATCACCACCGCCAAGAAAGGAGCAATCCCTTCCTTGCCAACTGAGTTGGAAGCGTTGGAGCTGATCAAACACAA

>TRINITY_DN19169_c0_g1_i6_3
CGCAAGCTCGCCGTCGACCTGTCGGCGGTGCAGGACGAGAGAAAGCTGAGGGAACTGCTGGAATTTGCAAATGCATGTGGGGCAATCACCACCGCCAAGAAAGGAGCAATCCCTTCCTTGCCAACTGAGTTGGAAGCGTTGGAGCTGATCAAACACAAGTGAAGGAATGGTTATTAAGCAGTTGCAGCCCTTCTCTTTTTTCTGTTTGGTTTTTAACTTTCAAATTGGATGAGCATTTGAAATATGATAGCCAATCCAGCTAGGGTTGCAATCCGAACGGGTTGCCTTCTTTTTTCCTTTTTTTTTTTTCTTCTCCCCACCACTCCCCCAACTTCAAGTTTTCGCTGGAAATCCCTTTTTAAACGTTTCATAAAAAAATCAGATGTAGTGGGCAATAAGAACCTTGTTCGAGTGCCAAATCGAGTTTTAGAGTCTTTAAGTTTGTGGTATTCATGTAGTATACAAGGAGAGTGTAGTATTGGTGGAACATTATTATTTTTTTGGAGGAGCAGAGTAATCATATGAATATTTTATTTTTGTTTCTTTTTGTCGGGACTAGGTAAAGAAATTTTATTCG

>TRINITY_DN21454_c0_g3_i1_1
AGCAGCCATGGCCGACCTCGTCGTCAGCTTCGGCGAGATGCTCATCGACTTCGTGCCCACCGTCTCCGGCGTCTCCCTGGCGGAGGCGCCCGGGTTCCTCAAGGCCCCCGGCGGCGCCCCCGCCAACGTCGCCATCGCCGTCGCCCGCCTCGGCGGCCGCTCCGCCTTCGTCGGCAAGCTCGGCGACGACGAGTTCGGCCACATGCTGGCCAACATCCTCCGCGACAACGGCGTCGTCGACGCCGGGATCTGCTTCGACACGGGCGCCCGCACCGCCCTCGCCTTCGTCACCCTCAGGGCCGACGGCGAGCGGGAGTTCATGTTCTACCGCAACCCCAGCGCCGACATGCTCCTCACCGAGGCCGAGCTCAACCTCGACCTTATCCGAAGGGCCAGGATCTTCCACTACGGGTCGATAAGCTTGATCACGGAGCCCTGCCGGTCGGCGCACCTGAGGGCCATCGAGGTGGCCAAGGAAGCCGGCGCCCTGCTCTCGTATGACCCCAACCTCCGGCTGCCGCTGTGGCCGTCGGCGGAGGAGGCGAAGACGCAGATCATGAGCATATGGAACAAGGCGGACATCATCAAGGTCAGCGACGTCGAGTTCGAGTTCCTGACGGGCAAGGAGACCGTCGAGGACGACGTCGCCATGTCGCTGTTTCACCCGGAGCTCAGGCTCCTCCTCGTCACCCTCGGCGAGAAGGGCTGCAAGTACTACACCAAGGAGTTTAAAGGGTCCGTGGTGGGCTTCTCGGTGAAGACGGTGGACACCACCGGCGCCGGCGACGCGTTCATCGGTGCGGCGCTGCGCAAGCTCGCC

>TRINITY_DN23579_c0_g3_i1_3
AGACCCTTTCGTCTATAAAAAAAAGGCAGGACCTTTCTTCCCATTCCTCCTCCTCCTACCTCTTCCTCCTCTCCTTGCAGGCCCTGCTCTCGCCTCCCGCAGCAGCAGCAGCAGCAGCCATGGCCGACCTCGTCGTCAGCTTCGGCGAGATGCTCATCGACTTCGTGCCCACCGTCTCCGGCGTCTCCCTGGCGGAGGCGCCCGGGTTCCTCAAGGCCCCCGGCGGCGCCCCCGCCAACGTCGCCATCGCCGTCGCCCGCCTCGGCGGCCGCTCCGCCTTCGTCGGCAAGCTCGGCGACGACGAGTTCGGCCACATGCTGGCCAACATCCTCCGCGACAACGGCGTCGTCGACGCCGGGATCTGCTTCGACACGGGCGCCCGCACCGCCCTCGCCTTCGTCACCCTCAGGGCCGACGGCGAGCGGGAGTTCATGTTCTACCGCAACCCCAGCGCCGACATGCTCCTCACCGAGGCCGAGCTCAACCTCGACCTTATCCGAAGGGTGCGTCTGCTAACCTGGATCCCCGATCCGATCCAAATCCTCCTTTTTGCCTCCTGATCAGTAACGTAAAGGGAACCAGAATATTCTTGCTCTTGTCATCTTCCAACAATTTTGCCCTTTTCATTTATTAATCCTCCTGATGCACCGGAAAAAGCGCATTTGGATCTAGAACAGGAAGAACATGTTATTGGCCAGATTTGATTGTTTATTTTCCAAAAGCTTTTAATCGAATCTGCCCCTCCCTGTTCCATCTCTCTCCCTCGCCGACTACCTTCTTCCGCTTTGTTTGACTGAACGGATGGAAGAGCCAAGGAGACGTCTGCAACTCCAAGCAGTAAATGCCATGCTCCGTCAAAGTTAGTGTGCGGCATCCACGAGAAATTTGGTTTTCCCACCGTTCTTAATGAGGAATCCGAGCTCTGCGGTCGCTTCAATTCCCCATTGGCCGTCCATTTGTTTTGGCCGCTTCTCTCGAGTAGATCCTATTATTTTTTTCCTCCCACCTGGTCCCGCATCAAGCCGCAAGAGCCAAAAGAAGCCCTAATCTGTGGGGAGTTCGTCCAGAATATTGATATTTTTTGTTCATTAATACCATCGCCCATCGTCTTGTTGAATCCTTGAACGATCTGAAATCATGACAAATTTCTGAAACCAATCTAAAAAGTTTCCTCATTTTTTTTCTCACTGGAATCTGTGTTGATG

>TRINITY_DN24556_c2_g1_i4_5
CTCAGGGCCGACGGCGAGCGGGAGTTCATGTTCTACCGCAACCCCAGCGCCGACATGCTCCTCACCGAGGCCGAGCTCAACCTCGACCTTATCCGAAGGGCCAGGATCTTCCACTACGGGTCGATAAGCTTGATCACGGAGCCCTGCCGGTCGGCGCACCTGAGGGCCATCGAGGTGGCCAAGGAAGCCGGCGCCCTGCTCTCGTATGATCCCAACCTCCGGCTGCCGCTGTGGCCGTCGGCGGAGGAGGCGAAGACGCAGATCATGAGCATATGGAACAAGGCGGACATCATCAAGGTCAGCGACGTCGAGTTCGAGTTCCTGACGGGCAAGGAGACCGTCGAGGACGACGTCGCCATGTCGCTGTTTCACCCGGAGCTCAGGCTCCTCCTCGTCACCCTCGGCGAGAAGGGCTGCAAGTACTACACCAAGGAGTTTAAAGGGTCCGTGGTGGGCTTCTCGGTGAAGACGGTGGACACCACCGGCGCCGGCGACGCGTTCATCGGTGCGGCGCTGCGCAAGCTCGCCGTCGACCTGTCGGCGGTGCAGGACGAGAGAAAGCTGAGGGAACTGCTGGAATTTGCAAATGCATGTGGGGCAATCACCACCGCCAAGAAAGGAGCAATCCCTTCCTTGCCAACTGAGTTGGAAGCGTTGGAGCTGATCAAACACAAATGAAGGAATTGTTATTAAGCAGTTGCAGCCTTTCTTTTTTTTTTTCTTTATAACTTTCAAATTGGATGAGCATTTGAAATGCTATAGCCGATCCAGCTAGGGTTGCAATCCGAATGGGTTGCCTTTTTTCTGGTTTTCTTTTTTTTTTTCTCTCCACCACTCCCCCAACTTCAAGTTTTCGCTGGAAATCCCTTTTTAAACGTCTCATAAAAAGATCAGTTGTAGTGGGCAATAAGAACCGTGTTCGAGTGCCAAATCGAGTTTTAGAGTCTTAAGTTTGTGTCATTCTTGTATTTTTAAGTTTATATGGTACAGAGTGTTGGACAATACGAAAGGAACACATAAATAAAATGTGTGTAG

>TRINITY_DN20976_c0_g1_i1_3
CTAAAAACCTTTTGTCAAGTAATACTTCCAAGTCACTATCAAAGTTTTAACAGCTTTGTATGTTTTCCTTGGAAGAACTTTTTATAGCATGCTTTAACTACAGTGGAATTGACTGACTGTTAAATCATGTTCGCTTTTTGATAATTTTGTCAGAAATGAGGCTATACTTCGCCTTCCCGCATCGTTAATGGAGTTTGGCCTTTGGTCACCTTCCAAAAGATTCCTGCATTGTTTACTTATTGATAATTTCATCATTGTCATTCTATTTTTTACTCCTGCATTGTATCATCGTAGCAATTGGTTTTCTTAACTGTCGTGAACTTCTGTGGTGGAGCATTAGATTTGTTAGAATTGGATTAATATAATCTAAAATTAAGAACTGCTGCAATGTTGTGGTGGTGTCACAACTGGGTCCATTTTCAAGTTAATCATGTTGAACTGCATCAAAGTTTAGGAAAAAAATGAAAAACAATGTGGCATTTATTAATATTTTTATCATTGCCTCGCCAAAGAAGTTTATGAATTATATAACATTGCCAGTACAATAAAAACGTAGCTTGAAGTTCACTGGATTTGGCGGACATGACAAATTTCTTAATACAAGTAAGTCTTGAGTGAATGCACCTTAAAGCTATTAGAAAATGAAATCTGATCAAAAGTGACATTGAGTATATTGCAAAAAGTTCATATAAGAGAAGAGTTGCCCTTTAGGTTACCTTTTTTATCACAAGCCATTCACAAACTGTCTTCTGTTTATCAGCTTACTCTAGGTTGTCTAGGCATTTATGTGCTAAACTGTCTGAAGCTAAACTTAATAAGCAAAGCTTGTGCTAATTATCATTTGTCTATTATGTTCTATTCATCTCCCCTAAATCTTGATAAACTATTTTCTTGTTCCTGTGGCTTGCAGCTCCTGGAAATTGCACAGGTTCCCAGTGAACATGTCAACGAATTCAAGTCAATCGAAAAATTCAAGATATTCAACACCAACAACTTGTGGGTGAATCTGAAGGCCATTAAGCGTCTTGTTGAGGCTGATGCACTTAAAATGGAGATCATTCCCAACCCCAAGG

>TRINITY_DN26607_c0_g1_i2_5
ATTTCCTCGCACCAGAGGAATGCGACGATACACCTCCTCCCTATAAATAGCGCGCGGATCGCAGCGCCCTCCGCTTCCACCAACCACACCGCCGTGATCGTCTCATTCGGGTCCTCTCGCGTCCCTTTCTCGCTCTTCGCCGGCGGCAGTCGAAGACTCGAAGAAGGTCCTGATCTCCTCCGATGGCTGCCGTGGCCGAGGCTCCTCTCGTCACTGAGACCGAGAAGCTCGCGAAGCTGCAGGCTGCAGTCGCAGGCCTCGACCAGATCAGCGAGAACGAGAAATCCGGGTTCATCAGCCTCGTGTCGCGGTATCTGAGTGGAGAAGCACAGGAGATTGAATGGAGTAAGATCCAGACTCCCACTGAGGAAGTGGTCGTGCCTTACGACAGGTTGGCTCCTCCCCCAGAAGATCTCGCATCCACTAAGAAGCTCCTCGACAAGCTTGTTGTATTAAAGCTCAATGGAGGTCTTGGGACAACGATGGGTTGCACTGGTCCAAAGTCTGTTATCGAAGTTCGAAATGGGTTGACATTTCTTGACTTGATTGTCATCCAAATTGAGTCTTTGAACACCAAGTATGGATGTAATGTTCCCCTCCTTTTGATGAACTCATTTAACACACATGATGATACGCTTAAGATTGTTGAGAAATATTCCAACTCAAAGATTGACATACACACTTTTAATCAAAGCCAATACCCTCGTTTGGTTGTTGAAGATTTCCTGCCACTACCAAGCAAGGGGCGGACTGACAAAGATGGCTGGTATCCTCCAGGCCATGGCGATGTGTTCCCTTCCTTGTTCAATAGCGGAAAACTTGATGCCTTGTTATCGCAGGGTAAAGAATATGTTTTCGTTGCCAACTCTGATAACTTGGGCGCCATTGTTGATTTGAAAATACTTAATCATTTAATCCACAATCAAAATGAGTACTGCATGGAGGTAACCCCCAAAACCTTGGCAGATGTTAAGGGTGGTACCCTAATTTCATACGAGGGAAGGGTTCAGCTCCTGGAAATTGCACAGGTTCCCAGTGAACATGTCAACGAATTCAAGTCAATCGAAAAATTCAAGATATTCAACACCAACAACTTGTGGGTGAATCTGAAGGCCATTAAGCGTCTTGTTGAGGCTGATGCACTTAAAATGGAGATCATTCCCAACCCCAAGGAAGTTGATGGTGTTAAAGTTCTTCAGCTCGAAACAGCAGCTGGTGCAGCAATTCGTTTCTTTGATAATGCTATTGGAGTCAATGTTCCTCGATCTCGTTTCCTTCCTGTGAAGGCAACATCAGATTTACTTCTTGTCCAGTCGGATCTGTACACATTAGCTGATGGCTTTGTTGAAAGGAATGAAGCTAGGACTAATCCAGAAAATCCTTCTATTGAGTTGGGGCCTGAATTTAAGAAGGTTGGGGACTTCCTCAAACGTTTCAAGTCCATCCCCAGTATCATTGAGCTTGACAGTCTAAAGGTTTCTGGTGATGTTTGGTTCGGTGCTGGTGTAGTTCTTAAGGGCAAGGTGGTCATCAGTGCAAAAGCGGGAGTGAAGTTGGAAATACCAGACGGAGCTGTCGTCGAGAACAAGGCGATCAACGATGCTGATGACATCTGAGATTCTGAATGTTTTGCGCACATTTCTTCCTATTCGATAAGTTCTCTTCTTCCCGGGTGCAGTAGCTTTGCCATGTTGCAATGGATCATTGCGTGAGATGCCTTTTTTTGTTCTAGTCTGAATACTGCTGCCATGTTATAAAAATAAAACAATGAGCCTATGAGAATGCCCGAGAGAGTCGTTTCCTCTCTTTTCGAGGATGGAATGGTGTGGGTGCCTGGCACGAGACTTGATCTGCATGTGGTCGGGAGAATGTTCATCGCTTCGTACTCTTTAGCATCCCATTTGTCACGTTTTGATCTTTAGATTCGATGGATGAGATGAGGTGGGCGTTGGACTTAAAGTTTGTATTCAAAATGTGTAGTAAATGAGACATTGTTGCTGGTGGATGGTGTGATACATATGGAGGGAGTGTGAGGCTCTCAAATAGTTAAGAGTAACTAGACTAGAGGTTTTACTCGCACATGAGATTCTACAGAGAGTTGGGCCGGAGTGGAAGACCACCCCACCTCCATTGCCCCATTGATGCTATCTGTCTCTATACACATACACATACACTAGCACCAGAGAGGAAGACCCCACACACACGTGCCACCCCCATTGCTCTCTCTCTCTCTCTCTCTCTGAGAGCCCCATGGGCCCCTCTCTTTCTATTTGTTCTCCCTTTGGTTGGGGCCAGCACCACGTGGCTATCTACAAATAACTCATTGTTGCCGTGGTAATCCAAATATTTGTGGTAATTTTTT

>TRINITY_DN28020_c0_g1_i5_1
ACTATTTTCTTGTTCCTGTGGCTTGCAGCTCCTGGAAATTGCACAGGTTCCCAGTGAACATGTAAGTGATGCCGATCTATTTTATTTTCCTGTTAATGTCTGTTATCTGAGAACAGAATGGGGGCATTGATCAGTTAAAGTTTTGGTACAGAGTCCTCTTAACTGATATATAACAATGTATTCAAATCTTCCCCAGGTCAACGAATTCAAGTCAATCGAAAAATTCAAGATATTCAACACCAACAACTTGTATGTAAACTTGCTTTATAGATCCAATTTTATCATGCTTGAAGTGTTGCTAATCACATTTCTGCAGGTGGGTGAATCTGAAGGCCATTAAGCGTCTTGTTGAGGCTGATGCACTTAAAATGGAGATCATTCCCAACCCCAAGGAAGTTGATGGTGTTAAAGTTCTTCAGCTCGAAACAGCAGCTGGTGCAGCAATTCGTTTCTTTGATAATGCTATTGGAGTCAATGTTCCTCGATCTCGTTTCCTTCCTGTGAAGGCAACATCAGATTTACTTCTTGTCCAGTCGGATCTGTACACATTAGCTGATGGCTTTGTTGAAAGGAATGAAGCTAGGACTAATCCAGAAAATCCTTCTATTGAGTTGGGGCCTGAATTTAAGAAGGTTGGGGACTTCCTCAAACGTTTCAAGTCCATCCCCAGTATCATTGAGCTTGACAGTCTAAAGGTTTCTGGTGATGTTTGGTTCGGTGCTGGTGTAGTTCTTAAGGGCAAGGTGGTCATCAGTGCAAAAGCGGGAGTGAAGTTGGAAATACCAGACGGAGCTGTCGTCGAGAACAAGGTGAGAACGGCTTTGTCATGTTCCCATTCATTGTCGAGCCTCTTTTTGCTCAAAAATCCATTTTCCCTGGTAAACCCTTTTTTTCTTATCCTATTTTTTTTCCCACAGGCGATCAACGATGCTGATGACATCTGAGATTCTGAATGTTTTGCGCACATTTCTTCCTATTCGACTATGTTCTCTTCTTCCCGGGTGTAGTAGCTTTGCCATGTTGCAATGGATCATTGCATGAGATGCCTTTTTTTGTTCTAGTCTGAATACTGCTACCATGTTATAAAAATAAAACAATGAGCCTATGAGAATGCCCGAGAGAGTCGTTTC

>TRINITY_DN23835_c1_g2_i3_5
AAATAACCTTACAAATCTTTGTCTGCAAGTACATCTCCTAAACCCAGAAATGACCCCAGAACCCTTGGACATCCTCAAGGGTAGCTCTTACCAGGATCAGATCTTCTCGATCTGGTTCGGTAAAAGACCTTCATAGACCCTCCTAATCCTCCATCTGACTTGTCGCGAAACTCAATCCGAACCGGATCAGATATAACCCACTATCAGGATTTACAAAGGTCCAAGCCCAAACTTGTGCAAGGCCTAGTTCGTTTGTTATTATGCAGATCGTGTCATCTCCTTCGTTCTAGTTGTTTATATGACGACATACTAATGCCTATTTTCCAGCTATATTTCTGCTAGTAGACTGCATTCTTTCATCGCTTCACTGTTTCTTACAATCCATTGTAGCAATACTTTTCGGCCTTGGTATATCTGTCAGCTGTTGATCTAAACAAAATTAGTGTTGGATCTTTCAAGTGGTTGATGTTTTTGTTTCTTTGCTCACCGTACAGAACTGCAAGATTCACCATTCTGTAGTTGGTTTACGCTCCTATATTTCCGAAGGTGCAGTTATAGAGGACACTTTGCTCATGGGAGCTGATTACTATGAGGTAATGGCCTTCAAAGCTAAAAGAAAGCTCATTGTCACACCAGCCGTGATGCCCGGCAGCCCTAGTCATCTCTTTTTTTAAAAAAAAAATGATTTGTTGATATTGGAATATGATGAGCATGGGGTTTTAAATGTGACCAAATCTGATTCCACTCGCGGACTGTGTATAAGATTTTCACCACAGCCAAAATGCATGACTATTCCATTTACAGCAAAAATGGTGCTGTGTAAATGGGGGCGTTCTTGTGTTTCCCCACTAAATGTTTCGTCTCCTTTATGATCCTGCCAGACAGATGCCGACAAAAGATTCCTGGCTGCGAAGGGGAGCGTTCCAATTGGCATTGGGAAG

>TRINITY_DN24188_c0_g2_i1_1
CATTTAAAATCTGAATGCATTTAAAATCAGAAAACACATACCTTTTTTGACACAAGCTCCTTCGTCGTCCTGTCCCCCGAAAGGAACAGACATTAAATCCAATAAGCATATGTCACATAGCAAATCATGGCATCCGATCATGGAACCATTTTCATGAGTCATACACAAACGGCAGTCCGCCAGATCCCCCAAAAGTCATGATCCGAGGAGTATGATGCCATGCTGCTGCTCATCTAAACAGGAAAATCGAAAAAACGATCGATCGATGTAAGCCGGAACTAGACAATCTCCACAGAGGCCAAGGAAACCACAGCCCTGGACAAAAGAACAGAGACCGATTAGGCTCACCTTGCTCGCCTCGGGATCGAGGCAAGTCTGTGAGCTCTTGGAATCAGAGACAGCCTTCGGCGACACCAGAATCGGCACCCGGCCCCCAGACGCGGGTCTCG

>TRINITY_DN24647_c0_g1_i1_5
CCGCCACCTTCGCCACCTCTCCCTCCTGCTCTTACGCACACTCACATTAGCTCTGACCCTCTCCTCTCTCTCTCTCTCTCTAACTCAGTCGGTGTGTCACTCTTTTTCTCTCACTACGCAATCAAGGAGGAGGAAGGGAGAGGAACCCAGCAAAGAATTCCCCAAATTTTCTCCTCCGCATCTGGGAGCAAAGATTTTTGTAGGATCACTGCAGAGTGCAGCACGAAGGAGAAGTAAGGATAGAAGAGTGGGAAGACGATAACCTTTGAAGCAAGGAGGAGGAGGAAAGAGGAGGAGAGTACTGTTTCGACATCCTGTGACCATGGAAGCCGGCAGTTCGGTGATGCAGCTCAGGGCCAACGTCCCATGCCTGAGCCGGGCGCGCCACGGAGGTGGTTGCCTTTTGAGGGAGGAAAGAGCGATTTGGGGTGGGGATGTGCTAGGTGGCAGCTCAGTGACCTCGGGGATTTGGGGCTTAAAGATGCCTATGCGCCGCTCTCTGACCGCCGCCGGCGCTCCGGCCGCCGCCGTCCGGAGCGCACCCCGTGCCGGTGTCACTGTCTCCGTCCTCACTTCAGATGTCAGCCAGGACACCCTGACATTACCGGCACCATTCTTTGAGATGGAGCGGGTGAATCCGAAGAACGTCGTCGCGATCATCCTTGGAGGAGGCGCTGGGACTCGGCTCTTTCCGCTTACCAGCAGAAGGGCTAAACCTGCTGTTCCAATTGGAGGATGCTATCGGCTTATTGACGTTCCGATGAGCAACTGCATCAACAGTGGCATAAACAAGATCTATGTCATGACCCAGTTCAATTCTGCATCTCTTAATCGCCACCTTGCTCGCACATACAACTTCGGAAATGGTGTTAGCTTTGGTGATGGATTTGTTGAGGTTCTGGCAGCCACTCAAACCCCAGGTGACAAGGGCATGAACTGGTTTCAGGGAACAGCAGATGCTGTGAGGCAATTTTTGTGGATTTTTGAGGATGCAAGCAAGAAGAACATAGAACATGTACTAATCCTGTCTGGTGACCATCTCTATAGGATGGACTATACAGACTTCATACAGAAGCACATGGATACTCTGGCGGATATCACAGTTTCATGCATACCCATGGATGAAAGCCGTGCATCTGACTTTGGGTTGATGAAGATCGATAAAATGGGTCGTATCTTACATTTCTCTGAGAAACCAAAGGGTGACGCTCTAGAGACCATGAAAGTTGACACCACCATTCTTGGGTTGTCCCCTCATGAAGCCAAAAAAAATCCATACATTGCATCCATGGGAGTGTATGCTTTTAAAACAGAAATTCTTCTAAATCTCTTGAGTGGTAGGTATCAATCGTCCAATGATTTTGGATCAGAAGTCATCCCATCTGCAGTGGAGGAGTATAATGTCCAGGCTTACCTGCACAATGATTACTGGGAAGACATTGGAACAATTAAGTCATTTTTTAATGCAAACATGGCACTGACGGATCAGCTTCCTAAGTTTCAGTTCTATGATCCCCATACACCTTTCTACACTTCGCCTCGTCACTTGCCGCCTACGAAAGTAGACAAATGCAGGATCGTCGATTCTATTGTCTCGCACGGTTGCTTCTTGGATCATTGCAGTGTAGAGCATTCTATTATTGGTGATAGATCTCGCTTGGAGTATGGTGTCGAATTGAAGGATACCATGATGATGGGGGCTGATCATTATCAAACTGAAGCTGAAAGAGCTTCTCTACTAGCAGAGGGGAAGGTCCCTATTGGAGTGGGACAATTCACCAAGATCAGGAACTGCATCATTGACAAGAATACGAAGATTGGAAAGAATGTTATTATTGAGAATAGAGATGGTGTTCAAGAGGCTGATCGGCCAAGCGAAGGATTATATATTAGGTCTGGCATCACCGTTATACTGAAGGGCTCAACAATAAAAGATGGGACTGTCATATAAGATGCTCAGGCTACAGGATGAGATCTCCCTATGTAGAATGCAGAGGAACCGATGTAAGCTGCCATATATCCATCTGCTGTGTGGGGTTTGGCCAAACAGTCCACAAACAGATGCACATGCATGCCAGTAATCTAGGAAACTCTGCATCCACAGTCCACTACAGCTAAATATCTATGAAATAATATGAATGCAGCTGCAGTCTCCCATGTACATTAGAAATTCCTGTTTCATGTTTGAGGACTAGCAAGTGTATACTATTTTTCCCAGATAACTTGATGGTAGTGTCCATGTTTTGATTTTGTAGTACTTTGTTCACAAAAACAATTGAAGTAAGGTGGGTCTTTGAATATA

>TRINITY_DN16038_c0_g1_i3_5
CTCTCTCTCTCTCTCTCTCTCTCTCTCTCTGGCCTGGCTTTGTCCTCCTTCTCCGCCTCTTGCTTTTGTTATTTTACTAGCGTCTTTGCCTGAGGAGGAGGCGGATTTCTGACCTTGTTGCAGCTCAATTCCTCCTCCGTGCCTCTTCTGCGAGGAAGGTTCCATCTTTACCTTTCTTCCTTCTTGATTGATTCGTCTTTATTTATCTCTCTCTCTGTCTCTGTGTGTTTTTTTTCCCTTTCCTCCTTCTTTAACGCGCTGGAAGAGGTACACTGAGGTTTCAAGAAGGAGCGCCCCCGCCCAGATGCCTGCGGATTCTGCGTTCGATTTGGGTTTCTGGTTGTACAGATTGCTGGCTTTTCTCGGGGGCTAACACCGACCGTGTAAACGGCTTTTGATTGGAGGTGCATCTGACCTTGGCGCATAAACGGGGTTTACCTGAAATCATGGATAGACTCAATCATCCTCCCCGTCTCATGATTGTATCCGACCTTGACCATACGATGGTTGACCACCACGATCCGGAGAATCTCTCGCTGCTTAGGTTTAATGCTCTGTGGGAATCTAACTACCGTCATGATTCTCTGCTTGTTTTTTCAACTGGGAGGTCACCTACGCTTTACCAGCAATTGAGAGAAGAGAAACCGATGTTAACTCCTGATATAACCATCATGTCTGTGGGTACTGAGATCACCTATGGTGAAGCGATGGTGCCGGACAAAGGTTGGGAGGAGGTTTTGAATCATAAGTGGGACAGAAATATCGTTGCCGAAGAAACTGCCAAGATTCCCCAACTTTCCCTTCAGTCAGAAACCGAGCAGCGACCACACAAGGTCAGCTTTTATGTCCAGAAGGAGCATGCTGAAGATGTCATGAAATCTTTATCAGCATGTCTAGAGAAACGTGGGTTAGATGTGAAAATAATTTACAGTGGTGGTCAAGATCTTGACATATTACCACAAGGTGCTGGCAAAGGACAGGCACTTGCATATCTCCACAAAAAGTTTCAGTCTGCTGGTAAATTGCCCTTGGCTACTCTAGTGTGTGGCGACTCTGGCAATGATGCTGAATTATTCACCATTCCTGACGTATATGGAGTCATGGTCAGCAATGCCCAAGAGGAACTACTGCGGTGGCATTTAGAAAATGCCAAAAACAATCCCAAAATAATCCATGCCACAGAAAGATGTGCTGCCGGTATTATTCAAGCCATTGGTCATTTTAAGCTTGGTCCTAGTACTCCTCCAAGGGATGTTGTGGACCTTTCAAGTTGCAAACTTGAGAACTTCAGTGCTGGTCTTGAGGTGGTAAAGTTTTACATTTTGTATGAAAGATGGCTTCGAGCAGAAATTGCTAGCTCTGAGCTTATCCAGAACTTGAAAGATATCTGTGTGAGTCTGAAAGTGGACTTTTATATGCTTTCACTATTTATACATGAATTCTTTCAGAATTAGAAAGATAAGTAGGAGAGGGGAATTCATGGCTAATCATAAGAGTCTTGTGCTAATAACTTCTTCCACTTTTTTTTTGCATATAATAGGCTTGGCTGTGAACAGCCAAAAGAATGAGAACTTAGGGCTTAACTCTAGATGATTATCGGTTATCAACTGTGACTTCCCTAGTGCTTGCTATGATCTAATTTGATCTACTTTGTTGAACAGCATCCAACTGGTGTTATAATTCATCCTTCCGGTGTTCAGAAATCCCTTCATGAGAGCATTGATGCCTTGAGGCCACTTTATGGTGACAAACAAGGGAAAATATTTCGTGTATGGGTGGATAAGGTTTCTTCTGCTCAAATTTGTTCAGATGCCTGGCTAGTAAAGTTTAATAAATGGGAGTTGTCTGGCGAGGAGCGGCACTGTTGTGTGACCACTGTGCTGCTGAGTTCAAAGCCTGAGACTCTGTACGGATTCTGCCTGGTGCATGTGCATCAGACATGGCTGGATGGACATGCGGGAGGTGATAAAGCCACCTGTGTTCTTTAGGTCGTTTCCTGCTCTACCCCTGGTTCTCCTATTATCATAACTCGATTCGGATGGAATAATAATAAGCAGTTTCCTGGTGGTTCTGTTCTGTATTCTCAAGTAATGTCAGCCCGTTAGGCTGGGGACCTGATCTATTCAGGCAAAGAAATGGCATACACAGCTTCTTCGTTCTCGTCATTGACCCTTTCTTCTGAGCTTCAGACGTAATTCCATGTCTTTCTATCTGTTTATACTGTGTTTCTCTGTGTTTTACATTATTCCATAGACCTTTCTTTATGTTTATGCTTATATGTGTTTGTTCGTTTTGCTTGT

>TRINITY_DN17016_c0_g1_i3_5
GAGAGAGAGAGAGAGCTTGTGGCTCTCCCTCTCCCAAGTCCCAATGGGTGCTCTGCTCCTCCGAACTCCGACGTCGACCCTGCGACGACGGGGCTCCCTGCTGCTTTGCCTCTTGCTGTTGCTGCTCCTGCAGGAGAGCCGGCCGCCGGCGGAGGCCGCCCACACGCTGTACAGCGAGTACCAGGCCGACGAGGCGGAGAGGGCCGTGGGGGCCAGCCAGCACAGGACAGGCTACCACTTCCAGCCCCCTAACAACTGGATCAACGACCCCAATGGACCCATGTACTACAATGGACTCTACCACCTGTTCTACCAGTACAACCCCAAGGGCGCCGTCTGGGGCAACATCGTCTGGGCGCACTCCGTCTCCTCCGACCTCATCAACTGGACCCCCCTCGACCCCGCCATCTACCCCTCCAAGCCCTTCGACATCAACGGCTGCTGGTCCGGCTCCGCCACCATCCTCCCCGGCGACCGCCCCGCCA

>TRINITY_DN25871_c0_g1_i3_1
GCCGACGGGTGGTGGCGGGTGACGGTGGGCAGCAAGATCGACCGCACCGGCAAGGCGATCCTCTACCGCAGCAAGGACTTCGTCCGCTGGGAGAAGGCGGCGCGGCCGCTGCACGCGAGGGAGGGCACGGGGATGTGGGAGTGCCCGGACTTCTTCCCGGTGGCGCTGCGCGGGAGGAAGGGCCTCGACATGACCTCGGCCGGCGGCTCCGGCAAGTACTCGAAGGCGACCAAGCACGTGCTGAAGGTGAGCCTGGACGAGACGAGGTACGAGTACTACACGGTGGGCAGCTACGACGAGGCGGCGGACGCCTACGTGCCGGAGGGGACGTCGCCGGACGACCGGACGGGGCTGAGGCTCGACTACGGCAACTTCTACGCGTCCAAGACGTTCTTCGATCCGGCGAAGGGGCGGAGGATCCTGTGGGGGTGGTCGAATGAGTCAGACAGCGTCGCCAGCGATGTCCAAAAGGGCTGGGCCGGGATTCAGACGATCCCAAGAGTAGTTTGGCTGGACGGGAGTGGGAAGCAGCTGGTGCAGTGGCCGGTGGAGGAGGTGGAGAAGCTGAGGGGGAAGAGAGTTGAAGCCCACGGCGTTGTGCTGGGGAAGGGTGACCACTTCGAAATCAAAGGGATCACAGCCTCCCAGGCGGACGTGGAGGTGACGTTCGAGGTGTCGGGGTTGGAGAAGGCGGAGGCCTTGAAGCAGGCCAACAGGTGGGTCAAGGACCCGCGGGCGCTGTGCGAGCGGGTTGGCGCCGACGCGGCGGCCGGCGGCGTGGGGCCCTTCGGGCTCTGGGTGCTGGCCTCCGGCGACCTCCGCGAGCGCACCGCCGTCTTCTTCCGCGTCTTCAGGACGCCCCACAAGAACCGCCACCTCGTCCTCATGTGCCATGACGCCATCAGGTCGACAACCCTGGCCGGAGTGTGGAAGCCGTCGTTTGCAGGGTTTGTGGACGTGGACATCCAAAAGGAGGGCAAGATATCTCTAAGAACCCTGATTGATAGGTCGGTGGTGGAGAGCTTCGGGGCCGGGGGGAAGACTTGCATCACGTCGAGGGTGTACCCCGTCGACGCCGTGGACGGCGCCGCCCACCTCTACGCCTTCAACAACGGCGCTGCACCGGTGAGGATCTCCCGGCTGCAGGCATGGCAGATGGCAACGCCAAATTACATGAACTGATCATCGTTAATAATATTTTAACAATAACGTACACGCGGCGCCGCCCCAAAAACCATGCACGTGTGGTGCATGCTGTTTGGTGTTGTAGAATAATTAGGA

>TRINITY_DN28844_c0_g1_i6_3
CCCTTCGGCCTCTGGGTCCTGGCCTCCGGCGACTTGAGGGAGCGCACCGCCGTGTTCTTCCGCGTGTACAGGACCCACCACAAGAAGCACGTCGTCCTCCTCTGCCATGATTCCAGCAGGTTTGTCCGGTAGCAACACAACAGTACTCTCCCCCCCACCGGCCGTCCCCACCTTCTTAATTTCCGCCGGCCAGGAAGATTCAGGCCGCAAACGGTGGTCATATATATATATATATATATATATATATATATATATATATATATATATATGGTCCCCCTACTCTTGTCTGATATTGGCCAGGTGGGAATCTAGCTAGTTTTTTTCCCTTACATCACATCATTGAAGGTATATATATGCACTAATTGACATGCTAATTAAGTTGTTGGTCGCCCTTGTTTTGGTTAGTAATCTTGGATCTTGTAGGATTGTAAATGGGTCGAATTTGATGTGATTTGAATCCGATGGTTTTGAATTATCGGAAATGTGATTCGAAAAGCAAATAATATATACTATATCATATATGTATAATCTATTATATAATATAATAGATTAATTGTATTATCAGATTTAGAAATTGTTAATTACTGTTTCATTATCTGCTTCTGATAGTTTATCAAAAATATCCAAATTTTAATCTGACTGAGCATCGATGGTTGAATGGCTTTTTGTATCAAATTTTATCAGAATCAGCGCAGATATATACAAATATTCAGATTTTTTTTCAGATATTGAGATAGCTAGCTAGCTAGTGAGCTGCGCATCCGATAATTAAGTCAGATTTTTTTGACTTTCCGCTGACTAGATTTCGCGACGTACCCAGGTCAACGTTCGGGAAGGACGTCTGGAAACCATCGTTTGGAGGCTTCGTGGACGTGAACATCGAAAGGACGGGAAAGATCTCCCTCAGGAGCTTGATCGATGCTTCGGTGGTGGAGAGCTTTGGAGCCGGGGGGAGGACCTGCATCACCTCTCGAGTCTATCCTGTGGAAGCGGTGGGGGAGGGCGCACACCTGTTCGCCTTCAACAACGGGGACGCCGCAGTGAAGGTCTCGAGCTTGAAGGCGTGGCAGATGCAGGCGCCCAAGCACATGAACTAGCTCTAGAACTTTTAATTAATTTTCTTGTCCTGATCAGCGCCGGTGATCCGTACCCTTCTATGCTTCCTCATGCATGCAGTTTTCATACAAATAAGAGAGAGATAGCTGGCTGGCCCATCTGTGATTCTGTGAGGGTTTTGCCTACTCATGTGTAGTACTTGTGGGGTTTTGCCCTCCCCCGGGAGAGCGATTATTAACATGGGGGATTGTGGAAATTAAATTTAGCACTTTGACAAAAAAAAAA

>TRINITY_DN25681_c0_g2_i1_2
GATCTCCCGAGATTTTACCGAGATCTCTTCTCTCCAAGCCCTCCGAGACAAAGCCGAGATCCGATATGGCAAACCTTGTGTGTGACTTCCTGTTTTTATCAGAAGCTCTAGAGTATTCTGAACTAATGAATTACTGTGGATCTGACCTGCTATCGTGATGCTTTTTAAATGGCATTGATAGGCTATCTGTCATATGACATCTTTATTGATGTTCTATTCTTGCATCTTCAACATATTGCTGACCAATAGCTATTTTGCCACAGGTGAATCAGGGCCTTACTGTCTATGGCAATAAAGGGAGCACAGATCAGCATGCTTACATTCAACAGTTACGGGAGGGGGTCCAT

>TRINITY_DN27307_c1_g2_i1_4
TGTTGTAGTCGCCAGACTCAAACAACCCCCATCTCATCCCCCGCGACCGCGCCGCCATTAGCCCACCCTTTCCTGCGGTCGAACGGGCGACGACCTCGATGGACTTCTCCTTCTGCGCATCTTAGCTCCCATTTGCCTCTCCTCCTCTTCTTCCCTCTCTCTCTCTCTCTGCTGCCGCCGACCTCCATGGCGGCGTCCATCTACGGCATCTGCTCCTCCTCATCCTCCGCCTTCAAATGCCACAAGCTCTTCCCTCACTCTCCCTCCTATAATGTCAAAGCGCTCCCGCCGGCCAAGTGGGCGCCGCTGCACCACCGATTCCGTCCCCTGCGCTCCGTCGCCAGGGACACTCCCCCCGCCAACCTCCCCACTGTCGGTGACGCCGCCAGCCCCTCGCCGCTGGAGTTGAAGAAGAGGACGGGGGCGCCCGTGGAGAAGGACCCGGTTGTCCTCTGGCGGCGCTACGTTGACTGGCTCTACCAGCACAAGGAGCTGGGGCTGTTCATTGATGTCAGCCGGATCGGGTTCACGGACGAGTTCTTCGACTGGATGGTGCCCAGGATGCAGAAGGCGTTCGCCGACATGCGGGAGCTCGAGAAGGGGGCCATCGCCAACCCTGACGAGGGCCGGATGGTTGGTCATTACTGGCTGCGGAACTCCAACCTCGCCCCCAACTCGTTCCTGAAGACGAAGATTGAGACCACGCTTGACGCGATCTGCCAGTTCGCCAACAGAGTTGTCAACGGCGAGATTAAGCCTCCATCATCTCCAGCTGGCCGGTTTACCCATGTGCTTTCTGTGGGAATTGGCGGATCAGCTTTGGGACCCCAGTTTGTTGCAGAGGCGTTGGCTCCTGATAATCCTCCCCTAAAGATAAGGTTTATTGATAATACAGATCCAGCTGGAATTGATCATCAAATTGCACAGCTGGGGCCAGAGTTAGCTTCTACTCTTGTCATTGTGATATCCAAGAGTGGAGGAACCCCTGAAACACGAAACGGTTTATTGGAAGTGCAGAAGGCTTTTCGAGAAGCAAGGCTAGATTTCTCAAAACAGGGTGTTGCAATAACGCAAGAAAATTCTCTGTTGGATAATACTGCAAGAATTGAAGGGTGGCTGGCAAGATTTCCAATGTTTGATTGGGTTGGTGGCAGAACTTCAGAGATGTCTGCTGTCGGTTTACTTCCAGCAGCACTGCAGGGAATTGATATCAAAGAGATGCTTGCTGGTGCATCTTTAATGGATGAGGCAAATCGTACAACTGTGGTGAAGAACAATCCTGCGGCTTTGCTTGCGTTATGCTGGTTCTGGGCTTCTGATGGTGTTGGATCAAAGGATATGGTTGTGCTTCCGTACAAGGACAGCCTACTATTGTTTAGTAGGTACCTGCAACAGTTGGTCATGGAATCACTTGGAAAAGAACTTGATCTCGACGGAAACCGGGTGAATCAGGGCCTTACTGTCTATGGCAATAAAGGGAGCACAGATCAGCATGCTTACATTCAACAGTTACGGGAGGGGGTCCATAACTTTTTTGTTACCTTTATTGAGGTGTTACGTGATAGACCTCCTGGCCATGACTGGGAGCTTGAACCAGGTGTTACATGTGGCGACTACTTGTTTGGTATGATGCAGGGAACCCGATCAGCTCTTTACGCAAATGACCGCGAATCAATCACAGTCACTGTGCAAGAGGTGACTCCAAGATCAGTTGGAGCGCTAATAGCATTGTATGAGAGGGCTGTTGGGCTTTATGCATCATTGGTCAACATAAACGCTTATCATCAGCCTGGTGTGGAAGCGGGAAAGAAAGCTGCTGGAGAAGTGTTGGCTCTTCAGAAGAGGGTCCTAGCCATTTTGAATGAAGCCAGCTGTAAAGAGCCGGTTGAGCCATTAACACTTGATGAAATTGCAGACCGATGCCATGCACCTGAAGAGATTGAAATGATTTACAAGATCATCGCACATATGGCTGCAAATGACAGAGCGGTTATAGCTGAAGGCAACTGCGGTTCTCCCAGAAGTGTCAAGGTTTACCTTGGTGAGTGCAACGTAGACTATCTATATGCCTGAGCGTTATAACCACACGGTCTTGTTTCTGTGCTCATCTGAAAGGCTAAAAGCAGATGAGTTAGACATGCACTTGGGATGGCCAAAATGGTGAGCGGCCAAGTTCGAAACGATGTCAATTTTTTTTGACGGTAGTGTGGTGGTTACGGTCGGTCTACCAAATAATTGTCATTGCCGGCTGTTGTGCCCCTTTCCCAAAACTAATGAGAAATACAGGAGCAAAAATGCCATTGTAGAATTTTAAGCAGTTCACATCATTCGTGCATGTTTTGGTTGTGAACTCTCATTTTGC

>TRINITY_DN29061_c2_g1_i3_4
GTGTCAGTTGGAATTGGCGGAAGCTTTTTAGGTCCTTTGTTTGTGCACACAGCTCTTCAAACAGATCCAGAGGCTTCAGAATGTGCAAAAGGCAGACAGTTGAGATTCCTTGCAAATGTTGATCCAATTGATGTTGCCCGAAGCATATCTGGCCTTGATCCTGAAACTACTTTAGTTGTAGTAGTTTCAAAAACCTTTACAACAGCTGAAACTATGTTGAATGCTCGAACACTGAGGGAATGGATTTCTTCTGCACTGGGTCCTGAGGCTGTTGCAAAGCATATGGTAGCTGTTAGCACAAATCTTGAGCTTGTACAGAAGTTTGGCATTGACCCTAATAATGCTTTTGCATTTTGGGACTGGGTTGGTGGTCGCTATAGTGTATGCAGTGCGGTTGGTGTCCTCCCTCTCTCTCTACAGTATGGTTTCACAATCGTTCAAAAGTTTCTAGATGGTGCGGCAAGCATTGACAATCATTTCCATTCCACCTCATTTGAGAAAAACATACCTGTATGTATTCTCCTGTATCTTTGTTCGAGATGTCCTTGTAAACTGTTAGCTCTATGGATTCACTATTTTTATCCAAACAGGTTCTTCTGGGTTTATTAAGTGTGTGGAATGTATCTTTTCTTGGTTATCCTGCCAGAGTGAGTATTTAATATATAACTAAGATCTTCAGTTCAACTGTAGCTTAAAGCAACGATATTTTCTCACAACTGTGTCCACATGTTCATCTGAAGAAAGACTATGCTTTCCTCTTCAGGCTATACTACCATACTGTCAGGCCCTGGAAAAATTTGCACCGCATATTCAACAGGTAAGCATGGAGAGCAATGGGAAGGGTGTATCTATTGACGGAAAACCTCTTCCGTTCGAGACTGGGGAGATTGATTTCGGAGAGCCTGGAACAAATGGTCAGCATAGCTTTTACCAGTTGATCCATCAGGGACGGGTTATCCCGTGTGATTTTAT

>TRINITY_DN17089_c0_g1_i2_1
AGTTGTCCATTTGTGGAGAGGAAAGTTTTGGAACTGGTTCTGATCATATTCGGGAGAAGGATGGGATTTGGTGAGCCTGAAGGCAATAGATTACATATCTGTGGTTTTATAAAATATTAAAATGTATACTCCTGCAGAAAGCAGATGGAGACAGCGTTTGCAAATCTGAGTTTGTAGAACGTAGCAGATTGCTGTAGCTTCTTTTGATATATTTTTTTAAGATTATCTTCCACCGGTTTGCATTGTCGTCCTTACTGCTCTGTATTTATGATGGGTGGAAACTGGATAGAAGTTTCTTCTTCTATCTTTCACAAAGTAGACACCTCAAGCAGTTCAGTGATTTTTTTGTCAGCTCATGTTTTTATTTTCCTAAGTTTGTGTCATATGAACAATTGCATATTTCAAACAGGGCCGTTTTAGCTTGGCTGTCCATCCTTGCCTATCGCAACAAGGATAAGAAACCTGGAG

>TRINITY_DN19349_c0_g1_i1_1
TGCTTCCATCTTTACAGAAAACAGAAGAAGATGCATGATATAACTTGCTTTTGTTTTCAATCAACCTCAGAGTAAGCCTGTAGATTTTTTCTTTTTTTAAAGAGTGGATGGTACCACTGGTATATAAATTGAAATAAACAGAAGAAATGTACAAGTATAGGCTGTACAGAAGGCCCAAGCATGTAGAGTATTAAGTTGACTTGTTGATATGTGCTAAAGATTTCTCTCTGTAAATGGCTGGATTGCATTCCTTGAAGAAAATTTATCTGTGTGCAAGAATTTGTTAACCACCATCTTTGCAACTTGGACTTATGGTGAGCACTTATCTGCTACCTCTACCTAATTACATTCAAAGCTGCAATTACTCTTCTCTCTTGCGTAGTTTTGTTCATTTTGCTGCCTTCCTAATCTCAGATGCCATTACTCATGAAGTTACACCAAAGTCATGCATTTAGCTTACAGTTATTAGGATGTGACCAAAGACATTTTCTTGGAAAAGATACAGTTATTGGCAAATCTGAAGTCTACTTCGTCCTTGTCATATTTGTCCAATCCCCCCCCAACCCCACACCCACCAGAAAAAAGAGTGTTTCTATGCTAGCTTGTGTTAAGATGTTGTAGTCCAGTATGCAATCCATTTAAAATTTCACTATCTTATTGCCAAAGAGAAAATGAATAATGTTAACGAATCCATTTTCTCAGAGATGTGCCTTACTGTATCATCGAATTCATCTTTGGGCTATCCGCTGAATGATTTAAAGATGGGAAGCAACAAAATGGACATGGAAGTATGCATATCAATTCAAGGAGACTGTTAGCGTAGGTGAGAACAGTATGCATTCTGTTACCTGGCCCAAGTAGTTAAATATGAGAGTAACAAAAATGATCCAAAGGATGAAAGAGGATTGAGCTAAATATGAGATATGAGAATCTCTTTGCAGGATTTGTTTTCTCAGATAGTTGTGTTTCACCTCTAACCAGAAATTGGAACCTTGTGTTACTTGTGACATGATTCACATGAACAAGCCTTTGTGACATGCGTACCCTTGGATAGTGTGGAATGGATTTAACCACCAAGTCTGAACTCTGTGCTTGAGTCACTACAATTAATAATTTATTCGATTACTACTGTTCTCCATTTGTTATAATGTCTGTCATTGGATGTTGGTAATATTAATATATTATGACTTGCTTCTAATCCTTTCACGACTCACTTAAAACTATCAGGGATGGTCTTATGTCAACCCCAGCTGTTTCTGCAGTCATAAGGAAGCAAAAGGCCAATGGTGGTTTTATAATGAGTGCGAGCCACAATCCTGGTGGGCCGGAATATGATTGGGGCATTAAG

>TRINITY_DN29622_c3_g3_i1_2
TTCACTGAAGTATGCGGATGACTTCGCATATACGGATCCTGTTGATGCAAGTGTAGCATCCAAGCAAGGTATCCGGTTTGTCTTCACAGATGGTTCCAGGGTTATATTCCGTCTTTCGGGAACCGGTTCAGCAGGCGCAACGGTCCGCATCTATGTAGAACAATATGAGCCCGACGTCGCCAAGCACGACATGGATGCTCAAGCTGCACTGAAGCCATTGATAGGTCTGCAGCTCCTGCGCCATCTGAAAACAGTAATTTCTTCTCCGTTGGTCTTCCTACAATAACCAGTTGGGTTTGTTTCAGATCTGGCGCTGTCCACATCAAAGCTGAGGGAGTTCACTGGCAGAGAGAAGCCCACTGTGATCACATAAAGGACGATAGTCATGAACCATTTCTTCCTGGAAGGACCAGCTGACGGGACCCGCGCAGAGCCGTGCATTCTATTCTTTGCTGCCATTTGATCTCTGATATATGATGGTTTAGGCTGCCCTTTGTAGTAGTAGT

>TRINITY_DN30162_c0_g2_i2_5
AGTGTAGCATCCAAGCAAGGTATCCGGTTTGTCTTCACAGATGGTTCCAGGGTTATATTCCGTCTTTCGGGAACTGGTTCAGCAGGCGCAACGGTCCGCATCTATGTAGAACAATATGAACCTGACGTCGCCAAGCACGACATGGATGCTCAAGCTGCACTGAAGCCATTGATAGATCTGGCGCTGTCCACATCAAAGCTGAGTGAGTTCACTGGCAGAGAGAAGCCTACAGTGATCACATAAAGGACTATATATAGTCATGAACCATTTCTTCCTGGTGTGTGTGTACCTTTTACAAATTTGCATGGACCAACTGATTTGATCATCTTATCTATATTGATCAGACCCTGGAACTGCAGGAAGGACCAGCTGACGGGACTTGCGCGGAGCCGTGCATTCTATTCTTTGCTGCCATTTGATCTGTGATATATGATGGTTTAGGCTGCCCTTTGTAGTAGTAGTAGTAGTCATGGATGCCCCTTTATTAGGTTGAAGAGGCTGCATCTCTTTCTTGTGTTAATATATAGAGTCACCCATCAACATTTCATCCTAGTCCAATAAATTGTTATATTTATTGTTAAAATTCCATTCCCAGTTGATGGGAATCTCTCAAATGAGCTCTTTCAGTTTCTGTGGATTGTGGAAAAACATATCCCAGAGTCACCGAGGAATGCAGGGATCTTACCACTGGATCTTGTGAGACGACCAGATTTGCAGCAAAAAAAATCATTAGTAGGTGTTGGTTTTGTAACAATTCCTGTGCTGTGTTTTTGGTCTTGAGATGAAGCGATGAGGGTTTTT

>TRINITY_DN18765_c0_g1_i4_2
GGCGGGCGTTCGACGCCGACGGCGCGGCGGCTCTGGTGACCGCGCTCCGGTGGAGCTACGGGACGGGGAGGACGAGGAGCTACGAGTGGAGGGCGGACCAGCTCCGGGCGCTGCTCCGGATGATGGAGGAGAGGGAGGGGGACGTCCTCGACGCCCTCCGCGCCGACCTCGGCAAGCCCGCTCTGGAGTCCTTCGTCCAGGAGATTGCTTTAGCAAAGGATGCGTGCAAGTTGGCTCTTAAAGAATTGATACGCTGGGGGAAACCACAGAAGGTTCCGACATCGATTACATCGTTCCCATCTTCCGCAGAAATCATCACAGAACCGCTGGGTGTTGTCTTGATCATTTCGGCATGGAATTATCCTTTCCTGTTATCTCTCGACCCAGTGATTGGAGCTGTTGCGGCCGGAAATGCTGTCTTACTGAAGCCATCAGATATCGCACCGGCAACGTCGTCGATGCTCGCAAGGCTCTTGCCGGAATATGTGGACAGATCCGCCATCAAAGTTGTGGAAGGGGGCGTTGCTGAAACGACAGCTCTCTTGGAGCAAAAATGGGATAAAATACTCTACACAGGCAGTGGTAAGGTGGCACGAATTGTGATGGCTGCAGCTGCAAAGCACCTCACACCGGTGGTCTTGGAGCTTGGTGGAAAATCCCCTGTTGTCGTCGATTCAAATGTCAATCTAGATGTTGCAACAAAGAGAATTGCTGTCGGCAAGTGGGGTTGCAACAACGGGCAAGCTTGCATTGCCCCAGATTATGTGATAACACCGAGGACATTTGCTCCAAAACTGGTAGATGCTCTGAAGACCACATTGGAGAAGTTCTATGGCAAAGACCCACTGGCATCAAATGACTTGTCTCGCATTGTGAACTTCAACCATTTCAGAAGGCTGACGGCTCTCCTGGACGATGACAAGGTTTCTGATAAGATTGTTCATGGAGGACAGAGGGATGAGACATCCTTAAAGATAGCTCCCACTGTTCTACTTGATGTCCCTCTTGATTCGCTGGTGATGAACGAAGAAATTTTCGGCCCGTTGCTCCCGATCATCGTCGTCGACGATGTCAAAGAAGCCTTTAACATGATCAACTCAAGAGCCAGCCCACTCGCTGCATACCTCTTCACCTCAGACAAGAAGCTTGAAGACAAGTTTGTGAGGACCGTCTCCGCCGGGGGGATGTGCATCAACGACACCGCTCTACACTTTGCAAACGCCAGCCTGCCATTCGGAGGGGTGGGGGAGAGTGGGATGGGTGCCTACCATGGCAAGTTCTCATTTGAGACCTTCAGCCACAAGAAGGCGGTCCTCCACCGGAGCTTCCTAGGGGAGGTCCCCGCTCGGTACCCGCCCTACACGCGCAAGAAACAGAGGCTTCTTAGGGCACTGCTGGACGGGGACTTCTTCGGCGTTCTCCTCGCTCTCATTGGATGGGCCCGGTAGCAATGTTGATGGACGACGGCCCATCAAGACATATGGGCCTCTTTTTATGTTAAGGCAGGCTGGGTTGGCCCAGTGATGAGTAATAACGGCCCATATTGACATATGGATCTCTTCTGGTTCTTGATATAAGGAAGACAGGATGGGCCCCATTTGACAAATAAGGACGGCCCATCTTGACATGTGGATCTCTTCTTGATGTAAGGGGGGCAGGATTCTTGTTCAGATGCCCATCATTCATGAGATCTGGCAGCTGTTTGGATGTAACCCTGTGACAAACGTGGATGGGAGTTTTCA

>TRINITY_DN20802_c1_g1_i1_1
CACATTGGAGAAGTTCTATGGCAAAGACCCACTGGCATCAAATGACTTGTCTCGCATTGTGAACTTCAACCATTTCAGAAGGCTGACGGCTCTCCTGGACGATGACAAGGTTTCTGATAAGATTGTTCATGGAGGACAGAGGGATGAGACATCCTTGTGAGTGACGAAGATATATACTCATGATCCCTCCCAAATGCAGTGTTGTCCAGTTCATTGAGTTAGCACATACCTCTGCCTGCTATAACCATCTTCACACACACTTCACAGAAAGATAGCTCCCACTGTTCTACTTGATGTCCCTCTTGATTCGCTGGTGATGAACGAAGAAATTTTCGGCCCGTTGCTCCCGATCATCGTCGTCGATGATGTCAAAGAAGCCTTTAACATGATCAACTCAAGAACCAGCCCACTCGCTGCATACCTCTTCACCTCAGACAAGAAGCTGGAAGACAAGTTTGTGAGGACCGTCTCGGCCGGGGGGATGTGCATCAACGACACCGCTCTACACTTTGCAAACGCCAGCCTGCCATTCGGAGGGGTGGGGGAGAGTGGGATGGGTGCCTACCATGGCAAGTTCTCATTTGAGACCTTCAGCCACAAGAAGGCGGTCCTCCACCGGAGCTTCCTAGGGGAGGTCCCCGCCCGGTACCCGCCCTACACGCGCAAGAAACAGAGGCTTCTTAGGGCACTGCTGGACGGGGACTTCTTCGGCGTTCTCCTCGCTCTCATTGGATGGGCCCGGTAGCAATGTTGATGGACGACGGCCCATCAAGACATATGGGCCTCTTCTTATGTTAAGGCAGGCTGGGTTGGCCCAGTGATGAGTAATAACGGCCCATATTGACATATGGATCTCTTCTGGTTCTTGATATAAGGAAGACAGGATGGGCCCCATTTGACAAATAAGGACGGCCCATCAAGACATATGGGCCTCTTTTTATGTTAAGGCAGGCTGGGTTGGCCCAGTGATGAATAATAACGGCCCATATTGACATATGGGTCTCTTCTTGTTCTTGATATAAGAGTACAGGATGGGCCCGATTTGACAAATAAGGATGGCCCATCTTGACATGTGGATCTCTTCTTGATGTAAGGAGGGCAGGATTCTTGTTCAGATGCCCATCATTCATGAGATCTGGCAGCTGCTTGGATGTAACCCTGTGACAAACGTG

>TRINITY_DN26053_c0_g1_i4_5
GAAATTCTTACAGCGCCCATCTCCTCCATACGGCAACAGGTGGGCCTCGTCGACCAACGGCTATTAATAGGTGCAGCTCGCAGGCCGCAGCAGATCCCTGCACTGCACAGCTAACCGAGCTGGCTTCGTTCGTACAGCTAGAGAGGAGCAGTAGCTAGCACCACCACTGTTGGCATAGGAAGAAGGAGTTTGAGCTAGAACTTAGCAGCGGCCGGCCGGCGGATCGAAGCCGAGGAGGACATGGCGGCGTTGGAGCAGAGCGTGGCGGAGCTGAGGGAGGCCTTCCAGAGCGGGAGGACGAGGAGCGAGCACTGGCGGAGGTCCCAGCTCAAGGCGCTGCTGGCGCTCGTCCAGCGCGAGGAGGACGCCATCTTCGCCGCCCTCCACCGCGACCTGGGCAAGCACAAGACCGAGGCCTACCGTGACGAGGTGGGGGTTCTGGTCAAATCCATCAGCTTTGCGCTGGACGGCCTGCGCGGCTGGATGGCTCCCAAGCGCGCTAAGCTACCGCTCATAGCCTTCCCGACGACCGGCCAAGTCATCCCGGAGCCGCTCGGCGTCGTGCTGGTCTTCTCCTCGTGGAACTTCCCCATCGGGCTGGCGCTGGAGCCGGTCATCGGTGCCATCACTGCTGGGAACGCGGTGGCGCTGAAGCCGTCGGAGTACGCCCCGGCCTCGGCTCAGTTCCTGGCGGACGCCATCCCCAGGTACCTGGACAACAACGCCGTGAAGGTCTTCCCCGGTGGCGTCGAGGTGGGGGAGCAGCTCCTGGAGCGCAGATGGGACAAGATCTTCTTCACCGGGAGCCCGCGGGTGGGGCGCATCGTAATGACGGCGGCGGCGAAGCATCTGACGCCGGTCGTCCTGGAGCTGGGCGGCAAGTGCCCCGGCATCGTCGACACGCTGACCAGCGTGAGCGATCAGAAGGTGGCTGCAAAGCGAGTGGTAGCTGGAAAGTGGGGGGCTTGCTGTGGACAAGCTTGTATAGGAATCGACTACCTGCTGGTGGAAGAAAAATTTGCATCGATCATGGTTGATTTGCTGAAGAAGACAATCAAAAGTTTCTTCAGAGATCCTAGCAACATGTCAAAGATTGTCCATAGGCAGCACTTTGTGAGATTGAAGAAACTCTTGGCGGATCCATCTGTTGCTTCCTCTGTCGTTCATGGCGGTTCCTATGACGACGACAAACTAAACTTTGAGCCAACCATCTTGCTGGATCCCCCGCTAGATGCGGAGGTGATGACCGAGGAAATATTTGGCCCGATACTTCCCATAATCACATTGAAGAAGATCGACGAAAGTGTTGCTTTTGTGAGGGACAGGCCAAAGCCACTCGCTATATATGTCTTCACCAATGATGAAGTTCTTAAGAGGCAAGTCATAGAGGGGACTTCCTCAGGAAGCGTCACATTTAACGACGCAATCATCCAGTTTGCTTGTGATGCACTCCCTTTTGGTGGCGTGGGTCAGAGTGGCTTCGGAAGGTACCATGGCAAATACTCCTTTGATACCTTCAGCCATGAGAAGGCGATCATGAAGAGGAGCCTGTCCTTGGAGTTTACTTTCAGATATCCTCCATGGAACGAGACCAAGCTCAAGTTCATCAGAGCTGTCTACAATTTTGACTTCATCTCGCTCGCGCTCCTCCTACTCGGACTGAAGAAGTGACATTTTAATCAATCAATTAATTATATATATATGCTTCTTGCAAGGCATGCCGTAGTGTGGGGACTCGTTTATGATTTATGTGATATATATATATATATATATGATGGTGCCGGATTCCAATTTATGAGATTGTGATTTCCTTTTGATCATGTGTGGTTCCCAATTTTATGAGATTATGGACCTATGCCAATGGCAGGAACCTAATGCAAGGCAG

>TRINITY_DN1888_c0_g1_i1_4
AAAAAAAAAGAATACCTTTAATTTCTCTGGTTTCTAATTGGTTTGTTTTGTTAATTACCTTGAGCCACGAAGGCACGTTGCTTCTGTATCCTGTCGCGAAGATCACAGAGTCGAACACCTCCTTCCTGCCGTCTGCGAACTCTACTACGCCTTCCCGCGCGAATCTTCGTATTGCAGGGACTACCTGTTTCAAAGCATCACGACGGCAACTTATATATACTTGTTAAGAAGATGTGAGAGATGGAAACAGAGCTTCCCGGAGCCAAGAACATGCAAGGGATAGTAACATCATTTGAGAAAGCAACAACATGTGTGGGTCTCAGCGGTGGTAAAGAGGACTAGGCTGACCTTGATGTGCCCGCTCCTGATCTTGGCGAGGGCTCCCACGTCCAGGACGGGGGTCTTCCCGGCGGTGTTCTTGAGCTGGAGAGGCCCCATCTCCGGCCGTTGCAGCCCCAGGCGCTGCGTGTCCCCCAGAACCGCCCTCGAGCAGAGCAGGAGCAACCTGTCGACCCATCTCACGGGGAGCCACCTGAGCAGGCTCATGGAGAGGCCGAAGGTGGAGCGACCCAGGATCTCCCTCGGCAACACATGCAGCTGCATGCAACGGCGGCGAGAACAATGGAAACCCTAGCTAATTAGTAAGTTGTTGTGTAAGTTGATGGGTGTGGGAGGCCACGGCGGAGTCGTAGACCAGAGCATATATATATATATGTTGTGCAGGGTTACCTACCTTATCTCTGACGACCATGGAAGTGTGGGCGCCATTGTTGCAGAGGTCCAAGCACACCTCCATGCCGGAGTTCCCGCAGCCCACGACCAGCACCCTCCTCCCTCCGAAGTCGTACCCGTTCTTGTAGGAGGACGAGTGCATGATGCTCCCCTGGAAGTCGGATATCCCGTCGATCTCCGGCAGCACAGCCTCGGCGTTCTCCCCCGTAGCCACGACGAGCCACTGGCAGATGAATTCCGTGGTGCCGCCGCCGTCGACGGCGGCGCGCACCCGCCAGAAGCCGATCACGGGGTCGTACTCGGCACGCCGGACCTCCACGCCGAACGCGGGCTCGATGGAGAAGTGCCGGACGTAGGCCTCCAGGTAGCTGATGAACTGGTCCCTCGTGGGGTACGTGGGGAATTCGGTGGGGAAGGCCAGGAAGGGGAGCTCGCAGAAGCGCTTGGGCAGGTGGAGGCGGAGGCGGTCGTAGGTGCGCAGCTTCCATGAAGACGCAATGCAATTCTCTCGCTCCAGGATCAGCGAAGGGACGCCCTTCAGCTTGAGGCAGGCGGCGGTGGCCAGGCCGGAGGGGCCTGCGCCGACGATCACGGGGCCCGGCACCCACACGCTCTTCGTCGTCGACGTCCCCGGCGCCGGCGCCGCCGTCGTCCCGCCTCGGCAGCTGCCGTTGATCTTGACGTCCACGACGGCGCCGGGGAGGTGCTCGTCTACGCTGCCGAGGTCCCACTGGGGCGGAAACACAGGTTTCCTTTGTATGGTGATCATCATACAC

>TRINITY_DN22798_c0_g1_i2_2
TTTACTTGTAGCTAGATACGGGGATGGGAAGCCTTGATGCGCCCCCTCTCACGCCCTTGATCAGTACGGGTTTCAACCCGCTGGACGTGGAGGAGTTCCGCATGCAGGCTCACCAGACCGTCGACTTCATCTGCAACTACTACAACAACGTCGACACCTACCCCGTCCTCCCCCGGGTCGAGCCCGGCTACCTCCGCCGCCTCCTCCCGCCGTCGCCACCCCTTCACAGCGAGCCCTTCGACTCCGTCCTCAGTGACCTCCACACCATCCTCCTCCCCGGCATGACGCACTGGACCAGCCCCAACTTCTTCGCCTTCTTCCCCGCCACCCTCTCCTCCGCCGCCCTCTCCGCCGACCTCCTCTCCTCCGCCCTCAACCCCGTCGCCTTCAACTGGCTCGCCTCCCCCGCCGTCACCGAGCTCGAGAGCCTCACCATGGACTGGCTCGCCCAGCTCATCGACCTTCCCGAGTGCTTCCGCTTCTCCGGCGGCAGCGGCGGCGGGGTTCTCCAGGCCACCACAAGCGAGGCCATGCTGTGCACCCTGGTCGCCGCGCGCGAGGCCGCGCTCGAGCGCGCCGGTCGCGACAGCGCCGCCAGGCTCGTCGCCTACGCCTCGGACCAGACCCACTCCACCTTCGCCAAGGCCTGCAAGATCGCTGGAATCCCTTCGTGCAATGTCCGCACCCTCCCCACGCGCAGGGAGGACGAGTTTGTTCTGTCGCCCGAGACGCTCCGAGGCGCCGTGGCGGCGGATGTGGAGGCCGGGCTGGTGCCCTTGTACGTCTGCGCCACCGTGGGGACGACGTCGTCCACGGCCGTGGACCCGGTGAGGGCGGTGGCGGAGGTGGCGGCGGCACACGGGGCGTGGGTGCACGTGGACGCGGCCTACGCCGGCAGCGCCTGCCTGTGCCCCGAGCTCCGGCATCACCTTGACGGCGTGGACATGGTGGACTCGGTGAGCATGAGCCCGCACAAGTGGCTGCTCACGGGGCTCGACTGCTGCTGCCTTTGGGTTCGGGACAAGGTCCGTCTCACCACCTCCCTCGCCATCAACCCCGAGTACCTGAAGAACCGCCCCAGCGACTCCGGCTCCGTCGTCGACTACAAGGACTGGCAGGTGGGCGTGGGCCGCCGGTTCAGGGCTTTAAAGCTGTACATGGTGCTGCGGTGCTACGGAGCGACCAACCTGCAGTCGCACATCCGGGCCGACATCCAGCTGGCTCAGGCCTTCGAGGGGATGGTCCAATCAGACCCCCGGTTTGAGGTGGTTGTGCCGCGGCGGTTCGCACTGGTCTGCTTCCGGCTCAAGCCGGCCACTCCGGGCGAGGGCGAGGGCGAGGTGGAGGCGTCGAACCGGTGGCTGCTGGAGGCCGTGAACAGCACCGGGCGGGCGTACCTGACGCACACCGTCTTGGGCGGCACCTACGTCATCCGGTTTGCTGTCGGGGCCTCGCTTACCCAGATGCGCCACGTGGAGGCTGCCTGGGGTCTCATCAAGGAGAAGACCGCCGAGATCAAGGGAGCAGTGGACCTCCCCGCCGCACGTGAGACTTAATCAATAGATTATTAGACGATCGCGGAAGTACTTTTACCTGTTATTTGAATTTGAATGCACATAATTGAGTATTTGAATTTGGATGTGAGTGGGTTCAAGCGAGGAAATAATAGCTCCGTGCTTTGTATGTGATATTTCTTATGATAAATGAATAAAAAATAATTTTATTTCGAAGAAAAAAAA

>TRINITY_DN26084_c0_g1_i5_2
CCCTCCCCACCACCACGCAGTGACAGAGGAGATGTGAAGCAGCATCACACCACATGGGGGGCTGAGAGAGAGGTGCAGCAACCGCTCCTCTTCTTCCCACCCTCCACCTGCGATAACAAACCAGTGCAGCGTGCCCTGGAAGATCGTAGCAGAGAAGACCTGTCCAAGGAGGCAAGGGAACAAGCGAGGCGGCGGAGCGCCATCTCTCTTTCACTCTTCTCTCATGGAGGGGGCTCTGAAGCCGATGGACGCCGAGCAACTCAGGGAGCACGCTCACCGGATGGTGGATTTCATCGCCGACTACTACAAGAGCATCGAGAGCTTCCCTGTTCTCAGCCAAGTCGAGCCCGGGTACTTGCATGGGCATCTACCTGATTCTGCCCCCGAGCATCCAGAGAAGTTGGAGAGTATTCTTGATGATATTCAGCAGAAAATTATACCTGGGGTTACACATTGGCAGAGTCCAGGTTATTTTGCTTACTATCCTTCGAATAGCAGTGTTGCAGGGTTCTTGGGGGAAATGCTTAGTGCTGGTTTCAATATTGTGGGGTTCAGTTGGATAGCCTCTCCTGCTGCAACAGAACTTGAAGTTATAGTCCTAGACTGGGTTGCCAAAATGTTGAAGCTTCCAGAGCAATTCCTTTCATCTGGGCATGGTGGAGGAGTTATTCAGGGAACCGCTAGTGAAGCAATTCTTGTTGCCTTATTGGCAGCTCGTGACAAAACGTTGAGGAAGATTGGAAAAAGCTCACTTGGAAACCTTGTAGTTTATGCATCTGATCAAACACATTCTGCTTTACAGAAAGCATGCCAGATTGCCGGCATCCATCCAGAAAATGTCAGAGTGATAAAAGCAGAATCTAGCACAAATTACTCACTCGATCCTGAAGTACTTCGCAAGACAATATCACTTGACATATCAACTGGCTTGATACCGTTCTTCCTCTGCGCTACAGTTGGAACAACATCTTCAGCAGCTGTAGATCCTTTGTCGGAATTAGGAAAAATAGCCACGGCTTATGGCATGTGGCTCCACATTGATGCGGCCTATGCTGGTAATGCATGTATTTGTCCAGAATACCGCCATTATATTGATGGTGTTGAAGAGGCAGATTCTTTCGATATGAATGCACACAAATGGTTGCTCACAAATTTCGACTGTTCCCTGCTTTGGGTAAAGGATAGAAGTGCTTTGGTCCATTCCTTGTCTACAAGTCCTGAGTTTTTGAAAAACAAGGCATCAGAAGAAAGAAAGGTGGTAGATTTTAAAGATTGGCAAATTCCACTTGGCAGACGATTCAGGTCATTGAAGCTATGGATGGTATTGAGACTTTATGGCACGGAATGCCTTCAAAATTATGTTAGGAATCACATTAAGTTGGCTCAACAGTTTGTAGAATTCATTAAGTCTGATCCCAGGTTTGAGGTTGTTACCCCTCAGATGTTTTCCCTTGTTTGTTTCTACCTTCATCCTCCTCCCAGCATTCAAGATGATGGTTACAAGCTGAACGAGAGCTTGTTAGATGCAGTAAATGCAAGTGGAAAGATATTTATGTCCCACACGGTCCTTTCAGGCAAGTTTGTGCTGCGTTTAGCTGTGGGAGCACCCTTAACCGAAGAG

>TRINITY_DN28614_c0_g1_i2_1
ATTCATCAAGTCTGATCCCAGGTTTGAGGTCGTTACCCCTCAGATGTTTTCCCTTGTTTGTTTCTACCTTCGTCCTCCTCCCAGCATTCAAGATGATGGTTACAAGCTGAACGAGAGCTTGTTATGTGCAGTAAATGCAAGTGGAAAGATATTTATGTCCCACACGGTCCTTTCGGGCAAGTTTGTGCTGCGCTTAGCTGTGGGAGCACCCTTAACCGAGGAGAGGCACATCAAGGAAGCGTGGAATCTAATTCAGGAACAGGCTAGCATCCTCTTGGGAAGCCTGTCACTTTGATTTGGTTGATATTATGGTACACTCTGGCATGATATATATACTCCACCATCACCAGGGAAAAAAAATTGGCAGAAGCATAAAGATTTTATTCTCTCTGTTACTTCCGCGCCTGGACAACCTTCAGTCTAGAAAGATAAAGAACAGGCTCCGTCAAGATGCAGCACTCATTCATCTGTGCAGGTGATTGGGATTTTCACCCTACTAAACATTTTTTTTCTTCTGACTCTGCACTGTAATGCTACTCCC

>TRINITY_DN24901_c0_g1_i4_4
TGGCGACCCGGCGACGCTGACCTGGTGCCCGAGCGGTCTCTTTCAGGAGATTCCTTCCTTTCTATAAACCCGAGCTCCCCAGCATCGGAGTCTCTCCATCCGCAGCAGCGCCATCACCAGATTCACCACGACCAGACGTGATGGGCGAGGCCGCACCGGAGTCGACCCTGCTTCCTCCGCACACCCCCGGCGACGTCAGCGGCATCCCCGACGAGGACGCCGTCGTTGTGAACCTCGACCACGGCGACCCGACCATGTACGAGCCCTTCTGGAGGGCGATGGAGGAGAAGGGCAGCATCGCCATCCCGGGATGGAAGACCATGAGCTACTTCTCCGACGGCGGCGGCCTTTGCTGGTTCATGGAGCCGGGCCTGGCCCGGGAGATCGTCCGCCTGCATCGCCTGGTCGGCAACGCGAGGACGGCCGGCTACCACATCGTCGTCGGCTCCGGTTCCACCCAGCTGTTCCAGGCCGCCCTCTACGCCCTCGTCCCGCCCGACGCCGCAGCACCCGTCAGCGTCGTCTCCGCCGCCCCATACTACTCGTTCTACCCTGCAGTAACAGAATACCTGCGATCGGGCTTATATAGATGGGCAGGAGATGCAAATTCGTTTCATGATGATGCCCCATACATTGAAGTTGTCTGCTCTCCAAACAATCCAGATGGGTTCTTGAGGGAAGCTGTTCTGAACAAAGAGAATGGTAAAACCATTCATGATCTGGCATACTACTGGCCCCAGTATTCATCCATTACTTACGAAGCCTCTCATGAGATCATGCTATTCACCTTCTCCAAGTTAACAGGCCATGCTGGAACCCGCATCGGGTGGGCTTTGGTGAAGGATAGGGATGTGGCAGCAAGGATGACCAAGTTTATTGAACTGAACACCATTGGTGTCTCCAAGGATTCACAACTCAGAGCTGCACAAATACTTGGGGCGATATCCGATGACTATGAACATCCAGATCCAACCAGGGTGGAAAGGTTCTTCAACTACGGCCAGCGCCTTTTGGCAGAGAGATGGGAGCATCTCAGGGAGGCTGTCCGGACCACCCGTTGCTTCAGTTTACCCGAATTTTCACCGTCATACTGTAAATTCACAGGGCACATGTCCGAGTTGCATCCTGCATTTGCCTGGTTGAAATGTGAAAAGGAAGATGTAGAGGACTGTGAGCAGTTCCTGAGGAGACTCAACATAATAGCAAGAAGTGGCAAGCATTCTGGCTCGGGGTCGAGGTACGTCAGAGTTAGCATGCTGGATACGGATGAAAAGTTTGATCTCTTCATAAAACGGATCTTGGCTATACGATGATGAGGGTGCATGTGAAGAAGATGTACTCTTACTTCAAGCTTCCAAAGTTTTCGTTTTGATCTCTTGAGGTGATTCAGAGGACAGTTAGAATGTCTTGCTAATAGT

>TRINITY_DN5975_c0_g1_i1_4
CAATTCACGATCTGGCGTACTACTGGCCGCAGTACACCCCAATCACCGCCGCAGCCGCCCACGACATCATGCTCTTCACCTTCTCCAAGATCACGGGCCACGCCGGAACCCGCATCGGGTGGGGATTGGTGAAGGACAGAGCGGTGGCAGAGAAGATGACCAAGTTCGTGGAGCTGAACACGATCGGTGTCTCCAAGGACTCGCAGCTCCGAGCGGCGCAAATCCTTGGAGCAGTATCCGACGGCTACGAACTCGGCGGCGGCGCCAGATTCTTCGACTACGGCCGCCGCGTCATGGCGGAAAGATGGCGGCGCCTCCAGGAAGCAGCACGGTCCACCGG

>TRINITY_DN16581_c0_g1_i1_1
CGCTCTCCAAGATCTACGACGACGGCGACGACGACGACGAGGCCTGCAACGGAGATGAGTTCAACTCTCTGAGGGAGGTGATGGAGAGGTTCGCGGGGGCCATCTCCGAGCTGGCCATGGAGCTCGCCGGCGTCCTCGCCGAGAACCTGGGCCAGCCGTCGGGGTTCCTGACTCGGAGCTGCGGCAAGAGCACGTGCTTCCTCCGCCTCAACCGCTACCCTCCCTGCCCAGTCTCGCCGGAGGTGTTCGGCCTGGTTCCCCACACGGACAGCGACTTCCTCACCGTGCTGTACCAGGACGAGGTCGGCGGCCTGCAGCTGATGAAGGACTCGGAGTGGGTGGCCGTGAGGCCCAACCCCGACGCCCTTATCGTCAACATCGGAGACCTCTTCCAGGCGTGGAGCAACGACGTGTACAGGAGCGTGGAGCACCGGGTGGTGGCGAACGGGGAGGTGGAGAGGTACTCCGTCGCCTACTTCATGTGCCCTTCGTACGACTCCGTCGTGGGCGCCTGCGGCGAGCCCTCCGTCTACCGGAGCTTCACCTTCGGGGAGTACCGCCGGCAGGTGCAGGAGGACGTCAGGAGGACCGGTCAAAAGGTGGGGCTCCCCAGATTCCTTCACCGTCCCAAACTCTCTTCATGATCCGGCTATGCGTACGGACAGCTAGCTAGCTAGCCATGGATCGATCTCACCCCGGCCGGCCCCTTCCATATATATCTCTCTATTCTCTCGATCTCTCTGCAGAGATCAGCGGAAGTAGCTAGAAAGAAGGGGAGGATGGGTGTGCATGGGAGGAGTAGCGCAGTATTGCTTGAACATGCATGCGACGAA

>TRINITY_DN16581_c0_g2_i1_1
AGAGAGAGGAATGGCCATGGCGGAAACCAAGTGCGAGCCCCCTCTCCTGGACCGCTGCGAAGAGCTTCTCTACGACTCTTCCCGCAGCTACGCCTCCCACCGGACCCTGGAGTACTACGGTAACGGCGGCGGCTGTGGCGCAGGGGAATGCGAGCTGCCCTTGATCGACCTGAGTGGTCTGAGCCGCGGCGGCGCGCAGGAGAGGCGGGCCTGCGTGGCGGCCATCGCCAGGGCGTCCTCCGAGTGGGGCTTCTTCCAGGTGGTCAACCACGGCATCAGCCGAGAGCTCCTCGGAGAGATGAGGAGGGAGCAGCGGAGGCTGTTTGGGATGGCCTTCCAGAGGAAGGCCAGCTGCTGCAGGCTCCTCGAGGGCTCGTACCGGTGGGGGGCGCCG

>TRINITY_DN17116_c0_g1_i1_1
AGGTGAAGTGTTTGTGCGAAACTTGCTCTCGTGCCGGCTGTGCTTGTATCGAAGTAGACAAGAGCTTCGACAGATCCGTTCATGGAGTTCGACCCGCCGTTCGACAGTGCCTACAAGTCCCTGCTGGACGCCATGGATCCCAGAGGGTGTCCCGCACTCGAGGTCGAGCAGTGCCGGCTGCCCCTCATCGACCTGGCCCGCCTCGACCTCGACGACGGCGCCGCCACCGGGAGGGAGCAGTGCAAGAGGGAGATCGCCGCCGCCGCCGCGGAGTGGGGCTTCTTCCAGGTGGTCAACCACGGCTTGCCGGCGGAGCTCCTGGATCGGATACACCGCGCGCAGGTGGACGTCTTCCGACAGCCCTTCGAGAAGAAGGCCCACGAGCGTCTCCTGGACCACTACTCCCCCGAGAGCTACCGCTGGGGGAACCCGTGCGCCACCACCCTCCGGCAGCTCTCCTGGTCGGAAGCTTACCATATCTCCACCACTCCATGTCCGGAAACCCAAAAAGCCAACAGCAGTACCCTCAGGTGCACCATAGAGGAGTACACAGCTGCGGTGTCACGGCTGGCCCGCAGGCTGGCGGCGGTGCTAGCCGAGCATCTGGGCTGCGGGGGTTCGCCGTTCGACTTCGACGAGAACTGCACCGGCAGCACCTGCTACCTCCGGCTGAACCACTACCTGCCCTGCGCGGCTCCGGCGGGGGTGTTCGGGCTGATCCCGCACACCGACAGCGCCTTCCTGACCGTTCTGCACCAGGACGAGGTGGGAGGGCTGCAGCTGATGAGAGGGGAGAGATGGGTCGCCGTGAAACCCATCAGGGGATCCCTCGTCGTCAACATCGGAGACCTGTTCGAGGCGTTGAGCAATGGGGAGTTCAAGAGCGTGCAGCACAGGGTGGTGGCGAATCCGGAGGTGGAGAGGTTCTCGGTGGCCTACTTCCTCTGCCCCTCTTACGAGACGGTGATCGAGAGCTGCAGGGAGCCGGCGGTTTACAGGGCGTTTAGCTTCAGGGAGTACAGGCGGCAAGTGCAAGAGGACGTGAAAGCCAAGGGTTCTAAGATTGGGCTGAGAAGGTTCCTCGCTTAATTAAGAGTGATCTAGCTAGTACTCGATCGATTGCCTATATGCATGGTGGCCAATATTGCTCTCTAGGTTCTGATGTGGGTGACCAAACACTCTTGTGGTGTCCCTT

>TRINITY_DN17998_c0_g3_i1_1
CCCACGCCATACGCATCTCCCCCACCAGGGATGCCACTTCTTCTCTCCTGTACTGCCACCCCCGTCGCTGCTGCTGCTGCTTCTCTCCACCCGCCCGCCTCGTCCTAGTGCGCTTCTGCTCTCGGCAAGAACTAGGATTCCGAGGACAACGGGAGTGCGTGTGGTCGGCTTGGTCGATGATGGCTGCCGCAGCTGCTGCGTCGTGCTCCACCGCGGCCGGCGTCGCCTCCGCCTATTTCGGGAGCAGGAGTGACCTCTTCGGGAGCTCCAGGGCCCTGCGCGGGGAGAAGCTCCAGCCGAGGCTGCTGTCGTCGTCGCGGCCGTGGGCGGGGCCGGTGCGGTCGTCGCTGGAGGCCGACGTTGCTGACATGAGCTCAAACGCTCCAAGAGGCTTGTTTCCTCCAGAACCCGAGCATTACAGAGGCCCAAAGCTGAAAGTGGCTATCGTTGGAGCTGGACTGGCTGGCATGTCTACTGCTGTTGAGCTCTTGGATCAAGGACATGAGGTTGATATATATGAATCACGTTTTTTTATTGGTGGGAAAGTGGGTTCTTTTGTGGACAAGCGTGGGAATCATATTGAAATGGGGCTGCATGTCTTCTTTGGTTGCTACAACAATCTCTTCCGGCTCATGAAAAAGGTTGGGGCAGACGAGAATCTACTGGTGAAGGATCATACACACACATTTGTAAATAAAGGAGGAGAAATTGGTGAACTTGATTTCCGTTTTCCAATTGGGGCACCAATACATGGAATACGTGCATTTCTGACAACAAATCAACTGAAGACATATGATAAAGCAAGGAATGCCGTGGCTCTTGCCCTTAGCCCAGTTGTTCGAGCTCTCATTGATCCAGATGGTGCAATGGTAGACATACGGAACTTAGATAATATTAGCTTCTCAGATTGGTTCCTATCCAAAGGTGGAACACGCATGAGCATCAAAAGAATGTGGGATCCAGTGGCTTATGCCCTTGGATTCATAGATTGTGACAATATTAGTGCCCGCTGCATGCTTACTATATTTTCTTTGTTTGCAACTAAGACTGAGGCTTCTCTATTGCGTATGCTCAAGGGTTCACCAGATAATTACTTGAGTGGTCCCATTAGCAAGTATATCACTGACAGAGGAGGCAGGTTTCACCTTAGGTGGGGCTGCAGAGAGATTTTATATGATAGGTCAGCTGATGGGGAGACATATGTCAAAGGCCTTGCCTTATCCAAGGCTACCAACAAACAAGTCGTAAAAGCTGATGCCTATGTTGCAGCATGTGATGTCCCTGGCATTAAAAGATTAATTCCTTCGGAGTGGAGAAAGTGGAATTTGTTTGATAACCTCTATAACCTAGAAGGAGTGCCAGTTGTCACAGTTCAGCTTCGGTACAATGGTTGGGTCACTGAATTACAAGACCTGGAACTGTCTAGGCAACTGAAACAAGCAGTGGGGCTGGATAATCTTCTCTATACTCCTGATGCTGATTTTTCCTGTTTTGCTGACCTTGCACTTACTTCTCCTGAGGATTACTACATTGAGGGCCAAGGTTCATTAATCCAAGCTGTACTTACACCGGGTGATCCTTACATGCGATTGACAAATGATCAAATAATACGTAAAGTTCATGAACAGGTTTTGGCACTGTTTCCATCCTCAAGGGGTCTGGAACTTACTTGGTCTTCACTGGTGAAAATTGGTCAATCCCTATATCGTGAAGCACCAGGAAAGGACCCATTCAGACCTGATCAGAAGACGCCGGTGAAAAATTTCTTCTTATCTGGTTCTTATACAAAACAAGATTACATCGACAGCATGGAAGGAGCAACCCTCTCCGGGAGGCAAGCGGCAGCCTACATCTGTGGTGCTGGGGAAGAGTTGGCAGCACTCCGAAGCAAGGTTGCCGCCGGTGAAGCACAAAGTTTTATGCCGGTGATGGGCTGAAGCTTGCATGATATGAAGGATCTTATTCTCCTTGTAGTAGTATTGACAAAAAAATGGGGTACAATCCCATGCATCCAAAATGATGAGATTGTGGAGAGCTCACTGCCGGTCGTATCGAGTTAGGTGTTTGTAATCCATATTTCCATAGTCCTGATGGGATGCTTTTCGCTGGACGTATTTGTTTGCCCATGCTTGTAAGTGATTCATACATCATTCAATAAATATACGAGCTTGCTGATTCCAGGTAAGGATCAGTATTATTTCC

>TRINITY_DN25479_c0_g2_i1_5
ATTCTAACCTTCATGCACGACGAGAAGACATCCAAGTCGGAGCTGCTCATCGTGAACGCCACCGACATGATGCTGGAGGCGTCCGTGAAGCTGCCCTCGAGGGTTCCGTACGGCTTCCACGGGACCTTCGTCAGCTCCAGAGACCAGCTGGAAACGCAAGCCTGACGGTGTATCCAGTACCGCCGTCACTGTACCTAGGATATATACTGTAGGTCCGGGCCGCTCACTGTCTTCTCCGGCACCGACGGGGGTGGGATTTCTACAGAGGGATGGTCTGGTCCTTCCGGCGTACGTCCCCGGGAAATCCCCCCCTGGTTATTTCTCATTTGTTCTTCGTGTAAATTCGATCGTGATTGCTGTTCTTCCATATGTATAATTACGAAGCCACCACATATATAGCTTGATCATGTGATCACTTCACAGACAGAGACCGACAGGTGGGTTTCAGTTTTAGTTCTTCGAGCTGAACTGGTTGCAGTCCACCGCCGTTCGTCACTCTTCCCCGTTCTGCTTTCTTGTCGGGGGCAGTCCTGCCGGGGTCTTGGGGCCCCGCAGAACTGTCCTGAGCAGCCATCTGTGTGCTACGTGTCGAGGACAAAGCGGAAACAGTCGGCGGCGTCAGCGCCCACCACATGCCGATCAGTTGTCCGTAGCGATGGCAGCTGCGGTTTTCTGTGGATCTGTAATTAATTTGCAAAGTTTCTGTCACACCGTAAATAAAACAAC

>TRINITY_DN26196_c0_g1_i2_4
ACGACAAACACCTAGCTAGCTGGGCAATCCCTTCCCCTCTCTCTCTCTATCTCTCTCCTCTCTCTCTTTCTAGATTTCTCTCTCTCTCTATCGATCTCTGTGCAAGAACAGGCTAGCGGCCCCCCACATCTCTCCCTCTCACAGATACTGCCATCTAAATCCCAATCTGAGTTCCGGAGAGGGCCATGGCTGCTGCTGCTGCTGCTGCTGCTACCAGTTTCGCAAAGAACCCCACAAGCAAACCAGACCTTCCCTCTCACCCTTATAACTCATCTCTACTTGGCACTTCAGTGGGGAGAGCAGCCGGCCGCTGCAAGTCCACCACGCCGGCGACGTCGATCTCCATCCGCTGTGCCGCGTCCTCCACACCCAAGGCGAATTCCCTCCTCGACTCACTAAGCCACTCCCCTCGAGACCTGTCGACCCAGCCATCCGCCTTCCCCTTGGCCGCTGCCGCCGTCGACACGACCCCCATTACCACGCAGGCCCAGCGGAGGCATGCACGCCGCCACGCCTCGCAAGAGAAACCCAACTGGAACCTCCTCCAGCGGTGGGCCTCGGCGGCGCTCGACACCGTGGAGGACGTGCTCATCCACAACATGCTCGAGCGCCAGCGTCCCCTGCCCAGGACCGCCGACCCCGCCGTCCAGATCGCCGGCAACTTCGCCCCCGTCGAGGAGCAGCCCGTCCGCAGCAACCTGCCCGTCGCCGGCCGCATACCCCCCTTCATCAACGGCGTCTACGTCCGCAACGGCGCCAACCCGCAGTTCGAGCCCGTCGCCGGCCACCACCTCTTCGACGGCGACGGCATGGTCCACGCGGTCACCCTCCGCAATGGCGCCGCCAGCTACGCCTGCCGCTACACCGAGACGCAGCGCCTCGTCCAGGAGCGCGCCATCGGAAAACCGGTCTTCCCCAAGCCCATCGGCGAGCTCCACGGCCACTCCGGCGTCGCCCGCCTCCTCCTCCTCTACGCCCGCGGCCTCGCCGGGCTGGTCGACCACAGCCGCGGCATCGGCGTCGCCAACGCCGGGCTCGTCTACTTCAACCGCCGCCTCCTGGCCATGTCCGAGGACGACCTCCCCTACCACGTCAGAATCGACCATTCCGGCGATCTCGAGACAGTAGGCAGGTACGACTTCGACGGCCAGCTGAAGTCCTCCATGATCGCCCACCCGAAGCTGGACCCCGTCTCCGGCGAGCTCTTCGCTCTCAGCTACGACGTCGTCTGCAAGCCATACCTCAAGTACTTCTGGTTCTCACCCAGCGGCGACAAGTCTCCCGACGTGGAGATCCCCCTCGACCAGCCGACCATGATGCACGACTTCGCCATCACCGAGAACTTCGTCATCATCCCCGACCAGCAGGTCGTCTTCAAGCTCCAGGAGATGGTCCGAGGCGGCTCCCCTGTGGTCCACGACAAAGACAAGAAATCCCGGTTCGGCGTCCTCCCCAAGTACGCCTCCGACGCGTCCCAGCTGAAGTGGGTGGAAGTCCCCGGCTGCTTCTGCTTCCACCTGTGGAATGCATGGGAGGAGCCGGCGACCGGCGAGATCGTCGTCGTGGGCTCCTGCATGACGCCGCCCGACGCCGTGTTCAACGACGGCGAGGGGGA

>TRINITY_DN28720_c0_g2_i1_2
TCGCCGAGCCCTGGCCCAAGGTGTCCGGCTTCGCGAAGGTGGACCTCGTGACGGGCGAGGTGCGCAAGTTCATCTACGGCGACGGCCGCTACGGCAGCGAGCCCTGCTTCGTGCCGCGCGAGTACTGCTCGCCGCGCCCGTCGAGGGCGGCGGCGGCGCCGCACAGGGAGGACGACGGCTACATTCTGACCTTCATGCACGACGAGAAGACGTCCAAGTCGGAGCTGCTCATCGTGAACGCCACCGACATGATGCTGGAGGCGTCCGTGAAGCTGCCCTCGAGGGTTCCGTACGGCTTCCACGGGACGTTCGTCAGCTCCAGAGACCAGCTGGAAACGCAGGCCTGACGGCCGGGGATCCAGTACCGCCGTCACTGTATCTAGGATATATACTGTAGGTAGACCGGGCCGCTCACTGTCTTCTCCGGCACCGGCGGGGGTGGGATTTCTACAGAGGGATGGTCCTTCTACGTCCCCGGGAAATCTCCGGCCCCCTGGTTATTTCTCATTTGTTCTTCGTGTAAATTCTATCGTGATTGCTGTTCTTCCATGTATAATTACGAAGCCACCACATATATAGATTGAGGTGATCACTTCCAGTACTCTTCC

>TRINITY_DN20992_c0_g1_i1_1
CGAGGGGTTATACGTAGGGAAACCGAACACCGGTGAAGGTTCCTGCGGACGTGTGTGAGGGGAGAAGAAGAAGGTCACTGGCTTCTGTCTCTCCTCTGCTTCTTTCAGTTCCTGCATCATCATTCTGCTCCTGCGAAGTCTTGTTTCTCAGGTGTTCTTCCTCCCGGTCTTCAAGATTCCGGTGGGTTCAGCTGACCTGTAGCAGCAGGCTTGTCATCGGATGTCGAAGAGGAAAGAAGGAAGGAGCTAGCAAAACCAGCAGGGAGGAGGAGCTAGCTAGCCGCTGGGGCCGGTGGAGCAGCAGCTGGGCATATTAACTCCGCCATGGCGGTTGCGTTCCTGCGTGTGGTCTCGCCGCCGGCGGAGGTCTCCTCCGGCCTCTGCTTTCTGGAGGCGGTTCGGGAGGGGACCCGCCTCGTCAACCCAAGGCATGACGGGGGCATGAAGAGAAGGAAGCCCGGGTTGAGCTTGCCGGAGAACAGAGGAAGCTGCTTCCCCATCCGGTCCAGTCTGGTGGCGAATCCGGCGGAGGTGGTCATCTCCTCCGAGCAGAAGGTCTACGACGTGGTCCTCAAGCAGGCCGCCCTGGTGAAGGAGCAGACGAGGGCTAACAGGGTTCCCCTCGACGTGCGGCCGGATTTGGCCGTCCCGGGAGCTGCCCAGTTGATGGGCGAAGCATATGATCGCTGTGGAGAAGTCTGCGCCGAGTACGCCAAGACGTTTTACTTGGGAACGCTGCTTATGACACCTGAGAGGCGAAAGGCGATCTGGGCAATCTACGTGTGGTGCAGGAGGACGGACGAGCTGGTGGATGGACCCAACGCCTCGCACATAACGCCGTCTGCTCTCGACAGGTGGGAGTCGAGGCTGGAAGATCTGTTTGAGGGCCGCCCATACGATATGCTCGACGCCGCCTTGTCTGATACTGTTGCCAAGTTTCCTGTCGACATCCAGCCTTTCAGAGACATGATTGAAGGGATGCGGATGGACCTCTGGAAGACAAGATACAAGAACTTCGACGAGCTCTACCTGTACTGCTACTACGTGGCGGGAACCGTGGGGCTGATGAGCGTTCCCGTGATGGGCATCGCGCCGGAGTCGCAGGCCACAACGGAGAGCGTCTACAACGCGGCCTTGGCTCTGGGGATCGCCAACCAGTTGACCAACATACTCAGAGACGTCGGAGAAGATGCAAGTAGGGGGAGGATCTACTTGCCACAGGATGAGCTCGCCCAAGCGGGCCTCTCGGACGGTGACATCTTCGCAGGGAAGGTGACCGACAAGTGGAGGGACTTCATGAGAAAGCAGATCAAGAGGGCGAGGATGTTCTTTGACCAGGCCGAGAAAGGTGTGACCGAGCTCAGCCAAGCCAGTCGATGGCCCGTGTGGGCTTCCTTGTTGCTCTACCGGAAGATCCTGGACGAGATCGAAGCGAATGACTACAACAACTTCACGAAGCGGGCGTATGTAAGCAAAGCCAAGAAGTTGCTGGCCTTGCCCGCCGCCTACGGGCTATCACTCGTGCGCCCGTCCAGAACCTCGCCGGGCCTCTCAAAAGCCACAACCTAGATGGCCACGTTGTATTCGGCCGCCACGTCCGCCCTTCAAATTAATGTTCCGAGAACTATACATATATTAATGCGTACAAAGCAGAAAATTAGTTTTTGTATGATACACAACAGTTCAGTGACGAG

>TRINITY_DN26480_c1_g2_i12_3
TAGAGAGAGAGAGATAAATGTCTGGGTGTCTTGTGTGGGTGATGCCTCCGGGGGAGAGCTGCTGCAGCAGCAGCTGCAACCAAGGGCCCTTGCTGCTTCAGCTCCTGGGGCGGGGGAAGAACTGGAGGAGAGCGGTGGCGCCCAGGTCGACCGGCAGGTCGCTGGTGTCGGGGAGCCTGGTGGCGAGCCCGTGGAGGTCGTCGGAGGAGAGGGTGTACGATGTGGTGCTCAAGCAGGCGGCGCTGGTGAGGGAGCAGAGTGAGGCGAGCAGGAAGGTGGAGGACGACGTGACCGTGCCCATGGATTGGAGCCTCCTGAACGAGGCCTATGACCGCTGCGGCGAGGTCTGCGCCGAGTATGCCAAGACCTTCTACCTGGGAACATTACTCATGACTCCACAAAGGAGAAAGGCTGTTTGGGCGATCTATGTGTGGTGCAGGCGTACTGATGAACTTGTGGATGGACCTAATGCTACACATATAACACCAACTGCTTTGGACCGGTGGGAAGAAAGATTGAGTAATCTCTTTGAAGGACGTCCATTTGATATGTATGATGCTGCTTTATCAGACACTGTCTCAAAGTATCCTGTAGATATCCAGCCTTTCAAGGATATGATTGAAGGAATGAGGTTGGACCTGAAGAAATCAAGATATATGAACTTTGATGAGCTCTATCTTTACTGCTACTATGTAGCTGGGACAGTAGGTCTAATGAGTGTTCCGGTAATGGGCATTGCTCCAGCATCAAAGGCTTCAACAGAAAGCGTATATAATGCAGCTTTGGCTCTCGGAATTGCTAATCAGCTTACAAACATACTCAGGGATGTTGGAGAGGATGCCAGAAGAGGTAGGATTTATCTTCCGCAAGATGAGCTCGCCCTAGCTGGTCTATCAGAGAAGGACATCTTTCAAGGAAAGGTTACAGACAAGTGGAGAAATTTCATGAAAGACCAGATCAAGCGAGCGAGAATGTTCTTTGATGAGGCGGAGAAGGGCGTGGCTGACCTTAATCCAGCCAGCAGATGGCCTGTGTGGGCATCATTGATGATATACCGACAAATCCTGGATGCAATTGAAGCTAATGATTATGACACCTTCACCAAGCGCGCATACGTAGGGAAAGCGTGGAAATTCTTGTCGCTGCCCATAGCATATGCCAGATCACTTGCACTTCCACCCAAGGGCATGGCATGAAGCTATCATCTTACTCACCTGTTCTGTTTATCTAAGCTATCTCTCTCAGGTGCCTTGAGGTGTTGGAATTTTTCATTGTAGATTCACCTACATGGAAGATATGGCAGCTTATTTTGAAGCTATTGAAATCTCTATACATGATTATTACCTTTTCAGGAAAAGCAAGAGCAAGTGTACATCTCTTTCTTTCTTTTGATGATGGAAATATCTGGTTAACACTGCTTTCTTCCTTTTCAAGGTTGAAGTTTGTACCCTGTGACCCATCTATTCTAGTTGTGGTTCATGTAAGGCTTCTTCCTCAAAGCACAAATAAAGATCAATATGAAGTTACCTATATGCTTCACATTGTGTGGGGATCTACAATTATTTCTTTGGACCTTGTATAATCATGTTATTTAGTTGTGAGATTAGGTCAATGAGATAAATGAGGGGTTAGTCCTGAGTGATCCTACAAGGCATTAACTGTAAATTTGATAGATTTCTAACACCTAGTGTTAGTCATGGAGGATTTTAAACTATTATTTTTAAAGCCCCTCGTTAGTCAAAAGATGTTTTGATTTAGACTTTAAATCCAAAGTTGAATCTCACTTCTGTCAGAGAAAAGTGTGTATTTTGAGATGCATCTAAATGACTAGGCATTTTCATATCATCATATGCTATAAAATGTGTTAAGTTGTCCATGGGAGAGAGAAAAGAAACAACAAGAACTCCAGATCTTCAGAGGGTATTGAACAAACTAAGATAGTGAAGACTTAATGTATTGACATACTATTGTGATAATATTATGTAATTTTTAAAAAATGAAACTAAAGTAAAAATCACATGAAACTAAAGTAAAAATCCTCTGCTTACTTGTAAAGTTTTATCATTACTATGAAGTACACAGTTTCATCGCATTTCATGGTGAGAGGCCCTGGATCTTTTACTTGTGGCCTTCATCTTTTGCTTATGCTCTTTGTTACAGGATGTTTTCAGACTATCAATGAATATAAGCTGTGCTACCTGTGCCTTCTTTTTTAAGTACTTGGTGTGGATTTCTTGCAACTGATTAAAGTTCTTCCACTTTAGAAGTTCTGCAATTGTTTCAGTTGATCATTTATGCTAATATCAATGGTATTTTCTGAATTCATTGGTGCCTTTTGGTTAGTTTTCTCTTGTCTCTCAAGTTTTTATTCCTGGTTGTGAGACGGGCTGTCTGTTTCTCTAAAACTTGAGCATTCTATTACCGATTGTTTTCTAATATAGATGGCGGCATGCAATTCTTGCTCAATAATAGCAAAGCGGATGCTGAAAGTTGATTTATTGGAGACTTCCCGATTTCATCTTCATAACACAT

>TRINITY_DN29667_c0_g1_i5_5
GTTTCTGCTCGAGGTCTGTAAAATAATGGCGGAATCATCTGGTTTTGTCGCCGGTGGCGCCGCTGGGTGGACCCCCGCTTCTTAGCCGGAAGTAACGTGGCTCTCGTCCACACAGGGCCTGGTGTGGCTGTAGCCTCTCGAGGGAGCTCTGAGAGGTCGCCGGCAGCTCCACGGTAGCGCTCAGCGAGAACGGACGGACCGCAGCTGGCCTCGCCGCCCGCCGGGAACTTTCCAAAACCAGTGACAGCGGGGAGGGGAAGAAGCCAGAAAAGGGAAGAAAGGAGGCTCCTTTTTCGCTCGCCGGCTCTCCTCACTTCCTTCTTCGTCTCCGTTGTTTCCTATTCCGTCGACCAGTAGCCTGGTTATTTCTGTCTGCGTTAGAGAGAGAGAGAGAGGAGATAGAGAGGGAGATAAATGTCTGGGTGTCTAGTGTGGGTGATGCCTCCGGGGGAGAGCTGCTGCAGCAGCAGCTGCAACCAAGGGCCCTTGCTGCTTCAGCTCCTGGGGCGGGGGAAGAACTGGAGGAGAGCGGTGGCGCCCAGGTCGACCGGCAGGTCGCTGGTGTCGGGGAGCCTGGTGGCGAGCCCGTGGAGGTCGTCGGAGGAGAGGGTGTACGATGTGGTGCTCAAGCAGGCGGCGCTGGTGAGGGAGCAGAGTGAGGCGAGCAGGAAGGTGGAGGACGACGTGACCGTGCCCATGGATTGGAGCCTCCTGAACGAGGCCTATGACCGCTGCGGCGAGGTCTGCGCCGAGTATGCCAAGACCTTCTACCTGGGAACATTACTCATGACTCCACAAAGGAGAAAGGCTGTTTGGGCGATCTATGTGTGGTGCAGGCGTACTGATGAACTTGTGGATGGGCCTAATGCTACACATATAACGCCGACTGCTTTGGACCGGTGGGAAGAAAGATTGAATAATCTCTTTGAAGGTCGTCCATTTGATATGTATGATGCTGCTTTATCAGATACTGTCTCAAAGTATCCTGTAGATATCCAGCCTTTCAAGGATATGATTGAAGGAATGAGGTTGGACCTGAAGAAATCAAGATATATGAACTTCGATGAGCTCTATCTTTACTGCTACTATGTAGCTGGGACAGTAGGTCTAATGAGTGTTCCGGTAATGGGCATTGCTCCAGCATCAAAGGCTTCAACAGAAAGCGTATATAATGCAGCTTTGGCTCTCGGAATTGCTAATCAGCTTACAAACATACTCAGGGATGTTGGAGAGGATGCCAGAAGAGGTAGGATTTATCTTCCGCAAGATGAGCTCGCCCTAGCTGGTCTATCAGAGAAGGACATCTTTCAAGGAAAGGTTACAGACAAGTGGAGAAATTTCATGAAAGATCAGATCAAGCGAGCGAGAATGTTCTTTGATGAGGCGGAGAAGGGCGTGGCTGACCTTAATCCAGCCAGCAGATGGCCTGTTTGGGCATCATTGATGATTTACCGACAAATCCTGGATGCAATTGAAGCTAATGATTATGACAACTTCACCAAGCGCGCATACGTAGGGAAAGCGTGGAAATTCTTGTCGCTGCCCATAGCATATGCCAGGTCACTTGCACTTCCCCCCAAGGGCATGGCATGAAGCTATCATCTTACTCACCTGTTCTGTTTATCTAAGCTCTCTCTCTCAGGTGCCTGAGGTGTTGGAATTTTTTCATTGTAGATTCACCTACATGGAAGATATGGCAGCTCATTTTTAAGCTATTGAAATCTCTATACATGATTATTACCTTTTCAGGAAAAGCAAGAGCAAGTGTACATCTCTTTCTTTCTTTTGATGATGGAAATATCTGGTTAACACTGCTTTCTTCCTTTTCAAGGTTGAAGTTTGTACCCTGTGACCCATCTATTCTAGTTGTGGTTCATTCAAGCTGCTGGATATTATTACCATGGCATGATCCTTGATCCTTGATGAAGGAAATACTCCCAGGGGCAGCTTTGCTCCTTATACTAGCTGCAGCCATGCTTGTGATCTGCCGGACACATGGTCGGCCAGTGATAGGTCCTTGGAAGAGTGGATTAAGTGGTCAGCTGCAGAAAGCATTTGTATCAGGGGTTCCAAAAATTAATCGAGCTGAGTTGGAGGCTGCTTGTGAAGATTTCAGTAACATTATCCAAGCATTTCCAGATTGCACTATTTTCAAAGGAACATTGTCAAGCGGAGTTGAGATAGCAGTTGTGTCCACTGCAATTGCATCTCTCAAAGACTGGTCAAAGCGTTCAGAGTCACTTTTCCAGAAAAAGATAGACAGTTTGTCACGAGTAAACCACAAAAATTTTGTCAATCTACTTGGTTTCTGTGAGGAGAATGAGCCCTTTACAAGAATGATGGTGTTTGAGTATGCCCCCAGTGGAACATTGTCTGAGCATCTTCATGTGAAAGAGCTTGAGGATCTTGATTGGGCCGTCAGGATGAGGGTTATTATGGGAGTTGCATACTGCTTACAGTACATGCACCATGAACTCAAACCACCTGTGGTGCATCCCAATCTACGATCAAATGCCGTATTCTTGACAGATGATAATGCAGCAAAGGTTGCAGATGTTGGCTTTTGGCAAGAGTTTATTGCCAAAAAGAAATTAGACGGAGATGATTTCACCGACATCTCAGAGTCACCCTCTACTCATACAGGAAGCAACATTTATGATTTTGGAATACTTCTACTGGAAATAATATCTGGAAGGCATCCACATTCTGAGGAACAAGGCTCTATACTGAACTGGGCTGTTGAATATTTGAACGATAAGCACAGTGTTAGCCACTTGGTTGATCCCACACTGAAGCACTTCAAAACCGACGAGTTGGATATCATATGTGAGGTTATTCAAGACTGCATTCATCAAGATCCGAAGACTCGGCCAACAATGAAAGACATTACCGCCAAGCTGAGGGAAGTGCTATCTATTTCTCCAGAGTCGGCGACTCCACGACTCTCTCCACTCTGGTGGGCAGAGTTGGAGATCCTCTCGTTGGAAGCAAGTTAAACAAGTTTGAGCTGCCCCCCTCTCCCTCCTTGTTTCTCTTCTAGAGTATGTGTATAATCTGCTTTTTTCTTTCCCCCCTCCCAAACAAATTTCTCATTCTTTTGTTCACTTCCTCTTTGTAATGGATGTAAACTATGGGATTTTACACAGGTGCTGAGGCGCATTTATACGTACTTGTATACTTTCAAAGACTCACCTACAGGGG

>TRINITY_DN28360_c0_g1_i1_2
GGATGGTCCCAATCCAAATGAGATCAATCAAAATTTCAGCTATTTACCTTTTGGTGGAGGACCAAGAAAGTGCATCGGTGACATGTTTGCTTCCTTTGAGACGATATGTGCTGTGGCTATGCTAGTGAGGAGGTTCAACTTTCAAATGGCCCCTGGAGCACCTCCTGTGGAGATGATTACAGGGGCAACCATTCACACAACTGAAGGTTTGAAGATGACAGTTACACGTAGGACCCACCCTCCAATTATCCCTAATTTGGAAACGAAAGTCGTCACAGTGAATGGTGATTACAAAGAGAACCCAGATCCGACTGAAGCAACGGCCGGCAATGCATCTGAAGAACAGGGCGAAGTATCAGCAGTGATGAATTCTTAGGTGGCCACCATGGCACGCATGTCCTTGTTCCAGGCACAGCAGTTGCTGCCCAATGGTCTGTGCCCTACAACACATCCAATTGTGAATATACAGAGTCGAGATTTTGCAGAAGGAAGTTGTGATTCTCAAGCTGTTCATAATCCCGTCCCATTATTTTGTCGGACCGCCACGGAACATGTGAGATCTAAAGCTTCAAAGTGCTCTGGCCTGGAGGAATGAGGCCAAAAAAAAGTGTCTTTACTCTTTATGAAACCGATTAAGATCTTTGTTTGTCTATTCGTCATGGAAACCTTGTCTTGATCTGTTTTCCTCTTTCAGAAATTCAATCAACGGCACTACTGCATCTCCGTGATACACAACAATCCCTTCCAGAATGGCTGTAAGTAAATAGCAATCCGGTGAGTCACTTTTGTAGTACGG

>TRINITY_DN29380_c0_g1_i7_5
CCCCACAATCCACTTCCCGCTTCCCCTTCCCCGCGCGCGCAGGTGGCACTCCATGGCCAATGCTGCCATGGCCGCCGCCGCCGCCGGCGCCAGCTTCTCCTTCCCGCAGATCCAGGGGCCTCACAAACCCCGCCTCCGCGCCTCCGCGGCGCCTCCCTTCGCCTATGCTCGTGGGAGCCCCCCATTGTTCTCCACTCGCCGCGTCGCGGTTGCCTGCTCTGCTTCGTCCTCCGGCGGCCCCGGGAAGGGCGATGAGAACCGGGCCGTGAAGGAGGTCGATAAGCTGATCGAGGAGAAGAGGCGCGCCGAGTTGGCCGCCCGCATCGCCTCCGGGGAGTTCACCGTCCAGCAGTCTGGCTTCGTCTCCTTGTTGCGGAAGGGCCTGTTGAGCTTGGGGCTGCCGGGGAAGTTCCTAGATGAGGTTTTCTCGCTTGCAAAGTTGGGTGCGGGTGCTCAAGGAGCGCCTGAGATTCCGAATGCGCGTGGGTCGGTGGCTGCCGTGGGCTCGCAGGCCTTCTTCATCCCTCTCTATGAGCTCTTCCTTATTTATGGCGGCATCTTCCGTCTCAACTTCGGGCCAAAATCGTTTCTGATAGTATCGGATCCTGCCATAGCGAAGCGTATACTGAGAGAAAATTCTAAGGCTTATTCCAAAGGTATCTTAGCGGAGATCCTAGAATTTGTGATGGGCAAGGGCTTGATCCCAGCTGACGGTGAGATATGGCGCATCCGGAGAAGGACGATCGTACCAGCTCTGCATCAGAAGTACGTTGCCACTATGATTGGCCTCTTTGGAAAAGCTACAAATAGGCTGTGCGAGAAGTTGGATGCTTCAGCTTCGGATGGAGAGGATGTGGAAATGGAGTCACTCTTTTCTCGTTTGACATTGGATATCATTGGCAAAGCAGTGTTTAATTATGATTTTGATTCTTTGACAAATGACACAGGAATAGTTGAGGCAGTTTACACTGTATTGCGGGAAGCAGAGATGCGTAGTACTTCTCCCATCCCTACTTGGGAGATTCCCATATGGAAAGACATTTCACCCCGGCAAAGGAAAGTTACAACAGCTCTCAAGCTGGTCAATGACACGCTTGATAGTCTCATTGCTATCTGCAAGAGAATGGTGGAGCAGGAAGAGTTGCAGTTTCATGAAGAGTACATGAATGAGCAAGATCCTAGCATTCTCCATTTTCTGTTAGCATCAGGAGATGATGTTTCTAGCAAACAGCTCCGTGATGACTTAATGACAATGCTAATAGCTGGACATGAGACAACTGCAGCTGTGTTGACATGGACCTTTTATCTTCTGTCAAAGGAGCCCAGTGTCATGTCCAAGCTACAGGAAGAGGTTAGTACACAAAGGAAGATTTGCAGCATAATTCTTCACATTTTATATGTTCAAATTTTCTTGGAGTTCTTTTTAGGTTGATACAGTACTGGGTGATCGGCTTCCAACTATTGAGGATATGAAGAGATTAAAATATACAAGTCGAGTAATCAGTGAATCTATGAGACTTTATCCCCAACCCCCAGTTTTAATTCGTCGTTCTCTAGAGGATGATGTGCTTGGAGGGTATCCCATAAAAAGGGGTGAAGATATTTTCATCTCAGTCTGGAACTTGCATCGCAACTCAAGATACTGGGTTGAACCAGAAGCTTTCAATCCTGAAAGATGGCCCTTGGATGGTCCCAATCCAAATGAGATCAATCAGAATTTCAGCTATTTACCATTTGGTGGAGGACCAAGAAAGTGCATCGGTGACATGTTTGCTTCCTTTGAGACAATATGTGCTGTGGCTATGCTTGTGAGGAGGTTCAACTTTCACATGGCCCCTGGAGCACCACCTGTGGAGATGATTACAGGGGCAACCATTCACACAACTGAAGGTTTAAAGATGACAGTTACACGTAGGACCCACCCTCCAATTATCCCTAATTTGAAAACGAAAGTCGTCACAGTGAATGGTGATTACAAAGAGAACCCAGATCTGACTGAAGCAACGGCCGGCAATGCATCTGAAGAACAGGGCCAAGTATCAGCAGTGATGAATTCTTAGGTGGCCACCATGGTGCGCATGTCCTTGTTCCAGGCACAGCAGTTGCTGCCCAATGGTCTGTGCCCTACAACACATCC

>TRINITY_DN29687_c1_g4_i1_3
AAGCGCAAGGCCATGCTCCGCGTGTTCGGGCCCTCCGTCGTGTCCAGCTTCTCCGGCGGCGACCTCGGCGGGTTCCACCACGTCGACAGCCTCTACAAGGACGGCATCCGCCTCAAGCGCGGCCTGCAGGAGGACCTCATCAACAAGTTCCCCTTCCTCAGCAAGATACAGGAGGCCTGCGAGGGGGTGTTCCGCTACGACAATCCCACCATCATATCAAGTAAGCACACAGCTGGTCGCCACGTACGTACGGCTGTCACATTCATCAGCTAGCTAAGTTAGCCATTAGTTTCTTCGTCCCCTGTTCTTGTAACAGTACTGGTACTAGTACTAGCTAGAATATATTAAAGAGTGTGGCTGACGTCTCTCTCTCTCTCTCTC

>TRINITY_DN29690_c0_g1_i13_2
GCCGCAGCCCCGCAGTTGGGGGGCCGCAGGGCGGGCAGGAGGCGGGAGGTGCGGGGGCGACCGGCCTCGACGGTCCGGGCGGTGATCAGCAGCGATGACAAGAGGGTGGGGACGGTGCCCTCGCCGGTGAAGCTGGACTCGGCGGGGGCGCCGTCGCAGGCGGTGTCGGAGGGGCAGGCGATCGAGGTGAGGGCGGTGGTCACCATAAGGAAGAAGATGAAGGAGAAGCTCGTGGAGAGGATCGAGGAGCAGTGGAGCTCCTTTATCCACGGGATCGGGCAAGGGATCTTGATCCAGCTCGTGAGTGACGAGGTGGATCCTGAAACCAAATCAGGGAAGCGGAGCACAGAGGCTGCTGTTAGGGGCTGGTTGCCGAGGCCAACTGAGCAGCTATACATAGCTGAATACACTGCTGATCTCACTGTGCCACCGGATTTTGGCCGCCCGGGGGCGATCCTTGTCACCAACGTTCACTCCAAGGAGGTCTTCTTGATGGAGATTGTTGTGCATGGTTTCAGCCAGGGCCCCATCTTCTTCCCTGCTAACACATGGATTCACTCCCAGAAGGATAATCCAAGCAAGAGGATCATCTTTAGCAATCAGGCATACTTGCCCTCGCAGACACCAGAAGGCCTCAAGGACCTTAGACAGGATGACTTGATCAGCCTTCGTGGGGATGGGAAGCATGAGAGGAAGAAGCATGACAGGATCTATGACTATGCACCATACAATGACCTGGGTAATCCAGATAAGAGCGGGGACCTTGCTCGGCCGGTGCTAGCAGGTGAGGAGAAGCCATATCCCAGACGCTGCCGCACTGGTCGACCATCAACGGAAACAGATCCTTTGTCTGAGAGTAGGATAGAGAAGCCCCATCCGGTGTATGTTCCCCGTGATGAAGCTTTTGAGGAGATTAAGGAAGGAACATTCTCAGCTGGAAAAATCAAGGCACTGTTGCACAACCTGATACCGCTTATGGTAGCCGCGCTCTCAGGATCAGACAAACATTTTGCTTGCTTATCTGAGATAGACGATCTGTACAAGGAAGGAGTAATTTTAAATCCTTCGGAGCATCAAGCTGCCAAAAAGTTTATGCTCCCAGGGATTCTAAAGAATATTATGAACATCCGTGAGCCCCTGAAGTATGATCTTCCATCAATAATTTCAAGAGATAAGTTCTCTTGGTTGCATGACAGTGAGTTTGCTCGCCAGGCATTGGCTGGTGTGAATCCAGTAAATATTGAAAGATTAAGGGAGTTTCCAATTGTGAGCAAGTTAGATCCGACAATTTATGGCCCTCCTGAGTCTGCAATAACAAAGGATGTTATAGAGAATGAGCTGAATGGAATGACTGTTGAAGAGGTATGGACGGTGAATCTGAAGATATGAAAATATTTTTTGGGTAGTAGCTTTGCTGTTTTGAATATCAATTTAACATAAAATATGATGGGTGTTCCAGGCAATTAAGCAGAACAGATTATTCATACTTGATTATCATGATATTCTCCTGCCTTTCATCAAGAGGATGAACTCTTTGAAAGATAGAAAAGCCTATGCTTCCAGGACAGTTTTTCTCAGCACTGGAACTACTCTTAAACCTATAGTTATTGAACTTAGCCTACCATCAAGTATATCTCCACGGAGAAAAAAGGTCTATACTCATGGGCATGATGCTACAACTCAATGGATTTGGAGATTGGCAAAGGCTCATGCTTGCTCAAATGATGCTGGTGTCCATCAACTAGTGAACCACTGGTTGAGAACTCATGCCTGCATGGAGCCATACATAATTGCTGCTCATAGGAACCTAAGTTCAATGCACCCAATTTTCATACTTTTGCATCCTCACATGCGCTACACCATGGAGATCAACGCACTGGCACGCCAAAGTTTGATAAATGGTGGGGGAATAATTGAAGCATGCTTTAGTCCTGGCAAGTATGCCATGGAGGTTAGCTCAGCTGCGTACAAGAGTCTCTGGCGATTTGACATGGAAGCTCTGCCAGCTGACCTAATTCGGAGGGGAATGGCAGTGGAGGATGCCACAATGCCATACGGCATAAAGCTCGTCCTCGAGGACTATCCTTACGCTGCCGATGGCCTCCTTGTGTGGTCTGCAACCAAAGACTGGGTGCATGAGTATGTCACACATTTCTATCCAGATCCCAACAGCGTCTCCTCTGACATTGAGCTCCAAGCGTGGTGGGACGAGATAAAGAACAAAGGACACCCTGACAAGCGCAACGAGCCATGGTGGCCTAACCTCAAGACCAGAGAAGACTTGGAGAGCATACTCACAACAATGATCTGGACAGCTTCAGGCCAGCATGCCGCCGTGAACTTTGGCCAGTACCCCTTCGGAGGGTACATGCCGAACCGTCCTGCGCTCATGAAGAAGCTGATCCCCGAGGAAGACGACCCCGAATACGAGAAGTTACTGAAGAATCCCCTGAAGGCGTTCCTGTCCTCCCTGCCAACCCAGCTCAAAGCCACCCAGCTCATGGCAGTCCAAGACACCCTGTCGACGCACTCCCCCGACGAGGAGTATTTGGGTGAACTGCCGCAGTCGCACTCCAACTGGATCAACGACCAGAGAATAAGAACATCCTATGAGAAGTTCTCGGCCAGACTGGAGGAGATTGAAGACATCATAAAGAAGAGGAACATGGATCTGTGCCTCAAGAACTGGACTGGTGCAGGGGTCCCTCCGTATGAGCTTCTGTTGCGTTCATCTGGCCCCGGGGCAACCGGCCGTGGAGTTCCCAACAGCATCTCCATTTGATCGATACTGCTCTGCGCACAAGCACATTTTGTCTTGCGAGGTGTACGGACGG

>TRINITY_DN31149_c0_g1_i9_1
CCCAGTTCGCAAGTCGGCGACTTTTAGTGTTCCAGAAGAGCGTCGCGGGATGTTCGCAGCTCAGCCGACAACCTTCCTGAAGCCCTCCCGGGCGGGCTCCGCCGCCGCAGCCCCGCAGTTGGGGGGCCGCAGGGCGGGCAGGAGGCGGGAGGTGCGGGGGCGACCGGCCTCGACGGTCCGGGCGGTGATCAGCAGCGATGACAAGAGGGTGGGGACGGTGCCCTCGCCGGTGAAGCTGGACTCGGCGGGGGCGCCGTCGCAGGCGGTGTCGGAGGGGCAGGCGATCGAGGTGAGGGCGGTGGTCACCATAAGGAAGAAGATGAAGGAGAAGCTCGTGGAGAGGATCGAAGAGCAGTGGAGCTCCTTTATCCACGGGATCGGGCAAGGGATCTTGATCCAGCTCGTCAGTGACGAGGTGGATCCTGAAACCAAATCAGGGAAGCGGAGCACAGAGGCTGCTGTTAGGGGCTGGTTGCCGAGGCCAACTGAGCAGCCATACATAGTTGAATACACTGCTGATCTCACTGTGCCACCGGATTTTGGCCGCCCGGGGGCGATCCTTGTCACCAACGTTCACTCCAAGGAGGTCTTCTTGATGGAGATTGTTGTGCATGGTTTCAGCCAGGGCCCCATCTTCTTCCCTGCTAACACATGGATTCACTCCCAGAAGGATAATCCAAGCAAGAGGATCATCTTTAGCAATCAGGTCTGTACAACACTTTTCTCCAGTCTCTGCGAAGATCCTTGTCTGCCCAAGTGCTAGACTATGTGCTCAGTGTTCTATTTGCCAACCTTGCTAGTTCTCCCTTTTACCTCATCAACTGAGTTTTGTGACTGCTCACAATGGCCATGAAAACAGGCATACTTGCCCTCGCAGACACCAGAAGGCCTCAGGGACCTTAGACAGGATGACTTGATCAGCCTTCGTGGGGATGGGAAGCATGAGAGGAAGAAGCATGACAGGATCTATGACTATGCACCATACAATGACCTGGGTAATCCAGATAAGAGCGGGGACCTTGCTCGGCCGGTGCTAGCAGGTGAGGAGAAGCCATATCCCAGACGCTGCCGCACTGGTCGACCATCAACGGAAACAGATCCTTTGTCTGAGAGTAGGATAGAGAAGCCCCATCCGGTGTATGTTCCCCGTGATGAAGCTTTTGAGGAGATTAAGGAAGGAACATTCTCAGCTGGAAAAATCAAGGCACTGTTGCACAACCTGATACCGCTTATGGCAGCCGCGCTCTCAGGATCAGACAAACATTTTGCTTGCTTATCTGAGATAGACGATCTGTACAAGGAAGGAGTAATTTTAAATCCTTCGGAGCATCAAGCTGCCAAAAAGTTTATGCTCCCAGGGATTCTAAAGAATATTATGAACATCCGTGAGCCCCTGAAGTATGATCTTCCATCAATAATTTCAAGTATGTATGAACCTACCGCTTGTTCGGATGGTAGTAATGTTATATTCAGATGCTCATAGTTAGTTTTGCAAGTGTAATTTAAACTTTTGTGCATACATGTGCAGGAGATAAGTTCTCTTGGTTGCATGACAGTGAGTTTGCTCGCCAGGCATTGGCTGGTGTGAATCCAGTAAATATTGAAAGATTAAGGGTATGCAACATACTTGGAGTGTTCATGCTTAATTCTAAATCCTCTATTATTTTATTTTAGAAATTTATTCTGAAACATTATTATAGGAGTTTCCAATTGTGAGCAAGTTAGATCCGACAATTTATGGCCCTCCTGAGTCTGCAATAACAAAGGATGTTATAGAGAATGAGCTGAATGGAATGACTGTTGAAGAGGTATGGACGGTGAATCTGAAGATATGAAAATATTTTTTGGGTAGTAGCTTTGCTGTTTTGAATATCAATTTAACATAAAATATGATGGGTGTTCCAGGCAATTAAACAGAACAGATTATTCATACTTGATTATCATGATATTCTCCTGCCTTTCATCAAGAGGATGAACTCTTTGAAAGATAGAAAGGCCTATGCTTCCAGGACAGTTTTTCTCAGCACTGGAACTACTCTTAAACCTATAGTTATTGAACTTAGCCTACCATCAAGTATATCTCCACGGAGAAAAAAGGTCTATACTCATGGGCATGATGCTACAACTCAATGGATTTGGAGATTGGCAAAGGCTCATGCTTGCTCAAATGATGCTGGTGTCCATCAACTAGTGAACCACTGGTTGAGAACTCATGCCTGCATGGAGCCATACATAATTGCTGCTCATAGGAACCTAAGTTCAATGCACCCAATTTTCATACTTTTGCATCCTCACATGCGCTACACCATGGAGATCAACGCACTGGCACGCCAAAGTTTGATAAATGGTGGGGGAATAATTGAAGCATGCTTTAGTCCTGGCAAGTATGCCATGGAGGTTAGCTCAGCTGCGTACAAGAGTCTCTGGCGATTTGACATGGAAGCTCTGCCAGCTGACCTAATTCGGAGGGGAATGGCAGTGGAGGATGCCACAATGCCATACGGCATAAAGCTCGTCCTCGAGGACTATCCTTACGCTGCCGATGGCCTCCTTGTGTGGTCTGCAACCAAAGACTGGGTGCATGAGTATGTCACACATTTCTATCCAGATCCCAACAGCGTCTCCTCTGACATTGAGCTCCAAGCGTGGTGGGACGAGATAAAGAACAAAGGACACCCTGACAAGCGCAACGAGCCATGGTGGCCTAACCTCAAGACCAGAGAAGACTTGGAGAGCATACTCACAACAATGATCTGGACAGCTTCAGGCCAGCATGCCGCCGTGAACTTTGGCCAGTACCCCTTCGGAGGGTACATGCCGAACCGTCCTGCGCTCATGAAGAAGCTGATCCCCGAGGAAGACGACCCCGAATACGTGAAGTTCCTGGAGAATCCCCTGAAGGCGTTCCTGTCCTCCCTGCCAACCCAGCTCAAAGCCACCCAGCTCATGGCAGTCCAAGACACCCTGTCGACGCACTCCCCCGACGAGGAGTATTTGGGTGAACTGCCGCAGTCGCACTCCAACTGGATCAACGACCAGAGAATAAGAACATCCTATGAGAAGTTCTCGGCCAGACTGGAGGAGATAGAAGACATCATAAAGAAGAGGAACATGGATCTGTGCCTCAAGAACAGGACTGGTGCAGGGGTCCCTCCGTATGAG

>TRINITY_DN18623_c1_g1_i4_5
AGGAAATTACAGTTTCAGTGGATGATGGAATCAGGCCTAGGACTTCAGTATCAAGCCTCGCAAAGCTTAAGCCAGTTTTTAAGAAAGATGGAAGCACCACTGCTGGTATTATCATCTTTCCATTTCCCACATTTGGCTCTGTTTTGTATTTGTTTAGATGGTGTCTTTTGATGCGTGTATTGTTTAGAGGGTGTCATTTGGCTCTGGTACCATCATCCTTCAATTGTATTGATTTGTCCTGACAAGTGACATGATGGTTATCTTGACAGGGAGAGAAGACTGGATATTATATATCCTGTGGGAGCACATGAGCTAGGAGGGTAGAGGTGACCAATTTTTTTTTCTTGGAAAGGATGACTGGTTGGAGTTGCTCTTTCTAGTTTCTGTCACATTCAATTAGCCAATGTTGTATTTGGCATTTATGTTTCTACATGTTTCTTGTGATTCTGGAGTAGGGTATTAGTGACAATCTTGAAAATTGCCATATGGACATTGAGAACTTAAATTTCTTCTCATCTCAGCCCAACAAGAAAATTAACATGGCAGGGGATATCTAATGCAAGCAGGCTCCAGTTTCTATAACTATTAAAGAGTCATGGTGTAAGCCTCTATGCGGGTCAAAATGAAGCTGAATGCTTCCTTTTGCGCAACATAATTTGTTATATTGACTGGTGATAAAGCTAATCTTTAAGAATTTGTCGTTGTTTGAGGACTTTAGTATATATGATATTCGACATTTTTTAGAATGCTGATTTTTTTTAACACAGCCATCTTGTATAAAGCAGCTTTAGTTGACCTTTTTTTAACACAGCCATCTTGTATAGGACTTCCTACTAGATTTGACATCATCTGGACTTTTTGTCTTAATAGAAAAAACTTACAGCTCTGAAACAGTCTCAACTTTCAATGAACTCTTCATTTATTTTAGCTATAACTTGTCTCGCTGTTGATTCTTAGCATGCTATGTTTGTAATTGTGATTTAGAATAGCTTGCTTAAGCTGATACAGCTGTTGTCTTGTTCCACATGAACAAACAGTACCATGGTAGAACTAATTTTGGACGCACAGCTGGAGGACCATTCATCACAAC

>TRINITY_DN21473_c0_g3_i1_2
TGCCTCCTGCTCCTCCGGAGCCTCCTCTCGTCGTCGAGCAGCAGGGAAGGATCGATGGAGAAGGCGATCAACAGGCAGAGGGTTCTGCTCCAGCACCTCCTCCCGTCGTCGTCCTCCCCGAACAACGCCTCGATCGTGTCGACGTCCACTTGTGCCGCTGGGGACAGTGCAGCATATCAGAGGAGTTCCTGCTTTGGAGATGATGTTGTGATTGTGGCTGCGTATAGAACTGCACTTTGCAAATCTAAGCGTGGTGGCTTCAAGGATACACATCCTGATGATTTGTTAGCACCCGTCCTTAAGGCACTGTTGGACGCAACTAAACTTGACCCAAGTGAAGTTGGTGATATTGTTGTTGGTACAGTTTTGGCACCAGGGTCCCAAAGAGCTAGCGAATGCAGAATGGCAGCATTCTATGCTGGTTTTCCTGAAACTGTCCCTGTCAGAACAGTGAATCGCCAATGCTCATCTGGTCTTCAGGCAGTTGCTGATGTTGCTGCAGCTATAAAAGCTGGATTTTATGACATTGGTATTGGAGCTGGTTTAGAATCCATGACTGTAAACCCAATGGCTTGGGAGGGGTCAATAAATCCTAAAGCTAAGATATATACACAAGCTCAAGATTGTCTGCTCCCTATGGGCATCACTTCAGAAAATGTTGCACATCGTTTTGGTGTTACACGCCAAGAACAGGATCAAGCAGCTGTTGAATCTCACAGGAAGGCAGCTGCAGCAACTGCTGCTGGTAAATTCAAGGATGAAATTGTTCCTGTA

>TRINITY_DN28879_c0_g1_i1_3
TGCAGAATGGCAGCATTCTATGCTGGTTTTCCTGAAACTGTCCCTGTCAGAACAGTGAATCGCCAATGCTCATCTGGTCTTCAGGCAGTTGCTGATGTTGCTGCAGCTATAAAAGCTGGATTTTATGACATTGGTATTGGAGCTGGTTTAGAATCCATGACTGTAAACCCAATGGCTTGGGAGGGGTCAATAAATCCTAAAGCTAAGATATATACACAAGCTCAAGATTGTCTGCTCCCTATGGGCATCACTTCAGAAAATGTTGCACATCGTTTTGGTGTTACACGCCAAGAACAGGATCAAGCAGCTGTTGAATCTCACAGGAAGGCAGCTGCAGCAACTGCTGCTGGTAAATTCAAGGATGAAATTGTTCCTGTAACAACAAAGATTGTTGACCCTAAAACTGGAGAGGAGAAGGAAATTACAGTTTCAGTGGATGATGGAATCAGGCCCGGGACTTCAGTATCAAGCCTCGCAAAGCTTAAGCCAGTGTTTAAGAAAGATGGAAGCACCACTGCTGGAAATTCTAGTCAAGTGAGTGATGGTGCCGGAGCAGTTCTTCTAATGAAAAGATCTGTGGCCGTGAAAAAGGGATTTCCCATTCTGGGTGTCTTCAGGAGTTTTACTGCTGTTGGAGTGGATCCTTCTATTATGGGTATTGGCCCTGCAGTTGCGATTCCTGCTGCAGTGAAATCAGCTGGTCTCGAACTTGATGACGTAGATCTTTTTGAGATAAATGAGGCATTCGCCTCCCAGTTTGTCTACTGCCGCAAGAAGCTTGGGCTGGATCAAGAGAAAGTGAACGTGAATGGAGGGGCCATGGCTCTTGGTCATCCATTGGGTGCAACAGGTGCCCGTTGTGTCGCAACGCTGCTCCATGAAATGAAGCGAAGGGGCAAGGATTGCCGGTTCGGTGTGATTTCTATGTGCATAGGCACAGGGATGGGGGCAGCGGCAGTCTTCGAAAGAGGAGATAGTGTTGACGACCTCACCAATGCTCGCAAGGTCCAACCCAACAATCTGCTATCCAGGGACGCTCTGTAGCTGCCTTGCCCTCTTGGATCTTCTAATAAAACAAAGAAAAGACTTGTTTGAGATTAATGCTTGAAGGCACTTTGTTGGATGCATGATGGAGATGCAAGGCCCGAAGCTGCGCTTCGGAATAAAGACCATTGTTGTTAGTCTAATTTTCTTTTCATCTTTATTGTCTTGATGTACCTTTTGGCCACGGGCAAGCATATATTTACAGGGAAGAATCAAATTTGTAGGAGGTTGATGTCAGTTGTTTGAGCTGCAAGATGATGAGGGTACTGGCATTGAAACCCCACAGAAG

>TRINITY_DN23699_c0_g2_i1_3
AAAAAATGGGATTAAGGACGGACATGATGGGTCGCTATTTGGTGTCCACTAGGACACCGAATAGCGACCGATCATAAATCGGTCGCTAACGTACAAAGTTAGCGATGCTAACGTACAAAGTTAGCGACGATTTCATCGGTTGCTATTCAGTCTCCATCCGAGAGACTGAATAGCGACCGATAAAAATTGGTTGCTATTCAATGGTTAAAGCTGTTGGTTGCTATTCAGTCTCCATCCGAGATACCGAATAGCGATCCATAAAAATTGGTTGCTATTCAATGGTTAAAGCTGTCTTTTGGTGTAGTGACTCTTGTACCGACCGATTTTCATCGGTTGCTATTCAGTCTTCATCCTAGAGATCGAATAGCGACCGATAAAAATCGGTTGCTATTCGGTGGTTAAAGCTGTCTTTTGGTGTAGCGACTCTTGTTGCCTTAACTTGAGACATTTACATAAATTTAGGAGTATCCGCTCTAGAGACGCACATATGAAGAAATTCAACTATCACCACACCGTTCAGAATAATTCACTTTAATATCATGTTGTTGGGTTAGAACTCTGAATTAAAACTCAGATGTTTAGTCTAAAAATTTCATGGTACATATATATCAGACAAATAACACAAAATTTCAATGTGATCAGACTAACAACAACACTAACATCACACACGTACACACACTCTCTCTCTAACAAGTAACAACACATCGATCTCAAAGTACAACTTAATAATTGATTATTTGCCCATTTCATGTCTCTTATTAATTATTTGCTTAGAGTACTGTGTGTGGGTCGGTGTAGACGACTACCTGAGCCAGGAGTGCAACCGCAACTTCCTCAACTGCATGAAGAGGTTCAGAAGCTCCCGGCGGCCGACGTTCAGGGGCAACCAGTGCTCGGTCGACGAGGTGGTGGACGTCATCTCCTTCTTCATCGAGGCCGCCCTCCTCGCCGGCAGGGCGCTCCACAGGCCATGATCATATATATCATCACACAGACCAAGCTATCTAGCTAGGTCCGGCGCGGCGGCCGCCGGCGACGGCAGCCGGTGCTCCCCCACATATCATACAAGTACTTGTACTCTCAAGTAG

>TRINITY_DN24506_c0_g1_i18_5
TCTCTCTCTCTCTCTCTACTCTCTCTCTCTAACCCAGGCTTTCCCGGCCGAAGGATATACATTTCTGGATCTCTGTGTGTCCTTCCACAGCACTACCACTTCGGATTATTCCAGCTGGAGGAGGGGGAGCCACAGCAGCAGCGGAGGCGGCAGCTGCAGAGAGAGAGAAGTGGGGGGCTAATGGCTACAGCCATTGGTGGACGGAGGAGGACGCAGCAGGGAGGGGCGGCGAGGCCCGCCGTCCTCTTGTCGTGTACCGTCCTCCTCCTGTCTCTCAGCATCCCGGCCGTCCCCGTCTCCGCCCTCAACGTCGGCATCCAGAGCGCCGGCGCCGATGTCTCCGTGAGTCTGCACTGCAGCAGGACGTGTGAGTCGGAGAACTGCTCCTTGCCGCCGCTGCTGAGGTACGGCAAGTTCTGCGGGATACTCTACAGCGGATGCCCCGGCGAGAAGCCCTGCGACGGCCTCGACGCCTGCTGCCAGATCCATGACGCCTGCGTCCAGTCCAAGCACGACGACTACCTGAGCCAGGAGTGCAACCGCAACTTCCTCAACTGCATGAAGAGGTTCAGAAGCTCCCGACGGCCGACGTTCAAGGGCAACCAGTGCTCGGTCGACGAGGTGGTGGACGTCATCTCCTTCTTCATCGAGGCCGCCCTCCTCGCCGGCAGGGCGCTCCACAGGCCGTGATCATATATCACACAGATCCCAAGCTAGCTCCCGGCACGGCGGCCGCCGGCGACGGCAGCCGGTGCTCCCCCACATATCATACAAGTACTTGTACTCTCAAGTAGTACTGCTCGATCTCCTATTTATAAATCTGTTGTATAACATGGCGATCGAGAGGGAGACAAATGGATTCTCTAGCTCACCCATATCTTGGATCATCTTTCTAATTATCTCTTCAAAAAACACCACCACCACCCCTCTCATGCATCAACAAGTGTTAAAGTTTCAGGCCTCTGATCAGCAATGTGCTTCCTTCCATAATTCCCTCTGTGTTTTGACAGATTGAACTGCCAGCACTGGTCCATCCGATCAGCTAGCCTATGTGTTTTT

>TRINITY_DN25462_c0_g1_i8_2
GACGCCTGCGTCCAGTCCAAGCACGACGACTACCTGAGCCAGGAGTGCAACCGCAACTTCCTCAACTGCATGAAGAGGTTCAGAAGCTCCCGGCGGCCGACGTTCAAGGGCAACCAGTGCTCGGTCGACGACGTGGTGGACGTCATCTCCTTCTTCATCGAGGCCGCCCTCCTCGCCGGCAGGGCCCTCCACAGGCCATGATCATATATATCATCACACAGATCCCAAGCTAGCCCCCGGCGCGGCGGCCGCCGGCGACGGCGACTTCAGCCGGTGCTCCTCCACATATCATACAAGTAGTACTGCTCGATCTCCTATTTATTATATCTGTTGTATAACATGGCGATCGAGAGGGAGACAAATGGATCATCTTTCTAATTATCTCTTCAAAAAACACCACCACCCTTCTCATGCATCAACAAGTGTTCAAAGTTTCAAGCTAGCTTTGATCGGCGCTCTGTGCTTCCTTCCATAATTTCCTGTGTTGTGACAGATTGAAGTGCCAGCACTGGTCCATCCGATCAGCTAGCTAGTTAAAGTTTTGTTGGGA

>TRINITY_DN25462_c0_g4_i1_2
TGGGTTATTGTTTCATGGCGGCGCGGGGCCGCGGACGGCGGCGATCGACACCGGTGGCGGCGGCGGCCTGAACAAATTACTTACTTATAATAACGTCCCACGGCGATCGATCGAGAACAGTGCCGCCGCTGCTGAGGTACGGCAAGTTCTGCGGGATACTCTACAGCGGATGCCCCGGCGAGAAGCCCTGCGACGGCCTCGACGCCTGCTGCCAGATCCATGACGCCTGCGTCCAGTCCAAGCACGGTTAATTATTAATCCCAACCCAACCCCCATCTCTCTCTCTCTCTCTAGATTTTGTTAGGTCAGAACTATAACTTACGTACTTGGGGGTTGAGTTAAGAAAAATTTTCACATTTATATCAGAAACATGGGTCTAGCAACAACACCCTTGTTGTCTCAACTTGAGACGTCTGTTGGACTTTACTTAGGATGCACATGTCGAAGAAATTCAACTATCATCACACAGTTTAGAAT

>TRINITY_DN11283_c0_g1_i1_1
GGCGTCGGTGGTGCTGGAGACCTTCCGGATGGACCCGCCGGTGCGGTACCAGTACGGCAAGGCGAAGCGGGACCTGACGGTGGAGAGCCACGACGCCGCGTTCCGGGTGAGGAAGGGGGAGGTCATCTTCGGCTACCAGACCTTCGCCACCCGCGACGAGCGGGTGTTCCGCGACGGCGGCAGGTTCGTCGCCGACCGCTTCGTCGGCGAGGAGGGGAAGGCGCTGATGAGGTACGTGGTGTGGGCGAATGGGCCGCACACCGAGCAGGCCACCGTCGGCAACAAGATGTGCGCCGGCAAGAACGTGGGGTTTCTCTTCGCCCGCCTGCTGCTTGTGGAGATGTTTCTGAGG

>TRINITY_DN13027_c0_g1_i1_1
ACACACGTTTAGCTAAGTAAGAGAAACTAATAGAATTCCAAGGCGCGCGGTGCAAGCAATGGCGCCCTCTCCTCTGGCGGACGAGAAGGTCCCTCTGGTACTGAGATCCACCATGGTCGACGGCGCCGGCGGCGAGGATCATGATCCCCCGACCAAGCTCCCAATCAAGACCATCCCGGGAAGCTACGGTATCCCTTTCTTCTCTCCCATCAAGGACCGCCTCGACTTCTACTACAACCAGGGCCAGGACAAGTTCTTCCGATCCCGCATCGCCACGTACGGCTCCACCGTCTTCCGCTGCAACGCCCCCCCGGGCCCCTTCATGGCCCGCAACCCCAAGGTGGTCGCCGTGCTCGACGCCCGGAGCTTCCCCATCCTCTTCGACACCTCCAAGGTCGAGAAGAAGGACGTCTTCACCGGCACCTACGTGCCCTCCACCGCCTTCACCGGCGGCCACCGCGTCTGCGCCTACCTCGACCCGTCGGAGCCCAACCACGCCAAGATCAAGCAGCTCCTCTTCAACGTCCTCGCCTCCCGGAAGGCCCACGTCATCCCGGAGTTCCGGGCGTCCTACTCGGACCTCTTCACCGCCATGGAGGTGCAGGTCGCCGGGGCGGGCAAGTCCGACTTCAACAAGCTCAACGACGCCTTCGCGTTCGAGTTCCTGTGCAGGGCTTTCTTCGGGGTGAGCCCCTCGTCGACGAGCCTCGGCTCCAGCGGGCCGACGAGGGCGACCAAGTGGCTCTTCTGGCAGCTCTGCCCGCTCATGGCCGGCCTCGGCCTCCCCAGGATCCTGGAGGACCTCTTGCTGCACACCTTCCCGCTACCACCCTTCTTGGTGAAGTCCGACTACCGGGAGCTGTACAGCTACTTCCGCTACGCGGCCACCGGCGCCCTCGCCGACGCCGAGAAGCTGGGGCTCTCCGAGGAAGAGGCGTGCCACAACCTCCTGTTCGCCACGTGCTTCAACTCCTACGGCGGCTTGAAGGTGCTCCTCCCGGGCATCATGCAGTTCCTGGCGACGGCCGGCGGCAAGCTGCACGCCCGGCTGGCGGCAGAAGTCCGATCGGCGGCGCCGCGCGTCGACGGCGGAGATCAGGTGATCACGTTCGCCGCGCTGGAGAAGATGGAGCTGGTGAAGTCGGTGGTGTACGAGGCGCTCCGGATGGACCCGCCGGTGAAGTACCAGTACGGGGTGGCCAGGCGCGACATGGTGATCGAGAGCCACGACGCGGCCTACCAGGTGAGGGCCGGGGAGACCATCTTCGGCTACCAGCCGTTGGCCACCCGGGACCCGCGGGTGTTCGACGACGCAGACGAGTTCGTGCCCGACCGGTTCGTCGGGGAGGAGGGAAGGAGGCTGCTCCGCTACGTGGTGTGGTCGAACGGGCCGGAGACGGAGAGCCCCACCGTGGGGGACAAGCAGTGCCCCGGGAAGGACTTGGTGGTGCTGGTCGGGCGGCTGCTGGTGGCGGAGCTCTTCCTGCGCTACGACACCTTCGCGGCGGACGTGGGCACTGTGGGCCTGGGGGCACAGGTCACCGTCACCTCGGTGTCCAAGGCGGCGGCCACTTGACGACGGAGGGCGCATGGCTTCGCCGCCGCCACCTCAACTGACGGAGGCAGGGGACGATTTGGTGGAAGACAAGGTGTGGTGGTGGTGAGGTCAAGTTGTTGGAGCAGGTGGACTACGTACGTAGAGCTAGCTTCCTGCAGGGCCGGGAATACCTGACGAGGTCCAACTAAGCGATTAGGTCAAGGTGGCAGTGATGGTTTTGTAGTTCCTGTTTGTCTACTAGCTAGTAAATTATTTTGCTAATAAACTGCAAGGTGGGTTGTTGACATGTGATC

>TRINITY_DN30468_c1_g1_i8_5
CAGAAGCTGCTGAAGCACGTGCTGTGGTCCAACGGGCCGGAGACGGAGAGCCCGACGCCGGACAACAAGCAGTGCGCCGGGAAGGACTTCGTCGTGCTGGTGGCGCGGCTGCTAGTGGTGGAGCTCTTCCTCCGATACGACTCGCTGGACATCCAGACGGGGGCGTCGGCGCTGGGCAACGCCGTGACGGTGACGTCCCTGAAGAGGGCGACCTTCTGAGATCGCCGGAGAACGGTGCAGAACAGCAGAAGAGAGAGACGGGAGGAGAGAGAGAGAATAGTTTGATACTGAGAGTGAGAGGTTCTCGTTCGGGAGTATGGTTGACCAGGTTCTCGAGAGAGGCGGAGACCACTGTGCTGAACAACCACCACCACACCAACAAAAGGTCTGGAGAGGCGGAAAGGGTAGTGGGCCGTACATATTTTACTAGATAAAATTTGTGTTCGACCATGTGATTTGATTATATTATTCTATAGTTGGGGTCATTTCATTTAATTATAGTATTATGCTTTTTACATGAAAAAAGATAGTGTTATGCTTTTTTATTCTCTCATTTTCGGCCGTGGTTGTAGACTCGTAGTGGTGGTGCGGTCTCAGGTTAATAAAAAAGAAATAAAAAAGAGAAATAAATAAAAAAAG

>TRINITY_DN21800_c0_g1_i2_3
TTTGTCAAGTGATCCGATTCCATGAGACACTTCCCACCAGCAGCCCCCTCACGTACTCCGGCCCCATCATTAAATGGGTTTGAGGAGCAACCACAAGGCGGCAGTCTCGGTGTAGGCGCTCTGGATCGCGGAGAGTAGTCGACAGGCAAGCGTGATCAGACACTTGAAGGGGGAGGAGCGAGGGGGAGTCAGCAGCATCGGAGGGGGATGGCGACGGGGAGAACGGAGATGGAGGTGGGGGCCGACGGTGTCGCCTTGATCACCATCGTGAACCCTCCCGTGAACTCCCTTTCCATCGATGTATTGTTAAGCCTTAAAGAGAATTATGAACAAGCTTTGCAAAGAGATGATGTTAAAGCAATTGTAATTGCAGGTGCAAGAGGAAAATTTTCTGGGGGTTTTGATATTTCTGCCTTCGGTGGAGTACAAGAAGGGAAAAGGAAGCAACCCAAGGTTGGCTATATATCGATAGATGTTATCACTGACATTTTTGAAGGTGCAAAGAAGCCTTCAGTGGCAGCTATTGATGGCCTTGCACTTGGTGGTGGATTGGAGGTCGCAATGGCTTGTCATGGGCGCATTTCAACTTCCACTGCTCAACTAGGTCTACCTGAACTCCAGCTTGGAGTCATCCCTGGATTTGGAGGAACACAACGACTTCCTCGTCTTGTGGGTCTTTCTAAAGCTCTCGAAATGATCCTTTTGTCCAAGCCAATAAAAGGAGAGGAAGCTTATACTTTGGGTTTGGTAGATGCCATTGTCCCACCTGAACAACTGATTAATACTGCTCGTCGTTGGGCACTTGATATTGTTGAATATAAAAAGCCGTGGGTCAGGAGCCTTTATAAAACAGATAAAATAGAGTCCCTTGGGGAAGCAAGGGAAATACTCAATTTTGCAAGAGTGCAGGCACAGAAGCAGGCAGCTAATCTTGAACATCCATTGGTATGTATTGATGTCATTGAGGAGGGCATTGTCTCAGGGCCACGTGCAGGGCTATGGAAGGAAGCAGAGTCTTTCCAGAAGCTTGTTTCTTCTGATACATGCAAGAGCTTGGTCAATGTTTTTTTTGCCCAACGTGCAACCTCCAAGGTTCCCGGAATTACTGACTTGGGTTTGAAGCCAAGGCACATTACTAAAGTAGCTATTCTTGGTGGTGGTTTAATGGGTTCTGGAATTGCAACTTCACTAATACTGAGCCACATTCCAGTGATCTTGAAAGAAGTAGATGATCGTTTCTTACAGGCAGGGATTGGACGAATCAGAGACATTTTGGAAAGACGTGTCAAGAAAGGAAAAATGACTGAGGAGAAATTTGAGAAAGCCCTCTCTCTCCTGACTGGTGTCCTTGACTATGAATGTTTTAAAGATGTTGATTTAGTAATTGAGGCAGTTATTGAGAATGTGCCTCTGAAGCAGCAAATCTTTGCTGATCTAGAAAAATACTGCTCACCCCACTGCATCCTTGCTAGCAACACTTCTACGATAGACTTGACTGTAATTGGTGAGAAAACTAAATCTCAAGATCGTATAGTTGGTGCTCATTTCTTCAGCCCTGCTCATGTTATGCCACTCTTGGAGATAGTCCGAACTCAGAGAACATCTCCTCAAGTAGTTGTTGATCTATTGGATATCGGAAAGAAGATTCGTAAAACACCTATTGTAGTTGGCAACTGTACTGGTTTTGCTGTCAACCGAATGTTCTTCCCTTACTCACAGTCAGCATTGTTATTTGCTGACCATGGATTAGACATTTATCAGATTGATCGTGCAATTACCAAGTTTGGAATGCCAATGGGCCCATTCAGAATGATCGATCTTGTTGGTTTCGGAGTTGGTATTGGATCAGGTATGCAATACCTCCAAAGTTTTCCTGAAAGATGCTACAAGTCAATGCTTTTACCAATTATGCTCGAGGATAAGCGCACAGGTGAAGCTAGTCGCAAGGGATTTTACAGTTATGACGATAAAAAGAAGGCTCGGCCAGACCCTGAAGTAAACAAATATATTGAGAAATCTAGGAACTTGGCTGGCGTTACCCCTGATCCCAAGCTCATGAATTTACCTGATAAGGATATCGTGGAGATGATATTCTTCCCTGTAGTGAATGAGGCTTGTCGTGTCCTGGATGAAGGAATTGCTGTCAAGGCATCAGATCTAGATATTGCCTCTATCATGGGCATGGGGTTTCCTCCTTACAGGGGCGGTGTTATGTTTTGGGCCGACTCCATTGGAGCGAAATACATATGCTCAAGGCTTCAGGAATGGTGCAACAAATACGGGGATTCCTTTAAACCGTGTCCCTACTTGGTCGAAAGAGCCAACAAAGGCATCTCTCTGAGGGCTGCTGGATATCTGGCAAAGCCTCGGCTGTAGCAGCAGGATGGATGCTTCCTCGATAAGAAAGAAAGAAAAAAATGGCCCATGCGGCTTGCTTAGGAGGTCGAATAACGGGAGTAAGGATAGGCCTGGTACCCGCCGAAGTATTTGAGTCTCCCCACCACGGCGTTCATCCATTTGGGTGTTCTCTGGGGATTTCTTATTATGGCTGTAAAAAGAGATTACCTTGGCTTGTTACAAAATCCAGACTTATTATTGGCTATCGCAAGACCTCCCTCTAATATCTATATTGTATACATGAGTCTCTTATTGTAGAACGATCTCTTGCATGCGTATTTACCGAAGTGCCATTTTTTAAACAACAAAACTTGTGCAAACAAGTGCTTCATCCCATCGGACCGTCGTAACATACTGCTTCATAAATCGGATAAACGCTGTAATTGTAGTTAGAAAATGGTCCCCGTATTTGAA

>TRINITY_DN23417_c1_g1_i7_5
CTCTCTCTCTCTCTCTCTCTCTCTCTCTCTCTCTCTCTCTCTGAGCTTGGCCTCTGCCTGCTGCTGCCGGCGGCGATGGCGAAGATCGGGGTATCGATGGAGGTCGGGAGCGACGGCGTCGCCGTGATCACCATCTCGAATCCGCCCGTTAACGCCTTGGCTCCCGCGATTGTGGATGGACTGAAGAAGACGTATACGGAGGCGATGAGGAGGGACGATGTCAAGGCCATCGTGCTCACTGGTGCGTCGGGCAAATTTTCTGGTGGTTTTGACATCAATGTTTTTGGAGAAATCCATCGAACTGGTCAACTCTCACTTTTGCCGGATGTATCTGTTGAACTTGTGGTCGACATAATGGAAGGTCTTACTTTGCGAGTATCTTGTACATGCTGCTTATCTACAAAATAATTTATTTTCACCTCCAAAGAATTAATTGTCTCCCTTATTTCCATCTTCTTATCATCTTGTGTTTTTATTGGAAAAACACTGAACTCAAAATATTTTCAGAGGGAAAGAAACCATCTGTTGCTGCTATTCAAGGGCTTGCACTCGGTGGTGGCTTAGAACTGACTATGGTTTTTCACTCACTTCAACAGTGCAACGATTCAATGTTTCAATATATTAAATTCTGTTGTGAAATGAATTTTGTGTTGTTAACTAATTGAAATACTACGACAAATTAACACAGGGTTGCCATGCACGGATCTCTACTCCCGATGCACAACTTGGTTTGCCAGAATTGACTCTCGGTGTCATTCCTGGGTTTGGAGGTACCCAACGGCTTCCAAGACTTGTTGGGTTGTCGAAGGCTATTGAAATGATGCTACTGTCGAAATCAATATCTGCCAAGGAAGGAAACAAGCTTGGTCTTATTGATGCCATTGCTTCTCGTGAGGAATTGCTGCGTGTATCTCGCCAATGGGCTATAGAAATTGCCATGCGACAGAAGCCTTGGCTGAATTCTCTTAAACGTACAGATAAAATTGGTTCTCTTTCTGAGGCACGTGAGATAATAAACGCTGCCAGGCAGCAAGCAAAGAGGATGGCTCCAAATATGCCACAGCATCAGGCCTGCCTTGACGCAATCGAAGAAGGCATTGTCTCTGGTGGATATGCTGGTGTTCTGAAGGAAGCTAAATTGTTCAAGGAATTGGTGTTGTCAAGCACTTCAAAGGGACTTGTTCATGTGTTTTTTGCTCAACGGGCAACTTCAAAGGTGCCTAAGGTTTCAGATATTGGCCTTAAGCCTAGAAATGTAAAGAAAGTGGCTGTGATTGGTGGCGGTTTGATGGGTTCTGGCATAGCTACTGCTCTTATACAAAGCAATATATCTGTACTTCTTAAGGAAGTTGATTCTACCTATCTGCAGCGGGGGTTAAAAATGATAGCAGCAAATCTTGAAGGTCTAGTAAAGAAAGGAGCAATGACACAAGATAAGATGAACAAGACGTTGTCCCTTGTCAAGGGTGTATTGGACTATTCAGAGTTCAGAGATGTTGATATGGTTATAGAGGCGGTCATTGAAAAGGTTCCACTGAAACAAACAATATTCAGTGACATTGAAAAGGCCTGCTCTCCTCAGTGCATATTGGCAACAAATACGTCTACCATTGATTTGAATATTGTTGGGGCAAAGACTAGGTCGCAAGATCGCATAGTGGGTGCTCACTTTTTCAGCCCTGCTCATGTGATGCCTCTGCTGGAAATTGTTCGGAC

>TRINITY_DN28545_c1_g2_i2_2
AAGGAAGGAAACAAGCTTGGTCTTATTGATGCCATTGCTTCTCGCGAGGAATTGCTGCGTGTATCTCGCCAATGGGCTATAGAAATTGCCATGCGACAGAAGCCTTGGCTAAATTCTCTTAAACGTACAGATAAAATTGGTTCTCTGTCTGAGGCACGTGAGATAATAAACGCTGCCAGGCAGCAAGCAAAGAGGATGGCTCCAAATATGCCACAGCATCAGGCCTGCCTTGATGCAATCGAAGAAGGCATTGTCTCTGGTGGATATGCTGGTGTTCTGAAGGAAGCTAAATTGTTCAAGGAATTGGTGTTGTCAAGCACTTCAAAGGGACTTGTTCATGTGTTTTTTGCTCAACGGGCAACTTCAAAGGTGCCTAAGGTTTCAGATATTGGCCTTAAGCCTAGAAATGTAAAGAAAGTGGCTGTGATTGGTGGCGGTTTGATGGGTTCTGGCATAGCTACTGCTCTTATACAAAGCAATATATCTGTACTTCTTAAGGAAGTTGATTCTACCTATCTGCAGCGGGGGTTAAAAATGATAGCAGCAAATCTTGAAGGTCTAGTAAAGAAAGGAGCAATGACACAAGATAAGATGAACAAGACGTTGTCCCTTGTCAAGGGTGTATTGGACTATTCAGAGTTCAGAGATGTTGATATGGTTATAGAGGCGGTCATTGAAAAGGTTCCACTGAAACAAACAATATTCAGTGACATTGAAAAGGCCTGCTCTCCTCAGTGCATATTGGCAACAAATACGTCTACCATTGATTTGAATATTGTTGGGGCAAAGACTAGGTCGCAAGATCGCATAGTGGGTGCTCACTTTTTCAGCCCTGCTCATGTGATGCCTCTGCTGGAAATTGTTCGGACAGAGAAAACTTCTCCTCAAGTGATTCTTGATCTCATGACCGTTGGAAAGGCCATAAGGAAAGTTCCCGTCGTAGTTGGAAACTGCACAGGCTTTGCTGTTAACCGTACCTTCTTTCCATATGCACAAGCAGCTCATTTCTTGGCAAATTTAGGAGTGGATATCTTCAGAGTTGATAGAGTGATTAGCAGTTTTGGCATGCCAATTGGACCCTTCCAACTCCAAGATCTGGCTGGATATGGTGTAGCTTTGGCTGTTAAGAAAGAGTATGCATTAGCTTTCAGAGGCCGTACTTATGAGTCTGATTTGATTGAGCTGATGGTGGAAAGTGGGCGCAATGGGAAAAACAACGGCAAAGGTTACTACATCTATGAAAAAGGAAGCAAACCAAGACCTGATCCAAGTGTGCGCTCAATCATTGAGGAGTCCATTAGACGTGCTAACATCTTACCTGGTGGAAAGCCTGTGACTCTGACGGATCAAGAGATATTGGAGATGATATTTTTCCCAGTGGTGAACGAGGCATGCAGAGTGCTGGATGAAGGTGTGGTTGTTCGAGCATCGGATCTTGATATTGCATCTGTTCTTGGAATGGGTTTCCCCAGATATCGGGGCGGACTTGTGTTTTGGGCAGATTCAATTGGTGCCGCTTATATTCATGCTACCCTGAGTAGATTGGCAGAAGCCTACGGTAGCTTCTTCAAACCTGCGCCATACTTGGAGGAAAGAGCAAAGAAAGGAATGCCATTGAGCATGATTCCCCCTGCGCCGTCACAAGGTTCGAAAGCCCGCCTGTAGTGGGAGAGGCGTCTCACACGACGCTCGAGCTGGTGCCAGGTGTCTTTGGAGAATAAAGGGTTCTAGTTCGTCTGCGGAGGGCCTCCTTGAGAAGGTTCTATCCGTCCACTATGGAAGTTCTGTTCAATCGTCGTCAGTTACCTGTAGACGTTTCAACCTTTCGTCAGTTTGCATCCTGACGATGTCTACTGCCTCCCCATATCCAGCCCAACTAGTTGGAACTTTCCCCCTTTTTAGGGTTTTCTTTAAATTCCTTGCAAAGGGAATGAAAAATGGAGAACAGTATATGGCTCAATTCTCATACAGAAGAAGGGTAAAACCAGCCTGAATAAACTTTTTCTTTCAGACCCCCCCTTCTGTACATGATCAAGAATTAAAACAACGTGTTATTATCTCGTATGTATGGGGATAAAGAGAAGCCCTCATAGACGAAGAAGGCTGATGTGGTCCGCTTGGTGTTCTGCAAGTTACACCCCTGTGCAC

>TRINITY_DN17150_c0_g2_i1_1
GCCACCTGTCGAAGAGATGGGCGCGAGATAGCATAGAGCAGTAGAAAGAAAGGGACGACGAACCAAGGGAAGGGGAACAAAAACATAGGTACGCTGCTCCGCCCTCCTCCGTCTCTCTCTCTCTCTCTCTCTCTCTCTCTATCTAGAGAAACAAGAACAGAAACGACCATGTCCCCGCCACCCTCTCTCTCTCTCTCTCTCTCTCTCTCATACAGTCCTCGGCGACTGTCGGAGGTTTCTGGTTGGGGATGGTTATACATAACAGCTATAAAAAAGAAAAGGTAGTGGGTATTGGAAATAATAAACCGAAAATTTTAATCTAAAAAAGTGGTTGCTTGATGGACTGGGGAACTGTTGTGATGAGAAAGCCAATCTGGGTTGGTGAAGAAAGTGTCCTAATTCGTCCCCACGCATCTGTTGGGTTCGATCTAATAAAGGAGTAGTGTTTGAACTCCCCCCGGCAGCTGATTTGATCATATCTGCTGAGCAGAGATGGATCTGTTGGGGTGGAGCTTCGCTACGTTTGCGGAGGAAAAGTGTCCGGTGCGATCTTTGTGACGCGTGCCTCCGCTTTGAATCCAGTTTGATCATTCGTTGAGTGGAAGTCTGGAACTGTCCGTCTGTTTTTGGTGTAATTTTTGAATTTAGGAACCCCACCCGGAAGTCTGGTTTCTAGGGTTGGGAGGTGTTTGGACTGTCTGTCGCCCTTCGTCCGTCTCTTCTTCGTTTCCTCTAGTGGTGATTCTGCTGCGCACTCGGAGGGAGGCAGACCTGGTCGATCCACTTTTGCTTCTTCGCTGTGTCGATCGAGTTGTGATGTTTCCCCATGAGGGTGGGGGTGGGTCCTTGAAATCTCCTTCTGATGTGATCCAGGGCACAGGGAGGAAGGAGGCGACCTCTCAGACATAAGATAAGTGTTTTTTTTTTCGTTGACGGCAGCAAGTGTCATCGGTAGGCGGCGCCAGAGGGGAATCCGGACTCTGGAAGGTATATCCCAGGAGGTTTTCCTCTATTGTTTTGTTTACTCCTGGGATTCAGGGAACTCGTTCCCACAGAGCGAGGTGGGAGTTGTCGTGGGACTAGAGATTTTTACAATTCCATGATGAACTTCAAGAGCATGGGTGGTCCAGATAGCAAGGACGTGCGGCCGTCCGCCGGCTCCTCTGCCGACGGCAACTTCGCGTCCACGCCACAATTGGCCGGGCTGGCGCGGCAACTTTCCGTCTATTCACTGACGTTTGACGAGTTCCAGAGCACAATGGGAGGGCTCGGCAAGGATTTTGGGTCGATGAACATGGATGAGTTCCTGAAGAGCATATGGACCGCTGAGGAGACCCAGGCAATGGCCTCGGCAGCGGCTCTGGGTGCACCGGCGGAGAGTGGTACTCCTGGCGCTGGGAACGGTGTGATGGGCCTTCATAGGCAGGGCTCCTTGACATTGCCCCGGACGATTAGCCAGAAGACGGTGGATGAAGTCTGGAGGGATCTGTTCAAGGAGGGCTCTGGCAGTGGGCAGGGCCTGTCTGCGTCTACTTTGCACCCGCAGCAGAGGCAGCCGACACTTGGGGAGATGACTCTGGAAGAGTTCCTTGTGAAGGCTGGGGTGGTGAGAGAGGACGTCTCGCAGCCTGCGTCTAGAAGCGTTATTTCTTCCTCAGACGTTGGCACCTGCGGGACTGGTAACATCGGTAACAACAATGTTTCCTATGGGGATGTTTGGTCCTCAAATATAAATAGTAACACTGGTCTCGCCCTTGGCTTCCCCCCAACGGCTCGGAGCAATATAACCATAGCACATAATCCAGTTCCAAACAATTCGTCAATCAATCTCACTGGTGGAGTGAGGCCTCCTTTTGCCCCTCCAATGCAGTTAGTTGACGCATCTGAACCAGCAACTCAGCAGGGGATGAGAGGCGGAGGGGGCATCATGGGGACTGGAGATCCATCAGTGAACAGTGGGCTGATGGTGGCCGCGAATGGTCTTCGGCCAGTTGGTGCAACAGTAGCTACAGGTTCTCCGGAGGATGACTTCCCTTCTGACAGACTGGGGAAGAGCAACCAAGATTTATCTTCTTCGTCACCAGTGCCATATGTGTTCAATGGAATACGTGGTAGGAAATGTGGCAGTACTGTGGAGAAGGTTGTGGAGAGAAGACAGAGGCGGATGATCAAAAACAGGGAATCGGCTGCAAGGTCACGGGCTCGCAAACAGGCTTATATCATGGAGTTGGAAGCTGAAGTAGCCAAACTTAAAGAGCAAAATCATGAACTGGAGAAGAAGCAGGCTGCGATCTTGGAGATGCAGAAAAATCAGGTATTGGAAAAGATTAATCAACAGTGTGGATCGAAGAAACCGTGCTTGAGAAGGACACAGACAGGCCCATGGTAAATTGAGGCTTGGACGGCAACGATCATCACAATAAAATCTTGTTTAAAAATCTTACTTCCTCAGAATTCTAGTTGTATATATAGAAGGCACATGTAGCATAAACTGTAAATGGAGTGGGAGGTGGTGCTGCCCTGTAGTCAAGTTTCTCATTCATATTGTTCTTGGTTTGCTTCATTCATGAATCCTGGCATATAGGTGGCTAGTGATCTTCACCTACACCTGCTGTGATGTTCACTATACTTCTATAGCTGACACACAACCATTGCCTGCGTGCGTAAGGCATGAAAAATGGGGCAGTTAAAACTAACGAAGGACACGTTTGCACCCTCATGTCTTTTCAGAGTTGAGCAGTGCCCAGTTTTGTTTCTCTCATTTTTGTCTTTTTTTTTTAGGAGGCCTTCTGCGCCTTGTTCACTATACTTCTATAGCTGACACACAACCATTGCCTGCATGCATAAGGCGTGAAAAATGGAGCAGTTAAAACTCATGAAGGACACGTTTGCATCCTCTTGTCTTTTCAGAGTTTAGCAGTGCCCAG

>TRINITY_DN20698_c0_g1_i2_3
GGTTGTGGAGAGGAGACAGAGGCGGATGATCAAAAACAGGGAATCTGCTGCAAGGTCACGGGCCCGCAAGCAGGCATACACTATGGAGTTGGAAGCTGAAGTAGCCAAACTTAAAGAGCAGAATGAGGAATTGCAGAAGAAACAGGCAGAGGTATTGGAGATGCGGAAAATTCAGATCATGGAAGTGATTAACAAGCAATGTGGATCCAAGAAGTTATGTTTGAGGAGGACACAGACAGGGCCGTGGTAAATCGAGCTTCGATGACGAATGATCAACATGACAAAAATAATGGGCCACAGAATCTCCTTGCATAAGGACCCAACCAATCAATTCAGGCATCAAGATCGTTGTTCACTAATAAGACTCATCTGTACACACATAGAAAGCACAGCCAAATATGTAAATTGAGTGACCGACCCATCATGCAGCTATGATGCACGGGGGCCCTTCCATTGGCCAGTGTGGGGCAGGCACCAGCCAATGAGAAGGCCGAGGACCTGTCTTTCTTGACTTGTCATGCAATAAGGTCCGTTTGGTTCCAT

>TRINITY_DN22939_c0_g1_i2_3
ATTCACAGGGCGTCCACTGGCTGCAGCAATACCACAAGGTGGCCCCTTTGCAGCAGCAACAACTGCTGTCTCAAAACCATCGCCCCACACAGCAGCAGCATCAGCAGCAGCGGCAGCAGCAGCAGCAAGATCAGCAGAGTGTGATGGCAGTGTATGCACCGAGCCGCCCGGTGGCGGTGCAGCCGGTGAGTGTTGGGGTGGGAAGCATGTTGGATGCAGGGTACTCCGACACCCAGCTGACCATGTCGTCCCCCATGATGGAGGCCCTCTCAGACACACAGACACCGGGGCGCAAGCGGGTCGCCTCCGGGGACATGGTAGAGAAGACAGTTGAGAGGAGGCAGAAGAGGATGATCAAGAACAGGGAGTCCGCGGCGCGGTCGCGGGCGAGGAAGCAGGCTTACACTAATGAGCTGGAGAACAAGGTTTCGCGCTTGGAAGAAGAGAATGAGAGGCTGAAGAAGCAGAAGGAAATGGAGACAATTTTATACAGCATACCTCCGCCAGAACCAAAGTTTCATCTTCGGAGAACGAGTTCAGCACCTTTCTAATGATGTTCATATGGGTTTTACTTCATGTGCTTTGTCTTTCTACATGTCTTAGATTTCAGATCCAAGGCCAGTACCACTTCTTTCCTGGTTCCCTATGAATGTGATGATCTATGGGCAACGATGCGGAAACCTGAACTGACTGCTCGGGAGCTCCTCGCTGCCAACGGTGCCATCTCTTGGGGCATTGCCATGTGTACATCTGTGTCCACCTTAGTTTGGAATTTACAGGCAAAAATAGAGATCCCTATGATTCCAATAATATCATACTGCTACTTACTAAAATTTTCAATGTGCTTCTAGCCTCCACTGATCTTTGCCTCTCCAAAT

>TRINITY_DN25103_c0_g1_i3_4
GAGAGAGAGAGAGAGAGAGAGAGAGAGAGAGATTTGGCAAGAAGAATTGCAGCGGCCCCGAAGCGCCCAGCAGCAAAAAAACAGGGAGTGGGAGGGGAACAGGCCAAGTTCACAGAAAGCGGACAAATTTGTGAAGGGATCTGGAGAAGCGGCGATGAACACCTTCAGTTGCCACTGCCCAGGACGCTGAAACGTTTCTTTTAGGGGTCGGAGAGGAGAAGAGCTGGAGAGAGGGAGCCGCACAGGAGGAGGGTTCTATCGGGGCCACTTCGTCACTCTGCCATGTTCTCCAGGAGCATGGGATCCGATGGCAGGGACGGAAGGCCGCCGGCGGGGATCGCGCGGCAGAGTTCGATCTACTCCCTCACCTTCGACGAGATCCAGACCACGCTAGGCGGGGTAGGCAAGGACTTCGGCTCGATGAACATGGAAGAGTTGCTCAAGAACATCTGGACTGCCGAGGAGACGCAGGCGGTCACCGTGGGCGCGGTGGGGGAGATGAGCAGCGCCGTCGGGGGTCTGCAGAGGCAGGGCTCCCTGACACTGCCTCGGACGCTCAGCCAGAGGACGGTCGACGAGGTGTTGAGGGACCTGTTCAAGGAGGGCAGTGGGATCGGGCAAGGAGGAGGCGCGGCGGAGGCCAGTGCCCAGCCGCAGCAGCGGCCAGCGATGCTGGGGGAGATGACGCTGGAGGAGTTCCTAGTGAAAGCCGGGGCGGTGATGGAGGAACCGGCGGCGCCTGCGCCAAGGCCGATCCATGGCTCTGCAAACACCGGACCCAGCAGGGCCAGTGCCTGCAACAATGGCAACATTCTCTATGGGGGGATCGCAAACCCACAGCTGAACAGCAGCCACCACGGGCTCGCCCTCGACTTCTCCCCCACAACAGTGAGGGGCGGCGGGGTTGCCGCATCCACTCCTGTTCCGACCAACCCGTCAGCCACTGGTCTAATGATGAATCTCGGTACCGTGAGGCCTCCCTTGCAGTTGGGGAATGCACATGAGCTAGCAACTCAGAGTGAGATGAAGGCCGGAGGGATCATGGGGATTGGAGATCCGGTGATGAATGCCGGACTGATGCCGGGCGTGGGCGGGATGAGCATCGGCGCGGCTCCGGTGGCTGCGGTGTCGCCTCAGGGCAACCTTCCTTCCGATGGGCGTGGTAAGGCCAATGGAGAATTGGCTTCTTTCTCTCCAGTGCCCTATGTGTTCAACGGAGGACTAAGGGGGAGGAAATGCGGCGGTGCCCTGGAGAAGGTTGTGGAGAGGAGACAGAGGCGGATGATCAAAAACAGGGAATCTGCTGCAAGGTCACGGGCCCGCAAGCAGGCATAC

>TRINITY_DN25376_c0_g1_i4_3
AAAAAAGGAAGGGAAAGGCTAGGCGCCATTAGCAAAGACGGAGGCTCCTCCCTTACACAGACCCCGGCCGGGGCGGCGCACAACGGTAGTAGACCAGCGCCAGGGCCCGGGACGGCCAAAAGATGACGGGCACTCCATGGCCCCCAACTCAAAGCTGGATCCAAGCATAGCTTTCTATTGCATGGCCCCTTAATTGAGCTTGTTGTCTTGGGGGCATGCCGATCCAAGGTATCTGAAACAGGGAAAGTTGGTGCAGCAGCAGCAGCTGCGCTTCCTCTGGTTCAGATCTGATTCGGCAGGTGGCAGCATGCGAGGCTGTTTGATCTGCTGTCGGATGCTTGGGCTGGGACTCCACCTGTGATGGTGAGACAGGAGGGGAATAAAAGGGACAGAGGAATTACCAGGACAAGCAAAAGAGGTTTGTTTTGGGAGTGGAGAAGAGGCCTCACTTGGTGGGATTACTGGGTTTTGTTGGTTTCCTTTGGTAGCTTTTGTTTTCTACTCGAATGCCATATGTTTCCATTTGTGGATTCCCCCCTTTTCTTCTGCTCATCTTGAGCTAGAGCATTGGCATCCCTCCTCTGTGTATTGGTGTTTGCCACGTATGTTTCCCCCCCTTTTTAATCTCCATTTGGTTGGTTTATTCTTTTCTTGTTTACCATGATCTGAGCTTGGGGGGGTGTTGGGGCATGGTGGAATCTGAGAGGGAAGTGAAAGGTCCCTTCTTTTTGCCTTATTTCTTGCCGAAAAGGTGAGACTTGCGGCCCTTTTAGGGGCGGCGGGGGGTTATTTGGTTTCTCCCGATTGCTCCTCCCAGATGCTATCTTCTGGTTTCTGGCAGTGGTTTCAATCCGGGTTTTGATTTGCCGCCGAGCAACATGATATGAAGGAAAATACAAAAAATTAGTTTTTTTGCGCTCAAAATTCGATTTTTTTCCCCTTTTATGTATCGGGTATCAAGGAATTTGCTGTGAAATCTGAGGCTGTTCAGTACATCTGCATGTGGGTTTTTCTTCTGGGGTTCTGAATTTTTTTTTGGAGGTGGGGGTGTGGAAGAAGGCGTCGGTGGATGGGGACTCAGACCATGGCGAGCCAGGGCGGCGGCGAGGGCCAGCAATCCCAGCTGCAGACCCTGGGGAGGCAGGGTTCCCTCTACAGCCTCACCCTCAACGAAGTCTAGAGCCAGCTGGGGGAGCCCCTGACCAGCATGAACCTGGACGAGCTCCTCAGGAACATCTTGCCGCCGGAGGGAGGCCAGCCGCCGGTGGCGGTCGACCTGGAGAGCCCCGCCTCCGGCTCCGGGCTCCGCCGCCAGGGGAGCGTGGCGGTTGCCAGAGCCCTCAGCAAGAAGACGGTTGACGAAGTGTGGAGGGACATCCAGAGGTGCCAGGATGAGGACGGCGGCGGCGAGGAGCGCAGAGTGGGTCCGGAGAGGGAGCGGACGCTGGGGGAGATGACGCTGGAGGATTTCCTGGTGAGGGCCGGCGTGGTGGTGGAGGGCCCGGAGAAGAAGAGAAATGGCGGCGCGGGTTTAACAGGGGGCGGTGATACCTTGGTGGGGGCGGCGCAGGGGTATTCACAGGGCGTCCACTGGCTGGTGGCCCCTTTGCAGCAGCAGCAACAACTGCTGCCTCAAAACCATCGCCTCACACAGCAGCAGCAGCAGCAGCAAGATCAGCAGAGTGTGATGGCAGTGTATGCACCGAGCCGCCC

>TRINITY_DN25459_c2_g2_i3_3
GCCTCAACGGCGAGGAGAGCCTGGCGATCCTGCTCTCCCGTACGCGCACAATTTCAACAATTTTTTGAGGTCGTTGATAGCTCCACGGCCTCGCTCAACGCCGCCGCCGTTGAGGTCGCTGCAGTGGAACCTGACCAGCACGTTGTCGTCGCCTCCCTCGTCGAGCTGCCTTGGCTCCCTCGGCGGCCCCCTTCATCTCCAGCTAAACCCCTCGCTGGCTCGGCTGGGCCGCCCACCACCTGCTCGACCGAAGACCTAACTCAGAAAACGGCGGGGCACCGGCTCCCAACCGGCCACACCTCGTCCACAATCTTCTTGTTGAGCGTCCTCGTTGGGGCGATGCTCCCATGGCGCTGGAGCTCGGCACCGGAGCTGAACCGAACAGGGTTCTCGAGGTCCACCTCAGAAGGTTGATCAGCCTCGCCGGAGAGCACATTCCTGAGGAGGTTGTCACGGTTCATGTTGTGCAAGGGGCTTGCCGGCCAACTTTTCCAGGCTGAGGCAGCGATCTCGGTAGACTCGGTGTACGACGGCTGGCAGGAAGACGAGAGACAGTTGGAG

>TRINITY_DN23165_c0_g1_i6_2
CCCGCATCGGCAACTTTGACGCCGCTCGCTCCATCAACAAGTGGGTCTGCGCGGTGTGCAACCAGAGGCATTCCGTGCGAAAGGTATTCGCCCGGAGCTGCCTTGCCCGGGAATCTCCGCTGCCAGTGCTGGTGTTGCCTCTTCAGCCGGTCGCCGCCGCCGCCGCCCGGTCGTTGATCGCCGCTGCCGCCTGCTTCCTCCTCGCCGGCCGCTTGCGGAACCTTTTGCTCTGGTTCTTCCGCCGCCTCCGCCGGGCGAGTTCTTTATTCCTCTCATGGTGGCTTCCGTGGGATGTTCAATAGAGCAGGTTGAGGAGTAGCCTTGGGAAGTGGGTGAGGTGAAATTTTTTTATTGAAAGGAAAAAAAGGATTTTGAGGAAGCTGTAATCTGAATGATATGGATCACTTGACAATGATTGCAAGTGGGCTCGGGGATGGATCGGATTTTGAGGTTGATGACGTCAGGTGTGCCAATCTTACTGAGCACGATGTTAGTGATGAAGAAATTGAACCAGAGGAACTGGAAAAGAGGATGTGGAAGGATCGGATCAAGCTGAAGAGGATAAAAGAGCGGAAGAAGCTTGCGGCACAGCAGGCCTTAGAGAGATCTAAACCAAAGCAGATGTCTGACCAGGCACGTAGGAAGAAGATGTCAAGGGCGCAGGATGGGATCCTGAAGTACATGCTTAAGCTGATGGAAGTTTGTAATGCTCGTGGATTTGTCTATGGGATTGTACTTGAGACGGGGAAACCTGTAAGTGGTTCTTCCGATAACATGAGAGCTTGGTGGAAAGAGAAAGTGAAGTTTGATAAGAATGGGCCTGCAGCAATTGCAAAGTATGAAGTTGACAACTTTACTGCTGAGGATGCAAAAACCAATGGTGAAAATTTTCACAGTTTGATGGGCATTCAGGATGCTACTCTGGGTTCCCTTTTATCTTCACTGATGCAACATTGTGACCCACCGCAACGGAAGTTCCCATTGGAGAAGGGCACCCCCCCTCCATGGTGGCCCTCGGGGAATGAAGACTGGTGGATAAATTTGGGATTACCGAAGGGACAAATTCCTCCATATAAAAAGCCTCATGATTTGAAGAAGACATGGAAGGTTGGAGTCCTTACAGGAATTATAAAGCATATGTCTCCCAATATTTCCAAAATTAGACTTCATGTTCGGAAGTCAAAGTGCTTGCAGGATAAAATGAGCGCCAGAGAAAGTTCAATCTGGTCTGGAGTTCTTAGCAGGGAGGAATCTGTTTCACAGAGAAGTGAAAATGGGTTATCTGGCTTAACTGATGTGCAGCATAGTGGTTCAGGGGAAAGAAGGGAGGATGCAACTAGTAGCAATAATGAATATGATGTGGATGATATAGGCGATGGTTTGTGTAACTTGCCATCTAAAGATGGCAGGGATGTGGAGCCATGCCAAGATTCTGTTGCACACTTGAGTTGCCCACCTGAAAATGATGATACAAATGTTCCACATAATGTGCAAGTAGATACACGGCCTAAGAAAAGGTCTCGTGGAAATGCTGTATGCTCTGATCAAGGCACACCTTTTTCTCACAATCAGAAAGTACAAAGTAAATCCCGAAAAGTCATATTGGACATGAATCACACACAACTTCCCCAGCTGGCACAGCAGCAAGTGCACACTGCCCCGGAGGGAGGGTATATACCATCTGCTGCAAGGCCTCAAGAAGAAGGTTTTGATAACCATTGTGTCTCTTCTGAACCTGTTTTCACTGATCATCCAAGTTTCCCTCCTGCACATGGTACTGCACAAAGCATGCATGTTAGTGGCCAACCCCTCTTATATTCATCTTTTGGCAATGCTGGACATGGAGCTGGGGATACATTTGGGTTCTTTTCATCATCTGGAGAAGGTAGAATTTGTCATGATACACACCGATTTCCTGATCCCATGTTCGATCATCATTTAAGGCCTGAGAATAATGGAGCTGATAATGAAAGTCAAGCCTTTGGACAGCCACTTGCCTTAGAGGGGAGTTCAAATCCGCTTACTGGTGATGTTCATCCACTTGTGGAGGATGCTTTCAGCAATGGGCAAGATAAACTTCTTGACAGTCATTATGGCTCACCACTTGGTGGTCTTTCGCTAGATTATGGGTTCACCAATAGCCCATTTGATCTCAGATTTGATAACTTTGAAGATTTTCTACATGATGAAGAAATCATGGAATATTTAGGAACATAGGCAGCAGAAGGTCCCACTGCGGTTTGGAAGTTTTGCAGATTTTTTATACAGTGGAAACCTACTTCGTGGACTGACTTTTACATTTGGGCGAAGGTTTGGGTCCACTCAGTGTATTTGTTTTAGCTATCCTCTGTTCTTGGTTACATGTGAGAGACCAATGGCAAACCTGTGTACCTATTGCCATGAATGGGTCTTCATGAGGCACCAAGCTGTGGAAATTTCTTGTCAAATTTTTTATATTGCTTGCTCTAACCCATTTGTTTCATGAGCAGCAATTGGAAAATTCGTTCTTGATTTAAGCTATAACTTTTATAATTACAAACAGGCTTACAATAAAGGATGACCTGAGAGCATTATCTTCATTTCATAGTCTTTTGG

>TRINITY_DN26234_c0_g1_i2_2
TTGCCCGCCTCCGACTCCGAGACCTCTCCGGTGGGGGGAGGGAGCGGAGGGGCAGTTGCGGCTTGCCTTCGGAGGCGGCTTCTGTTCATGGTTTCTGTTTGCTTGTGATTACTTCTCCCCACCTCTTCCCTTTGAAGTTTGACCTTTTCTGTGGCAGATGCATCTGAACAAAGACTTGTAGGAGGAACTAAACTTCATTTTCTGCATCTGCAACTCGTTCTGTGATATCTGCCATAGAACAATGGGTGGTGTGGTAGTGGATGATGTTTTTCCGAGTTCATTTGAATTTTGGTCTCTTCCACCCTCTGCCAGTCTTGAGAGCCTGCAGTTTGCCTCTATGACTGCTCCTCCAGCATCAGTTGGCGGTGCAGATGTGATGGGACTTAGTGCAGAAAGCTTTAATGACTCAATACCTGACGATGATGACAGTGAAGAGGATATTGATGTCGATGAATTGGAGAGAAGAATGTGGAGAGACAGGTTGAAGCTGAAGCGCCTGAAGGAACAACAACAAAACAAGAGCAAGGAGCAAGGTGATGCAGTGAAGCAGCGCCAGTCCCAAGAGCAGGCACGGAGGAAAAAGATGTCTCGTGCACAGGATGGGATTCTGAAGTACATGCTGAAGATGATGGAGGTTTGCAAGGCCCAGGGATTTGTTTATGGTATAATTCCTGAGAAGGGCAAGCCAGTTAGTGGTGCATCTGATAACCTTCGTGGATGGTGGAAGGAAAAGGTCCGTTTTGACCGGAATGGGCCTGCAGCCATTTCAAAGTATCAGGCTGACAATGCTATCCCTGGGAGTAACACTGAGACTGACACTGATATCTCAACTCCCAGAACCTTGCAGGAACTTCAAGACACTACTTTGGGGTCCCTTCTCTCTGCTCTGATGCAACATTGCGATCCTCCACAGAGGAGGTTCCCATTGGAGAAGGGAATTCCACCCCCATGGTGGCCCACTGGGAATGAAGAGTGGTGGCCCCAGTTAGGCATTCCAAGAGAGCAAGGTCCTCCTCCATACAAGAAGCCTCATGATCTGAAAAAGGCATGGAAAGTCAGTGTTCTTACTGCTGTGATCAAGCACATGTCCCCTGATGTCACTAAAATTCGCAAGCTTGTGAGGCAATCAAAGTGCCTGCAGGACAAGATGACGGCCAAGGAAAACCAGACCTGGTCGGCTGTGATAAGTCAGGAGGAGGCCCTAGCTCGGAAGCTGAATCCTGATGCTTGCCCCCCACCATCTTTAGGTGGCGGCAGCAGTGGTACCTTCTCATTTGGTAGCACAACGACTGAGTACGATGTGGATGGATTTGATGATGTCACCAACATTGGGATGGGGGACAGCAGTATGGATGGCAGTCTGTTCAATATAGGTGCTGTTGCTGGGAAGGAGAAGCTTCTTGGAACACACACAATCAAGGAAGAAATTGGTGTGGATTATATGCAGAAGCGGCCACCTACATCAGACGTAGAGCAAATGGTGAAGCAGAATACGTTTACCTGTGAGAATGTCCAATGCCCTCACCATGACCATCGTTTTGGGTTTCTGGACAAGAACGCCAGAAATAACCATCAGTATGCTTGCCAGTATCGTAGTAATTTCTCTCAGCGCCTTGGTGGCTCAGGCTTCCAGCTGAATGAGAACAAGACCCAGGTTTTCTCCTTGCCTTTTTGCCAGCCAAAATCTTCTCCGTTCTCTAGCAATCATGGGGCCATGGACATATCTGCTCTGGGGATTCCTGCAGATGGTCAGAAGTCAATCAGTGAACTCATGGCATTCTATGATAACAATATCAATCCCAGTAAGCAGGTGGATGGTGGAGCATTGAATGTGGTGGATGATCCTAATCCTCTCCAGCCAAGAATGCAGATGGATAATAACATCTTTGGTCAGGCTCTTGGGATTGAGACAACCAACATGTTTGAAGAATCAAGTGCTTCAATACAGACCAATGCCTGCCTTCGGGATGACATCACCTTCCAACAGGGATTTGAGAACCAACATGGAGATCTGAATGCAGATTTCGGATACACGTCCTTCAATTTCTCCAACATTGAATATGGTGATGGTCTGCATAGGGTGACAGGGGATTCGATGTCAAAGCTGAATGGTTCCAGTTGGGCCTTCTGAATCAACTAAGCAGCTGCCAATCGAACGCTCTGCTGAATTTCTTGGATCATTTCACAGAGGTGGTGGGTTAGCTCATGTACTGTCCAAGTTGCTGCACGTCTGCAGACTGGGGATCAGTTGATCTGCTCTCTGCGTGTAGTTAATAGTATATTTAGGATGGCTTGAAAACTTAGTATCCTGTAATGTGCTCCTCATGTTGCTTTTATAAGTTACTACAAGGGAGTAGTTCCTCAGACTGCTGATCACAGGGATTGGATTCTGTTTTATCCATTGAAGGTGACAGGATTCTAGGCCCACATTTAGGATGAACCCAAAGCTCCTGTACTCGGAACGTTCTACCAGTGGAACTTCTATCTGATGTATACTATCTGTTGTCTACTCTGTTTCTTACTGACTGTGCCTTGCAAGATTTTGATCGCCGCCTCGCCTCGAGATGGATTGTAAAACTCCACGGTCGAGTGTCGACTAATACTTGTTAATTAATTGAGCTCTCTGTCATTGTTCTTCGCCATCTCTGTGTTCTTTATTCTTGTTGCCTCAGATGACACTCGCTTGTTTGCTATTGAATCTACCAGTTTGCTGGTGCATGTGGTGTTCGGAATCCCCAGCCGAGTGAATTCGGTTTCGCTTCAACTAAGTTAGGCTGCTCCATACACTGCAGGCGACAGGTTTTGAGCTGGAAGAACCTGTTGGTTGACACTGGTGTTTCTTCCCTTTGTAAACAATGTATGAGTGCCATCCTCAAAACGATGGTTTGCTGTTAAAGTATG

>TRINITY_DN28746_c0_g1_i8_2
ATTCCATCTCGTCCCCCTTCGCCACACCACTGCCCACCCCGCCGGGGCTCCCCCACCTCCATCTCTCCTCCTTCCCACACCTCCCCTCCTCTCCTGTTTCTATCTCTATCCCCTTCGTCCCGCTTCCTGCTGCTCTTGTTGGCGTCCGTGATCGAGCTCTGCAGGGGGAGCGGTGGTGCTTCCATGGCGTGCTGGAGGTGATGAGACGAAGAACGTGGAAGGGGGGCGCGTCTTTTCTTCCGGTGGTTAATCTGATCCGACAAAGACAGTTGTAGGAGGGAATTTAGCTTGGTTTCCTACATCTGTGAAGTGTTTGCTGATCTCTTGCTTTATAGCCATGGGTAGTCTTCTAGTAGACGATATTGGCTTTGCCGGTTCCTTTGAATTTCTTCCCCTTCCAGGTTCTGCCAATGTGGAGAATCTTCAATTTGCTTCCGTGAATGTTCCAACAACTCTTGGAATGTGTGATGTTGATTTAATGGAATGCCCCACCGAGAACTTTCCTGAGGCCATGCCTGATGAAGAGGACAGTGAGGAGGATATTGATGTAGATGAGCTGGAGAGAAGAATGTGGAGAGACAGATTGAGGCTGAAGCGCCTGAAGGAGCAGCAGCAGAACAAGAGCAAGGAGCAAGGGGATGTTGTGAGGCAGAGCCAGTCACAAGAGCAGGCGAGGAGGAAGAAGATGTCCCGTGCACAGGATGGGATCCTCAAGTACATGCTCAAGATGATGGAAGTTTGTAAGGCACAGGGTTTTGTTTATGGCATAATACCTGAGAAAGGAAAGCCTGTAAGTGGAGCATCTGATAACCTCCGTGCATGGTGGAAGGAAAGGGTTCGCTTTGACCGCAATGGACCTGCAGCAATTTCTAAGTATCAGGCTGACAATTCTATCCCTGGGAGTAACACTGAAATGAATTCTGCTGTCTCAACTCCTCGTACCTTGCAAGAGCTTCAAGATACAACATTGGGATCCCTACTGTCAGCACTGATGCAGCACTGTGATCCACCACAGAGGAGGTTCCCACTGGAGAAGGGTATTCCACCTCCATGGTGGCCCACTGGGAATGAGGAGTGGTGGCCCCAGTTAGGTATTCCAACAGAACAAGGGCCGCCTCCATACAAGAAGCCACATGATCTGAAGAAAGCATGGAAGGTCAGTGTTCTAACTACTGTGATCAAGCACATGTCCCCTGATGTGAACAAGATTCGCAAGCTTGTGAGGCAGTCCAAGTGTCTGCAGGACAAGATGACAGCCAAGGAGAACCAAATCTGGTCAGCTGTGATTAACCAGGAGGAAACCTTGTTTCGGCAGCTCAACCCTGGCACCTGCCATCCCCCAAGTCTGGGTGGTGGTGTCCCTAGTGGAACCTTTTCATTTGGTAGCAGCGCTAGCGACTATGATGTTGATGGGTTTGATGATGCAACAAACATTGGAATGGAGGACGGTAAGGCAATGGATGTGAATTTTTTCAATGTAGGTTCTGGCATGGCAAAAGAGAAGCATTTGGCGCCAGTTCCGATTAAGGAGGAAAGTGGGCTGGAGTTTATACGGAAGAGATCATCTCCATCTGAGGTAGAGTTGATACTGAATCAGAACACTTTTACCTGTGAGAATGTCCAGTGTCCTCACCATGATTTCCAGCTTGGTTTTATGGACAAGAATGCTAGAAACAACCATCAGTATGGTTGTCAATATCAGAATAATTTCTCTCAGGGTCTGCAGACAGGCTTCCGGATGGAGAACAGGTGCCCACTTATATCTCCAAATTCCCAGCCAAAGCCATTGTCTGGCAATAATATCTCTGGCTTGGGAATTCCTGCCGATGGTCAGAAGTCAATCAGTGAACTAATGTCATTCTATGACAACAACATCAATCCCAATAAGGCGCTGAACGGATCTGCGAGCTTAGAAAGCAATCGCAATCCTCACCAACAGAGAATTCAGATGGACGATAACTTCTTTGGGCAGTCTCTTGGCATAGGATCAAACAGTTCAATGCCACCACATGCCTTCCTTAGGGAGGAAGTGCCCACCTTTCAACAAGGGCTTGAGAACCAGCACCTAGATATGAATGCTGACTTCAGATTCAGTTCCTTTAACCTCTCCAACATCGAGTATGGTTATGGGCTCCCCAGGGGTTCTGGAGATTCCATTCCAAAGCTGGACAGCTCAATCTGGTCCTTCTGATCTATCATCACTTGGTGGTGGTGGTGGTGGGTAAGAGTAGTGTCGGAGTCTCTGTAAATTTGCAGGAGACCATCTCGGTTGTTGCATATCTATATACTGCAACTTTAATTTATCCCTGCGTCTAGTTTTATAGGGTCCTGGGATGACATGAAAAGTTCAGTCCTGGGATGTCACTATTAAGTTTTTCTTTGAAAGATGCATTAAGGTCGTCATTGTATCAAACTTTTGGCAACCCGCATGGAAACGTACTGAGAAATCGGTTCACTGGGTTTTATTTAAGTTCAGCTGTTCTTCGTATTCTTAAATTTGTGGCGCCTGTCTTCTTGTTGCACCATTCATGAAATGGTTCCCATTCTAGTTATATGACAAAGTTTGTTTACTTAATCACTGGATCGTTTGCTTGATGCCAGGCTTTCTATTTTTCCATATCTGAGGGATTGTCTTCCTTGTCGGCCATGAACCAGGTAATGTTTGGTATCGGGACAGATTGAGG

>TRINITY_DN17002_c0_g1_i1_3
GTGCAGTCCACCCGGAGGAACCACCCCTCGGCGAGGCTGATGGTGGCGCTCAGGGAGCTCGACCTCGAGGTGGTGTACGCCAGCGTCTCCGTCGTCAACGAGCTCATGCTGCAGCAGGCCACCGTCAAGCTGTCGGCCAGGGCGGCGTACACGAGCGAGCAGCTCACCGCTGCACTGCTCTCTCGGGTTGCCGCCGACCCTTTGCCGCCGGTCAGCCGGTGATCCACCTCCCCTGCTGCTATCTTGGGATGCTGCAATCTCTAGCTGCTCCAGCTTGGGACTGGCCACCTCAAGCTTGGGAAGAGCTTCGCCGAGAGCAGAGTGGGTTTGAAGAGAAGGGGGGCTCAGAAGCCCAGCAACCCCCCACTCCAGTGAGTAGCCATGTTTTGATCAAATCCGGCAATGTCTGTAAACTAGGTAGGTGTATCAGCTTAGCCAGTTAAGTAGGTTACTTGCTCTGTACAATATATATGCATATATCTATACTTGTGTGTAAATATGAGAGAGAGTGTGAGAGAGTAGCTAGATCAGTTATGTGTTATTTTTTTTTGACCAAATGCTAGATCTGTTTAAATTCCATCTGAGATTGCGTCCTATGTATGACTAATTAGATGTTGTTTCAGTGGAAATTTAAGTGTAGAAGTTACTTCTTGTCATGAAAGGTTTGCCCATTCTGTGGAGCT

>TRINITY_DN19736_c0_g1_i4_2
GTCTGCAGGCCCTCATCGAGGGCGCCAGCGAGAGCTGGACCTACGCCATCTTCTGGCAGTCGTCGGTGGATGTGGCCAACGGGGCGTCGCTGCTGGCGTGGGGCGACGGGTACTACAAGGGAGGCGAGGAGGACAAGCGGAAGATGCGGGCGGCCAGCGCGGCGTCGGCCGCGGAGCAGGAGCACAGGAAGAAGGTCTTGCGCGAGCTGAACTCGCTCATCTCCGGCACGCCGGTGTCCGCAGACGACGCCGTCGACGAGGAGGTCACGGACACCGAGTGGTTCTTCCTGGTGTCGATGACGCAGAGCTTCCTCACCGGCGCCGGCCTACCTGGGCATGTTCTTCTCAGTTCGTCGGCGGCGTGGGTCGCTGGGGCTGAGAGGCTAGCCTTGACGGGCTGCGAGCGCGCCCGGCAGGCTCAGGAGTTTGGTATCCAGACGATGGTGTGCGCCCCCGTCGCCGGCGGGGTGGTGGAGTTTGGGTCCACGGAGCTGATCTACCAGAGCGCGCACATCATGAACAAGGTCAAGATGCTGTTCAACTTTGTAGGCGGCCGCGGAACCGGCCTCCAGGCTGCGGCGGCTTCATCGCCGGCCACGGCGCCGTGGATGCCTGAGGGCGAGAACGACCCTTCCGCCCTGTGGATCTCCGATCCATACGCAGTGGAGATCAAGGATTTGCCTCCACCCCCGCTGGACATCTCGTCCGCGTCGAAGCCCCAGCCCCAATTCATCGAGAACCCTAGCAAGGGCAGCCTGACCGGAAACCCTAGATCCGTCCAATTGAACCGCTACGAATCACCACAACAGCAGCAGCAGCAGCAAAATCATCATTCCCATCACCATCTACAGCAGCAGAATCATAGCACTAACCCTCAGATCCAGCAGGGTTTCTTCACCAGGGAGCTCAATTTCTCAGAGTTTGGGTTGAGCAGCACCAATCCGCTCCCGGCGTGCAAGCCGGAGTCCGGCGACATGATGAGCTTCGGTGAGAGCAAGCGGAATCCCCCCGTGGGAGCAGCCGCCCCCGGCGCCGGCAACGGTGCCCTGTTCACGCATCTCTCGAGCTCTCAAGCAGAGGAGAGGAAGAAGAAGCAGAAGCCGGCGGCGGTTGTGGCGTCGTCAAAGGGGGGGAGCCCAGACGAGGGGATGCTGTCCTTCGCCTCCGCCGTCGTGTCCGCGCCCTTCGACGAAAGCATAAGGTCCGGCAGCGCCGACTCGGTCCACTCCGACCTGGAGGCCTCGGTCCGGGAGGCGGAGAGCCGGCCGCCGCCGCCACCGCCGCCGGTGCCGGAGCCGGAGAAGCGGCCGAGGAAGCGGGGGAGGAAGCCGGCAAACGGGAGGGAGGAGCCCCTCAACCACGTGGAGGCGGAGCGGCAGCGGCGGGAGAAGCTGAACCAGCGGTTCTACGCCTTGCGAGCGGTGGTGCCCAACGTGTCCAAGATGGACAAGGCCTCCCTCCTCGCCGACGCCATCTCTTACATCAACGAGCTGACCTCCAAGCTGCAGTCTTCGGAGTCCGACAAGGAGGGGCTGCAGAACCAGGTGGAATCCCTGCTGAAGAAGGAAGCGGCGTCCCCACCTCCGGCCAGGAGCTCCGGTCCCCTGTGCATCTCCCGGGCGTCGACCGCCCCTCCCGCCGACGAGGATGTCAAGAAGGTGCTGACGCGGTCCCCCGGGATGGAGGTGGACGTGAAGCTGATGGGGGGCGAGGCCATGATCCGGGTGCAGTCCACCCGGAGGAACCACCCCTCGGCGAGGCTGATGGTGGCGCTCAGGGAGCTCGACCTCGAGGTGGTGTACGCCAGCGTCTCCGTCGTCAACGAGCTCATGCTGCAGCAGGCCACCGTCAAGCTGTCGGCCAGGGCG

>TRINITY_DN23166_c0_g1_i8_3
CCGTCTTCCACCATCTCCCTTCCACCTCTCTCCAACCCCGCCGCCACCACCAGCTGTCCTCTTCGCCGGCGGTGACCTCGCCGCCGACGCCCCACCTTCTTCCCTCGTCATTTCATGCGACGGCTGCTTCGCTGAGAAGAGCACCCTCCCTCCTTCATCCCGTGATGAACCTGTGGGCCGACGACCACGCCTCCATGATGGAGGCCTTCATGGCCTCCGACCTCCATGCCCAGGGCTTCCCCTGGGCGGCCTCCACCTCCTCCGACCACGCCTCCCGGCCGGCGCTCTCGGCGGTGCCGCATCCGGCACCTGCGGCAGCCGAGTACTTCAACCAGGACACGCTGCAGAACCGGCTGCAGGCGCTGATCGAGGGCGCTAGGGAGAGCTGGACCTACGCCATCTTCTGGCAGTCGTCCGTGGATGTGCCCGGCGGGGCCGCGGTTCTCGTGTGGGGCGACGGGTACTACAAGGGGTGCGAGGAGGACAAGCGGAAGGGGCGGCGGGGGGCCGGCGCGGCCTCCGCGGCGGAGCGGGAGCATCGGAAGAAGGTGCTCCGGGAGCTCAACTCGCTCATCTCGGGGACGCCGGTGTCCGCCGACGAGGCCGTGGATGAGGACGTCACCGACACCGAGTGGTTCTTCCTGGTGTCCATGACGCAGAGCTTCCTCACCGGCGCCGGCCTACCTGGGCATGTTCTTCTCAGTTCGTCGGCGGCGTGGGTCGCTGGGGCCGAGCGGCTGGCCTTGACGGGCTGCGAGCGCGCCCGGCAGGCTCAGGAGTTTGGTATCCAGACGATGGTGTGCGTCCCCGTCGCCGGCGGGGTGGTGGAGTATGGATCCACGGAGCTGATCTACCAGAGCGCGCACATCATGAACAAGGTCAAGATGCTGTTCAACTTCGTAGGCGGCCGCGGTACCGGCCTCCAGACGGCGGCGGCTTCATCGCCGGCCACGGCGCCGTGGATGCCCGAGGGCGAGAACGACCCTTCCGCCCTGTGGATCTCCGATCCATACGCAGTGGAGATCAAGGATTTGCCTCCACCCCCGCCGGACATCTCGTCCGCGTCGAAGCCCCAGCCCCAATTCATCGAGAACCCTAGCAAGGGCAGCCTGACCGGAAACCCTAGCTCCGTCCAATTGAACCGCTACGAATCACCACAACAGCAGCAGCAGCAGCAAAATCATCATTCCCATCACCATCTACAGCAGCAGAATCATAGCACTAACCCTCAGATCCAGCAGGGTTTCTTCACCAGGGAGCTCAATTTCTCAGAGTTTGGGTTGAGCAGCACCAATCCGCTCCCGGCGTGCAAGCCGGAGTCCGGCGACATGATGAGCTTCGGTGAGAGCAAGCGGAATCCCCCCGTGGGAGCAGCCGCCCCCGGCGCCGGCAACGGTGCCCTGTTCACGCATCTCTCGAGCTCTCAAGCAGAGGAGAGGAAGAAGAAGCAGAAGCCGGCGGCGGTTGTGGCGTCGTCAAAGGGGGGGAGCCCAGACGAAGGGATGCTGTCCTTCGCCTCCGCCGTCGTATCCGCGCCCTTCGACGAAAGCATAAGGTCCGGCAGCGCCGACTCGGTCCACTCCGACCTGGAGGCCTCGGTCCGGGAGGCGGAGAGCCGGCCGCCGCCGCCGCCCCCGCCGGTGCCGGAGCCGGAGAAGCGGCCGAGGAAGCGGGGGAGGAAGCCGGCAAACGGGAGGGAGGAGCCCCTCAACCACGTGGAGGCGGAGCGGCAGCGGCGGGAGA

>TRINITY_DN31398_c1_g2_i1_4
CTGCCGCTCCACCTCGCAGGTCTCTTACACGCCTCTCTACTTCCTCCCCACCCCGCCGCCGCCGCCGCCGCCGCCGCTGGCGGCCGCCTTTGTCTCTTCCCTCTGTGCATGCGAAGAAGGCGGCGGAGATGAACCTGTGGACCGACGATAACGCCTCCATGATGGAGGCCTTCATGGCCTCCTCCGACCTCCAGATCAGCTGCTTCCCGTGGGCGGCGGCTCCCACCGTGGGCTCGTCCTCCTCCGACCACCCCAGCGGCAAGTCCCTGGCCGCGGCCCCGCCGGTGGCGCAGATGGCGGTGCCGTCGTCGTCCTCCGCCGCGTCGGGGTCCTCCTCCGCCTACTTCAACCAGGAGACGCTGCAGCACCGTCTGCAGGCCCTCATCGAGGGCGCCAGCGAGAGCTGGACCTACGCCATCTTCTGGCAGTCGTCGGTGGATGTGGCCAACGGGGCGTCGCTGCTGGCGTGGGGCGACGGGTACTACAAGGGAGGCGAGGAGGACAAGCGGAAGATGCGGGCGGCCAGCGCGGCGTCGGCCGCGGAGCAGGAGCACAGGAAGAAGGTCTTGCGCGAGCTGAACTCGCTCATCTCCGGCACGCCGGTGTCCGCAGACGACGCCGTCGACGAGGAGGTCACGGACACCGAGTGGTTCTTCCTGGTGTCGATGACGCAGAGCTTTCTCAATGGGGCCGGCCTGCCGGGGCATGTCTTCTTCACGGCTTCGGCGGCGTGGATCGCGGGGGGCGACCGGCTGGCGGCCGCGTCGTGCGACCGCGCCCGGCAGGCCCAGCTCTTCGGCATCCAGACCATGGTGTGCGTCCCCGTCGGTGGCGGCGTGGTGGAGTTTGGCTCCACCGGCCGGATCTACCAGAATTCTGAGATCATGAGCAAGGTGAGGGTGCTTTTTAACTTCGGATCCAGTGGCGGCGTTGGCGTCGATACGGTGGTGGGCGGCGGCGCCTCCCCTGCCGCGACGGCGTCGTGGATGCCCGGGCCGTCCACGGTCACTACCGACCAGGGCGAGAACGACCCCTCCGCCCTCTGGATCTCAGATCCATCAGTGATCGAGATCAAGGATTCCCCTCTCGCCGTGCCGGAGATCTCTTCCATGCCAAAGCCCCATGTCCAATTCGAGAACCCTAGCAAGAGCAGCCATATAGCAAGCTCCAGTTCCGTCCAATTGAACCTCTACGATCCACCATTTCCTGATCAACAGCATAACCAGCAGCAGAAGCATCATTCTCAGCATCATCCACAGCAGAATCAGCAGCAGAATCCCGCCAACTCACAGAGTCAGCGTGGCTTCTTCAATACCAGGGAGCTGAACTTCTCTGATTTCGGGCTGAACAGCTCGATTCCTCCTCAGACTTGCAAGCTAGAGTCGGGCGAGATCCTAAACTTCGCGGAGAACAAGAGGAATTCCGGTGTCGGCACAGCTCAAGGTGCAGGCGGCGGACTGGTCGCCCACAATTCCGCCTCCCAAGTCGACGACAAGAAAAAGAAGTCCGTGATGGCGACCTCCAGAGGGAGCAATAACGACGAAGGGATGCTGTCCTTCTCCTCCGCCGTCGTCCGCCCCTCGTGCGACGAGACGATGAAGTCGGAGGGCGGCATCGCCGGCCTCGCCGACTCGGACCACTCCGACCTGGAGGCGTCTATCCGGGAGGTGGAGAGCCGGCCGACGCCGCCGGAACCGGAGAAGCGCCCGAGGAAGCGAGGGAGGAAGCCGGCCAACGGGAGGGAGGAGCCCCTCAACCACGTGGAGGCCGAGCGGCAGCGGCGGGAGAAGCTAAACCAGCGGTTCTACGCCCTCCGCGCGGTGGTGCCCAACGTGTCCAAGATGGACAAAGCCTCCCTCCTCGGCGACGCCGTCTCCTACATCAACGATCTCCGCTCCAAGCTGCAGGCAGTCGAGTCAGACAAGGAAGCGCTGCAGACCCAGGTGGAGTCCCTGAAGAAGACGGCAAAGGAGGCCCCGTGCAGGGCCGGTCCCGCTCCACCGGCAGCTGCGGACCAGGAAACGAAGCTGCTTCCCAACGGGGCACCGCCGCGGCTTGCCGGTGTGGAAGTGGATGTGAAGATAATGGGGAAGGAGGCCATGATCCGGGTGCAGTCCAACCGGAAGAACCACCCGGCGGCGAGGCTGATGCTAGCCCTCAAGGAGCTGGACCTCGACGTGGTGTACGCCAGCGTCTCCGTCGTCAACGATCTCATGATCCAGCAAGCCACCATCAGCATGTCCAGTAGGGCCTTCACAACCGAGCAGCTCAGCGCAGCGCTCTACGCGCGGATCGCCGGTCCTCTTGGCCCCACCCGGCAACCGACGCAGCTCTGCCGGGA

>TRINITY_DN31644_c2_g1_i3_5
GGGAGAAGCTAAACCAGCGGTTCTACGCCCTCCGCGCGGTGGTGCCCAACGTGTCCAAGATGGACAAAGCCTCCCTCCTCGGCGACGCCGTCTCCTACATCAACGATCTCCGCTCCAAGCTGCAGGCAGTGGAGTCAGACAAGGACGCGCTGCAGACCCAGGTGGAGTCCCTGAGGAAGACAGCAAAGGAGGCCCCGTGCAGGGCCGCCGGTCCCGCTCCACCGGCAGCTGCGGACCAGGAAACGAAGCTGCTTCCCAACGGGGCACCGCCGCGGCTTGCCGGTGTGGAAGTGGATGTGAAGATAATGGGGAAGGAGGCCATGATCCGGGTGCAGTCCAACCGGAAGAACCACCCGGCGGCGAGGCTGATGCTGGCCCTCAAGGAGCTGGACCTCGACGTGGTGTACGCCAGCGTCTCCGTCGTCAACGAGCTCATGATCCAGCAAGCCACCATCAGCATGTCCAGTAGGGCCTGCACAACCGAGCAGCTCAGCGCCGCGCTCTACGCGCGGATCGCCGGCCCTCTTGGCCCCACCCGGCAGCCGACGCAGCTCTGCCGGGAGTAGCTCGCCGTGGGTCAACATGCTACACTCGGTTGTAACATATTGTTGCATAAAAGAGCGGATTTGGGCGTAGCGTCTTGCTGTTGCAGCTTGGGGACGGCAAGAGCTCGTCGTCTCAAGCTACTGAAGCAGAGGTCGATATGCCAGTGCTGGGACTCCATTCCTGTCCACCGAAGGTAGATTCTTCGGAAATCAGGTGCGGCAGCTCCTCGATCACAGTACTGTGTATTATTAGCTCTACCTGAATGTGTAAATATAGATGCCCCTTGTGGGTGAGGGGGAGATAGAGAGAGATTAGGGGATTAGTTTCTCCGTGATCAACCTGTGTAGGCAGGAATTGCAGTTTCTGCAAATCAGAAACTCATCTTACTGGGGAAGAGGAGAGAGAGAGAGAGAGGATTAGTTTGGAATTGCAGTTTCTGCAAACCAAAAGCTCATCTTGTCTGGAAGAATCCTCATTTTCTGTACCTGTTCTGTGTAGATCTGTTTCAATAACTCAAGTAAATTTTTCTCTGTGCTATATAATATGAG

>TRINITY_DN11736_c0_g1_i3_3
AACAAGAGGCCTCGCCCGGCGGTGGATGCCGCCTCAGTTCTTGATGTTGGTCAACACTACTGGTATCCAGGGGCCACTCAGTCACATCATGATCTTAACAATACAGGTAACATGAGTAGTACTAATGAACCTTACATCTCGGAAACCCAGATCATCTGGCCTCCGGTTTCAACAGAGATCAAGGGGAGGACAGCCATGGCCGCCGCGGCGGCGCCCCCGGATGGGTGGGGGCGTCATCAAAACCACCCCATCAAGAGCCCCCCCTCGCACCCTGTTGCGGATGTTTCCCTCAAGCTCTTCCAGGACGTTAATGGCGGAAACCAGACTGTTGTCCCCCCATGTCCTCCTCTTCCGCCGCTGTCTGCTGCTTTCGTGGCCGAGGACGCGGCGGAGAACGGCGCGGAGAGGTGGAAGAAGCCCGAGATGGGTGGTGGGTACCGGTTGTTCGGCATTGAGCTGGTGAGCGGCGCCACCACCGCCGTGGCTCCTACGGAGAAGGGAACACCGGCGGCCACGGCCAGTACTGTGTCCAGTGCCACCACTTCTGAGGAGCATGCCCATGGAGACTCCGACCAGCACTCCTGGCTCTCGAAAACTTCCAAGCAGCAGAAGCCAACGCTGCAAAAGGACGCTCAGATCAGGCAGAGTTGTTCCACCACTAGAAGCCGCACTAAGGTTCACATGCAAGGTGTTGCTGTTGGGCGGGCT

>TRINITY_DN16776_c0_g1_i2_3
ACCAAGTTTTTCATCGAACGGGGAGTCCTGAAGTGGTTAACAGCATCAAACCCATGGAGAGGGCGTCCTCATGCTGCTTATAGAATCGGCCATGTATATGCTCTTGATGTAATAAGCAGAGTCGCGAGCGTCGGCGGCGGCTGCTACCGTTTCTGGTTTCTCTGCATGGTGGACTGAGCTCTGTAGACAGGGAGAGTTGCTGAGAAATGTCGAGTGCTGGCGTGCACTGCACATGTTTCTGACGAGCATCGCCATTCTTCTCGCTGCATGCCAGGAGAGATGTCTTCTGTTCAAGAGAAGGTCCGGACGGGGGACGCCAACAATGCGAGGACTCTAATTGAGGAGATGAAGCTCCTGGGAGAGTTTAAGGGCCAGTCAGGAGCAAGAAAGGTGATAAATTCGGAGCTTTGGCATGCTTGTGCCGGTCCACTTGTCTCTCTGCCTCAGCCTGGTAGCCTTGTTTATTATTTCCCTCAAGGTCACAGTGAACAGGTTACAGCTTCAACAAGAAGGACTGCTAATTCCCATATCCCCAACTATCCTAGTCTTCCATCTCAAATGATGTGTCAGGTTCACAGTGTGACACTGCATGCAGACAAGGACACTGATGAAATATATGCCCAGATGACACTTCAGCCTGTGAACTCTGAAAACGATGTTTTCCCTATTCCAGACTTTGGGCTGACGAAAAGCAAACAGCCCAGTGAGTTCTTCTGTAAGACGCTAACAGCGAGTGACACCAGCACACATGGTGGATTCTCTGTTCCTCGCAGGGCTGCAGAGAAGTTATTTCCCCAGTTGGATTATTCCATGCAACCTCCAAATCAAGAACTTATTGTTCGAGATTTGCACGATAATTCGTGGACATTTCGCCACATTTATCGAGGACAGCCAAAGAGACACCTTCTGACATCTGGTTGGAGTGTCTTTGTGGGATCAAAACGGCTTAAAGCTGGCGACTCTGTTCTCTTCATTAGGGATGAGAAGTCACAGCTTCTTTTGGGTATAAGGCGTGCAAATCGTCAACAAACAGTGTTACCATCTGTGCTGTCTGCTGATAGTATGCATCTTGGAGTCCTGGCTGCAGCTGCTCATGCTGCTGCAAATCGCAGCCCCTTTACTGTTTATTACAACCCAAGGGCATGCCCATCTGAGTTTGTTGTTCCTTTGGCCAAGTACCATAAGGCTGCGTATGGTACCCAAATATCAGTTGGAACAAGGTTTGGGATGATGTTTGAAACAGAAGATTCAGGCAAACGCAGATATATGGGAACAGTTGTGGGAGTCAGTGACTGTGATCCATTGAGATGGCCAAACTCCAAGTGGCGGAATCTACAGGTGGAATGGGATGAACATAGTTGTGTTGAGAGGCCAGATAGAGTTAGCTTGTGGGAAATTGAAACCCCTGAAAGCCTTTTTGTTTTCCCTGCACTGGCCTCGAATCTCAAACGGCATTTTAGTACTGGAATTATTGGTTGTGAAGGTGCGCAAACATCTATGATAAAAAGACATGCATTGCAATCCCCAGAAAATGTAAATTTGAACTTTACATATCCCATACCCCCGAGCCTTCAGTCAGAGCAATTGATAGAGCCACTAATTAAGGCTATGAATCCTGAAATTCAGGTCGAATGTAGTAAGTCACTATATGCCAATACATTACAAAATTTCAGCAAACATGAGTTGCTGCTGTCGTCTGCCACTTTACCCTGCCAGATGAGAGGCCAGGTACAACAGGATGAAGCAGCTGATCAGGCAGGAATATGGCAGAATTGTCAGTTCCCTTTGCTACAGCAACTAATGCTCCCTTTACAAGGAATGATGGGACAAATGCATAACTCTCAACTTTCATCAGAACAGAAACAGTTGGTTAATTTGGTTTTATCATCATCAAGGTGTCTTTCACAAATATCACAAACACATCCAACAGAAAAGCAAACCATTAGTGAGCCTATGCATGAGCATGAATGTCAAGATCAAAATGAGCAGAGCAAGCACAAAGATGACATTATCATGAGTGAACATGAACGTGTTGCATTACAGGAAGAAGTTCATGTAAAATCTCAGGCAGTCGCTGATCAACTGGCTACAGAGTCTCATAGGCAGTTGCCGCCATCAGCTCATGGTTTACAGACTGAACTACCAACTGCGCTTTCAAGAAAGCATTTTGAAGAAAATTTAGGGTGTCCTAGAAATACCGAGAATACTTCAACTCAAATGTCTCTAAACAAGGCAACAATCCAGAGACTGAGAGGGGATCTCCAACCTGTGGAGCAGCAACAGCAGCTTGTATCAAGCAGTAGGCAGTCAAATTTAGAAGCAACATCTGATGTCATCATCAGCAATAATCATTCAGGTCACATATCATCTCCATGTCCAGAAAATGCTTGGTGGCCATTGGATGGAATCTGCCAACAACCATCTACTTCTGGTTTCAGTCCAATTGAGCTTCCATCCTACTGTGCCAAGCAGCCATCCCTTTTGCTTTGCCCTTCTGATAACACTACCACCAGCTCAGCAGGTATATCCAATGTCCTTGACCCAGAAGGATGTTTGGCCACTGAGGGGGCTTATTTCTCATTAGTTTCAGAGACTACTGAGGTTGGAATTCTGCAGTCCATGGGTCATGGACTTCTAACAACTAATCATGCAATGCATCAGGACATTACTGGAATGCAGAGTATCTCTAATTCATATGGATTGGCAGGTTTCTCAGATGAGAACAGTGAAAGTGTGTTTTACGGAAATCTGCACTGTGAAATTGGTGGAGGCATGATTGTCGATCCTTTTTCTACATGTACGGATGGGTTTGGTGCTGCGAAGCTTTCTGGTTTATTTGTACCTCCAGAAATTCTTCAAAGCAACTGCAATTCATGTCAGGATGTCCAGTCTCAGGTTACTTCCACGAGCTTGGCAGATTGCCAGGCCATTTCCTTTCAAGAATTTCCTGACTGCTCGGGAGGTACATCATCAAGCAATGTAGAGGTTGATGATAATTACTATTTGAATATAGGCTCAAGGAAACAAGTCTCCCCACCTTTGCGGACCTATACTAAGGTACAGAAGGTAGGATCAGTAGGAAGGTCTATTGATGTTACACGCTTCAGAAACTATGATGAGCTGAAGTCTGCCATTGCCTGCATGTTTGGACTTGAGGGGCAGCTTGATGATCCGAGAGGATCAGGGTGGAAGCTCGTATATGTGGATTATGAAAATGATGTTCTTCTTGTTGGTGATGATCCATGGGAGGAATTCATCACTTGTGTCAAGTGCGTTAGAATTTTATCGCCTTCTGAAGTGCAACAGATGAGCAAGGAGGGACTACAGCATATGAGCAGCGCTATCATGAACCAATTTGATGAGAGTTCCTTGCAAGAAAGTGGCCAGCACTGTGAGTCATAGGCATTGCGAAGCGAAGTGGCGTATGAACTCGATAGGTCTCGATGAGCACTCGTTTTTCTCTCGTCAGTATGGATGAGAATTGGACTTCTTGTTGGTATGCTAGCTGTCCACAAGTCAGCTGCATTAGAAGCTTGTGCTTGCTAAGATTGAAGCGCCTCTTATCTGGTCTCATGTAAGTACTAGTCGCACAGCTGGTTTATCTGTCATTTAGGAGATGATGCTTGTAAGCCCTCTGTTTCTACTTGTGTTATTAGATTAATTTTGTAGACAATTTGTAAATTGTAGGGGACAAAGCATGATAGCAATTTGCATAATTTACTGTTATTT

>TRINITY_DN29325_c0_g2_i4_1
GGGGTGATAGCAACAGCTGCAGCAGCAGCAGCAGCAGCAGCAGCGAGGCAGGGCTACAGGCGCAGCTGCAGCAGCAGCTGCTGCGGTACGGAAGGGTGTATGGCATGGCTAGCCCGACTCTTTTGACCCCATCACAACCACTCCGCCCCGTCCCCTTTCGCCAAATCTAAGCAGCTGAGGGAGAAGCCAGAGTGTGTGAGAGAGAGAGAGTCCCCCTCAACCCCCCACCGAGAACTGGACTGCCATGGCGGAGAGTGAGGCGCATTGCCCTTGCTCCTTGAGGATGTCCCAATGCCTCTCTTCCTCTCCCTCTTCTTCCCTCTACCACCGCCCCAAACCCTGAACCGGGTTGAGACGCCTGACCCGACAGCGCCAGAAGGGAGTGGCTGCTAAAAAGTCCTCTAATTTCGTCCCATCTCCGTCTATTTCTTCCCTCCCTTATTTCTCTCCTGTACATGTGAACGATGATGGGGATCGACCTCAACATGGTCGACGAAGACGATGGCTCGGCTGAGAGCCTCGCGGCGCCGCAGCCGGCTCACCAACCGCCGCCGCCCTCGTCCTCCCCTTCCTCCGTCTGCCTCGAGCTGTGGCACGCCTGCGCGGGGCCGCTGATATCCCTGCCGCGGAAGGGGAGCGTGGTGGTCTACTTGCCGCAGGGGCATTTGGAGCAGATGGCGGGGGACTGCGGCGGCGGTTCTGCCACTGCAAATGGAGTGGCCGTCGGAGGGGGGCCGGCCAGCTATGATGTGCCTCCCCACGTCTTCTGCCGGGTCATCGACGTCAAGCTCCATGCGGAGGCGGCCACGGATGAAGTGTATGCGCAACTTTCTCTCGCCCCCGAAAGTGAGGAGTTTGAAAAGCAGCTGCAAGGAGGTGAGGTTGAGGAAGAGGGGGACTCCGAAGAGGTCAATGGCTCGACCAGGTCATCACTTACACGCCATATGTTCTGCAAGACGCTCACTGCCTCTGATACAAGCACACATGGAGGCTTCTCTGTCCCACGAAGAGCTGCTGAAGATTGTTTCCCTCCACTGGATTACAAGCAACAGAGGCCTTCTCAAGAGCTCGTAGCAAAAGATCTGCATGGGACAGAGTGGAGATTTCGGCATATCTACAGGGGTATGTATGAAATCATTCCCTATGCATTGTCAAATATCACTGCTTCTGTCTGCTTATTCATCCAGTTACTGCTTTTACACATTGCTCAAACTTTTGTTTTCAGATGACCGATTGTTTATATTGGAAAAAGGATGGCCTTAGAGTTATTGGTACTTCTGTCATATATATGCTTAGTAGTCTAAATAACTTGCTTTGATTTCCTGTATTCATGTGTTACAGTTGTTTTTAGGTGTCATGCAGATATTTCTCCGAACTAACTGCAGGAAGAAAAAGTTCATCACTAGCAAACTGTGGGAAGGCAGTTGGGGTGGGGTAACCATGTCTGTTTATTATACTAATGACTGTTAGTGGCCTTTTACAGGTCAACCACGTAGGCATTTGCTCACAACAGGGTGGAGTGCATTTGTCAATAAGAAGAAGCTTATCTCCGGGGATGCCGTGCTTTTTCTTAGAGGTGATGGTGGAGAATTAAGGCTTGGAATACGAAGGGCAGCTCCAATCAAAAGCAACATTCCATATTCTGTCATTTCTGGTCAAAGTTCAGGTTCCGGAATGTTTTCTGCTGTGGTTAATGCGATATCGTCAAAATCTGTTTTCCATGTTTACTATAATCCAAGGACAAGGCCATCAGATTTCATAATTCCTTATGGGAGGTTCTTAAAAAGCCTGAATAATTCATTCTCAATTGGAATGAGGTTCAAGATGCATTTTGAAGGTGAAGATGCTGCAGAACGCAGGTACACAGGATTGATAACAGGTGTTGGTGATGTGGACCCCGTCAGATGGCCTAGTTCAAAGTGGAAGTGCCTGATGGTGAGGTGGGATGATGACATTGAGACAAATCGGCTGACTAGGGTGTCTCCATGGGACATAGAGCAGTCTGGTTCAGTTGCAGGTTCCAATTGCTTGTCAGCACCTGCTTCAAAAAGAAGCAAGATATGCCTTACTTCAGTGAATCCGAATTTCCAAGTTCCTCGTGAAAGTGGGTGTTCGGACATTGGGGAATCAGCAAGATTCCATAAGGTCTTGCAAGGTCAAGAAGTTTTGGGGTTTAGACATCCTTATGATGGGCTCAATGCACCAAATAGTAATCAATTTTCTGAAGTAAGGAGGTGCATTCCTGATGCTAGCCGCCATATATTGGCTGCAACCAGGAACAATGTTGGATTTCCTGTAGGAAATTCTGACATTTCCTACAAAGGCATGGGCTTTGGGGAATCTGGTAGGTTCCATAAGGTCTTGCAAGGTCAAGAAATTTTTCCTTTGAAGCCACCATTCATGAGAGGCCACGGTGATATCCAGGCTGAGGAAAATGGTGGTTTCAGGTCCTTTGAGAGGCTGTATGTCTCAAATACTGTGAACAAATGGCCTCAGTTACATGGATACAGTTCTTTGGTGCATCAGCAGGCTAAACCTCCAATGCAGGTCTCTTCACCATCTTCTGTGCTCATGTTTCAGCACGCCACAACTCGCCTCCCATGTCAGCAATCTGTCTATGGAGCCAACTGTGGTGGCACAAGTGACTGCCGTGATGGATCGAGTGGGAGGCATTTGACAATGCCTTATGCCGGTCATCATTTGAGTAAAGATCTCGGAGGAGCATATACATCAGATTTTTCTATAATGCAAAAAGAGGTTGCAACACATCAGTATCATGCTCTGACATCTCCTAAAGCAGCAGATCAGAAAGAGGGGCAGGATTCCCCTTCTAGCAGTGGGAGCAGTTGCAGACTCTTTGGTTTCTCCTTGAACGAGTCCAATCAACAAAGAAATGGAGATGGCAACATATCATCTCCCCAGTCACCAAGGGAGATTATGATGGAATCAACTCCACCCTCAGCGACCCAAAGGGTTGCGAAGGCAATGGGGCAGAGTTGCACCAAGGTAAGTGCTCTCTATGATCTGTGCGCTACCCCATTGTGCAGATCCCCTTGCTTTTTCACTTTATTATGAGATACAGGCTGCAGGTCCACAGGCATGGAATTGTGATCTGAGGGGGAGGAATCTCGATCCTAAGACTTGGTGAATATGATGATGTTGTATGCAGGCAGGCTTCAACATCTCCTCCTCGGTATGGAATGAATGGGACCTCTGATTATTCCAGATGAAGTATGATGATGTTGCCTGCAACGAATGAAAAATGGGAATCTCTTCCACAGATCCAAGGCTGTCTCCAAAATTTTGATTTTGCACCCATGGGGTCACACCTGTCAACAAACACGGTTGGTGATGTTACCCAAGAGTATGCATGCAGTGGGTGGAGGCAAGTTTGTTTCGGTTTTTATGATATGGGTGGTCTACCATGGTTATGCTTGATAAAGCAAAACATTCGGCTTTATATACATATTCTGTCTTGACTCTGTTTCCCTATCTTATCCTATCCTAGTAACACTTGACAGTGCTTGTTCATCCATTACTTGTGCTTAACTAGAACTGACACTGTGCATGCGACTGTATTCCCAAATTAGTTTGTACCTGCTTATGAG

>TRINITY_DN30046_c0_g2_i1_1
TCTGCCGGTTCCATCGCTCTCGTCCCCTCGCCGATCGCTTCGTCGACGTCCTCCTCTACCCTCCGCGACGGCGCTGCTTCCCCCTCCAAGATGGACCACCTCGATCATCTCCCCTCTGCCCTCCAGGTGACAGCACAGTGCAGCCGGCGCAGCTCCCTCCTCCACGGGTTACAACAGTGAGATGAGGGACAACTTTAACACGCACAAGTCCTTTTGCGACGCATTGACGGAGGAGAGTGTGTGGGTGATCACACCGAAGATCACGGCGGTGACCTGCAATGGTGTTCTCCAGCAAGACGGCGGTGCAGATGAGAAGAGCAATGTGAATGGAATCAGGATTACAGACTCTAGAGTAAAGCATCTGATGAAGAGCTGCAAAGGGCCCTCCGACGATGCATTGTACAGAGAACTCTGGCGTGCATGTGATGGACCTTTGGTTAGTCTGACCCAAGAAGGCGAGCAAGTTTATTACTTCCCCC

>TRINITY_DN24811_c0_g2_i4_3
TTAGGATCGATGAGCTTTATAAATCCAGAAACTTGGGGATCTTGGAACTAATTTGACCTGTTGTTATCGGTGAAATCCCAGATCTCTGATCTGTATCATAAAAACATGGTTTTATAGAAGAACGAGTTGCTTTCAGTGAAATTTGCTTTCCACAGTTCATTTGGTCTGCCAAATTGGTTCAAGCCATCATAGAATATGTACCTTTCCTTCTCCCTGTTCCTGAAGGTTCTCCTCAGAAACACAGGATCTTAAATAGTGTCAGATCATCGAAAAAATGCAGAATAATGAAAACTAATGCCTCAATCAAACATCCGAAACTTCACAAGCTCATCCCAATTTCCTTTGGTTTCCTTCAGATCTCATCCCCAAATTCCTACCATCTTTGAAGAAAAAAACTGACGTTTCAATGGCTCAATGGCAGATGTCTGGAAGAAGGGGCAGAAGAGTTCTTCCTGAAGCCGGTGAAGTTATCAGACATGGCGAAGCTTGTACCTCACATCATGAAAGGGAAATCCAGAGAGCAGCAGACACAGCAGGCAGAGCAGCCACAACCAATGACACAAGAACCACTGCAGCACCAGGAAAACAACTACAGCGGCCAAACCACAGCTACCATAACACAAAATAACAGCAGTAATGGCAGCAACAGTAACAAAAGGAAGAGCGTGGAAGAGGGGCTTCTGCCTGAGAGTAGCCTCTCCCCAGAGAGGACCAGACCAAGATACACAGTAGCAGCTTGACAGCAGTCGAATAAGATTGTTGGATGGGAAACTCCTTCGTACACTTTTTCTTCTTATTTTGCCTTTCTTTCCCTTTATTTTCTTTGCTCTTTGGGTTTGTTCTGGATCTAGGGGTTTCCTTCCTTAATCGGATAAGATTGTTGGATAAAGATCGAAACCTTCAAGCATACAGTACAGCCGTTCGATTACAATTTTGTTGTGTTCTCAGGCTAAAATGGTTTTGAGAAGAAAGTTTGTACATATTTTTTTTTGATGGGCATCTGATGGCTTGCCACCCAAAACTGTAGAGTGGAGGCTGTGGTCCTGTGGAGCTTGTGGAAGATTTTACTCAAATTAGG

>TRINITY_DN27108_c0_g1_i4_5
TTCTACCTGATTCTTCAAGCTTATTCTTGTGTGAATTGTGGAGGAAGCGCCTTCATGGCGGTCACAGCCGAGGCACAATTCCATGTTCTAGCTGTTGATGACAGCCTCATTGACAGGAAGCTGATCGAGAGGCTCCTCAAGACCTCTTCTTACCAAGTTACCGCTGTGGATTCCGGGAGCAAGGCCTTGGAGTTGCTGGGTCTGCGTGACCACCGCTCTGATCACTCTCCCTCTGGCTCTGCTTCTAACCAGGAGATTGCGGTGAACTTGGTGATCACCGATTACTGCATGCCCGGCATGACTGGCTATGATCTGCTCAAGAAAATTAAGGAGACATCACCGCTGAAGGACATCCCTGTCGTGATCATGTCTTCTGAGAATGTGCCCTCCAGAATCAGCAGATGTCTGGAAGAAGGGGCAGAAGAGTTCTTCCTGAAGCCGGTGAAGTTATCAGACATGGCAAAGCTTGTACCTCACATCATGAAAGGGAAATCCAGAGAGCAGCAGACACAGCAGGCAGAGCAGCCACAGCCAGTGACACAAGAACCCCTACAGCACCAGGAAAACAACTACAGCGGCCAAACCACAGCTACCACAACACAAAATAACAGCAGTAATGGCAGCAACAGCAACAAAAGGAAGAGCATGGAAGAGGGGCTTCTGCCTGAGAGTAGCCTCTCCCCAGAGAGGACCAGACCAAGATACACAGTAGCAGCTTGACAGCAGTCGAATAAGATTGTTGGATGGGAAACTCCTTCGTACACTATTTCTTCTTATTTTGCCTTTCTTTCCCTTTATTTTCTTTGCTCTTTGGGTTTGTTCTGGATCTAGGGGTTTCCTTCCTTAATCGGATAAGATTGTTGGATAAAGATCGAAACCTTCAAGCATACAGTACAGCCGTTCGATTACAATTTTGTTGTGTTCTCAGGCTAAAATGGTTTTGAGAAGAAAGTTTGTACATATTTTTTTTTGATGGGCATCTGATGGCTTGCCACCCAAAACTGTAGAGTGGAGGCTGTGGAGCTTATGGAAGATTTTACTTGAATTAGGCTACTCTGCTCATTTGACTGGTTCCTCCAGT

>TRINITY_DN27108_c0_g3_i3_5
TTCCCCCTCATCACCTGCTCCTCCACCCCCCATCCTTGAGTAGAAGGGAGCGGTGGAAAGCCTGAGAAAAAGAGAAGCGGGGGAAAAATCCCAACCAAACACCAATTCCCATCCATATTCCTACTTCTACCTGATTCTTCAAGCGTATTCTTGTGTGAATTGTGGAGGAAGCAGCTTCATGGCGGTCACAGCCGAGGCACAATTCCATGTTCTTGCTGTTGATGACAGCCTCATTGACAGGAAGCTGATAGAGAGGCTCCTCAAGACCTCATCTTACCAAGTTACCGCTGTGGATTCTGGGAGCAAGGCCTTGGAGTTGCTGGGTCTGCGTGACCACCGCTCCGACCACTCTCCCTCTGGCTCTGCTTCGAACCAGGAGATTGCGGTGAACTTGGTGATCACCGATTACTGCATGCCCGGCATGACTGGCTATGATCTGCTCAAGAAAATTAAGGTATTTACTGGGAGTTGTAGAGAAATATTGTTGAGATTTCTATGCCAACTCCTTTTTTGCTTCTCTCTCGGGAGAATTAAGGGATTCGTTTTCCGTTGTTCAATCAGGAGACATCACCGCTGAAGGACATCCCTGTCGTGATCATGTCTTCTGAGAATGTGCCCTCCAGAATCAGCAGGTGAGCAACAACATAAAGAAAAAA

>TRINITY_DN28010_c0_g4_i1_4
CGCCAGCAGCAGCAGGAGAGGAGAGTAGTCGAGAAGCCCTTGGCCTTGCTCCTTCTCAGATTTTTGTTTGTCGTTCCTGTGGTTTCAAATTCTGATGTTTGTGTGTGGGTGATCTCTAATTAATTCCAGTCACCACTGTGGATTCGGGAAGCAAGGCCTTGGAATTCCTGGGCCTCCAGGATGAGCAGTCTGACCCACCCTCTGTGTCCCCTTACCATCAGGAAATAGTTGTGAATCTAGTCATTACAGACTATTGCATGCCTGGGATGACTGGCTATGACCTCCTCAAGAAAATTAAGGTATTCATCCCCAGAGTTCTGATCATTATTTATGTGATATGCTGTTGTATCTTTGGATTTAGCTTTATATGTTCATGTGTCCAAGAGAATTAAGGGAGCCCTATGATTGTTGAATCAGGAGTCATCATCCCTGAAGGACATCCCCGTTGTGATCATGTCCTCCGAGAATGTGCCCTCCC

>TRINITY_DN24193_c0_g1_i6_1
CCCCGCCCCCTCTCCTCCTCCTCCGCCTCCCAGTTCCACATCACTCACTCCTTGGCAATCCAATCCGTCTTCCATTTTTCTAGTCCGCTTTCCCCACCGGGAAAAGCGAGCGGCTGGAAGATTGAGAGCGCAGGCAGCCACCGCTCCTTGGAGGCCGCGCCCCCCCCAACCCATCGCCAGAGCCCCCGACCCATCCCCCTCGCCCAGCCTTATCCCTCGTCTCTCGCCACTCACCACAGTTCTTCTTCTTCCTCTTCGAAGCCAACGGCGTCGGTGTGCCCCAGCCAGCACAGTCCAGGTTCTGTGAGTTTCTGACACCGCAATCCCATATATACCACGGCGATATAGCAAGGAGGGCAGATGAAGCGCGAGTATACCGACGGCAGAGCCTGCCGCGGCGGCGGCGACGCTTCGGACGGCTACGGCGCGGCCTGCGGCGACGGCGGCGGCGGCTTCATCGGCGGCAAGAGCAAGATGATGATGCCGGAGGAGGAGCAGCAGGATACCGGCGTGGATGAGCTTCTGGCGGCGCTGGGCTACAAGGTGCGGTCTTCTGATATGGCCGACGTGGCCCAGAAGCTGGAGCAGCTGGAGATGGCCATGGTTAGCGATCATGGCGGCCTCGGCGGCGGCGCTCTGGACGATAACACCGTCCTTGCCCACCTTGCCTCCGAGACCGTCCACTACAACCCCTCTGATCTCTCCAACTGGCTCGAAAACATGCTCTCTGAGCTCAACGCACCTTCCTTCCCGCCTGCCCCTCCCGTGAAGGCCCCTTCATCCTCGTCGTCGACCTCCACCGCCATGGATCACCAGCATCTGCGGGGGCACCCTGCCGGGCCCTCCTCTTCTCATCATCCATCGGCAATCCTCATGGAGGATCCGCCCCCTACCGCGGAATCGTCCATCACCACGATCGATTTCCCAGACCCGACGAGGCCGTCCACGGACTACGAGCTCCATGCTATTCCTGGCGGCCGGGTCATGTACGGTACTAGTGAGTCGCCTCAAGCAACATCCAGGGACAAAAAGCGATTGAAATCGTCGTCGGCAGCAGCGTCGGCGCCATCAACAAGGGGGACGACGCTGCCAGCGGCGTCGACGGCCTCCAGCCTTGCCCAGTCCTCGTCGTCCCCGGAGTCTGCGATGCCTGTGGTGCTGGTAGACTCGCAGGAGACTGGGATCAGGCTTGTGCACACCTTGATGGCGTGCGCCGAGGCCGTGCAGCAGGAAAACCTCAAGGCCGCGGAGGCGCTGGTGAAGCAAATAGGTGTGCTCGCCATGTCGCAGGGAGGTGCCATGCGGAAGGTCGCCACCTACTTCGCGGAGGCCCTCGCCCGGAGGATATACGGGTTTCACCCCGCGCAGGACCAGTCTCTGGTCGACGCCGCCTTCGCCGACATCCTCCAGATGCACTTCTACGAGAGCTGTCCGTATCTCAAGTTTGCTCATTTCACCGCCAACCAGGCCATCCTTGAGGCCTTCGCCGGCAAGAGCCGTGTCCACGTCATAGATTTCAGCATGAAACAGGGGCTGCAGTGGCCGGCGCTGATGCAGGCCCTGGCCCTCCGCCCCGGTGGCCCTCCCTCTTTCCGGCTCACAGGCATCGGCCCGCCGCAGCCGGACAACACCGACGCTCTGCAACAGGTTGGATGGAAGCTGGCCCAACTAGCGGAGACTATGCACGTAGAGTTCGAGTACAGGGGATTCGTCGCCAATAGCCTGGCCGACCTCGAGCCCTTTATGCTCGATGTTGCATCTGCTCGGACATCTTCCTCGTCCTCTTCCTCCTCCGCCGGCAGGACGTCGGGTGGGGTTATCCAGAAGAGTTCTAGCAAGGGCGGCAAGAATGAAGGCGGCAGCTGTGGTGAAGCAGAGGAAGAGGAGGCCGTGGCAATCAATTCGGTGCTCGAGCTGCACAAGCTGCTGGGCAAGCCCGGCGCCATTGAGAAGGTACTGGGGACGGTGCGGGCGCTGCGGCCCAAGATCGTCACGGTGGTGGAGCAAGAGGCTAACCACAACAGCCCCGTGTTCCTGGAGCGGTTCAACGAGGCGCTGCACTACTACTCCACCATGTTCGACTCGCTGGAGGGTTGCAGCACTCCATCGCCCACGGCCGCGGGGATGAACGGCGGCGGTGGCAGCGCTGGGCAGGACCAGATGATGACGGAGGTCTACCTAGGGAGGCAGATCTGTAACGTGGTGGCGGGCGAAGGCGCGGAGAGGACCGAGAGGCACGAGACGATGGCGCAGTGGCGGGGAAGAATGGAGGCCGCCGGCTTCGAGCCGGTACATCTGGGCTCCAACGCCTTCAAGCAGGCGAGCATGCTGCTGGCGCTCTTCGCCGGCGGCGACGGCTACCGGGTGGAGGAAAAGGACGGCTGCCTAATGCTTGGCTGGCACACCCGATCGCTCATCTCCACCTCCGCCTGGCGGCTCTCCGGCTCGCCGCCGCCAGCCGCCGCCGCCGCCCGCTGACACAATACAGAGTCTTCGGGTTGCACGCCTCCTCGCCGAACGATGGGCGTTCAAAGCCGTCTGATCGCTGATCTGCGTCCATCAACGTTCCGTCCATATCGTCAAGGCTCCGATCCTTCTGGGAACCAGACCAATGATGCCTCTCTCTCTCTCTCTCTCTCTCTCTCTCTCTCTCTGCAGTAGGACATCAAGCCTTTTCCTCTCTTTTTTTGTTTAATTATCTCTGCTCCCTTTCTCTTTAAATTTATCTCCTTTTTATTTTTCCTTTTCGTTCGGCCCTCTGATGAGGAGGTTTTTCTCCGGTTCTCCACTGACCTCCCCATCTCTCCTGGTTCTGGGGACTACTACTACTACTCGACCATCTCCGTCGGCATCCGCCATCGCCCACTGAACTGTGCATCTGCCCTTCAAACTATGGATATTATTCCCCTACTAGAGAAAAATAAGAAAGGAGAGGCTTTCTTCGTCCATATGTATTATTATTATTTGCCACTCGTTTTT

>TRINITY_DN29302_c0_g1_i4_4
TCTCTCTCCCTCCTCCTCAGAACACCGAGGCCCCCGGCGGCTCTGCTTCCCCCAGCCTCAGCCGCCTCCATGGGGCCCTACGGCACGAGTGCGGCCGGCAACGGCTGGTGCGGCGGCGACTTCGCGGGCAGCTCCCCCTGCGCCGTGCCCGACATCGACGGCCTCCTCGCCGAGGCCGGCTACCGTGTCCGCTCTTCCGATCTCCGCCACGTCGCGCACCGCCTCGAGCGCCTGGAGTCCGCCATGGTTGGCGGCGCCATCCCCGGGTGCTTCGGATCGGCGGCCGCGCAGCTTGACCAGCACCAGCACCAGCAACAGCAGCAGCAGCAGCAGCAGCTCTTCCTTCCCCCCGACGCCGTCCACTACAACCCCTCCGACCTCGCGGCCTGGGTGGACTCTATGCTCTCCGAGATCTCCCTCTCCCAGTCCGATTCCAACGCCGGGGTCCCTGGTCAGGACATCCCCCCCTGCCATCCAGTCGACGATCTCGCGGAGGCGTCGGCGTTGGATTCGGCATTGGGTACGCCGGAGCAGCTCCTCCATGGCGGCATGCAGCAGGCCCCGCTTCCGCCCGTCATGGCGCCGCAGCCGCTGGTCGAGGAGGAGGACTCCGGGATCCGGTTGGTCCACATCCTGATGACCTGCGCCGAGGCCGTGCAACGAGGCGACGCGGCGCTGGCGGCGGCGCTGATCGAGGAAATGAGGCCGGCCCT

>TRINITY_DN29715_c0_g1_i2_1
TCTTATCACACTTAGCCGGCTTTCTCTTATAAATTAGCTTATTCCTCTTTCCTGATCATGTCAAATATTCGCCAAGAATCTCATATCGATTTTGTGTGCTCCCGTCGGTCGATGTCTGTGCCGTGTAGCGCTGCTGGGATCCGGCAGCCATGAAGGAGGAGAAGCCGTCGGTATGGGAATAGCGGCACCTCCACCATCGCACCTCGCCAGCAGCAGCTACTACCACTATCACACGAAGCCGCACCACTCGGGACCCCTAGCCGGGGCCAACCCAGCGATTTCTTCTATGTCCCAGGGCTTCCACCAGGGCATCTACAGCTTCTCCGATGGCTTCGACAGACCAACACACGCGGCCGCCCCTACGCAGCAGCAGCAGCAGCAGCAGCAGCACGTCGCGCAGCAGAGCCGGAGGGACAAATTGAGGGTGCAAGGCTTCGACCCCACGGGGGCCCCCCTGGTTCCCCTAGACGATGGGGAGGAGGCCATTTATGAGTCCCCCGGCGCCGCCGGCAGCATGTTGTCGGACATGTTCAATTTTGCCGCTGCCGGCGGACCGTCCTCCACCGACCTGCTCGCCAGCCAGATCCCGGCCAATTACCGCATCCAGAGGCCCGCAACAGCGGCTCCGGGGTTCTCCGGTGACTGGTACGGTGGCGGCAACAGGGAGGCCGGGTTGGTGGTGGGTGGCGGTCTGGGCTCCCTTGGGGACATGGCGAAGCTGCAAGGCGGCAACCCGGCGCCGCAGCATCAGCTGCACGGTTTGAATGCCGACTCGGCCGCCGCGATGCACCTCTTTCTGATGAACCCGCTCCAGCAGCACCAGCAACAGCAGCAGCAGCAGCAGCAGCAGCTCTTCCTCCCCCCCGACGCCATCCACTACAACCCCTCCGATCTCGCGGCCTGGGTGGACTCCATGCTCTCCGAGATCTCCCTCTCCCAGTCCGATTCCGACGCCGGAGTCCCTGGTCAGGAGATCCCCCCCTGCCATTCGGAGGCGTCGGTGTTGGATTCGGCATTGGATTTGGGTACGCCGGAGCAGCTCCTCCATGTCGGCATGCAGCAGGCCCCGCTTCCGCCGGTCATGGCGCCGCAGCCGCTGGTGGAGGAGGAGGACTCCGGGATCCGGTTGGTCCACATCCTGATGACCTGCGCCGAGGCCGTGCAACGAGGCGACGCGGCGCTGGCGGGGGCGCTGATCGAGGAAATGAGGCCGGCCCTGGCGCGCGTCGGCACCAGGTTCGGCATCGGCAAGGTGGCCGGTTACTTCATCGACGCGCTGAGCCGGCGGCTCTACTCGCCGCCGCGCGCAGTGGCGGCCACGGCGGCGGAGAGCGAGGTGCTGTACCACCACTTCTACGAGGCCTGCCCCTACCTCAAGTTCGCCCACTTCACCGCCAACCAGGCCATCCTGGAGGCCTTCGACGGCCGCGACCGCGTCCACGTCGTCGATTTCAACCTCTTGCAGGGCCTCCAGTGGCCGGCCCTGATCCAGGCCCTCGCCCTCCGCCCGGGCGGGCCGCCGGCTCTTCGCCTCACCGGGATCGGCCCGCCCTCCCCCGACGGCCGCGACACGCTCCGAGATGTAGGTTTCCGCCTGGCGGAGCTGGCGAGGTCCGTGGGCGTGCCTTTCGCCTTCCGCGGCGTGGCGGCCAACCGCCTGGACGACGTGCGTCCCTGGATGCTACAGGTGACCCCGGGGGAGGCCGTCGCCGTTAACTCGGTGATGCAGCTCCACCGCCTCCTGGGCGACTCGGACGAGGACGACGCCGCAGGCGGCGGCGGCGGCCCCGCGACGCCGATCGAGTCCGTACTGGCTTGGATCCGGGGGCTGCGGCCCGAAATCGTGGCCGTGGTGGAGCAGGAGGCGAACCATAACAAGGCGGCGTTCCTGGACCGGTTCACGGAAGCGCTGTTCTACTACTCAACCATGTTCGACTCGCTAGAGGGCGGCGCGCAGCAGCAGCACATGGGGGTCGTCGTGTCGGCGGGGGAGCGGGCCGCGGCGGAGGCCTATCTCCAGCGAGAGATCTGCAACATCGTCTGCTGCGAGGGCGCCGCGAGGGTGGAGCGGCACGAGCCGCTGGCGCGGTGGCGGGGGAGGATGGGCAGGGCGGGGTTCCGGCCGGCTCACCTCGGCTCCAACGCCTTCAAGCAAGCCAGCATGCTGCTCACCCTCTTCTCCGAGGAAGGCTACTGCGTGGAGGAGGTCGACGGCTGCCTGACGCTGGGGTGGCACAGCCGGCCTCTCATCTCCGCCTCCGCCTGGAGGGCCGACGACCTCGACGCCGAAGCCGCTGCCGGGGAGGAGGAAGAGGACGAGCACCACGTGGCGCCACTGCCGGTCCTCCCGGCGGCACCACCATTGGCAGCACAGCAGCACAACAGCAACAGTAACGGCACCAGTTACAACAGCATTGCGCATAACAACAACAACAGCAGCAGCAGCAACAGCAAAAACAACACAGGCGGAGGCGGAGGCGGAGGCAGTGGCGGTAGCAACAGTAGCAGTAGTACTAATAACAACCACCACCACCACCACCACAGCAACAAGCACAACGGTAACATTAACATGAGCAGCAACAGCAGCGCCTGGAGCAGCAGCAGCGGCAGCGGCAACGGCAACAACAATAACGGCGCCACCACCACCTGTTCTACTAGCAGCAGCAATAACCACCACCATTGCCGTCAGATGAGAATTATATGAACTACTAAGGTGCATGATCCTGATGAAGTACCCTTCCGAGTCTTATGATTAGAGACCCACAAACGAGTGTTCTTCTCTCTCTCTCTCCCTCGCTCGCCATTAAAATTCTGATCTTTTATCAAGGAGAGCTGATCCTTCAAAACCCATGTGATCTACCTCCTGTGAATAGTACTAGCGGCTCAATGATCAGTTGTCTGTGTAATAAAATAGATAAAAAGTCGATTCTGCAGTTCCCCAGTACTGATCTGCTGCTAGCTAGAGAGCATCTCCGGCACCCAGCTGACTCTGATCGGGGGAGCGTGGGTGGTAGAGCATGGATACTTGAATAATGAACACTACTGCTTCCAAC

>TRINITY_DN22427_c1_g1_i1_2
TCTGCTCAGCAACACAGCCGCTCTGGTAGTGTCTCTCCGGTGAGCAAGAAGACGACCACAAGACCAGAGCAGGGCGAAGAGGAAGGAGGGAAAGCAAGAGACTGCGAGCAGGCACAGAGAGAGAGAGAGAGAGAGAGAGTGGGAGAGGACAGGAGAAGCCGGGGAGTGCCAGTTTAGAGATATCTGCGCAGCGATGAGGATGGCGGCGAGGAGGAGCAGGGACTCCACCGCCGCCTGCGCTGGTGGCCGCGACCACCACCACCACCCTCCGTGAACCTTCCTTCCCATCGAGCTCATCCAAACCCACCCCGCACTATTCCGCAGACACGTGCCTCCGGTGGTCCCCCTCCGGCCCCTTCTAAAGCTCCGCCGTGGTAGAGCTATTTAAAGGCCATTGCGCCACCTGCTGCTCTCACCATGCCGGATCTGCTCAGCAACACAGCCGCGGTGGTGGTGTCTCTCCGGTGGTGAGCAAGAAGACGACCACAAGACCAAAGCAGGGCGAAGAGGAAGGAGGGAAAGCAGGCGACTGCGAGCAGGCAGAGAGAGAGAGAGAGAGTGGGAGAGGAGAGGAGAAGCCGAGGAGTGCCAGTTTAGAGATACCTGCGCAGCGATGAGGATGGCGGCGAGGAGGAGCAGGGACGCCGAGGCCGAGCTTAACCTGCCACCGGGTTTCCGCTTCCACCCGACCGACGAGGAGCTGGTTCTGCACTACCTTTGCCGCAAGGCCTCCTACCAGTGCCTCCCCGTCCCCATCATCGCCGAGGTCGACCTCTACAAGTACGACCCATGGCAGCTCCCTGAGAAGGCGCTCTTCGGGAACAAGGAGTGGTATTTCTTCACGCCGAGGGACAGGAAGTACCCTAACGGGTCGAGGCCCAACAGGGCCGCCGGGAGCGGGTACTGGAAGGCCACGGGCGCCGACAAGCCGGTGACGCCGGCGGGGAGCGCCAGGCCCCTCGGCATCAAGAAGGCCCTGGTGTTCTACGCCGGCAAGGCCCCCAAGGGCGTCAAGACCAACTGGATCATGCACGAGTACCGACTCGCCGACACCAACCGCGCCGCCAACAGCAAAAAGTCCGGCGGCAGCCTCCGGCTTGACGACTGGGTTCTCTGCCGCCTGTACAACAAGAA

>TRINITY_DN24731_c0_g1_i8_4
ATCGGCCGCAGCAGAGACCGAAACGATCGAACTGCGACGCTTCTATCTGCCGTGCGACTACCATTACTTGTCTCAGATCTGCAGTAGTATACTTACTTGCGAGCTGAGGTCCTAAGTGCCGGCACTGCTTCCAAGCTGCCGCAGCTGCACGGAAACCTTGGAGATCATGAACTGGTCGGAGCTGGACCTGCCGGGGTTCCGGTTCCACCCCACCGAAGAGGAGCTGCTCGACTTCTACCTCAAGGGCGCCGTCCGCGGCAAGAAGCTCCAGCCCGAGATCATCCACACCCTCAACATCTACCGCTACCATCCCGTGGAGCTCCCCGGGATGGCGAGGAACATCGGGGAGAGGGAGTGGTACTTCTTCGTGCCGAGGGACAGGAGGAGCAGCAACGGCGGCCGGCCGAGCCGGACGACCGACAAGGGCTTCTGGAAGGCCACCGGCACCGACCGGACCATCCGCAGCGCCGCCGACCCCAGGCGGAAGATAGGCGTGAAGAAGACGCTGGTCTTCTACGAAGGCCGCGCCCCCCGCGGCACCAGGACCGACTGGGTCATGAACGAGTACCGGCTGCCCGAATCGGACAACACAACCGCCGCCGCCGACGACAGGAGTACCCGTGCGTCCGATATTGTTATGTTGCAGGAGGACGTAGTGCTGTGCAGGATCTACCGCAAGGCCAAGTCGATGAAGGAGCTGGAGCAGCGGGCAGCCATGGACAAAGACGCCTGCTGGGGCGGCGCCGCGCAGTGCGGTTCTATTAATAGTACTTCTAACACCTCCGGCGCGACGTCGACCTTAATGATCTCGTCCTCTTCCTCGGACCAGAAGACGACGGCGCCGCTGCACAAGCTGTCGCGCACGGTTCCAGTAGTACAGCAGCTGGTCATGGGGGAGGAGGTGGAGACAAAGAAGGAGATGATCATGATGAAGGTGGAGACGTCGTCGACGGAGACCGAGAAGGATCAGGCCGCCGAGGTCACGTCCCTCGCGGTGGTGGGCTCGGGGGACCCGGCCGCAGCCCTGACGAGGACGACGACGACGCTGCCGGAGCTGGACAAGTTGCAGGTGCCGCCGCGCGAGGGGTTCTTCGACTGGGCACAGCACGATCCGTTCCTGGCGCAGCTGCGCAGCCCATGGCTGGACAACTGGTCTCCCTTGTACGCAAACATCCTCAATTTCTCTAGCTAGCTAGCTAGAGGATCACTTACTGAAAAAAATAGCTAGCTAGAGGATCGACGACAAATAATTAATCCGAACTATATAGTCTGTAGATCTGTACAGAGATCGATGTTGGCATGCATTTGATCCAAATAGGGAAATTAGCTGTCCAATGTATATCGAGATCAGCCAATGCATAAGATCATAACATGATATAGAAGAGATCGTGAATAATCTGTGGATATAATTGTACGTTCAGGAAG

>TRINITY_DN26997_c0_g1_i3_4
CAGACACTTGCTCGAGTTCTACCTCCGCCGCTGCGCCCACTGCAACTCGCCCAAGGGAGATCCCGCAGCAGAAGAAGAAGAAGAAGAGGGGAGCTCGGGTTACAGGTGGTGGTAGCAGCGCCGCCCTTGTCGCCTTCGTTTGGGGATTCCGGTGCGGGGAAAGGGAAGGGGAGCGCCGGAGCCGACGCGAGAGATGGGGAGGACCAGAGACGCCGAGTCGGAGCTCAACCTGCCGCCGGGGTTCCGCTTCCACCCCACCGACGAGGAGCTCGTGGCGCACTACCTCTGCCGGAAAGCCGCGCAGCAGCGCCTCCCCGTCTCCATCATCGCCGAGATCGACCTCTACAAGTACGACCCATGGCAGCTCCCAGAGATGGCGCTGTTCGGGAGCAGGGAGTGGTACTTCTTCACGCCGCGGGACCGCAAGTACCCCAACGGGTCCCGGCCCAACAGGGCCGCCGGGAGCGGGTACTGGAAGGCCACCGGCGCCGACAAGCCCATCACCCCCGCCGGCAGCGCGCGGCCCCTCGGCATCAAGAAGGCCCTCGTCTTCTACGCCGGCAAGGCGCCCAAGGGCGCCAAGACCGACTGGATCATGCACGAGTACCGCCTCGCCGACGTCAACCGTGCTGCGGCCAACAAGAAGGGCGCCGCTGCCGCCGGCGGCAGCCTCAGGCTGGACGACTGGGTCCTCTGCCGCCTGTACAACAAGAAGAACAACTGGCAGAGGACGACTGGGTCGGCCGGGGAAACGATGGACTCCTTCGAGGCTGCCGGCGACGCGGGATCGGAGAGCTTGAGGACGCCGGAGTCGGACGTGGACACCGAGGACTGCGGGGAGGAGCAGGGAGGTTTCCGTCGGCCCTTCTCCTTTGCGCCTCAGGCCCCCCACACAGCTGCGCAGGCTCCTTCTGGTGGCGCCATGGTCGGCAGTGTGAAGGAAGAGAACGACTGGTTCATGGACCTGCACCTGGACGACCTGCAGAGCTCGCTGGTGGCCGCCGGAGTAGCTCCACTGGATTTGTCGTACCAAGATAGCTACTTGTTAGGCATGGCTTCGCCGCAGCTGAAACCCGGGCAGAACAACTTGCCACCCTTCTGAGGGGCGGAACACCCTCAGAATGGGGAAACCCCATGGTCGTCGCTGACTCTCTGTACATAGCAAGAACAGCTACACACAGGCTCCGAACGATGGTGGAAGACAAGGGAATAGGAAATGTTGCCAGATGAAGCCATGGTTAGGATTCTTTAGTGTTCTTTTTTCCTCATTTTATTAAGATTTGAAGATGCTGTTTGGATGGGATGGGGAAGCTACTGCACATATGTAAGGGCTGACTCATTTTCTTGTATTTTTGTTTCCCCTTGTTCTCTTTAGGGAGAGAA

>TRINITY_DN27640_c0_g1_i3_5
GATCGATCGGCCGCAGCAGAGACCGAAACGATCGAACTGCGACGCTTCTATCTGCCGTGCGACTACCATTACTTGTCTCAGATCTGCAGTAGTATACTTACTTGCGAGCTGAGGTCCTAAGTGCCGGCACTGCTTCCAAGCTGCCGCAGCTGCACGGAAACCTTGGAGATCATGAACTGGTCGGAGCTGGACCTGCCGGGGTTCCGGTTCCACCCCACCGAAGAGGAGCTGCTCGACTTCTACCTCAAGGGCGCCGTCCGCGGCAAGAAGCTCCAGCCCGAGATCATCCACACCCTCAACATCTACCGCTACCATCCCGTGGAGCTCCCCGGTGAGTTATACACGTACAAACGTAATTAAGTGATGACCGAAACCTACCTCCGGGGGCTAATTAATTGACGATATATCTCCGGTTGCAAAAATTTATTCGATCGTTGCAGGGATGGCGAGGAACATCGGGGAGAGGGAGTGGTACTTCTTCGTGCCGAGGGACAGGAGGAGCAGCAACGGCGGGCGGCCGAGCCGGACGACCGACAAGGGCTTCTGGAAGGCCACCGGCACCGACCGGACCATCCGCAGCGCCGCCGACCCCAGGC

>TRINITY_DN28822_c0_g1_i1_4
CGTTTGTGTTTTGCTCTACATATATAGCAGCCTCCATGGCTAGCTCACTAAACTAGCTGGTTTCTGAGAGCGGGAAGATCCCAGACGCAACACACCCAAGTCTGGCTGCTGCGATATGGATCTGGACGGCTGCTACGTCCCGCTCCCCGGCAGAGACGACGACAACGTCGTCGGCGATGAGCAACTGACGAAGCTGCCCCTGGGCTTCCGCTTCCACCCCACCGACGAGGAGCTCGTCACCTACTACCTCTCCAGGAAGGTCGCCAACCACCGCTTCTCCGCCGGCGTCATCGGCGAGGTCGACCTCAACAAGTGCGAGCCATGGGACCTCCCCTGGAGGGGCGCGTCGGCGACGGGGCAGAAGGAGTGGTACTTCTTCTGCGTGCGGGACCGCAAGTACCCGGCGGGGCTGCGCGCCAACCGGGCCACCGACTCCGGCTACTGGAAGGCGACGGGCAAGGACAGGGAGGTCCGCCGGGGCAAGAGCCTGGTCGGCAGGAAGAAGACCCTCGTCTTCTACGAGGGCAGAGCACCCCATGGCGAGAAGACCTCCTGGGTCATGCACGAG

>TRINITY_DN28993_c1_g1_i5_2
CAATCGCATTCGTAGGATTCGTTGTTCTTTACGCTTTTATCTCGATCGATCTGAGGAGGGACGAGATCGGATTTGGTTCCCCTGGAGCTGCCTCCTCACTGGTGTCGTTCTCGTGCTGCTGCTGGTGGTGCTCGTCGCCTGTGGAAGAAGGGGTTTTGCAAGGGCTTTAGGCGTCTGCGATTGAGGGCTTTAGGCGTCTGTGATTGAGGGCGTGCGGCGTGGGGCAGTGGGAGTTTCAATGGAAGCCATAATTGGACTTTCTCTGCCGCCTGGCTTTGGTTTCCATCCTACAGATGTGGAACTCATTTCTCACTATCTGAAGAGGAAAGTTCTTGGCCACAAAGTTGATTTTGAAGTGATAC

>TRINITY_DN25060_c0_g1_i5_4
CTCCACCTTGCAGGAGCAGTGGTCGTCTTCCTCCTCCTTCTACCTTCTTGATCTCCACTCCCCTCCTCCGACCGGAGAAAGCTAGCCCCCACCCCCGGCGCCCTCAGCTTCTCTATTGGAGCCATCTCCTGATCATATATGGAGCCGTCTGCCTCGTCGGCCGCTTGGACCGATCCCACGTCCCTCACCCTCGGCCTCAACCTAGGCGGCGGGGGCAGGGGAGGCGGCGCCGCCGCCTGCCCGCCGTTCCCCAAGGGAGAGCCGCCGGTGAAATTACCGTTTGCGAATATGCTCGGGGCGAGAGATTTCATGGTCGCTGAGAGGAGTACATATGCCCAGTCGTCTACCCCCGTCAAGCAGGAGGTGATCAGGAGCCACCCCGACAGCCGTGCTCGTGTTCTACTAGCTTCTTCGACTCCGAGCGTTGAGAGCAGTAGTACTGTTATGGAGATCGACCAGTTTCGGGTGTGCCTGCCGCAGGCGGGTAACTATTCCCTGGAAGCCGAGCTGGCCCGAGTGAGCGAGGAGAACAGGCGCCTGAACGAGCGGCTGGCGGCGGTGTGCGAGAGCTACAGCGCGCTGCAGAGCCAGCTGATGGAGGTGATGGCTGCGGCCGCCTCGGCCTCCTCGGAGAGGAGGCCTTCGTCCGACCCATCGCGGAAGAGGAAGGCCGCCGAGAGCCGCGAGTCGGTGAGCAACGAGGACGCCGGCGGCGGCGGCGGCGGCGCTGCCGCCGTGCAGGACGGGCGCAGCAGCTGCCGGACGGCCGGCGCCGCCAACTGCGTGGAGAGCACCTCCAGCGAGGAGCTGGACTCCTGCAAGAAGCTTCGGGAGGAGTCCAAGCTCAAGATCACCAAGGCCTACGTGCGCACCACCCCCTCCGACACAACCCTCGTAAGATCTCTTAACCTTATATATTTACATATACATATATATACATACATGCATATATATTTGCACATCTCTGCTGAGAAGAAGCAGTGGAGTTACATAAGAGAGATTAATGCTCACTTGGGTTCCCTTTGTTATGTGTTATCTCCATGCACAGGTGGTAAAGGATGGGTATCAATGGAGGAAATATGGGCAGAAGGTCACAAGGGATAACCCATCTCCCCGCGCTTACTTTAGGTGTTCCTTCGCGCCTGTGTGTCCTGTTAAGAAGAAGGTGCAAAGAAGTGTGGATGATCGGTCCATCCTGGTGGC

>TRINITY_DN27869_c0_g2_i8_3
AGCTTCGGGAGGAGTCCAAGCTCAAGATCACCAAGGCCTACGTGCGCACCACCCCCTCCGACACAACCCTCGTGGTAAAGGATGGGTATCAATGGAGGAAATATGGGCAGAAGGTCACAAGGGATAACCCATCTCCCCGCGCTTACTTTAGGTGTTCCTTCGCGCCTGTGTGTCCTGTTAAGAAGAAGGTGCAAAGAAGTGTAGAAGACCGGTCCATCCTGGTGGCCACCTACGAGGGCGAGCACAACCACCCGCACCCTTCCAAGCCCGAGGCCGCAGCCGCCGCCACTGCCGCCACAGCCGGGTCGAGATCCGGCGCCACCGCCTCCCCCAGAACGGCACAGGCGGCCGCCGCCACCCTCGACGTCGTGCCGCAGGCAGCACCGAGTTCTCACCGGAGTACTCCCGCCGCCCTAGGCTTCCCTGGCGGAGAGATGCAGGCGCCGCAGTTCCAGCAGCTGGTGGAGCAAATGGCCTCCTCCTTGACGCAGGACCCCAGCTTCACGGCGGCGCTGGCGACGGCCATCTCTGGGAGGATACTTAATTTGTAGGTCAAAAGCACTGCAGATCGAAGCA

>TRINITY_DN29800_c1_g1_i1_2
AGCGGGCCGGGGGGTTTCCAACAGGGCTGGCGGCGGCGTGCCCAAGTTCAAATCTTTGCCTCCGCCGTCGCTGCCCCTCTCCCCCACGCTGCTGTCCCCCTCTTCGTATTTCACCTTGCCCGCCGGCCTCAGCCCCGCCGCGCTCCTCGACTCCCCCGTCCTCCTCCCCTCTTCTAATCTTCTGTTTTCTCCAACCACGGGAACTTTCCCTGTTCAATCTCTCAACTGGAGGATGAACTCCCCTGCTTACCAGAAGGCCGACAAGGATAAGGAGAATAACTATTCTGATTTCTCCTTCCTAACAAGTTCAGCAAACGACAGCTCCTCGGCCTTCCAGAACTCCTCAGCATCCAAATCCACAGCGGAAGTCATGAGGGCGTCTGAGATCTCTGCGCCCACGGCCGGCACGAAACCAGAGCTCATCCCTTCTTCAACCAAGAACAACTCCTCGGAGGTCTCGTCCATGGCGTCCAACATCCAGAGCAACAACGCCGCGACCCGATCTGAGCAGAGGAGGTCGGAGGACGACGGGTACAACTGGAGGAAGTACGGGCAGAAGCAGGTGAAGGGGAGCGAGAACCCCCGGAGCTACTTCAAGTGCACCTACCCCAGCTGCCCCACGAAGAAGAAGGTGGAGAGGAACATGGAAGGGCGAATCACCGAGATCGTGTACAAGGGCGCCCACAACCACCCGAAGCCGCCGACCACCAGGAAGAACTCGTCGTTGCAGCAGATCCAGCCGCCTGCTCCGGCAGAGACATCGGAGGGCTCCTTCGGCGGCCGAGCGGGCACGCCGGCCGACTCCGCCGCGACTCCGGACAATTCTTCCGTCTCCCTCGGCGGCGACGACGACCTCGACATGAACTCTCAGAGGAGCCGGTCCGCCGGAGACGAGTTCGACGACGAAGAACGGGAAGCCAAACGCCGGAAGAAGGAAGAAGACGACGAGGGGGTCTCCGGCTCCGGGAACAGAGCTGTAAGGGAACCCAGAGTGGTGGTTCAGACCACCAGTGATATCGACATCCTTGACGACGGGTACAGATGGAGGAAGTACGGGCAGAAGGTGGTGAAGGGAAATCCCAACCCAAGGAGCTACTACAAGTGCACCAGCGTCGGGTGCCCGGTGAGAAAGCACGTGGAGAGGGCGTCGAACGACCTGCGCTCCGTCATCACCACCTACGAGGGGAAGCACAACCACGGCGTCCCGGCGGCCCGCGGATCCGGTGCCGCGGCCGCGAGCAGGCCGCCGACGGACAGCTGCGGCAGCATGGCCATGGCTGTGAGGCCGTCCGCCACGGCCGGCGTCGCGAGGCACGTGACGGCCAGCTCTCTCCTCGGCGGGAGGACACTGACGGCCGCCGGCGGCGGCCAAGCCCCGTTCACCTTGGAGATGCTGCAGCCGCCGGCGAACAACGGGTTCCCGGGGTTCGACGGATCGGCCGGGTCCCGCGAGGAGTTCTCGAAGGCGAAGGAGGAGGCGAGGGACGACATGTTCTTGGAGTCGGTGTTCTGCTAATTACTGGCGG

>TRINITY_DN30384_c0_g1_i11_1
CCCCCATCATCTTCCTCTCAAGAAATGGTGCGTATATAGGTTTCCATGGATCACCATCGCTCCCCACTGCACCCTTCTCCCTTCTGCTTGCTCGCCGCAAAGAGTGGAAGGTGATCAGAAAGAGAAGAGACAGGGAAGAACTCGAGAGGATTGCTCAGTTTGATGTGCTTTTGCAGTTGCTTTCCTCTTTCTGCATGTGGGTCCTTCTCAGAAGGCCACCGATCGCTGGGAGTTCGCCGGAAAGGAGGCTGGTTTGCTTCCTGCATTGATCTAGAAAGCTTCGACCCGTTTCTTCTCCCCATCTGGAGCTCTGTGCTCTGTTCTTCCTCTGTTCTTTCCTTGCTTCCCAGCTCCAAATTTGTCTAGAGCAGGAAGAAGAACACGGCCTTTTTCTTCTCAAAAAGGCCAGCTTGGAGGCACACTTCCGTGGAACTCGGATTTAGCGATGGAGAAGTGCGGCGCCTGGGGATCCAACATGCTGGTCAGCGAGCTGGCCCAAGGGCAGGAGCTGATGAGGCAGCTCAATGCCCTGCTCGAGCACTCCTCCACGCCCCCGCAGTGCAGGTTGCTGGCGCAGAGCATACAGTGCGTCCTGGAGAAGGCCATGTCCATGGCGAGCAGCGACTCCGGCGGGCCGCCCTCCGCCGCCGCGGGCGGGGAGTCGCCGAGGTCCGACAGCGGGAGCCCCCGGAGCGAGAATTCCGACAGGGTGCTCAAGGACCAGGAGCGCAGGGAGATGTGCAAGAAGAGGTAACCCACTACTCCTACTACCGCTGCACTTCGCGGAGGTAGCTGCAAACAAGTCCAAGATTGCTCCTTTTTTTCTTCTTCCCGGGATCTTCTCCGCTAATGGCTTCTCCACACCGGTATCTGCTGCAGGAAGACGCTGCCCAAGTGGACGAACCAGGTGCGCGTCTGCTCGGAGACGGCCCTGGAAGGCCCCCCCGACGACGGATTCAGCTGGAGGAAGTACGGGCAGAAGGACATCCTCGGCGCCAAGCACCCACGAGGCTACTACCGATGCACCCACCGCAACGCGCAGGGATGCCTGGCGACGAAGCAGGTGCAACGGTCGGACCAGAACCCGTGCGTCTTCGACATCACGTACCGCGGGACTCACACCTGCGTGCCGAGAGGAAGCTCTTCCCCGGCGCCGGCGCCGCGGCGGCAGCAGCAGCCGACGGGGAACGACTCCCCCAACCTGCGCAGCGACGCCGAGAACGCGTTCCTCCTCAGCTTCAAAGCCGGCCTCACGGTGAAGACCGAGGGCCTGGACGACCACGATCTGGCCTCGTCGCCCTTCTCCTTCCGTTCCACGCCGGTCGACCAGGAGGGCCACGTCTTCTCGTCGCAGTCGACCCCGCTGGACGCCCAGTTCGGGAGCAGCTTCTCCTCGCCGTTCATCTCGCCGGCGGCCTCTGGTTCCAGCTACTTCACGGTGTCGCCGTGTCAGGGGAGCGGCTTCGGGGGGAACCCTAACTTCCAACCTTTGGAATCCGACCTGACGGAGATAGTTCCCGCCGGCGCCTCCGCCACCAGTTCTCCGGTCTTTGACCTTGACATCATCCTGAACGCGGAGTTTGACCAGAACTTCCCCTTTGACTCCAGCTTCTTCTCATAGATA

>TRINITY_DN28417_c0_g1_i6_4
GAAAGTTTGGTAAAGGAAGCGACTTGTTGCGGCGGAGAGGCGTGTTGGGATTGTTCTTCCCGGTGGTGTTCTCTGCTGCCACCGCCAACCCACTGCCGTCTTTCTCTTCTTTCTGGTGTTGGGTTTTGCTGTTGGGAGCGTCGTGTGTGGAGGGGAGGGGGTATAAGGATGTGCAGCGGACCGGACGCCCTGAAGAGGAAGCCCGCCATGGCTCTCGGTCATGCCGACTGTGAGGGGACGGACGATGTGGGTGGGGAGAAGCGGAGCAGGGGTGCCGTCCTTCTCGAGCTCTCCGCCGCCGACGACCTTGCCGGCTTCCGGCGGGCCGTGGAGGAGGACGGGCTGGATGTGAACCTCGCGACCCCTTGGTACGGCCGTTGGGGGCGGAGGATGGGGCTCGAGCCGCGGACCCCGCTCATGGTCGCCGCCATGTACGGGAGCACCGCGGTGCTTGGGTACATCCTTGCCACCGGCGCCACCGACGTCAACCGAAGCTGCGGGTCCGACGGGGCCACGGCCCTCCACTGCGCCGCGGCCGGGGGAGCGCCGTCCTGCGTGGAGGCGGCCACGATGCTGGTGGACGCCCTCGCCAGCGTCGACGCGGTCGACGCCGCGGGGAACAGGCCCGGAGATGTGATCCCAAGGAAGAAGGTGGTCGCGCCCGGGGGTTCCCCCAAGGCGGGGCCACTCGAGGTGCTGCTGAAGGCCTCCAACTTCGGGGGCGGCGACGCGGCGGTAGCGAAGGAGTCGTCTTCCTCCTCTCCGGCGAAGAAGGAATACCCAGCGGACCTGACGCTGCCAGATATCAAGAACGGGGTTTACAGCACCGACGAGTTCCGGATGTATGCCTTCAAGGTGAAGCCCTGCTCAAGGGCTTACTCCCACGACTGGACGGAGTGCCCCTTCGTGCACCCCGGGGAGAACGCCCGGCGCCGAGACCCGCGCAAGTACCACTACAGCTGCGTGCCGTGCCCGGAGTTCCGGAAGGGCGCGTGCCGCAACGGGGACGCCTGCGATTACGCCCACGGGGTGTTCGAGAGTTGGC

>TRINITY_DN29028_c0_g1_i10_4
GCGAGAACGCCCGCCGGCGGGACCCGCGCCGGCACCACTACAGCTGCGTGCCGTGCCCGGAGTTCCGCAAGGGCGCGTGCCGCAGCGGCGACGCCTGCGAGTACGCCCACGGGGTGTTCGAGTGTTGGCTCCACCCGGCGCAGTACCGCACCCGGCTGTGCAAGGACGAGACGGGGTGCACCCGCCGGGTGTGCTTCTTCGCCCACAAGCCAGAGGAGCTGCGCGCTCTGTACCCGTCGGCCGCGCCTCCATCCCCCGCCGGCGGCTCCGTGTTGCCGTCCCCGAGGTCCTCGGTGCTGTCTCCTCTGGACGTGGCTACGGCCGCCCTGCTGCTGAACCAACCCCTGACGCCGTCGTCTTCCTCGTCTCCGATGGCTTCCGCCGCGGCCTGGCTGAACCAGGCGACCGGGGTCAAGACGCCCACCCTGCAGCTCCCTGCCAGCCGCCTCAAGGCGAGCTTGAGCTCCAGGAAGATCGACATGGACATGGAGTTGCTGGGTCTGGAAGGTTACCACCGGCAGCAGCAGCTCGTCGACGACATGGCAAACCTGTCTTCTCCTGGCTGGAAGAGCACCCTGGTTGCGGCGTCCACCGCAGACGCGTTTGCGTCTCTGAATTCGCCGTCGTTGCTCTCCCAGTTCCAGGGGCTTTCCCTGAGGCAGTCCTCCGCCGCCGCCACTGGAGCTGCCCAGTTCCAATCGCCGCACCAGAGCGTGAGCCAGCAGCAGCTGCTTTCCGGGTATAACAGCAGCAACCTTCTGCCGTCGCCATTATCGTCGTCGTCCTCATCATTTGGGCTCGACCACTCGCTGGCCAACGCCCTCGTTAATAACTCAAAGGCGTCTGCTTTTGCCAAGCGGAGCCAGAGCTTCATCGATCGCGGGGCGACTGGTCGCCCGTCCGGCTTCGGGATGGCGAACCTGGCAGCGCCGCCGCCGGCCCTGTCCGACTGGGGCTCCCCGGACGGGAAGCTGGACTGGGGAATCCATGGCGAGGAGCTTAACAAGCTGAGAAAGTGCGCCTCTTTCGGGCTCCGCAGCAACGGCAGCAGCGCCGGCGCCGGCGCGGAGGTGCCGCCGCTTGCGGGGCAACTGCGAAGGGAGGAGCAGTTGCAGAGGCAGTACCAGGTGAACAAGAACAACGAGTATAACCTTTCCGTGGAGATGCTCTCGCCATGGATGGAGCCAATGTATGCAGAGCAGGAACAGATGGTGGCATAATCGCCGTCACCCCAACCCCACCCTCTCCGCACCCATTATTCTTTTTTTTTTAAATATGTTATTAGTTTCCCTAATTTTCATGGATGCTGTCTTTGAAGAAGGTTGCAGGCGCACCCGAAGGGACGACGCTCCTCTAGAGGCAGTTCTATTCATTTGAAAAAAAAAA

>TRINITY_DN29417_c1_g3_i1_2
CTCCGGCATCGCCTGCCCCGAGTTCCGCAAGGGGTGCTGCAAGAAGGGGGACGCCTGCGAGTACGCCCACGGGGTGTTCGAGTGTTGGCTTCACCCGGCGAGGTACCGCACCCAGCCCTGCAAGGACGGGGTGAGCTGCCGCCGCCGGGTCTGCTTCTTCGCCCACACGCCGGAGCAGCTCCGGGTGCTCCCCCAGACGCAGCCGACGACGCAGCAGCAGTGCCCGCCGTCTCCGCGGGGGCTCGACGACGGATCCTCCCCGCTGCGTCACCCTTCTTCCCTGGATGCCTACTTCGCCAAGAACCTGATCTCGTCCCCAACCTCCACGCTGATCTCGCCGCCCATGTCGCCCCCCTCGGACTCCCCGCCGCTGTCCCCCAACGGGCCCTCGATCCGGCGGTTCTCCCTCCCCGTGGGGTCGTCGTCCATGAACGAGGTGCTGGCTTCCTTCCGGCAGCTCCAGCTGAGCAAGGCCAAGTCCGTGCCGGCCTCCTGGGGGATCCAGGTCGCCGCCGGCCTGGGCTCCCCGCGCGGGGTGGGCGTGAGGCCGGGGTTCTGCAGCAGCCTCCCCGCCACCCCGACCCGCACACCGATGGGCGGCTGCGGCGGGCTGTTCGAGGACTGGGGGGAGCCGACGGAGAGGGTGGAGTCCGGGAGGGACCTCAGAGCCAAGATCTACGAGAAGCTGAGCAAGGAAATCGGGATGGGGAGGGCCGAGGAGGAGGAGGACGGCGGCGGGCTCAGGGGCGGCGCCGCCGCCCCCGACGTCGGCTGGGTGTCGGATCTGCTGAAGTGAGATAACCGGTAACGGATACCTTACCGGGCGGTTGACGGATCTGATGGGAGA

>TRINITY_DN31472_c3_g1_i2_4
CGCGGCCGCCCCCTGGTACGGCCGGTGGGGGCGGAGGATGGGGTTCGAGCTGCGGACCCCGCTCATGGTGGCGGCCGCCTACGGCAGCACCGCGGTGATGGGGTATATCCTCGCGACCGGGGCGGTGGACGTCAACCGGGCATGCGGGTCCGACGGCGCCACCGCCCTCCACTGCGCTGCCGCGGGCGGGTCGCCGTCCCCCGCCGAGGCCGTGAGGATGCTGGTCGAGGCGTCGGCGGATGTGGACGCGGTCGACTCCCTGGGGAACAAGCCCGTGGACGTCGTCCCCCTGCGTGTGTCGTCCGGCGGGTCTGCGAAAGCCAGGTCTATGATGGCGCTGCTCAAAGGCGGGGACGATGCGGCGGCGGATGGGGAAGATTTCCCGTCGAAGAAGGAGGAGAAGAGAGGGGAGAAGAAGGAGTACCCGCCGGACTTGACCCTCCCCGACATCAAGAACGGGATCTACAGCAGCGACGAGTTCCGCATGTACACGTTCAAGGTGAAGCCGTGCTCGAGGGCCTACTCCCACGACTGGACCGAGTGCCCTTTCGTGCACCCCGGCGAGAACGCCCGCCGGCGGGACCCGCGCCGGCACCACTACAGCTGCGTGCCGTGCCCGGAGTTCCGGAAGGGCGCGTGCCGCAGCGGCGACGCCTGCGACTACGCCCACGGGGTGTTCGAGAGTTGGCTCCACCCGGCGCAGTACAGGACCCGCCTCTGCAAGGACGAGGTGGGGTGCAGCCGCCGGGTGTGTTTCTTCGCCCACAAGCCCGAGGAGCTCCGCGCTCCCACGTCGCCTGCGGGCTCCGTGCTTCCGTCGCCCAGGTCCCCCGCGCTGCCGCCGCTGGACGTCGCTGCTGCCGCCGCAGCTGCTTCCTTGATGCTGAACCAGCCCATGTCGCCCACCGCTGCCTCCTCCGCCATGGCCGCTGCCTCGGTGTGGCTGAACCAGGGTGGTGCCGGGATGGTGACGCCGACAAGGCCGCTCGCTGGAAGCCGGCTGGGGTTGTCATCGAGAAAATCAGACATCGACATGGATTTGCTCAGCCTGGATGGCTACCAGAGGCAGCTTCTCGACGAGTTCGCCGGTCTGTCTTCTCCGAAGAGCTGGAAGAGCTCCATGCCTGGTGCTGCTGCCGCCTCGCACGCGTCGGACTACGGTGACCTGCTTGGGTCTTTGGAGTCGCCGTCGCTTCTGTCTCAGCTCCAGGGGTTGTCTCTAAGGCAATCTCAGGCAGCCGTGGGCACCGGCGCAAGCCAATTCCAGTCCCCGGCTGGAATCCAGATGCAACAAAACATCAGCCAGCAGCTGCTCTCATCCTACAGCAACAGCAACCTGCCGTCCTCTCCATCCTTGAGGGCTTCCTCTGGTTCATCCTTCGGCATCGACCATTCTCTGGCCGCCGCCATCATGAACTCGCGGTCCTCCGCGTTCGCCAAGCGGAGCCAGAGCTTCATCGACCGCGGAGCGATGAGCCGCCTGTCCGGGCTGAGCACTGCGAACACGCTGGCTGCCGGGGCGGACCTCTCCAGCTGGGGCTCGCCGGACGGCAAGCTCGACTGGGGCATCCAAGGTGAGGAGCTCAACAAGCTGAGGAAATCGGCTTCCTTTGGCATCCGCAGCAACGGCGCGCCGGCGGCCATGGCCGCCAGCACGGAGGAACC

>TRINITY_DN23040_c0_g1_i5_5
CAGTCTTTTCCCGTGGGCTAGTCTTTGAAATCTGTACTAAGTCGGAATGGCTACCCTGGAGTCGCTGACGTCAACTGCAGCCGGTTCTAGTCCCTTCTCCAGCGCTGGCGACGACCTCCCCTTCCTCGAGCCATGGACGAAGCGGAAGCGCTCCAGGCGCGGCTCGCCCTCACCCAGCGAAGAGGAGTACCTCGCCCTCTGCCTCATCATGCTCGCCCGCGACAGCGGCGGCGGCGGACGCTTCCCTCCCCGCCGCCATTGCTCTCCCCCTCCGGGCCCGGCGGCGTCGAAGCTCCTCTTCAGCTGCTCCGTCTGCGGCAAGGCCTTCCCGTCCTACCAGGCGCTCGGCGGCCACAAAGCCAGCCACCGGAGACCCCTCGCCGCCCCGACCACCGAGGGGGCCGACTCCTCCGCCACCGCGACCGCCGTCTCTCTCGGCCCTGCCGCGACCGCTTCCTCCGCTTCCCTTGGCGGCGGGAGGCCGCACGAGTGCTCCCTGTGCGGCAAGGTGTTCCCCACGGGGCAGGCGCTGGGGGGTCACAAGCGGTGCCATTACTGGGAAATCGCCGCCGCCCACGGCGGGAGGAGTTTCAACCTCAACCTGCCGGCCCTGCCGGAGTTCGGCGCTGGGGCAAGCTCGTTTCCCCGCAATCACGATATCGAGGAGGACGAGGTTACTGGTCTGGCCAACAAGAGGCTACGGCTGCCGACCTCGGACGAAACGACTGCACTGTCTCCAGAGGAAACATGAGAGTCTTGTTATTTTTCTTTCCGCTTCATTCGTCCATTCCTTTGTAGTAAGTTTGTACAGTTCATCAGGATTCGTTTGATGTTCTTTGCTCGATTTTTGTT

>TRINITY_DN24286_c0_g1_i1_1
CTCTCTCTCTCTCTCTCTCTCTCTCTCTCTCTCTCTCTCTCTCTCTCTTGTCACACACATTCCTTCCCCTTTCTTCTCCACTCCTTCCTTTCGGATCTCCTCTACAAGCATTTATGGCTGCTTTCATTCTTTGTCCCTCATAGAATATATAGATTTCGTAGTTCTTGGGTTTCTAAATCATTGGATTGTTCATTGGTCCAGCAGGTGTTGTTCTTGGCAGCAGGGCTTAATTCTTCACTCGGTGTGTGCATGCTGATGAGAGAGAGAGAGAGAGAGAGAGAGAGAGAGTTTGGTGCAGCTCCATTCCCCCCAGCTAGACCCCTTTTGGCCTCCTCCTTTTCTTCAGTATAGCCCCTTTTTTTCTTTGCTTTTGCCTCTTCCTTTTCTTCCTCCTCTCCGTTCCCAAGAAAGCATCTTTTTCCCCCCTTCCCTACAGTGAGCATCTGTTTGGTACTTGACAGAATTGCATGCTCAGCTAGATCTGTGGGCAATAATCCCCACCTTCATTCCATCTGCTTTCTCCTCTGTACGTAATTTCAGTGGGATATTCTCCTCTCTAGCTAGCTCATGCCTCCTGGCAACCTGTGTAAAGTGTGGAATATGCATATATGATCAGAGAAGCAGCGCTTTAATTTGTGACCTTCTGATACCTGTGATGGGTTCCTTTTATCAGTATTCGGAGGAGGTATAGATGTAGCTGCTGCTCGAGCTATTTTTTTTTTTAGGTAAGTAGCTGCTGCTCGAGCTAGCTAGTTCCTGCTTATAATTATACTTTTTTTCTCTCCCACCGGGGACATATATATGTATGTACCGCTCCAAACCCTAGAATTGTCTGTGATATCCTCCCACCCCAAGGAGAACCGACCAGTTGGGAGATCTGTAGCAGTTGCGCGTGGAGCTTCTGATGAAAGGGTTGCTAATTCAGCAACACCCAGCTGCTGCAGAGGAGAACATGTCCAATCTCACCTCAGCATCCGGTGAAGCCTGCAGCGGCTCCTCCAACCAGCAGTCCTTCGCGTCTGATCAGAATCCAAATCCCGGCCCAGCGAAGAAGAAGCGGAAACTCCCTGGCAATCCAGACCCAGATGCGGAAGTGATGGCGCTGTCGCCCAAGACGCTGCTGTCGACGAACAGGTTCGTGTGCGAGATCTGCAACAAGGGGTTCCAGAGGGACCAGAACCTGCAGCTGCACCGGAGGGGGCACAACCTGCCGTGGAAGCTGCGGCAGCGGTCGAGCGAGGCGAAGAAGAAGGTGTACATCTGCCCCGAGGCCAACTGCGTGCACCACGACCCCGCCAGGGCGCTGGGCGACCTCACCGGCATCAAGAAGCACTTCTGCCGGAAGCACGGCGAGAAGAAGTGGAAGTGCGACAAGTGCTCCAAGAAATACG

>TRINITY_DN25451_c0_g1_i4_4
GGTTTCTTCACCACAGCAAGCTTCGACGCATCTCACAACAAACAGATAGAGAGAGAACGAGAGAGAGAGAGAGGCGTAGTGTTTGATCCTCCCGGTGGTAAGAACACAGTTGACGGCGTTTGTTTCTTGTCCCCCATCGTCTATATACAGTTCGCCGGTGAGCATGGCCATCCTGGGCATGAAGCGGTTCAGGACTGAGGAATATGACGGCGGCCAGGTGATGGACGGCGCCGGCGATGCGGCCGACCTCCTGATGATGCTCTCGCGCCGCACCGGTGCCGGAGAAGTCGGGCGACGGCTGTTCAGGTGCAAGTCCTGCGGGCGCCAGTTCCCGTCGTTCCAGGCGCTGGGCGGGCACCGGGCAAGCCACAAGCGGCCTCGCCTCGCAGGGGAGACGCAGGTGGTGCCGGAGAAGCGGAGGGCGCACGAGTGCCCCATCTGCGGCGTGGAGTTCGCCATGGGGCAGGCGCTGGGCGGCCACATGCGGCGGCACCGCACCGGCGCCGGGGCCTTCCCGCTGGTGTTCGCGGAGAAGAAGCCCGGCGTCGGCGCCGGCGAGAGGCGAGGGGTCCTCAGCTTGGACCTGGACCTGAACCTGCCGCCGACGTCGGACAAAGACGGCGACTTCTGCTCGCTAAAGCTGGGGTTGGGGCATCACGTTGTGGGTTTTTTGTATTAATTGATCCCTTCAGAGTTCAGACCATTGTGTAGTTCTGCTGTTTCTCTCAAACCCCATGGTTAGATTTTAGGGTTTTTTATTTTTCTTAATTTATTCTTTCATTTATTTTTTGTAATCTGGGATGTTATTTTTGTACAACTTTTGCTTG

>TRINITY_DN25639_c0_g1_i3_4
CCCCCTCAACACCGCCACAACAACAACTCCAGCAAGAAAGCTAGCTAGCTCCATCCCACTGACCTACCTGTCCGCTCCCCCTTACCTCTCCCGCTAGCTAGCTCAGCTCCCACCGCTGGTTCCTTCTCCGCTGCAATTCCCACTAGATCAGATCGAGTTCCCAGCCATGGTTCTCGACGCCCTGAACACCGCGGAGTTGCCCAAGGTGCTCGCCCCGCCGGCCGGGGTCGAGGAGGAGGTGGAGGCGGCGCCGTGCCCGGGGTCGACGTGGGCGAAGCGGAAGCGGTCCAAGCGCCCCGGCCGCTTCGGGGATCACCCCCCTACCGAGGAGGAGTACCTCGCTCTCTGCCTCGTCATGCTCGCCCGCGGGGAGCACGGCCGTTCTTCCCGCCTCGGCTCGCCCTCCCCGCCGCCTCCCCAGCTGCCGCAGCAGCTGTCCTACAAGTGCTCCGTCTGCGGCAAGGCGTTCCCCTCCTACCAGGCCCTGGGCGGGCACAAGGCCAGCCACCGGAAGCTCGTCGGCGGCAGCGGCGTTGCCGAGGACGTCGCCTCATCCTCGGTCAACGCCGGATCCCCGGCGGTGGCCAGCGGCGGGCGGGCGCACCAGTGCTCCATCTGCCTCAGGACGTTCCCCTCCGGCCAGGCCCTGGGGGGCCACAAGAGGTGCCACTACGACGGTGGCGCCGCCAGCGCTAGCGCCAGCGCCAGCGGTCCCGGCGCCGTATCCTCCTCCGAGGTGACCGACTCGAGCCTCCGCAAGTTCGACCTCAACCTGCCGGCCCCGCTCGAGTTCGTGTTCGACGGCGCCGCCGCCGCGACCAGGAGATGCGTGGCCGCGGAGGAGGACGAGGTCCAGAGCCCGCTCGCCTTCAAGAAGCCTCGCTTCCTTATTCCAGCCTAGACAGAAATTAATTAATTAAAGATCCCTCCTTTTTCTCTCTCGTCTCTCTCCTCTGTAACTGTTTTCTTCTCGCGAAGGGTTTGGGATGAATCCGAATTCTTTTGTACAGAGAATCGAATTCGTTCTTCCTTGTAAACGTACTCTGCTATTCATTCTTCTCATATGTTCTTGCGATCGATATCAACAATTCAATTCATTTGATTGATCATCTCCA

>TRINITY_DN26831_c0_g1_i1_2
GCCTTTTTGTGCGCGACGCCGGGAGTGTTTGGTGTTTAATATATGTATGATGGCGAGGGTTTTAACAGGTCCGGGCCATTTTTTTAACCTTAATTAGATGCTGAGATTTGAGGGCTGCGGAGGTCTGCGCGGAGGGAAGAAGCTGCGACAACGCCCAAAACCTGACTGCTTTGACATCTGGGAGAGGTGGAGGTAGCTATAGAGAATGGATTATAAGCAGAAGCAAATCTGTGAAACCTCCACACAGGCTTCTACGGTCGCCAGAGATCAGCCAAGAATCATGAGCTCAAACCAGCAGTTTGCTCACTTTGATTCTCCAAATAATTGTTATAGTCTTCAAAATGAGCAGTCTTTCCCTGAGTTTGCCCCAGGTTTGGATCCAGGATCCCGACATGCTTATCCAACTGACAAAGGCCAATCCAATAGCGAAATGAATTGGGGGAATCATGTGAATCCAACTTCTGTCAACAGCACGACTATGGGTTGTGATCCAAGAGCTATGTTAAGCAACTTGTCCTTCCTGGAGCAAAAGATTCACCAGGTTCAGGATATAGTCCGTTCAATTATGAACCAAGAAGGTCAGATCTGCAACCAGCCAAATGAATTGGCAGCGCAGCAGCAGCTAGTGACAGCTGATCTGACTTTAATCATAATTCAGTTAATATCCACTGCTGGTACTCTTCTTCCATCTATCAATGGGACACTTTTATCAACTAATTCTTCTGTTGAGCAGTTGGGGAGCATCATCGGCTCTTCTACTAGCCTGGTAGGTTTGAATGTCAACATGGAGCAAAGTGTGGTCTTTACGTCAGAGGAGGCAAAGACTCCGGAACATGAGGGGCAAATCAAAGGGACGAGTAAAAATTGTGTGGAGGAGCGCTCCCCTGTAGAAGAACAAGATGGGAAAGACCTTGAAGATGGAGGAGAAATTGAAAACCTCCCACCAGGTTCTTATGAGGTCTTGCAGCTGGAAAAGGAGGAAATTTTGGCACCACACACCCATTTCTGCACAATTTGTGGGAAGGGTTTTAAGAGAGATGCAAACTTACGAATGCATATGAGGGGCCATGGAGATGAGTACAAGACTGCGGCGGCACTTGCGAAGCCTAGCAAAGATCAGAACTCTGAACCTGTGCTTATAAAGAGATATTCCTGCCCATTTGTAGGTTGCAAGCGGAACAAGGAGCACAAGAAGTTTCAGCCTTTAAAGACCATCCTCTGTGTTAAAAACCATTACAAGAGGAGTCACTGTGATAAGAGGTATGTCTGCAGCAGGTGCAACAGCAAGAAGTTTTCTGTCATTGCAGATCTGAAGACTCATGAAAAGCACTGTGGGCGTGATAAATGGATTTGCTCTTGTGGAACAACCTTCTCGAGAAAGGATAAGTTATTTGGGCATGTTTCTCTATTTCAAGGTCATGCTCCAGCTCTTCCCCTTGAGGAAACTAAAATGCCAGGAACCTCAGATCAAGGACAAATCAATGGAGCAATGACTAATATGGGAGATACAGAACTTAGCTTCAGCGGAAGTGGTGGAGATGACATTGATGGTCTGGACATCAAAGTTCTTGACAATGATGCCAACTTTTTCTCACCTGTGAGCTTTGATTCATATAATCTTGGGGGTCTTAACGACTTCCCAAGACCAGCATACGAAGTTTCTGAAAGCTCATTCTCTTTCTTTCCTTCAATGTCCAGCAATTATGTCCGAAAGTCTGGAGAAAACTTGAATTCCAGGTAGCTATGGGTGTGGCGGTGGTTATTTATGCATTAATAATCGGCAGCAGATGATTGTCTCCATGTTTGCTCGCCAGTGTAAAAAAATGTCTGGTGGCTGACGTTCTCTATGTATGCAGGTTTATGGTTAGTATAATGATTGATATTCTGTTCAACAAATGCTACTTAATCCACTTCAGTAGCATTTTTCAAGTTCTGATTTTCATTTTTCTCTGGGTTCTTTTTCATTTTGTTGCCTTTGTTTCGCACCAGAACAATAACAAAAGGACAACCTGAAGGTCGAGGCAAGCTGTTTTGACCACTGATGTTGAGAAACATAGACGTATTGATGATGTTCTACACCTTTTT

>TRINITY_DN21149_c3_g1_i12_5
CTCCCATCCCTACAACCTCGCTTGCTAGCTACACCAACCCTTCTCTCTCTCTCTCTCTCTCTCTCTCTCTCTCTCTCTCTCTCTGGTAGCCATGGGTGAGATGGAGAGGTGCCGTTTGATGCATGGGACAGTGATGCCCGCCGGCAGCTCCCTCGGGGCAACGGTGGCGGCGGCGGAGTCCGACGACCTGAGAAAGGGACCATGGACGGCCGACGAGGACCTCGTGCTCGTCAACTACATCTCCCTCCACGGCGAAGGCCGCTGGAACTCCCTCGCCCGCGCTGCCGGCCTGAAGCGGTCCGGCAAAAGCTGCCGTCTCCGGTGGCTCAACTACCTCTGCCCGGACGTCCGGCGGGGCAACATCACCCCGCAGGAGCAGCTCCTCATCCTCCAACTCCACGCACGGTGGGGCAACAGGTGGTCCAAGATCGCGCAGCACCTGCCGGGAAGGACCGACAACGAGATCAAGAACTACTGGCGGACCAGAGTGCAGAAGCACGCCAAGCAGCTCAAGTGCGACGTCAACAGCCAGCAGTTCAGGGACG

>TRINITY_DN21434_c0_g2_i1_4
CCCCACATGATAACAGTAATTACCCATCCTCTTCTCTCCTGTCTCAGCCTGATCTATAGCTGTCTCTTTCCTCGGGTCTCTTTCGTCTTCTCTACTTAATTCCCTTTGCAGCATATCGATCCTCTCATGTCAAGGCAGAAAGGAACTGGATCGACTAATTAATTGAAGCAGCTAGTGCATGGTACTACGTATACGTACTATTAGTAGTACTGCCACAACTGGCCGTAACACTACTGCAATGGAGAGTACTACTACCAACGGCAGTAGCAGCTGCACTGCCGAGGACGCTGCTGGCCCGATAGAGCTGCTGAGGAAGGGTCCCTGGACGGCGGAGGAGGATCTCTTGCTCGTCAACTACATCGCCGTCCACGGCGACGGCCGGTGGAACTCCCTCGCCCGTTCCGCCGGCCTGAAGCGGTCCGGCAAAAGCTGCCGGCTCCGGTGGCTCAACTACCTTCGCCCCGACGTCCGGCGAGGCAACTTCTCCCCGGAGGAGCAGCTTCTCATCCTCGAGCTCCACTCCCGCTTGGGCAACAGGTGGTCCAAGATCGCGCAGCACCTGCCGGGGAGGACCGACAACGAGATCAAGAACTACTGGCGAACGCGGGTGCAGAAGCACGCCAAGCAGCTCAAGTGCGACGTCAACAGCCAGCAGTTCAGGGACGCCGTCCGCCACCTCTGGATTCCCCACCTCATCGAGAAGATCCAGGCAGCCTCCGTAATTAACTCCGCGGCCGCCTCCTACGGCGAGGCCGCCTCCGTCTCTGTCAGCCCCGCCGACGTCGCCGACACATACGTCGTCCCGACAGGGACAGCAGAACCAGTGCACAGCATGGTCGGCAGTGTCGTCCATGCGACCAAGCTGATCAGCCCGGACAACACGAGCGCGGCCGCGACGCCTTCCGAATCGTCCGGCGACACTAGTTGGTGGTCGTCGCCTTCTGTCTCCGACGTCCTTAACGACTGCTACCTCGTCCAGGCAAGCAGCGGCAGTGACGAGCTCCCCGCCGGCCAGATAAACGGCAGCGTCGACGGCGGTTGCTCGGAGTCGCTGCTGATGAGCCCCTGTGAATATTACCTCCGGCAGGGGTTCCCGGACTTTGCGCAGAACGTGTGGGGGGCGGACGGTGTTATCTCGGCGGAGAACTTCTGGACCGACGAGGATATCTGGTTCTTGCAACACAGCAGCTATAATTACTGAGGCTGCCGTACGTCTCAGGCGAATTCACAACAAGATGGAGAAGAACGAGGTTGTTGGAGGCCATTGTTACAATGTAGAGGAAGAGAGAGAGAGAGATAAAATTATAAAAATCTGGAATATATATATGGCAGAGAACAGAAAAGAAGAAGAACTCTGAAGAACTAGATGTTCTTTTCAACGGAGACCAAATCTAAAAGGGGAATTTTTGTGCTCCTCCATATCGACCTAGC

>TRINITY_DN24845_c1_g1_i3_2
CTTGGAAAGTTGGGTGACACATTATCTTTTCAAATGTATATAGTTTTGCTCTGTCAGAAACTAACATCATATTATTCCTTAAAACAGGTTGTTTTCTGTTCTATTTCCTCTTGAAATGACATTTTCTTTTTGCTGACATAGGATTATGAATCTGCAAATTTCAGTTCAGATACATTATGAAGAATCCAAGACTATGGATGTTGTTTCCTAGAGATGAAGGAAAGGCAGCGCTGGAAATCTGAAGAAGACGCTCTCCTACGTGCATATGTGAAGCAGTATGGACCGCGGGAGTGGAACCTCGTCTCGCAGCGCATGAATGTGCCGTTCCATCGGGATGCCAAGTCCTGCCTGGAAAGGTGGAAGAACTACCTGAAACCTGGGCTCAAGAAAGGTTCTCTAACCGAGGAGGAGCAACGCCTGGTGATCCGCCTGCAAGCCAAGCACGGGAACAAGTGGAAGAAGATTGCAGCTGAGATCCCCGGCCGTACAGCCAAGAGACTTGGCAAGTGGTGGGAAGTGTTCAAGGAGAAGCAACGGCGAGAGTTGGAAGAGAATAACCGAGCCATTGTGGAACCCATTGACCAGGCGAAGTACGACCAGATTCTTGAGAACTTTGCTGAGAAACTTGTGAGAGAGCGACAGCCTTCGCCATTTTTCATGGCCGGGCCGCTCCTTCCGCCATGGCTCTCAACTTCCAGCAGCCCAAGTTCGATAGGGCCACCCTCTCCCTCTGTCACTCTGAGTCTCTCACCCACCACATTACCCACAGAGCCCGTCAAGTGGCTTCAGCCAGAAAGAGCTGCAGATAATAGCTCAGTCACAGCTGCTTCCCAACCATCTGTTTCCTCTGGAGCTCCACCAGTTGACATGCGGATTGTCTCAGAGCTCATCGAGTGTTGCAGAGAGCTGGAAGAAGGGCATCGAGCATGGCTGGCACACAGGAAGGAGAATGCATGGAGGCTGAAGAGGGTGGAGCTGCAGCTGGAGTCAGAGAAGGCTAGCAGGAGGCGAGAAAAGGCCGAGGAGATCGAGGCCAAGGTCAGGGCGCTGAAGGAGGAGCAGAGGCTGATGCTGGAGAGGATCGAAGCAGAGTACAGGGAACAGCTACTGGTTCTGAGGAGGGATGCAGAGGCCAAGGAGCAGAAGCTGGCTGAGCAGTGGGCTGCAAAGCACCTCCGACTGACCAAGTTTCTTGAGCAGATGGGCTGTCGGTAATGGCCCTGTGCTGATGCAAATGGACGCTGAGATGTGTGCTTTTTTGGGGTTCCTCTTCCATTGTTGAGATGCTTCTTGCCTTTCCCTACACTCTTGCAGCCTTGCTAGACCATTTGCCAATGCCCACATATTAATGTCAATGCAGGAGTTATCTTCTAATATTTCTTCCGTACATCTCTAAT

>TRINITY_DN27220_c0_g1_i1_3
CTTCGGGGCCCTTCCTCCCCCGGCCGCCACCTCCTCCGCCGTTCCCCGGAGGGCCTCCCTGCTTGAAACGTGTGCGCCGATCCGATCCGATCCCATGGCCGCGGGTGCCGGCAATACAACATCGCCGGCGACGGCGACGGCGATGGCGGGGAGGAAGGATGTTGATAGGATAAAGGGGCCGTGGAGCCCGGAGGAGGACGAGGGGCTGAGGAAGCTGGTGCAGAAGCACGGGGCGCGCAACTGGTCGTTGATCAGCAAGTCGATCCCCGGGCGGTCGGGGAAGTCGTGCCGTCTGCGGTGGTGCAACCAGCTGTCGCCGCAGGTGGAGCACCGCCCCTTCACCGCCGAGGAGGACGAGAGGATCCTCCGCGCCCACGCCAAGTTCGGCAACAAATGGGCGACCATCGCCCGCCTCCTCAGCGGCCGCACCGACAACGCCATCAAGAACCACTGGAACTCCACCCTCAAGCGCAAGTATCCTGCGTCCTTCTCCGACGACGCGATCGGCGCCGACGAGGCGGCGG

>TRINITY_DN27904_c1_g1_i2_4
CCCAACCCTTCCAGTTCTTCTTCCCATCAAAAGCTTCTGGAACCCTAGGAAACCAGAGGAAGAAGAGGCAAAAGCTTCCATCTTTATCCGTGTGATCGGGCTCTAAGGGGGGCTCTGGTTAAGGTTTGGATCGTCTGGGGTAAGAGGTGGGTGGGGGTTGTGGGTCGCATGCAGCAGCAGTCCATGGCGGCTGGGTCGATCAATCCCGCGGCGGCGTCCGGCCGCAAGGACGTGGACCGGATCAAGGGGCCGTGGAGTCCCGAGGAGGACGACGCCCTCCAGAAGCTGGTGCAGAAGCACGGCGCCCGCAACTGGTCCCTGATCAGCAAGTCGATCCCCGGGCGGTCGGGCAAGTCGTGCCGGCTCCGGTGGTGCAACCAGCTATCGCCGCAGGTGGAGCACCGGCCATTCTCCCCCGAGGAGGACGACACCATCGTCCGCGCCCACGCCAGGTTCGGCAACAAGTGGGCGACCATCGCGCGCCTCCTCCACGGCCGCACCGACAACGCCGTCAAGAACCACTGGAACTCCACCCTCAAGCGCAAGTACTACGCCGCCGCCGTGGGGCCGTCCGCCTCGGACGAGACGGCCGGCGGTAGGACGGCCCGCGGCGTGGGCGTGGGTGGGGGCCTCGACCGCGACGAAGAGGAGGAGCACAGCAGCGAGCCGCCGCTGAAGAGGACGGCCAGTGGCGGGGCGGCGCCGGTGGGGTTCTGCCACAGCCCCGGCAGCCCGTCGGGGTCGGACGTCAGCGACTCGAGCCAGCACTCCGCGCCGGCGATGCGCGTCTACAGGCCCGTCCCCAGGGCCGTCGGGATCCTACCGCCGCCGGTGGAGGCCTCTTCTTCCATCAACAGCGGAGGCGGCGGCGGCCACAACACGGATGACGAGGACCCCGCCACCTCGCTCACCCTGTCTCTCCCTGGATCGAGCACCTGCGAGTCCTCCAACCCCGTCGTGGCCCCGCACACGCCCCCCTCCGCTGTCGCCACGTCCGTGGAAGCACTGCACCACAGCCTGCATCGTCAAGAACAAGAACGGATGATGCTCCTGCCATTACAGCCGTCGGCCATACGCCCGACCCATCAGCATCAGCAGCACCGGAATCCATCCCCCGCCGGCTGTGCTTTCATGAACGCCGTCGCGGCACCATCCCTGGCCGCCGCGCCCGAGCGGCAGGAGCAGCCGCAGCAGTTCCCATTCAGCGCGGAGTTCCTGGCGGTGATGCAGGAGATGATAAGGAAGGAGGTGCGGAGTTACATGTCGGGCCTCGACCAGAGCGGCATGGCCTTCCTCCAGCACCCGCACCAGCACATGGACGCCATCCGCAACGCCGCCGTGAAGCGCATCGGCATCAGCAAGCTCGAGTAGAGCTCTCCTCTGCTCTGCTCCCAAAAGATCTTTGCTTTAAAAATCAAAAAAAAAAAAAAAAAAAA

>TRINITY_DN28276_c0_g1_i7_2
CTCTCTCTCTCTCTCTCTCTCTCTCTCTCTCTCTCTCTCTCTCTCTCTCTCTTGCAAGTCTCGGCCTTTTTCCGGGGAGATGGGTTTCCAGCAGCCGAGCACAACTGGGGAAATGGGCTTCTTCCCTCCACCGCCGGCGGCGGCGGCGGCGGCTCCGTTCTTGGGCGGCGCAGGTTCTTCTCCCTCTGGGGAGGGAGAATGCAACAGCGGGGATGCTGGGTTCTCGGCGCAAGGCCATGGCTGGGGCTTCCGGCCCTTCGAAGGGGAAGAAAGGCATTTCGGCGGAGTGAAGCGCGGCGCCATGGGGATAGAGGATGGGGAGGAAGAAGAAGAAGGCGAAGAAAGCTTCACCGGAGGAAAGGATCAGGAGGCCGAGCAGTCCAAGCTCTGCGTCCGCGGCCACTGGAGGCCGGCCGAGGACGCCAAGCTAAGGGAGCTCGTCGCCCGCTACGGCCCCCAGAACTGGAACCTCATCGCCGAGAAGCTCGAAGGGAGATCAGGGAAGAGTTGCAGATTGAGGTGGTTTAACCAGTTGGACCCCAGGATCAACAGGAGAGCCTTCGGCGAGGAGGAGGAGGACCGGCTGCTGGCCGCCCACCGGCTCTACGGCAACAAGTGGGCTCTGATCGCCAGGCTCTTCCCCGGCCGGACGGACAACGCCGTCAAGAACCACTGGCATGTGATCATGGCCAGGAAGCACAGGGAGCAGTCCAGCGCCTACCGGCGGCGGAAGGCGGCCGCCGCCTGCACCCCTCCCTCCAACCCGCCCCCGGCGGCGGCCACCGCGAAGCGTGCGGACGTGCACAGCAGCACCACCAACGCGTGCAGCGTCGAGTCCACCGTCACCAGCAACAACAGGGACGAATCCGCCTCCACCGCCACCGACCTCTCCCTCAACTCCCCCTGCAGCCGCCCCGTCGTCCTCCACGGCGCCTA

>TRINITY_DN24201_c0_g1_i4_3
GGGGTGAGGGGTGTTGGCGGTGGCGAGGGGGAAGGGAAGGTGGGCGGCGGAGCCGGGCTGGAGATGCGGTTCAGGGGCGTGCGCAAGAGGCCATGGGGGAGGTACGCGGCGGAGATCAGGGACCCCGGGAAGAAGACCCGGGTCTGGCTCGGCACCTTCGACACCGCCGAGGAGGCTGCCCGGGCCTACGACGTCGCCGCCCGGGACTTCCGCGGCCCCAAGGCTAAGACCAACTTCCCCTGGGTCACCGGGTCGGCGGGGATTGCGCTGCCGGCCGTCGCCGTGCCGCGGCGGGATCACCCGCACGAGTCCGCCGCCCTCCAGGTGAGCAGCCCCGGGAGCCAGAGCAGCACGGTGGAGTCGTCGAGCCGGGGGCCGGCGACGCCGTTCCCTCTCCAAGCGGGGATTAACCCAGGCATCTCCGACCCTCCCTATGTTATGGGCGTCGCCGCCGTCCCCCACCCCTACCCCCACGCGATCGCCGGCGGCGGCGGATGCTTCCCCTACCACGCCTACCCTTCGCTGGCCCAGGCCTACGCCGGCGCCGCAGAGGCCGCGCGGGCGCGGCTCTACTTCTTCGACGCCTTCTCCGCGGCGGAGAAGACGGCGTCCACGGCCGGATCCCTGGCGCCGGAGGCCGCCGGCGCGACCCCCGTTGGAAAGTGGTACCAGGCCACCACCGGCCCCACCCGCAGCTGCGGCGGCGGCGCGGCGCAGAGCGACTCGGACTCGTCGTCGGTGGTAGTGGACCTGCACCCCCGCCCGCGGGGACTCGGCCTCGATCTGGACCTGAACCAGCCGCCCCCAGCGGAGATCGTCGAATCCTAGCCGTCCAATTTTGCAGCAGATGGGGACTGGTCGACGGCGACGTCACCTCCCGCGATTTGACGAATGGGGGTGGGTTCTCGGCGGGCACACGGCATGTAAATATATGTGTCTGGAGCAAGAGAACCCTCCTCTCTCATTATTCCGCTTCCTGACCACTTTTTTAGAGTTTCTATCTCAAAATTTTTCCCACTTTTTTTTTGCACCGTAAAATCACATCCGCCTCTGGCGGATCCGATGATCGTCCGGTGTTGGGTGTAATCTTATAATTTGTTAATTTGCTCGAACCATCTACTAGTGATCATCAATTCGAAAG

>TRINITY_DN24247_c0_g1_i2_5
CCCGCCTTCCTCCCTTCTCCATCGCCCTCTGGTTTGCGCTCTTTCTTCTTCTTCTTCGATCCTTGTTGGAGTTTTCCGGCGATCGGTGTATCAGCTCTAGAACCGTGTTAATGGCGCCCAAGGCCCAGAAGACCTGCGCCCGTAGTGGCGGCGGCGACGTCGACTTCGGGGACTTCCACTTCAGGGGCGTGCGCAAGAGGCCGTGGGGGAGGTACGCGGCGGAGATCAGGGACCCCGTCAAGAAGACGCGCGTCTGGCTCGGCACCTTCGACACGGCCGAGGAGGCCGCCCGCGCCTACGACGCCGCAGCCCGCGACTTCCGCGGGTCCAGGGCCAAGACCAACTTCCCCTGGCCCGCCG

>TRINITY_DN25439_c3_g1_i5_4
GGGGGATCCGGCAGCGGCCGTGGGGGAAGTGGGCGGCGGAGATCAGGGACCCCACGAAGGGCGTCCGCGTCTGGCTGGGCACCTTCGACACCCCCGAGCAGGCCGCCCGCGCCTACGACCGGGAGGCTCGCCGCATCCGCGGCCACAAGGCCAAGGTCAACTTCCCCAACGAGGACGACGACTGCCGCGGCGGCGACGGGCCCCCGCGCCACCGCCACCACGCGGAGCAGGCCGTCTTCGCCTCCCCGCCGAACCCTGCTCCCCTGCCCTCCGCGTCGTCAGCCCCTGCTTCCAGCCTCCCCATTGCGCCGGAGCCTGTTGCGGCGGCGGTGGAGGCGCAGGAGGAGGCCAGGAGGGAGGCGGCCGCGGCCGGCGAGGCGAGGAGGCTCTCGGAGGAGTTGCAGGCCTTCGAGACGTACATGAAGTTCCTGGAGATCCCATACATGGAGGGCGGCGTCGTCGCCGGTGACGTCGTTGCCGCAGCAGAGCAGCCGGCGTCGGCTCTGGAGCTGAACCTGAACTGCAACCCGTACATGGAGGAGGCCCCTCTGGAGCTCTGGAGCTTCGATGACAGCCTCCCCGTCGCCATTTGATCTCTCTCTCTCTCTCTCTCTCTCTCTCTCTCTCTCTCTCTCTCTCTCTCTCTCTCTGCATTGTGTAAAAAAAAAAATTAACATATGTTATGTAATGCCGCTAATTACTAAGTTAGGTAGGTTTTTCTCCCTTTTCTTTCTATTAGAAAAGGGATTTTTATTTTATTTTGTTTTGGTTTTTTTCCCTGTTGTATGGAGCCCAAGCTCTGGTTTTTGTTAACTTGATCAGGTGGATGTAATGACTAATGAGGGTGTGCCTCCTGGATTCCTT

>TRINITY_DN26535_c1_g3_i1_1
ACCTCGCAGCTTCCGTTCTCTTCCTCTTCCTCTGCATTTGTAGACCAGATCCTTCTCTCTCTCTCTCTCTCGCTCGCAATTTGTGGATGGTCATGGCGCCGAGGGGGGCCGAGAAGATCTGCGCCCATGGTGGGGTGAGGGGTGTTGGCGGTGGCGAGGGGGAAGGGAAGGTGGGCGGCGGAGCCGGGCTGGAGATGCGGTTCAGGGGCGTGCGCAAGAGGCCATGGGGGAGGTACGCCGCGGAGATCAGGGACCCCGGGAAGAAGACCCGGGTCTGGCTCGGCACCTTCGACACCGCCGAGGAGGCTGCCCGGGCCTACGACGTCGCCGCCCGGGACTTCCGCGGCCCCAAGGCTAAGACCAACTTCCCCTGGGTCACCGGGTCGGCGGGGATTG

>TRINITY_DN27758_c0_g3_i2_5
AGAAGGCTCGCGTCTGGCTCGGCACCTACGACTCCGCCGAGGACGCCGCCCGCGCCTACGACGCCGCCGCCCGCGCCCTCCGCGGCCCCAAGGCCAAGACCAACTTCCCCCTCGCGCCCGGCGCGCCCCCGCCCGCCGCCCTGTCCCCTCTCGGCTTCTTCCCCCGTCCCCACAACCAGCAGCGGCGGCCGCAGGCGTCTCCGCCGGATCAGCCGCCTCGGCCGGCGTCCAGCAGCCATAGCAGCACGGTGGAGTCCTCCGTCGGGCCCCGGGGCTCCGCCGCCGCGCCGCAGCCGGCCCCGCGACCCAGGATGCCGGCGATCCAGAAGCGGCCCTCCTTTACCCCCGACGATTGCCACAGCGACTGCGGCTCCTCGTGCTCCGTCGTGGACGACGCGGAGGACGCCGCTTCCTTCCCTCGGCCTCCGTTGCACGACCTGCCGCTGCCCTTCGATCTCAACCTGCCGCCGGCCGACATCGACTGCCACCAGTACACGGCCCTCGCCCTCCTCTGAGGGCACCGACGGATCGGACCTGATCGGATCGAAACACCAGAGGAAACGGAGAGAACGCGGTGGCTATCATCCTCTTCCCCACTTTTTTTGTTGTTGTCCGTTGTTGTTTTTGCCGCCATCGCCATATTATACTACGGCGGATCCACCCGAGCGCCATGGAGGGGTATTCCCTTCGGCTCTTGTTAGATCTTTACACTTTTTTTCTTCTTCTGTTCTTCTTTCTTTCTTTGTTTCTGCTTTAACCCATATGTACAAAAAAGACATCTTTAATCAATTCTACGGCTAGAAAGGAAG

>TRINITY_DN27911_c1_g3_i3_1
TATCATCTACTTTTTACTATCTGTGTGTTGGTAGTATGATATTATGTCTCCTCCATGCCACCGTGGCATCAAGGATGTCTGTGTCTGTTATTTCTTAGTTATTTTGCTTAGCATCTTTTCTTCCCGGTGATTCATCAAGGTGTTGTTAAACTTAAGGATAGCTCACCCAATTGTATGGCTGGTAAAATAGGATCATGGATAATTGTTTCTACATTTGAGAAACTTTGTGGCAGTTCTATTTTTAACTTGTATCCATATCCATGGCAGCAGGAAATTTGACTGTAAAAGCTGCTGAATTCAGTGGCCTGGCTGAGAAATCTGCAAAGAGAAAGAGAAAGAATCAGTACAGGGGAATCCGACAGCGTCCTTGGGGCAAATGGGCTGCTGAGATCAGAGACCCAAGGAAGGGAGTCCGAGTGTGGTTAGGTACCTTCAACACTGCTGAAGAAGCTGCAAGAGCCTACGACGCAGAGGCCCGCAGGATTCGTGGAAAGAAGGCAAAACTGAACTTCCCTGATGAGGCAGCTAGTGGTCAGAAATGCGGCCTGAAGGAAACTGCACTGAAGGCTTCAAAACCTAATCCAACTGAGAAACTGAGTGCCAGTCAGGGTTTTGATTATACAAGTGGTCTCACTGGTCTGGAACATGAATTGTACTCTAGCTTTGGTTTCATTGAGGAGAAGGAACTGAAACAACAGGAACATCTGAATGCCCTCCCTGCCTCTAACCCCGTGGCACAAGGTAATGGGTTGTGTTTCCAGTCTGATCAAGGTAGTAATTCTTTTGGTTCCGAGTATGGTTGGGAGCATGAATCCAAGACTCAAGAGATCACTTCGACATTTCTTCCCACCATAACAGAATGTTATAAGCCAGCCATTGTAGAAGATGGGAACCCACAGAAGAAGCTGAAGAACAATGATGAGGAAGCAGTGGCCACCGATGAGAATGCGTCAATGAAACTCTCTGAAGAGTTATTGGCTTTCGAGTCTTACATGAAGTTCCTGGAGATCCCATATCTGGAGGGGAGTCCTGAGGAGTCAATAGAGAATTTCCTTGGCAGTGATGTCACTCAGGATGGGAAGAACCCAGTCGACCTGTGGAGCTTTGATGACATGCCCCTGTCTGATAATATCTTCTGATGGCCGTTTAGCGGAAGTTGCTGTCTTCTGTAAATAAAGTAGAAATGGACATGACCCATGGGTCGTGATGC

>TRINITY_DN27944_c0_g1_i1_5
CCTGTGGCCCCGCCGCCGCTCCCAGCTCTCGCACCACCACCACCACGAGGAACCCGCAGACGTTTTCCCCGACGGCCTCGTCGACGCACTCGCCTTTGTCGCAGGTGAAGCCCCCGTCGAGAAAGTGGGGAAGAAGAAGAGGGAGCGGAAGAACCTGTACAGGGGGATCCGGCAGCGGCCGTGGGGGAAGTGGGCGGCGGAGATCAGAGACCCCACGAAGGGCGTCCGCGTCTGGCTCGGCACCTTCGACACCCCCGAGCTCGCCGCCCGCGCCTACGACCGGGAAGCCCGCCGCATCCGCGGCCACAAGGCCAAGGTCAACTTCCCCAACGAGGACGACGACTGCC

>TRINITY_DN28023_c0_g1_i3_1
CTCCGCCGCAGCAGCAGCAGCAGCTGGAGAAGAAGAAGAAGAAGAAGAAGTGGGTCGTCGTCGCTGCTCCAGTGATCAGGCCACTGCGTCATGGAGAAGTGGACCAGCTGCTACCAGTCGGACTCACTGCCGCCGGCGACAGAGCTGGACTCGCTGCTGCGGTCGGCGGCGTCGGACGAGGAGGCGTACGCCACGGTGTCGTCGGCGCCGCCGAAGAAGCGGGCGGGGCGCACCAAGTTCCGGGAGACGCGGCACCCGGTGTACAGGGGGGTGCGGCGGCGCAGCGCCGGGAAGTGGGTGTGCGAGGTGCGGGAGCCCAACAAGAAGTCCCGCATCTGGCTGGGCACCTTCCCCACGCCGGAGATGGCGGCGCGCGCCCACGACGTGGCGGCTATGGCGCTACGGGGGCGCTCCGCGTGCCTCAACTTCGCCGACTCGGCCTGGCTGCTCCCCGCGCTCCCCCACTCCGCCACCGCCCGGGAGATCCAGCAGGCCGCCGCCCAGGCCGTGGAGAGGTTCCGCCCGCTTGAGGAGGCCGCGCAGTCCGAAGATGTCGACGGGCGGGTGGATGCGGTCGAGACGCCTCCCGCGACTGTAACAACACAGTCGTCGGCGGCGGCGGCGGTAGGGGAATGCGGCGGGTACTACGGCGCGGACGGGGACTTGTCCTACGTGGAGATGCTGGGCAACATGGGGATGATGTCGCCGCAGCACGTCCAGATGCAGCAGTGGCCGGGGCAATGCGGCGGCGGCGGCTACTGGGACGACGCCATGGAGACAGAGCACGACATGTCACTCTGGAGCTACACCTTCTGACTGAGAGCACCGCCGGACCAGTACTGGCGTGGCGGCCGCCCGCCCGTTTACCTGCTTAGCCGCAGCAGCAACCTCCGGCAGCCGCCATTTCCTAGCTAATCTGTACAGAACTACTACTAGTAGCAGCGGCAGCGAGAGGTACCAGCGTACGTATAACCAGTACCCCCTTTTTTGGTACTTTGGCACAAACTATAAAAAGCACCGTACCTACAACTCTCTCTCTTTCTCTCTCTCTCTCTCTCTCTGCAAGAGGACAGAGACCAGGCGAAGCCCATTTGTAACCAGAACCGTTTGAATCGGAGGGGGGAAAGAAGAAAGGGAGATATCAAAGTGTTCCCTATCTTTAGTTCCCTGATCCGGGTTTGTACTAAATTTTCAGTAATTGTGTGGCAAAGAGTGAGAAGTACTTTCC

>TRINITY_DN28739_c1_g3_i4_2
GGGGATCCGGCAGCGGCCGTGGGGGAAGTGGGCGGCGGAGATCAGGGACCCCACGAAGGGCGTCCGCGTCTGGCTGGGCACCTTCGACACCCCCGAGGCGGCCGCCCGCGCCTACGACCGGGAGGCCCGCCGCATCCGCGGCCACAAGGCCAAGGTCAACTTCCCCAACGAGGACGACGACTGCCATCGCCGCCGCGACGAGCCCCCGCGCCACCGCCACCACGCAGACCAGGTCGCCTTCGCGTCCCCGCCGAACCCAGCTCCTCTCCCCTCCGCGTCGTCAGCCCCTGTTGCGGCGGTGGCGGCGCAGGAGGAGGCAAGGAGGGAGGCGGCTGCCGCCGGCGAGGCGAGGAGGCTCTCGGAGGAGCTGCAGGCCTTCGAGACGTACATGAAGTTCCTGGAGATCCCTTACATGGAGGGCGGTGACGTCGTTGCCGCAGCAGAGCAGCCGGCGTCGGCTCTGGAGCTGAACCTGAACTGCAACCCGTACATGGAGGAGGCCCCTCTGGAGCTCTGGAGCTTCGAAGACAGCCTCCCCGTCGCCATTTGATCTCTCTCTCTCTCTCTCTCTCTCTCTCTCTCTCTCTCTCTCTCTCTCTCTCTCTCTCTCTCTGCATTGT

>TRINITY_DN30004_c1_g2_i2_5
GTGCGCAAGAGGCCGTGGGGGAGGTACGCGGCGGAGATCAGGGACCCCGTCAAGAAGACGCGCGTCTGGCTCGGCACCTTCGACACGGCCGAGGAGGCCGCCCGCGCCTACGACGCCGCCGCCCGCGACTTCCGCGGATCCAGGGCCAAGACCAACTTCCCCTGGCCCGCCGACGTCGTCCCCACCACCGCCGACCCCGGCTGCCACCGGCAGGAGCTGCAGCGGGGAGGGAGCCCCAGCAGCCAGACCAGCACCGTGGAGTCGTCGAGCCGCGGGGGGCCGCCGGCGCTTCCGTTTCATCCGGAGGGCGTCACCCTCGCGTACCCCCCGCCGATCCTGGATCTGGAGCTCCGTTACCCTTCCCAGGGCCAGACCGTCGGCGGCGTGGCCGCCGGCGGCAAAGTCCGCTTCCCCTTCCAGAGCTTTCCCATGGTGCCACCGTCCTATCTTGCCGCAGCAGCGGCGGCGGCGGCTGAGCCGCGTCCCAGAACCCTGTTCTTCCTCGACGCCTTCGCAAGATCTGCGGAGAAGCCTGCCACCGCCGACCCCCGGCCGATGCTGGGCGCCGTGCCCGGAGTGGACTACTGCCAGGCCGCCCCCGTCAGCAGCGGCGGGGCGCAGAGCGATTCGGATTCGTCGTCGGTAGTGGTGGACCTTCAACCACGGCGGCGCGGGGTGGGCCTCGATCTGGACCTGAACCTTCCCCCGGCATCGGAGGCCGTCGAATGCTGAACAGCCCCCCGATCCAGACGGATCTGAGAGGAGCAAAAGCTAGCTTTTT

>TRINITY_DN30469_c0_g1_i3_4
CAGCTGTGCTAGCTCCGCTCTCCTCCTCTTCAGCTTCCCCCACCTCGGCTCCCACTGCCGGACCAGCTCCTCTGCCTCACCGCCGCCGGGGAAGAAGAAGCTGATTAGCCCGCCGGAGATGGCGAGGGGGGCAAACACTGCCGACGCCGCCGCCGCCGGCGAGGCCGAGGCCGAGGAGAGGAGGAACACTAGGTACAAGGGCGTCCGGAAGAGAAAGTGGGGCAAGTGGGTGTCGGAGATTCGGCTGCCCAACAGCCGAGAGCGCATCTGGCTCGGCTCCTACGACACCCCGGAGAAGGCGGCGCACGCCTTCGACGCCGCCATGCTCTGCCTCCGCGGCCCCGGCGCCGGCCGCTTCAATTTCCCCGACGTCCTCCCCGACGTCGCGGACGCCGGCGTGCGAGCCTTCTCGCCGTCGGAGGTCCAGGCTGCCGCCGCCAGGTTCGCCAACAAGGCCCCCGCTGTGGCGCCGCCGCCGCCGCCCAGACCCGACCAGCTGCCCCAGCTGAGCTCCCCCGAGGTGGCGGCGTCGGAAGGGACGACGTCGAAGACGACGGCGGAGAGCGGGGGGGAGCTGGACTGGTCGTTCCTCCTGGACTCGGCGGGGCCGTCAACTTCACACTCCGGGCCGGACTTCATGGGGATGTTCGACTTCAACGACTTCCCCAGCGACTACTTCCCGGTGCCGGTGACGACGATGACGGCGGCGGCGGCGACGGCTCCGGCGGATCACTTCATGGATGATGGTGGTGGTGCAGCGGAGTTCGATCAGTCCTCGTTTCTGTGGCATTTCTAGACGAAGTATATGATCAGGCATCAGCACATGAGTTTTAAGACAGCTGCTGCCGGCACTATCAAAATTCATACTACGTACTGAAGATATATACTACCACAAGGAGGAGGGTTATAATTAAGTTTGCTGCCCGGTTTTTTTAAAAAGGGGC

>TRINITY_DN31070_c0_g1_i6_5
CTCTCGGCGCGTCTGTGTGTTGCGCACACCCACCTCTCCTCCCAACTAGAACTATAAAGAGAGGCCGGCCGGCTTTCCACCGTTTCTCACATCTCTGCTCGACCAAAAGCTTCAATCTTTCCCCACAGAACCCAAAAGGAAGAGCTTTCCTCACCCGCACAAAACCCCCCTTTGGTCTTCTCTCTGAGCTCGCCGGGATGTGTGGGGGTGCGATCATCTCCAACTTAATACCGACCGCCAAGTCGAGGTTGGTCACCGCCGATTACCTCTGGCCCAACCTGAAAAGGGGGGCGCTGCAGGGCTCCAAGCTCAGGAAGAAGAAGGAGGTGTCCGAGGAGGACTTCGAGGCCGACTTCCGGGAATTCGAGGACGACTCCGAGGAGGACGATGAGGAGGACGAGATCCTTGATGTAAAGCCCTTCTCCTTCACGGCCAAGGCCCCCTTCACCCAAGAATTCAATGGACTTGCTGACAAAGCTGCAAAGATGAAGAGAAGGAATCACTACAGGGGAATCCGCCAGCGCCCATGGGGCAAGTGGGCAGCTGAGATCAGAGACCCGAGGAAGGGAGTTCGTGTCTGGCTTGGCACATTCGGCACTGCAGAAGAAGCTGCGAGAGCCTACGATTCCGAGGCCCGCAGAATTCGCGGGCAGAAAGCCAAGGTGAACTTCCCTGAAGGGGCAGCCGGTGCTGGCCGGAAACGTACCAGGAAGGCGGCTGCCACAAAGATTCCCCGAGACATGAATGCCAACCAGAATGCCAGTCATAATTTCATCGAGGAGAAAGAACCTGCCGAGCAGTCTCAATACAAACATTCTCTTCCTGTGGACAAGGCTGTCACCCCACCTGCAAATGGGGGTGGGCTCTCTTACCACTCGGATCAAATTAGCAACTCTTTCGATTGTTCAGACCTTGGTTGGGGCCATGAATCCAATACTGCAGAAATCTCGTCGGTCGTTGATCCCAAGATGGAAGTAACTGAAATGTGCAACATGGAAGATGGCAATCCACAGAAGACGCTGAAGAACAATGTTGGAACTGCAGTTCCAATGGAAGAGAACACAACAATGAAATTCTCCGAAGATCTTCCATATTTTGAGTACATGGACCTTTTCCAGATGCCATATGTGTATGGTGATTCAGTGGCGAGCCTCTTCAATGTCGACGAGACGCAGGA

>TRINITY_DN31297_c1_g1_i3_4
CGGGAATTCGAGGACGACTCCGAGGAGGACGATGAGGAGGACGAGATCCTTGATGTTAAGCCCTTCTCCTTCACGGCCAAGGCCTCCTTCACCCAAGAATTCAATGGACTTGCTGACAAAGCTGCAAAGATGAAGAGAAGAAACCACTACAGGGGAATCCGCCAGCGGCCATGGGGCAAGTGGGCTGCTGAGATCAGAGACCCGAGGAAGGGAGTTCGTGTCTGGCTTGGCACATTCGGCACTGCGGAAGAAGCTGCGAGAGCCTACGATTCAGAGGCCCGCAGAATTCGTGGGCAGAAAGCCAAGGTGAACTTCCCTGAAGGGGCAGCTGGTGCTGGCCGGAAACGTACCAGGAAGGCGGTTGCCACAAGGATTCCCGAAGACATGAATGCCAACCAGAACACCAGTCATAATTTCATCAAGGAGAAAGAACCCGCCGATCAGTCTCAATACAAAAATTCTCTTCCTGTGGACAAGGCTGTCACCCCACCTGCAAATGGGGGTGGGTTCTCTTACCACTCGGATCAAATTAGCAACTCTTTCGATTGTTCAGACCTTGGTTGGGGCCACGAATCCAAAACTGCAGAAATCACGTCAGTCGTTGATCCTAAGATGGAAGTCACTGAAATGTGCAACATGGAAGATGGCAATCCACAGAAGAAGCTGAAGAACAATGTTGGAACTGCAGTTCCAATGGAAGAGAACACAGCAATGAAATTCTCCGAAGATCTTTCGTATTTTGAGTACATGGACCTTTTCCAGATGCCATATGTGTATGGTGATTCAGTGGAGAGCCTCTTCAATGTCGACGAGACGCAGGAATGCGATGACCCGATCGGTCTGTGGAGCTTCGACGACACACCACTGACATTCCCTGTTTTCTGAGGGCGCTTAGCTCTGGCTACTGCTTTCGTGTGTTGAAAAAAAAAAATTGTAAGACATTTACTACTCATCTTCCGTTGCTAATTAACAATAAAACTAGGGGATTCTTTTATTCTTGTGTCTAGCAACAAGTCTCCCCAGTCCAGTCCACTGTGTCTTGTTGAAGAGGGAGTTTGTCTTTCTTTTTTTGCCTGGTGCAGGAAGATGTTTGTTGATGCAAGGAAATGAAGAGCGATTGGAATGCCAGGCCTGTTGAACTTGCCATGCCTCCCCATATGCAATTACTACTACTATTACTCATAGTAGGTTTTGTTACCAGCATATTGGTCTCCTTTTATGTTGTCTTGTGTAGTCTGTATAGAGTCTTGCACCTGGCAGATCAG

>TRINITY_DN23968_c1_g1_i3_2
GAGGAAGAGGAGGATGGGGACGGGGGCAGGGAGGGGCTTCTGGAGCGAGGAGGACAGGGCGATGGCGATGGCCGTGCTGGGCGCCCAAGCGTTTGACTACATCACCACCACCCATGTGTCCTCGGAGGGCCTCACGGCCGTCGGCGGCGACCCCGACCTCCAGAATAAGCTGGCGGCGCTAGTGGACGGGCCTAACCGGTCCGGTCTCACCTGGAACTACGCCATCTTCTGGCAGGTCTCCAGGTCCAAGGCCGGCGACGTCGTGCTCGGCTGGGGCGACGGCCACTGCCGGGAGCCCCGGGAGGGGGAGGATCCCTACCACTACGGCGGCGGCGGCCACCGCCACCACCACCACCACGACGAGGCCCACCAGAACATGAGGAAGCGGGTGCTGCAGAAGCTGAACACCTTCTTCGGCGGCTCCGACGACGAGAACTTCGCCCTCCGCCTCGACCGCGTCACGGACACCGAGATGTTCTTCCTGGCGTCAATGTACTTCTCCTTCCCCCAGGGGAAGGGCGCCCCCGGCAGGGCGTTCGCCTCCGGCAAGCACCTCTGGATCTCCGACGCCGCGCCCAAGCCCTCCTCCGACTACTGCGTCCGCGCCTTCCTCTCCCGATCCACCGGCACGCGCACGGTGGTGCTCGTGCCGTCGGAGGCGGGGGTCCTCGAATTGGGGTCGGTGAGCTCGGTGCCGGAGAACGCGGAGATCCTGCAGATGATCAAGTCCATGCTGAAGCCCTCCCCATCCGCGTCCTCCGGCGGGGAGAAGAGGGAAGGCAGCGTCGGCGGCGCCGCCCACCCCGCGGCGTTGGCGCTGGGAGGGAAGCAAGCAGAGGAACGCCCAAGGATCTTTGGGAAGGATCTGAATCTGGCGAGGAGTACTCAGACCAGTGAGAAGATTACGGTGCCCAAGGTGGAGGAGCCACCGTGGGATCTGCAGAGCAGCAATGGCGCCGCCAGCCACTTGCCCTTCCCCAACATCAGGAAGAACCCCCATCTTCTGAATTGGAACCAGGCCCGCCATGTGAACGGAAACCAGACCCTGATCAACAGCAACCACCAGAAGTTTGGCAACGGGATCGTGATCAGCGGAGGCGCCGACGGGGTTAGGGGCGGCGCCGACGCAGACGCATCTCACAGATTGTTTGGCCACCAGAGCAACGGGATCAGGGAGGAACCCCGGATCAGCGGCTTCCAAGCACAGAAGCAGCAACCGCCGCCGCCCCCAAGGCAGATCGATTTCTCCGGGACAGCTGCAACTTCCAGGACGGCCGGCGGCCCTGTTGCGGCGAAGCTCAACAATCTGGATTCCGAGCACTCTGATGCGGAGATCTCCTGCAAGGAAGACCGGCCGGCCGCGGTGGAGGAGAGGCGACCCAGGAAGAGAGGCCGGAAGCCTGCAAACGGGAGGGAGGAGCCCCTCAACCACGTCGAAGCCGAGCGCCAGAGAAGGGAGAAGCTTAACCAGAGGTTCTACGCCCTGAGGGCCGTGGTGCCCAACATATCCAAGATGGACAAGGCCTCCCTTCTCGGCGACGCCATCGCCTACATCACCGAGCTGCAGAAGAAGCTCAAGGAGATGGAATCGGAGAGGGAGAGGTTTCCTCCTGAGACGCCGGCGGCGGCGGCGGAGCACAAGACGCCGGCGGCGGCGGACTGCGCCGACATCGACGTCCAGACTGTG

>TRINITY_DN27913_c1_g1_i3_2
GAACAGAGAGAGTAGCTAGCTCTCTGTGGAAGCTCATCATGCTTCGATAAGAACAGCTCGGATCGATCTCGGCCGGGGAAGCCAATGACGGAGATGGAGCCGTTCCCCTTCTTCTACCAGGAGGATGCAACGGCGTCGGCGGCGGCGGCGGCGGCGACGACGTCGGCGGCCATGGAGGAGCTCTTGTTCCCTTGGGACAGCTTCCGAGGCCTTTCCGATGTCGTCTCCCCCGCCTTCGACCTCTCGCCGTTGCCGTCGGAGGAGGACGTGCTCCTGTGGGGTGGCGACTACGACCTGCCCGCTTCCCTGATCAGCTTGCCACCTCCTCTTCCTCCTCCGCCGCCGCCGCCGCCGGTGACTGCTCCTGAAGTGGAGATGTTGCAGTACTATGCGGTGCCGCCGCCGCCGGAGCAGATCCCTCCGCCGGAGCGGGGAGTTCGACGGAGTGCGTTTGTGGGGTACGAGAGGTGGAAGAGTTACGGGGGTTGCCGGAGGCCGACGGCGACGGGAGAGGGGAACATTCACCGGAGGGTGTTGGAGTGCCTGAGGAGGATGCCGGCGGAGGAGGAGGAGAGGAAGGCGGCGGAGGGCAGCCGGTGCTTCCGGCACATGATGAGGGAGCGGCGGCGGCGGGAGAGGCTGAGCCAGAGCTACGCCGACCTCCACTCCATGATCGTGCCCAGGCCCAAGGCCAACAAGAACGCGATCGTCCTCTCGGCGGCGGCCTACGTCCGGGAGCTGAAGAGCACGAGGGATGCCCTGCAGCGGCGCCACCAGGAGCTCCGGGCGAGCCTCGGCGAGAGGGGCGCCTCCGACGCTGGAGGGAGGGCGCCGGAGGGGGGCGCCACCGTCAAGGTGCGCGTCGGCAACCCCACCTCCGCCGTCGACTCCATGATCGCCGCGCTCCGGCAGCTCAAGGGGATGGGCGTCCGGGCGAGGTCCATCCGGTCGTGCGCCGACGGCAAGGAG

>TRINITY_DN28444_c0_g1_i9_3
AGAGGGTGAGGAGGACCCGGATCAGCGAACGGATGAGGAAATTGCAGGAGCTTGTGCCCAACATGGACAAGCAAACAAATACGGCGGACATGCTGGATTTGGCCGTGGATTACATCAAGGATCTCCAGAAGCAGGTGAAGGGCTTATCGGAGAGCAAGGCCAACTGCACCTGCTCCAGCAAGTAGATAGATAGAAACGATGCAGCTGCAGCGTCACACATTGCATGCATGCCCTCGTAGATCGATAAGTACCCAAAACAAGGACGATCTCAGAGCCGCGGTGTCCCTTTAGCTGAGACGTACGTTTCAGCTAAAGGGAGAACAACGATATAGGTGGCTGCACAGAGAGCAGAACGGCGCAGGTAATATTATGCTGAGACGTTCTGTTAATTGACAGGACTCCCAGGCCAGCTGGTTTATATTTTTCAAGGAAGAGCTGCACGTTGAAGAAGGCGAAGGCAGAGGCAGAGAAGAGTAGCATATGTTTCCAATCCCATACGTTGCTGATCGTATAATGGTGTGTAACCTTAATGCTCATCGGCCGGTGAAAGGTAGAAGATCCGTACACTTTTGAGCAGATTGATGAAATCTCAGGGGATGCGGGGAGGTGTTGCATCAATAATGGGCGAAACAATATAAACTCTGATCCGGATACTTAAACGTGGTTGGGCAGTGTTACTCCCTCCGGCATCCGCATATATAATCTTCTGTGTGCACTTAGAAGGTTGTGTAAATACAGCTCTCGTTTGTTTTCCTTGTTGTGGGACCTGTGGGTACCTTCTATTTGTATATCCCATCGGAGCTTGAGGTTGGACCAAATGAGATTCCACGAGATCAGATGAATTGAAATATCCCTAGCTATTAGTATGAACAAATTTTAGAA

>TRINITY_DN29408_c1_g1_i4_3
GCCCACCAGAACATGAGGAAGCGGGTGCTGCAGAAGCTGAACACCTTCTTCGGCGGCTCCGACGACGAGAACTTCGCCCTCCGCCTCGACCGCGTCACGGACACCGAGATGTTCTTCCTGGCGTCAATGTACTTCTCCTTCCCCCAGGGGAAGGGCGCCCCCGGCAGGGCGTTCGCCTCCGGCAAGCACCTCTGGATCTCCGACGCCGCGCCCAAGCCCTCCTCCTCGGACTACTGCGTCCGCGCCTTCCTCTCCCGGTCCACCGGCACGCGCACGGTGGTGCTCGTGCCGTCGGAGGCGGGGGTCCTCGAATTGGGGTCGGTGAGCTCGGTGCCGGAGAACGCGGAGGTCCTGCAGATGATCAAGTCCATGCTGAAGCCCTCCCCATCCGCGTCCTCCGGCGGGGAGAAGAGGGAAGGCAGCGTCGGCGGCGCCGCCCACCCCGCGGCGTTGGCGCTGGGAGGGAAGCAAGCAGAGGAACGCCCAAGGATCTTTGGGAAGGATCTGAATCTGGCGAGGAGTACTCAGACCAGTGAGAAGATTACGGTGCCCAAGGTGGAGGAGCCACCGTGGGATCTGCAGAGCAGCAATGGCGCCGCCAGCCACTTGCCCTTCCCCAACATCAGGAAGAACCCCCATCTTCTGAATTGGAACCAGGCCCGCCATGTGAACGGAAACCAGACCCTGATCAACAGCAACCACCAGAAGTTTGGCAACGGGATCGTGATCAGCGGAGGCGCCGACGGGGTTAGGGGCGGCGCCGACGCAGACGCATCTCACAGATTGTTTGGCCACCAGAGCAACGGGATCAGGGAGGAACCCCGGATCAGCGGCTTCCAATCACAGAAGCAGCAACCGCCGCCGCCCCCAAGGCAGATCGATTTCTCCGGGACTGCTGCAACTTCCAGGACGGCCGGCGGCCCTGTTGCGGCGAAGCTCAACAATCTGGATTCCGAGCACTCTGATGCGGAGATCTCCTGCAAGGAAGACCGGCCAGCCGCGGTGGAGGAGAGGCGACCCAGGAAGAGAGGCCGGAAGCCTGCGAACGGGAGGGAGGAGCCCCTCAACCACGTCGAAGCCGAGCGCCAGAGAAGGGAGAAGCTTAACCAGAGGTTCTACGCGCTGAGGGCCGTGGTGCCCAACATATCCAAGATGGACAAGGCCTCCCTTCTCGGCGACGCCATCGCCTACATCACCGAGCTGCAGAAGAAGCTCAAGGAGATGGAATCGGAGAGGGAGAGGTTTCCTCCTGAGACGCCGGCGGCGGCGGCGGAGCACAAGACGCCGGCGGCGGCGGACTGCGCCGACATCGACGTCCAGACTGTGCGCGACGAGGTTGTCGTCCGGGTGAGCTGCCCCCTGGAGGCCCACCCTGTCTCGAGGGTCATCCATGCGTTCCGGGAGGCGCAGATAAACGTGGTGGAATCCAAGGTTTCAGCCGGCGGCGACATGGTTTTCCACACCTTCGTGGTCAAGTCCCATGGCTCCGAGCAGCTGATCAGGGACAAGCTGCTCGCTGCCTTCTCTCGTGAGATCCATCCTTCATAGCTTGGCCGGGGCTGCTCGTCACAGGTCACATTAAGCGCCGCCGCCGGAGGAAGAAGACGCAGAGCAAGACTAGTTTGTAGGATCATGACTGCACAGGTGCCGGTGATGCTTCAGTGTTCCAATTGTGTTTACTACTGTACTTTGAACATGTTCTTCTATAGATCATGTGCATGAAACTTAGTTGCTTCACACCACAGCTTTGTTCTAGAAATCAGGTTGCTGTTTTTTTTTTTTTTTTTTTTT

>TRINITY_DN22533_c0_g1_i12_2
CGCGCCCACGCCAAGTTCGGCAACAAATGGGCGACCATCGCCCGCCTCCTCAGCGGCCGCACCGACAACGCCATCAAGAACCACTGGAACTCCACCCTCAAGCGCAAGTATCCTGCGTCCTTCTCCGAGGACGCGATCGGCGCCGACGAGGCCGCGGGGGAGTCGATCCACAGCGGAATCAGACTCTTCGATCGCCACCACGACGACGATGACGACGAGCCGCCGCTGAAGCGGACGTCTAGCGGCGGGACCACGGTCTCCGGGCTCTGCATGAGCCCGGGCAGCCCCACCGATTCAGACGTCAGCGATCCCAGCCGCCACTCCTCATCCCCGCCCATCGCCACCGCCGCGTGCCACATCTTCCGGCCCGTGCCGAGGGGCGGCGCCATCGTCGCTCCGCCCATGGATCTGTCGCCGTCGATCAACAACGGCGGCAGCCTTGTGAACGACGACGATCCTTGCACGTCTCTCACCCTCTCTCTGCCCGGATCGGAATCGTGCGACGCAGCTATCCACCAGTTGGCGACGCCCACCCCAACCTCTGTTCCGACCGGCCGATATTTACAGGAGCAACAACAACAAGAACATCACGCCCTGGAAAATCCGCAGCCGACGGCGACGATACCACCACCCGTACAGCAGCTGCACTCCTTCATGGATCCCATCGACCCGCAGCGGCATCAGCGGCAGCAGTTCCCCTTGAGCACCGAATTCCTGGCGGTGATGCAGGAGATGATAAGGAAGGAGGTGAGAAGCTACATGCTTGGGCTGGAGCAGAGTGGGGCGATGTTCCTGCAGCAGCACCACCACCACCAGCAGCCGCAGCAGCCGCGCGTGGTGGTCGATGGCCTACTGAACGCCGCCGCCGCGAACCATCGCATCGGAGTTCGTAGGCTCCAGTAGTGTTCGGATCAATCCCCTCCCGCGTCGTCCTCATCTCAGCATCACGACACTCTCCACTCTCGGAAGAAATAATACTACTTTTATATATAATATTATATGGATTAGGAAGCGAACGGAGGAGCAGATGGGTTATCTCGTTAGTCGTAGCATCTCCCACTCTCTCTGGTTAGGCTGGGAAGCTGTAGATTCTTCCTCTTCTTCGGGAACTCTTCTCTCTGGCATCCACCATCTCTCTCTCTCTCTATCATATCCTGTTTAAGCCTCTCTTCATCGATCTTTTTACACTTCTTGTTTTGGTGGGCGGGCAAAATGGGGATTGTCTTCTTCTTCTTCCACACGGAGACGAATATGAAGCAGAGGGAGTCGGGCTTTTTGGGTTCAATCTGTGTACAGAGGCAAAACTTGAATGAGCGAGGATCAAATAAGATCCCAACGAAATGGCTAACGAAATAGTGGCAAAGAACGAGTCTTTCTTCCCCCTTCTTTCTGTAATCGCGACTCTCTTTTTTCGTGTAGATTTTTGAAAGACAGGTCTTCGCTGCTTCTCAAAGTGTAGTCCGATTCAATTGAGGGGAGAAATATCCTTCTTTTCCACAAAAGAAC

>TRINITY_DN22903_c0_g2_i1_3
CGCGCGGTTCTTGGAGGGGCTGGAGAAGTACGGCAAAGGGGACTGGAGGAGCATCTCGAGGAACTCGGTGGTGACGCGGACGCCGACCCAGGTGGCCAGCCACGCCCAGAAGTTCTTCCTCCGGCAGAGCTCGGCGGGCAAGAAGGAGCGGCGGCGGAGCAGCATCCACGACGTCGTCAGGCCCTGAAACCCAGAAGCTGCCGCAGCTTCTTCTCTCATCTCCAAGTACTTGCAAGTTGCAGGAGACATGACTCCACCACCCCCTCCCCAACCCCCAATTGGGATTAGCTAACACATATTATTACTTTTATACAACTGTTTGATCATGGTCAGGTGGTGTAAATAGGAATAGCATCTATTATAGGTTCTTACTTAGCTCTTTCAAATAAGATCAACTTGTGTAAATAACAGGCATTATTACCTTTTGATTTGTCC

>TRINITY_DN24043_c0_g1_i1_1
CGCAAAAGAAAAGAAAAAAAAAACCCTCCTTTTCTTCTTCTTCTTCTTCTAGCTCTCTGGTTCTCTCTTGCCAATCTACTTACGATGTTTGGAGAAGGCGGCGACCATTTCTGGGCGGCGGCGCTCCCCATCGACCCCCGCCTGCCCGGTCACTTCCCACCGCCCCTCTCTGTTTCCATACCAGCCGCTCCCTACCCAGACCACCACCAGGGCTTCGCTCCACCGACGACGGCGGCGGCGGCGGCTCCCTATCCCTGCGGCCCGGAGCACCAGCGGGGCTTTGCGCCGGCTTGGGGGCGGGCAGCATGGACGAAAGAGGAGAACAGGGTCTTCGAGGCGGCGCTGGTGGAGTTCCACGGGGCCAGCGAGGAGGAGCGGTGCAGGGGGATCGCGGCGCGGCTGCCGGGGAAGACCTGGGAGGACGTGAGGGATCACTACCAGCGTCTGGAGCGCGATCTGAGGGACATCGAGTCCGGCGTCATCCCCCTTCCCGACTACGCCATCGACTGGTTCGACTCCGGCGCCTGTGCCGGCTCGTCCGGAGGCGGCGGCGGCGGCGCGGGGAGGGCCAAGTCCGAGGAGCGCAAGAAGGGGATTCCGTGGACGGAAGAAGAGCACAGGTGCGCACGCACGAGAACCCGACCCAGCTGATCTCCATCTTTATCTGATCTCCATCCATCTAGCCTTTTCCGCTTCCGGAATTGATGATTTGGTTGCTCTGTGTTTGTTTTCTTCTGCAACCAGGCGGTTCTTGCAAGGGCTGGAGAAGTACGGGAAAGGTGACTGGAGGAGCATCTCGAGGATGGCGGTGGTGACGAGGACGCCGACCCAGGTGGCCAGCCACGC

>TRINITY_DN29083_c0_g1_i3_5
GACGGAGGCGGAAGGGGGGAGAGCGGCGGGGAGGGCGAGACGTGAGCAGGGAGAAAACCCAACTCGACCATCCGTTCCCCGACGGCCCTAGGGTTTGCGAGGGGCGGGGCGAGAGATGGGAATATAAAAAGGGACATGAGGCCGCGATGACGAGATCGGACGGCGCCGACGAGGAGATCTGGGGGACGCGGGAGGAGCTCCTGCTCGCCTGCGCCGTCAACCGGCACGGGACGCAGAGCTGGGACTCGGTGGCCATGGAGGTGCGGGCGAGGAGCCCCCCCTCCTCCCGCCTTCTCACCGCCCAGACCTGCAAGCGGCGGTACCACGACCTGCAGCGGCGCTTCGCGGCCGAATCCCACGGGATCGACGGCGGCGAGGCCGAATCCGCCGCGATCGCCCTGTTCGAGGCCCTGCGGGAGCTCCGCGTCGCCGAGCTCCGGCGCGAGGTCGCGCGCTACGACGTTTCCATCGGGTAATCTCCGCCTCGTGGAGCTTGTGTTATTTTTTTTGCGTATGATTTGATGGATGTCCACTGTTGGATAAGGATCCAACGGTTGATGGCCCTCGACGTTTCGAGATCTAAATCGCTATTTATTTTTACTTATTTATTCGCTTCTTTACTCGTTTATCTTTGTTTTTTTGTTGCTGTTAAGATCTCTCCAATTAAAAGTGAGAAGGCTCAAGGAGGACCGCGAGCGGAGCCTCCAAGAGGACCAGTCCGGCGATAAGTCCGATCCACCGGAGGTCGAGGGAGCCAAGAAGGAGGCCAACTCGCCGGCGTCCACTCCGGTAGACCTCCTCGTCGACAGAGTCGTCGATGGCGAGTCCGGCCGCTCGTGCAACGAGTCCAACTCGACCGAGCCCAAGGAAGGCCAAGATCCCCGCACCGCCGAGGACGGCAAGCTTGACGACGCATCGGGCGGCGCCGAGAACGGCGCCGTCCGGATGGATCTAGCCGCCGGCGGGGACGAGAAGCTGGCAGGCGAGGGATCCTATAACGGGAGCTCCGACACGGTCGCCAAGGGCGCGGAGGCGGCGACGACGCCCGCCGAAGAGCCCGCGGCGACGAACCCGACGCGCCAGCCGGACGCGGGCGAGTCGGGCGAGTCGGTGGCCGAATCGAAGGGAGACGCGTCGAAGGAGAGCAGCGACGTGCAGAGCTCGGCCAGCCTCTCCCGGCGACGCCGCGGCCGGAGGAAGGCGGCGGCGGCGGCGCCCTCCGGCGGGAGCAGCGGCGGGGAGGAGGCCGAGGCCGACGAGGTATCCCCCGTGACCAAGCGGGAAGCCGCCAAATCGCAGCCGTTGGCCTCCTTCCTCCAGACCATCCGCTCTCACAAACACGGCTCCGTGTTCGAGCGCCGGCTTGAAAGCCAGGAGAGCGCGCGGTACCGGAGTTTGATCCGGCAGCACATGGATCTGAGCACGGTGCACGGCAAGATGGAGGAGGGTGCGTACGCTACCAACAACGTCGACTTCTACCGCGATCTGCTGCTGCTCTGCACAAACGCCGTGGTCTTCTTCCCCAGGGGCTCCCTGGAGCACAAGGCGGGGGTCCAGCTCCGGCAGCTCGTCCGGAGACAGATACGGACGGCGTTCCAGAAGATCTCCCCTGCGGCGCCGGCGGCCTCCGCGGAAGAACCCGCTGCACCCCTGCCTCCCCCGCCGGCGCCTCGGCTGCCGGTGGCTCCCAAGGTGAAAGCCGAACCTGATCCTTCGGGCTTGCTCCGCGATAAGCCGATCTCCGCCCCGCCGGTGGTCGCATGCCGGAAGCGGAGCTCGATTTCGGGGAAGGTCCCGGCGAAGGTGCAGGCGGCAACGAAGGTAGAGGTGAGGCCGGAGCTGGAGAAGAAAGAGAAAGAGAAGGAGAAGGAGAAAGAGAAAGAACAAGAGAAAGAGATGGAGACAACGGAAAAGAAGCGGACGAAAGAGAGACAGACGGCAGTTGTGGCGACGAGGGGGTGGAGGACAAGCAAAGGTCGGGGGAGCAACAGCGGGGCCGGTAAGAACTCCGGTTCGAGCTCTGCGTCGGCCCCGGCAGATACCACAATAGGAACCTCCAGGGCGGAGCGTAAGAACGCTGGGAACAACGTGGCCAACCAGGCGGCCAACTCAGTGGCGAAGAAACGTAGCGCAGTGAACTTCTTGAGCCGGATAAACCGCAGCGCGACGTCCTCAAACGGCACGCTTCTGGAAACGCTGAAGAGCTCCGGCGGCGGGGGCAGGGTCGGGGGCGCCGAGCATAAGAAGGGGGCGCGGGCGGACGGAAGGAAGGACCAAGGGGGGAAGCAGGGATCGGGACCCGCCGGCAAGCAAGTGCGGGAGCAGAGTCCGCCGGTAAAGAGGAGCGTGGGGAGGCCCCCGAAAAGGCCGCCGCCGCCGCTGCCGTCGCCGCCCGCGAAGAGAGCGAAGGAGGCCGAGGCAGACGTCCCACCAGCCACGAGGTCGGCGGTGGCCAAGAAGAGAGGGAGGAGGTGATGGTTCTTGGTAGGTTCTGTATATTTATGTAGCAAATCGGGGCCATTAGCGTAACGTAACATCTTTTGATTTTTTGGCTATAACGTTGCCCCTTCACTTTTTTCCTCTCTTTTTCTTCTAGGTTTTTAACACTTCGGTTTCAACTTTTCACACATTTTATTTATTTATCATAGTCTGTAGTTGATAAAACCATCGAATGTGTACATTTTGTTGGGCATTGCGAGAAAAGGGTAAAGCCCATATGCAGCAACCGGTTTATATGTTAGGTGGTGGGCGTCCCCATTTATATGATCTCGATGAAGCCTGCCTCCGTCATTTTTTTGGTGTTGAATTAGAG

>TRINITY_DN29368_c0_g1_i2_4
GAGAGAGAGAGAGAGAGAGAGGGCGAGGTCACCCAACTCGACCTGGTTTTAGGCGGTGGTGGTGCCAGAGCTCGCCTCTTCTTTGCTTCCTCTGCCGCCGGCTGCAGTCTTCGGACAGCAACCGCCGCCTCTGCGGGCGTTAGCTCGCCAGTTGTCGAGATCTGGGAGCGGCAGCGGCGACTGGTGGAGGAGTTGGGGAGAGGGTAGAGATGATGAGGAGGAGAAGAACAGCGGATCACTGGTGATGGGCTCCGTCTCTTGTTGTTTAATTCAAACGTGGTCGTGGCTGGAGGGGAGTCTGATTGACTAATGAGGTACTCGTTTCGTCGGGTGGAGCGTCTGGTAGGGGACGTGGAAGGGGGATTCATACTTGTTGTTGAGTATTTGAAGTGAACCAGTGACGTGCTTCTCTCTTCCCTGTGAATTTGGTTCCGGCAGGAGCATGGCAATGGAAACCTATTCCTCTGGCGAAGATGTGATCATAAAGACGAGGAAACCATATACTATCACAAAGCAGCGGGAAAAATGGACTGAGGAGGAGCATAACAAATTTCTAGAAGCACTAAAGCTATATGGCAGAGCTTGGCAGCGTATTGAAGAGCATATTGGTACAAAGACTGCGGTTCAGATCAGAAGCCATGCACAGAAGTTTTTCTCAAAGTTGGAAAAAGAAGCACTAACCAAAGGTGTTCCATTGGGACAAGCTCATGGTATAGAGATTCCTCCTCCACGTCCTAAAAGAAAACCAAATAATCCATATCCTCGAAAGACTTTTGTTGGTGTTGTCCCATCATCTGGTGAAGGAAAGAATGAGAGACCATTGATTTCTGGTTCTTTGTGTTCCAAGAATAAAGTATTTGAATTGGAAATGGACACTCCTCATGAGAAAACTGCTGGAAGCCTAACATTGGGAGGAACGAAAGAGACATCTGAAGATAGTGAGTGTTCGGTGGTCCTGTCTCTTTTTCAGGAAGCACCATCAGCTTTTACACCCCCTGGAAACAATGTTCCCGCAGATGCATGTATCTCTAGGGAATTTGTTCCCATGGTGAAAGAAAATAAAGACAAGGAAGGAGATAAGGAATGTTCCCCAACTCTCAAAGGCAACAAGGAGCCGCATAATGGAAATATAGACATTGGCAGATTTGAAGGGCTTAGCATTGATACACAGATGAAATTGGCGCAAGGAACGTCAACTGGGATTAAGCAGCCAATCAATGTTGGTACATCATTACAGGAAGATTCCAAAGTTAACAAAAGCTACCTTAAACACATCCATGTATGTTCAATAGATAGACAGAGCAATGAAAGTGTGCAAACTCCAGGTTCAGGTGCAACATGCCTCACATCCTTATCTAATCGAGGACCAAATCGAAATCCCAACTTGGAGAAAGCTCCATCCCTCTCAGCAACAACTGATCACCATGCAAGCACATCAATGTCATCTGTTCATCATTCATTTGCTGCTTTCTCTCCCTTTACCCAGTTCTGTAGCAGTCAAGATGCCTATAGATCTTTCCTCAACATGTCATCTACATTTTCTAATCTCATCATGTCTACCCTGCTGCAAAATCCTGCTGTGCATGCGGCAGCTAGTTTAGCCGCTACTTTCTGGCCAGCCGCAGATGTCGAAAATTCCATTGGATCGACTTCAGAAACCTTTGTTGGAGGAATTCCTGTGAGACACATGAGTGGATCTCCAAGTATGGCTGAAATAGCCGCTGCTACAGTTGCAGCAGCTTCTGCATGGTGGGCCACACATGGGCTCCTACCCTTTTCCCATCCTGCTTTTCACAGTGGCTTCACATTTGCACCAGCATCTGCTGGCACAATTCCAGAGGCACAGGCTGCTCAAGCTACGGAAGATAACAAACAAAGGAAAGATGAAGTAACTCAAAGTCAGACGCGGATGGATACACAGCGAACTGTTGGTCCAGAACTATCCGCTCCAGGAATACAGTCATCTCCCCTGTCCTCATCGGACGCTCATGAGAGTGGCGGAGATGGAGGGTCTCAGAACATTATTCCAAAAGGCCATGTGGACAAGCAGGTGAATCCATCGCCAGGTTCTGCTGTCGATGAATCAGATATGGCAAGAAGCAAAAAGAAATTGGATCGTTCTTCCTGTGGCTCCAACACACCTTCGAGCAGTGAGGTGGAAACACATACTCTGTTGGAGAACATCGAAAGAGGCAAGGAAGAAACTAGAGAAGTTCATTTGAGTCATCCTCCTTGTGGTGGTGACACAAACAACCGTAGGCTAAGAAGTTGCAGCAATGTCAGCGAATCTTGGAAGGAAGTCTCCCAAGAGGGTCGACTCGCGTTTCAAGCGCTCTTCTCCAGAGAAGTACTTCCACAAAGCTTTTCACCTCCACGTGCCAAAGATGGGACAGTGGAAACAGCCAAGAACGAGAAAGAAGCGGCTGCTTTGACCGTAGATCTCAACAGCACTGTTTCTTCAGCGACTGATGTCGATCGTGCTCTGGGGGTCGACAAGCCCCCAAGGACCAGCAGCATGGACCAGGGTCTTCCAACAAATGAGATTGGACAAGTGAAGTTGAAGTCACGTAGAACGGGATTTAAGCCCTACAAGAGGTGCTCCATGGAGGCCAGAGAAAGCAGAGCACCACCGGTTGAAGAGAATGACAACAAGAGAATACGCCTGGAGGCTGAAGCCTCCTTGTGACTCCAATTTCCAAGCGTGCAATGGCTTGGTCGTCATAGCCGTTTACCCGTACATGCATGTAATATAAGATTCCTATTATTTATATCCGAGACCGAGAGAACTCGGAACTTTGTCATGCACTTGCGACTTCGGACTCATGGACTATTTAATTGTGTCCTGTTTCTCTTTTCATGTAATTACTGGTTCCAATCGATAGCCTTCGCCAATGCCGGTCAGTTAAGTACCTGGTCTGCAAATAAGGTGGAGTCCTTTTTTTTCTGTTCTCATTTTAGCAGTAGCTTCTTGTACTGTTCAGTTGCATGGAATGGCAAACATTATAATCG

>TRINITY_DN30052_c0_g1_i9_5
AAGAAAGAAAAAAAGACCTCCTTTTTCTCCTCTGGTTCTCCGGTTCTCTCCTCTCTCTCTCGCCGATGTTTGGAGAAGGCGGCGACCCTTTCTGGGCGGCGGCGCTCCCCATCGACCCCCGCCTGCCCGGTCACTTCCCACCGCCCCTCTCTATTTCCATACCATCCGCTCCCTACCCAGACCGCCACCAGGGCTTCGCTCCACCGACGACGACGGCGGCGGCGGCTCCGGTTCCCTATCCCTGCGGCCCGGAGCACCTGCGGGGCTTTGCGCCGGCTTGGGGGGGGACAGCGTGGACGAAAGAGGAGAACAGGACCTTCGAGGCGGCGCTGGTGGAGTTCCACGGGGCCAGAGAGGAGGAGCGGTGCAGGGGGATCGCGGCGCGGTTGCCGGGGAAGACCTGGGAGGACGTGAGGGAGCACTACCAGCGTCTGGAGCGCGATCTGAGGGACATCGAGTCCGGCGTCATCCCCCTTCCCGACTACGCCATCGACTGGTTCGACTCCGGCACGGCGGCCGGCTCGTCCGGAGGCGCGGGGAGGGCCAAGTCCGAGGAGCGCAAGAAGGGGATTCCGTGGACGGAAGAAGAGCACAGGCGGTTCCTGCAAGGGCTGGAGAAGTACGGGAAAGGTGACTGGAGGAGCATCTCGAGGATGGCGGTGGTGACGAGGACGCCGACCCAGGTGGCCAGCCACGCCCAGAAGTACTTCCTCCGGCAGACCACGGAGGCCGGCAAGAGGGACCGGCGGCGGAGCAGCATCCACGACGTCACCACCACCTGAAGAAGAAGAAGAAGAAGAAGAAGAAGGGGAAGAAGAAGACCACCCGCCCAGAAAAGCCCCTTCTTTTGGATTATTGATCCCTGGTCCCTCCCTCCAACTTGCTTTTGTCGTTTACCCTCTAACTTTTGTTCATATGTGTCAGCTTCAGGAACCAGTTCTAAGTCCTCAATATTCTTGTCCATAGTTAATGTAATTAGTGGTGGAGACAGTGATTAGGAGGGGTTAATACCGGATTTATATATCAGTTTAAGGGTATGTTTAGGGACTTCCTCTTTGATGTTGATATAGGAATACGGTGTCTTCTTGACTGTGACATTAAACACTTCGATGTGGACTGATTAAAAGGACAGCAGGGGTCTGTAGTTAGTGGATAAAATTGGTATTTCTTTTATGGATAAGAAATTCCATTTATAGGTTGTAAAAAAGGGATTTAAGGATTTTTTAATGGTGCAAGGATGGATGAAAGGTTCCAAAATCTTGTGGCAATGCGAAGGTCCAAAGGCCACA

>TRINITY_DN30096_c0_g1_i8_5
TTCGCAAGGGAACGAACTCGTCCCCAAGGTGAGGAAGCCATACACCATGACTAAGCAGAGAGAGAGGTGGACGGAGGAAGAGCATAACAAGTTTCTGGAGGCACTAAAGCTATATGGCCGAGCATGGCGCCGCATTGAAGGTAGTCAAGGATCCCCAGCGATGTGGAGCAGAATCCATTGAAATTCCTCCTCCTCGCCCCAAAAGGAAACCTTTACATCCATATCCTCGGAAATCGGGGCATTCACCATCAAGGGATGTTGCCCTCACAGCTGCAAAAAGATCCCCCTCACCGACGCCATCGGTACTATCCGAACAAGGAGACAGATCTCCAACATCAGTATTATGTTCATCACCAATCTGTAGCCAGCAAAGCTGTTGCACACCTCCAGATTCATCTGCCAATTCCTCCTGTGAACTTGGTACATCTTCCACTGAACAAGACAACTTGTGTTCACAATTCGCCTCACGGAATAGGGAGGAAGATAGATCTCAGTCTGAACACCCACCAAGCATGGATATTGGATCGCAGGAGGCTGATTCCGTGATGGAAACTGAACTGAGCACCCAAGATAATCCCCGCGCTGCAGGGAGCCTTATGCTGTTTGGGAGAACTGTTCTGGTGGCAGACTCACAGAATCCTTCAGAACATCTCAAGACACCTCCAGGTATCTACAGTAACGACGGGGAAGACGCCGCCAATGTTGAGGTAGAGCTAAGAGGTGGATCACGAAGACAACTTTTTGAGATTCCATGCCAAGAGGGATGGAATCCATGGCCGCCTTGTGGGATGCCGCCAATGGTCTTCGTTGCAGCTTGGCCTGCAAACCTTGCAGGTGTCAAAGAGGATGGCATTTCATCTCAACCTTGGTGGCCTTCCTGTGCAACGCCATATCCGTTGATTCCTTCATACAACGTTGGCCAGCCACCGGAATCTGCCTCTGTATCGATAGAAGTGCGAGATGTCGAGGAGACGCAAAAGCCAGGCTCTTCTTGGGATGGTTCTAGCACTTGTGAGTCGATGACAAGGAGACCAGTAACACAGCTGTTGCTTATTTAAGATCTAGAGTAAGAACAGCTCCAGTGCTGGCTTTAGGCAGCTGTGGGAGTGGGTTCGTGGTTTACAAGTGTTCTTGAGCAATCTAAAAATTGCGACTGACACAATAGTTGTGTAGGCTTTGGGGATGACCGTGATATTGACCAAAATTTTCCGTTTTTTTTACCCCATTTCAATTGTGTGATCCCCACCCTCCATTCTGAAATATCTGATTCTTGTTACGGTCAGCAATGATCGTAATGCCCAGTTGTCAACAACTGACAAGATGAAAGTG
