# Supplementary material for: Transcriptomic and biochemical insights into key gene networks driving bulbil development of Pinellia ternata (Thunb.) Breit
Source: PLoS One. 2025 Feb 11;20(2):e0314396. doi: 10.1371/journal.pone.0314396 (PMC11813136; doi:10.1371/journal.pone.0314396)
Supplement: Attachment — Submitted filename: rebuttal letter.docx [file pone.0314396.s016.docx]

**Response to Editor**

We thank the academic editor, Tobias Isaac Baskin, for his comments. Based on your suggestions, we have made point-to-point revisions and additions, we have reviewed a large number of recent studies, in the introduction we have added appropriate content and further elaborated on the significance of this work and additions to previous studies, and in the experimental materials section we have explained the division of the sampling phases, thank you once again to the journal and Tobias Isaac Baskin for his invaluable comments.

**Editor Comments 1:** I am rejecting this version without review because your introduction is incomplete. In lines 85 - 89, you wrote: “However, at present, the research on *P. ternata* mainly focuses on the physiology of stress, while the growth and development of bulbils, especially the differences between sucrose and starch metabolic pathways and endogenous hormone metabolic pathways, have rarely been reported.” Consistently, your introduction cites no research about what has been learned about bulbil metabolism and development in this species.

However, earlier this year, Shu et al. (Front. Plant Sci. 15:1343222. doi: 10.3389/fpls.2024. 1343222) published a comprehensive review on bulbil development; the review focuses on *P. ternata* and includes discussions of development, metabolism, and transcription factors. Many papers were cited with relevant work in this species.

**Author's Response:** We have reviewed the most recent and up-to-date literature on the development, metabolism, and especially endogenous hormone and sugar metabolism of *P. ternata* bulbils, including, of course, the review by Shu et al. (Front. Plant Sci. 15:1343222. doi: 10.3389/fpls.2024. 1343222) that you referenced, and have added an entire paragraph to review the results of previous studies and thus further elaborate on the significance of our work in the field, as described below:

*P.* ternata bulbils have become the focus of production workers during operations because of their dual roles as medicinal materials and propagation materials. A large number of studies have been conducted on the bead buds of P. ternata, including the development of bead buds, the mechanism of bead bud genesis, the response of bead buds to biotic or abiotic stresses, and the high-yield cultivation techniques of bead buds, and so on. Similar to other bulbous species, the development of pearl buds is closely related to phytohormones, and there is evidence that IAA synthesized by the leaves and transmitted by the petiole promotes the initiation of pearl buds and the early development of pearl buds in *P. ternata* [20]. In some other plants such as lilies, IAA may act on bead bud genesis and development either indirectly or in synergy with cytokinins (CKs) and solanum lactone (SL) [21]. In addition, several growth hormone analogs have shown modulation of bead buds [22]. The complex regulation of IAA on the bead buds of *P. ternata* has not been deeply revealed yet. Brassinolide (BR) and cytokinins (CK) have long been recognized to play important roles in asexual reproduction, and the release of CYCD and ARF proteins triggered by BRs in *P. ternata* bead buds further regulates the expression of a series of downstream genes [23]. CKs, on the other hand, are thought to play a role in the early initiation of bead buds, and these effects may also interact with other hormones. This has been corroborated in species such as Lilium lancifolium [24] and Lycoris chinensis [25]. Unlike CKs, the regulatory effects of GA are often exerted in the later stages of the development of *P. ternata* bead buds [26]. Often considered a developmental inhibitor, ABA inhibits stem elongation and bead budding in *P. ternata* and promotes bead bud dormancy [27]. A recent study suggests that salicylic acid (SA), and jasmonic acid (JA) may act synergistically to positively regulate the genesis of *P. ternata* bead buds [28]. It is well known that sugar metabolism plays the role of the most basic energy conversion as well as storage in the plant body. The development of buds in *P. ternata* is essentially a transfer of energy substances, and the catabolic transfer of sucrose and starch is the starting point of bud development [29]. In addition, the role of sugar metabolism for bead buds may not be limited to initiation, as it has been shown that a large accumulation of starch is observed in the later stages of development of hemlock bead buds [30]. Similar findings have been made in lily bulbs. [31]

Endogenous hormones and sugar metabolism, as the two core modules of *P. ternata* bead budding, have been the subject of numerous previous studies. However, the large number of gene regulatory networks involved and the regulatory relationships among these genes are the trend and direction of future research on *P. ternata* bead buds. Therefore, In this study, according to the shape of bulbils, their growth was divided into five stages, namely, initial formation stage (ZY_1), scale differentiation stage (ZY_2), maturity stage (ZY_3), emergence stage (ZY_4) and harvest stage (ZY_5). The physiological and biochemical indexes of the bulbil samples obtained at the five stages were detected, and then the transcriptome sequencing was carried out. Next, the starch and sucrose metabolic pathways, endogenous hormone metabolic pathways, and transcription factor (TF) and hormone signal transduction pathways were analyzed. At last, based on the Weighted Gene Co-expression Network Analysis (WGCNA) algorithm, a whole transcriptome co-expression network was constructed for phenotypic traits, so as to screen potential transcripts related to endogenous hormone, sucrose and starch metabolic pathways. Through WGCNA, the transcriptional regulatory network of sugar metabolism and endogenous hormones affected by the development of bulbils was identified, which will help to discover functional genes and molecular mechanisms related to the development of *P. ternata* bulbils.

**Editor Comments 2:** In addition, others have already divided the development of P. ternata bulbils into stages that are similar but not exactly the same as yours. You should either use the accepted stages or clearly explain why you adopt a different standard.

I presume that your work goes beyond the many cited studies. However, you are responsible for comparing your work to what has been previously published and explain what specific gaps you are trying to fill. Such an account is a standard part of scientific papers. I believe that you can revise accordingly so that the paper can be sent out for review.

**Author's Response:** We have added the appropriate explanations in the Experimental Materials section as follows:

The development of *Pinellia ternata* buds is routinely divided into three stages: initiation stage, differentiation stage, and swelling to maturity stage, but considering that in actual production, P. ternata often appears to be inverted, and then the new petiole will be born from the buds, so we set up two additional sampling stages, ZY_4 and ZY_5, As shown in the figure below, the newborn bead buds expanded and emerged secondarily.
