# Supplementary material for: Transcriptomic and biochemical insights into key gene networks driving bulbil development of Pinellia ternata (Thunb.) Breit
Source: PLoS One. 2025 Feb 11;20(2):e0314396. doi: 10.1371/journal.pone.0314396 (PMC11813136; doi:10.1371/journal.pone.0314396)
Supplement: Attachment — Submitted filename: reviewed.docx [file pone.0314396.s017.docx]

**Analysis of key gene networks controlling the characteristics of *Pinellia ternata* Bulbils development by transcriptome,physiological and biochemical responses**

Xiwei Jia^1,2^, Xijia Jiu^1^,Yuan Liu^1^,Chao Guo^2^,Dong Liu^1^,Xin Zhao^1^,Honggang Chen^1,3^ and Tao Du^1,3^*

^1^ College of Pharmacy, Gansu University of Chinese Medicine, Lanzhou, Gansu Province, China

^2^ College of Life Sciences and Technology, Ningxia Polytechnic, Yinchuan, Ningxia Hui Autonomous Region, China

^3^ Northwest Chinese and Tibetan Medicine Collaborative Innovation Center, Gansu University of Chinese Medicine, Lanzhou, Gansu Province, China

* Corresponding author

Email address: gslzdt@163.com

35 Dingxi East Road, Lanzhou, Gansu Province, 730000, China

**Abstract**

Bulbils of *P. ternata* is is an important raw material for medicinal use. At present, the understanding of the growth and development characteristics of *P. ternata* bulbils and the gene regulation network is incomprehensive. It is of great importance to deeply understand the growth and development and molecular mechanisms of bulbils for protecting and developing its medicinal resources. Based on physiological and biochemical reactions as well as transcriptome technology, this study determined the content of endogenous metabolites and related enzyme activities during the five growth stages of the bulbils, obtained the transcriptome information of all samples, and finally, screened the key transcripts involved in the development of the bulbils by WGCNA.The results showed that the contents of sucrose and starch increased significantly in the ZY_2 and ZY_4 stages, and the changes in the activities of SPS, SuSy, and SS were basically consistent with the changing characteristics of sucrose and starch content. The contents of ABA and JA generally showed an increasing trend from ZY_1 to ZY_4, while the content of IAA was significantly higher only in ZY_1 and ZY_4 stages compared to other stages. Based on RNA-Seq analysis, there were 12 key enzyme genes differentially expressed in the sucrose-starch metabolic pathway, and 14 enzyme genes differentially expressed in the above-mentioned endogenous hormone metabolic pathway. Their expression characteristics well supported the measurement results of physiological and biochemical substances. Eight TF families also showed characteristic differences in different periods, mainly reflected in the ZY_3 and ZY_5 stages. In addition, four core transcripts involved in the bulbils development process were screened using WGCNA. This study provides an information source for analyzing the molecular mechanism of bulbils growth and development, and also helps to address the lack of genetic information in non-model plant species.

**Keywords:***Pinellia ternate*Bulbils; sucrose and starch metabolism,; plant endogenous hormones; transcriptome.

**Introduction**

**Abstract Guidance – remove this box before submitting!**

- No more than approx. 500 words (or 3,000 characters).
- Self-contained and concisely describe the reason for the work, methodology, results, and conclusions. Uncommon abbreviations should be spelled out at first use. Do not include footnotes or references.
- Headings in structured abstracts should be bold and followed by a period. Each heading should begin a new paragraph. For example:

**Background.**The background section text goes here. Next line for new section.

**Methods.** Methods section here, then new line.

**Results.** Results section here, then new line.

**Discussion.** Discussion section here.

*P. ternata*, belonging to the family Araceae, is a perennial herb of high medicinal value, and its dry tuber is used in medicine[1]. Currently, *P. ternata* serves as a botanical medicine commonly used by traditional Chinese medicine practitioners in China and many Southeast Asian countries[2]. According to the theory of traditional Chinese medicine, *P. ternata* can be applied to eliminate dampness to reduce phlegm, descend perverse rise of stomach-qi to stopvomiting, dissolve lumps and resolve masses [3,4]. However, the chemical constituents of *P. ternata* are relatively complex, including organic acids, proteins, amino acids, alkaloids, polysaccharides, and trace elements, among which organic acids ehibit antitussive, expectorant and anti-tumor properties [5]. As reported by modern pharmacological studies, *P. ternata* can relieve cough, dissipate phlegm, and stop vomiting, and exert anti-gastric ulcer, coagulant, anti-tumor, anti-bacterial, anti-inflammatory, anti-oxidative, anti-epileptic and other pharmacological effects, acting as a prospect for medicine development[6,7]. Since *P. ternata* has been proven to be effective, there is a growing demand for it in China and foreign countries, and the wild medicinal resources can no longer meet the market demand[8]. Notably, *P. ternata* is a vital herb in the traditional Chinese medicine formula for treating and preventing COVID-19. According to statistics, in the first half of 2020, the total export volume of *P. ternata* exhibited a year-on-year growth of about 34% [9].

Bulbils are special organs for asexual reproduction formed by some plants in nature [10], which share similarities with tubers, bulbs and bulblets. Plants such as the genus Pinellia (family Araceae), the genus Lilium (family Liliaceae), the genus Sedum (family Crassulaceae), and the genus Dioscorea (family Dioscoreaceae), produce bulbils containing a lot of nutrients and secondary metabolites[11]. Sucrose and starch metabolism in the bulbils of *Sagittaria sagittifolia* have been previously explored using transcriptomics and the vital genes in its metabolic pathways have been identified, indicating that sucrose and starch metabolism play a vital role in the formation and development of bulbils [12]. In higher plants, sucrose and starch are the main forms of carbohydrate transportation and storage, respectively. Starch synthesis needs to be completed after sucrose is decomposed and transformed in the "reservoir" organs of plants [13]. Sucrose and starch metabolism have attracted the attention of many scholars in the field of plant physiology and biochemistry [14], and their metabolic processes are very complicated, involving dozens of enzymes [15]. Additionally, sucrose and starch metabolism turns out to be the primary metabolic pathways during the growth and development of lily bulbs. The sucrose synthase (SS) and invertase (INV) expression levels are remarkably up-regulated at the stage of bulblet scale differentiation, and the sucrose phosphate synthase (SPS) expression level is up-regulated at the stage of bulb morphogenesis. At the same time, the expression levels of enzymes involved in starch synthesis differ at different growth stages of bulblets and mother bulblets[16]. Other studies have manifested that the sucrose level may serve as the main regulator of potato tuber formation, and the formation of tubers in vitro displays high dependence on sucrose concentration [17]. It is widely known that endogenous hormones are efficient organic compounds synthesized in plants, which exert vital effects in the plant organogenesis and morphogenesis. It has been revealed that cytokinin (CK) promotes potato tuber formation, while gibberellin (GA) and strigolactone (SL) exert inhibitory effects [18]. He also confirmed that CK can positively regulate the formation of lily bulblets [19].

*P. ternata* bulbils have become the focus of production workers during operations because of their dual roles as medicinal materials and propagation materials. A large number of studies have been conducted on the bead buds of *P. ternata*, including the development of bead buds, the mechanism of bead bud genesis, the response of bead buds to biotic or abiotic stresses, and the high-yield cultivation techniques of bead buds, and so on. Similar to other bulbous species, the development of pearl buds is closely related to phytohormones, and there is evidence that IAA synthesized by the leaves and transmitted by the petiole promotes the initiation of pearl buds and the early development of pearl buds in *P. ternate* [20]. In some plants such as lilies, IAA may act on bead bud genesis and development either indirectly or in synergy with cytokinins (CKs) and solanum lactone (SL) [21]. In addition, several growth hormone analogs have shown modulation of bead buds [22].The complex regulation of IAA on the bead buds of *P. ternata* has not been deeply revealed yet. Brassinolide (BR) and cytokinins (CK) have long been recognized to play important roles in asexual reproduction, and the release of CYCD and ARF proteins triggered by BRs in *P. ternate* bead buds further regulates the expression of a series of downstream genes[23].CKs, on the other hand, are known to play a role in the early initiation of bead buds, and these effects may also interact with other hormones. This has been corroborated in species such as *Lilium lancifolium* [24]and *Lycoris chinensis* [25]. Unlike CKs, the regulatory effects of GA are notabe in the later stages of the development of *P. ternata* bead buds[26]. Commonly considered a developmental inhibitor, ABA inhibits stem elongation and bead budding in *P. ternata* and promotes bead bud dormancy[27].A recent study suggests that salicylic acid (SA), and jasmonic acid (JA) may act synergistically to positively regulate the genesis of *P. ternata* bead buds[28].It is well known that sugar metabolism plays the role of the most basic energy conversion as well as storage in the plant body. The development of buds in *P. ternata* is essentially a transfer of energy substances, and the catabolic transfer of sucrose and starch is the starting point of bud development[29].In addition, the role of sugar metabolism for bead buds may not be limited to initiation, as it has been shown that a large accumulation of starch is observed in the later stages of development of hemlock bead buds[30].Similar findings have been made in lily bulbs.[31]

Endogenous hormones and sugar metabolism, as the two core modules of *P. ternata* bead budding, have been the subject of numerous previous studies. However, the large number of gene regulatory networks involved and the regulatory relationships among these genes are the trend and direction of future research on *P. ternata* bead buds. Therefore, In this study, according to the shape of bulbils, their growth was divided into five stages, namely, initial formation stage (ZY_1), scale differentiation stage (ZY_2), maturity stage (ZY_3), emergence stage (ZY_4) and harvest stage (ZY_5). The physiological and biochemical indexes of the bulbil samples obtained at the five stages were detected, and then the transcriptome sequencing was carried out. Next, the starch and sucrose metabolic pathways, endogenous hormone metabolic pathways, and transcription factor (TF) and hormone signal transduction pathways were analyzed. At last, based on the Weighted Gene Co-expression Network Analysis (WGCNA) algorithm, a whole transcriptome co-expression network was constructed for phenotypic traits, so as to screen potential transcripts related to endogenous hormone, sucrose and starch metabolic pathways. Through WGCNA, the transcriptional regulatory network of sugar metabolism and endogenous hormones affected by the development of bulbils was identified, which will help to discover functional genes and molecular mechanisms related to the development of *P. ternata* bulbils.

**Materials & Methods**

**Plant materials**

In this experiment, sampling was performed in the *P. ternata* planting bases in Qingshui County, Tianshui City, Gansu Province, China, for subsequent experiments at Gansu University of Traditional Chinese Medicine. Bulbils of the same size (0.8-1.0 cm) were planted in three plots (10 m^2^ per plot). After planting, the growth state of bulbils was subjected to irregular observation. Samples were collected when new bulbils were initially formed (ZY_1, the first stage), and then collected at 26 d (ZY_2, differentiation of the bulbil), 52 d (ZY_3, swelling to maturity), 78 d (ZY_4， secondary seedling emergence period) and 104 d (ZY_5， harvest period). respectively， The development of *Pinellia ternata* buds is routinely divided into three stages: initiation stage, differentiation stage, and swelling to maturity stage, however considering that in actual production, *P. ternata* often appears to be inverted, and then the new petiole will be born from the buds, so we set up two additional sampling stages, ZY_4 and ZY_5, and the changes in the morphology of *P. ternata* buds at different sampling stages are shown in Figure 1. Immediately after the rinsing with distilled water, the samples were put into liquid nitrogen for quick freezing, and then stored in a -80°C refrigerator until further analysis.

**Figure 1:** Morphological characteristics of bulbils at different growth stages.

**Determination of the content of endogenous hormones, sucrose and starch as well as the activities of sucrose and starch synthesis-related enzymes**

Enzyme-linked immuno sorbent assay was adopted to determine the content of endogenous hormones and the activities of sucrose and starch synthesis-related enzymes. Specifically, fresh samples (0.1 g) were extracted with 0.9 mL of phosphate-buffered saline (pH 7.4) and centrifuged at 1000×g for 20 min (REFERENCE?). The determination was conducted using the Indole-3-acetic acid (IAA), GA, abscisic acid (ABA), jasmonic acid (JA), SPS, SuSy and SS detection kits (Shanghai QCheng Biotechnology, China) according to the kit instructions. In addition, the kits provided by Nanjing Jiancheng Bioengineering Institute were utilized to determine the sucrose content and starch content by ultraviolet colorimetry and spectrophotometry, respectively, according to the kit instructions for specific steps.

**Ribonucleic acid (RNA) isolation, complementary deoxyribonucleic acid (cDNA) database preparation, and transcriptome sequencing and assembly**

According to the designated scheme, the total RNA was extracted from samples, and the DNA was digested by deoxyribonuclease (DNase). After the enrichment of the messenger RNA (mRNA) by Oligo (dT) magnetic beads, the first-strand cDNA and second-strand cDNA were synthesized with small fragments of the mRNA as templates,. Next, after purification by a kit (name company), the double-stranded cDNA was subjected to terminal repair (method?), A-tailing for sequencing adapter connection, and finally polymerase chain reaction (PCR) amplification. After qualified by Agilent 2100 Bioanalyzer, the constructed database was sequenced by Shanghai OE Biotech Co., Ltd. using Illumina HiSeq TM 2500.

**Bioinformatic analysis**

Trimmomatic[32] was employed for raw data (raw reads) processing in FASTQ format, and clean reads were obtained after the removal of reads containing ploy-N and low-quality reads, and then they were assembled into expressed sequence tag clusters (contig), followed by de novo assembly into transcripts using Trinity software[33]. Next, unigenes were aligned with NCBI's non-redundant (NR), Swiss-Prot, and gene phylogenetic databases to annotate their functions. After the functional annotation of unigenes, the number of reads aligned to unigenes in each sample was obtained using bowtie2 software[34], and the expression level [the value of fragments per kilobase of exon model per million mapped fragments (FPKM)] of unigenes was calculated by eXpress [35] software. Differentially expressed genes (DEGs) were filtered with q<0.05 and foldChange >2 as the criteria. Based on the hypergeometric distribution, Gene Ontology (GO) enrichment and Kyoto Encyclopedia of Genes and Genomes (KEGG) pathway enrichment analyses of DEGs were performed by R, respectively.

**Quantitative reverse transcription and PCR (qRT-PCR)**

QRT-PCR was used to verify 9 DEGs involved in the metabolic pathways and signal transduction pathways of starch, sucrose and endogenous hormones. QRT-PCR included two steps, namely, RT and PCR. Firstly, RT reaction system was prepared using 0.5 µg of RNA, which was kept at 42°C for 15 min on the ABI 970 PCR instrument, and then the RT enzyme and gDNA remover were inactivated at 85°C for 5 s, and cooled at 4°C. Next, the cDNA in each tube was diluted using 90 μL of nuclease-free H_2_O to obtain the final volume of 100 μL, and detected on the machine after the PCR Mix. Finally, the relative expression levels of genes were normalized and calculated by 2^-∆∆Ct^ method.

**Data analysis**

SPSS software (26.0, IBM, Chicago, United States) and Excel 2016 were adopted to statistically analyze all data. The physiological responses of bulbils and the differences in the levels of sucrose, starch and hormones at different growth stages were examined by analysis of variance (ANOVA). The least-significant difference (LSD) of significant data was calculated at *p<*0.05, and the results were expressed as mean ± standard error. There were three biological replicates of experimental data.

**Results**

**Variation characteristics of carbohydrate content and related enzyme activities in *Pinellia ternata* bulbils at different growth stages**

The contents of sucrose,starch and the activities of sucrose phosphate synthetase, aldolase, sucrose synthetase and starch synthetase were measured at different growth stages. The results showed that the changes of sucrose content and starch content were basically the same during the growth period, the contents of both increased significantly in the ZY_2 and ZY_4 stages, and were significantly higher in the ZY_4 stage than in other stages, and the contents of both decreased significantly in the ZY_5 stage. The difference was that sucrose content decreased significantly in the ZY_3 stage, while starch content still increased slowly (Figs 2A and 2B). The characteristics of sucrose and starch content in the five stages are closely related to the growth law of *P. ternata*. The ZY_2 and ZY_4 stages are the key stages for polysaccharide accumulation in the bulbils. In the ZY_3 stage, the germination of new leaf stalks requires energy consumption and active physiological and biochemical reactions, so it is speculated that the decrease of sucrose content is closely related to this process. In the ZY_5 stage, the leaves and branches of *P. ternata* withered, and the content of sucrose and starch in the bulbils decreased, which might have been caused by the consumption of respiration.The change in characteristics of sucrose content were consistent with those of sucrose synthetase and sucrose phosphate synthetase (Figs 2C and 2D), however, the variation characteristics of starch synthase and starch content in bulbils were similar (Fig2E).

**Figure 2:** Determination of carbohydrate content and related enzyme activities in *Pinellia ternata* bulbils at different growth stages. A. Determination of sucrose content at different stages. B. Determination of starch content at different stages. C. Determination of SuSy activity at different stages. D. Determination of SPS activity at different stages. E. Determination of SS activity at different stages. The x-axis displays different growth stages, while the y-axis represents the content or activity of the measured substance.

**Variation characteristics of endogenous hormone content in *P. ternata* bulbils at different growth stages**

The contents of endogenous hormones in *P. ternata* bulbils at 5 growth stages were measured simultaneously (how simultaneously?). The results showed that there was no difference in gibberellin content between the ZY_1 and ZY_4 stages, which remained at a low level and decreased significantly in the ZY_5 stage (Fig3A).The content of abscisic acid increased significantly only at the ZY_4 stage, and then decreased significantly at the ZY_5 stage (Fig3B). Jasmonic acid content generally showed an increasing trend during the growth and development of bulbils, and reached the maximum value in the ZY_4 stage (Fig3C). Auxin content generally decreased in the early stage, and significantly increased in the ZY_4 stage (Fig3D). In general, the contents of the four hormones decreased significantly in the ZY_5 period, which was the end of *Pinellia ternate* growth period.

**Figure 3:** Determination of endogenous hormone content in *Pinellia ternata* bulbils at different growth stages. A. Determination of GA content at different stages. B. Determination of ABA content at different stages. C. Determination of JA content at different stages. D. Determination of IAA content at different stages. The x-axis represents different growth stages, while the y-axis represents the content of the measured substance.

**Whole-genome analysis of *P. ternata* bulbils at different growth stages**

The transcriptome data of *P. ternata* bulbils at five different growth stages were compared using the second-generation transcriptome sequencing method on the Illumina platform. Each group of samples had three biological replicates, totaling 15 samples, and a total of 107.62 G of Clean reads were obtained. The effective data volume of each sample ranged from 6.87 to 7.45 G, the Q30 base distribution was 90.18-91.83%, and the average GC content was 53.24%. Through splicing, 68,391 unigenes were obtained, with a total length of 69,797,270 bp and an average length of 1,020.52 bp. A total of 40,251 genes were annotated to the NR database, 26,966 genes to the Swissprot database, 8,084 genes to the KEGG database, 21,371 genes to the KOG database, 33,841 genes to the eggNOG database, 23,296 genes to the GO database, and 22,343 genes to the Pfam database (Fig4A). Subsequently, the reads were aligned to unigenes, with an alignment rate of 80.56-88.67%, which enriched the available genomic information of *P. ternata*.

According to the statistical analysis of DEGs in *P. ternata* bulbils at different growth stages, totally 11,880 DEGs (7,472 up-regulated and 4,408 down-regulated) between ZY_2 and ZY_1, 9,865 DEGs (5,253 up-regulated and 4,612 down-regulated) between ZY_3 and ZY_2, 7,989 DEGs (3,606 up-regulated and 4,383 down-regulated) between ZY_4 and ZY_3, 24,785 DEGs (11,617 up-regulated and 13,168 down-regulated) between ZY_5 and ZY_4 were identified (Fig4B). There were 857 DEGs shared by the four comparison groups of samples, which suggested that these 857 transcripts are continuously expressed at different growth and development stages of *P. ternata* bulbils (Fig4C). The NR database was used for the functional annotation of *P. ternata* transcripts (Fig4D). The results revealed that the number of annotations in *Colocasia esculenta* was the largest [29,399 (73.04%)], while that in *Macleaya cordata* was the smallest [246 (0.61%)]. In order to verify the reliability of RNA-Seq data, 9 single genes encoded, namely, GBE1, pgm, WAXY, E2.4.1.13, IAA, LUT5, ABF, E1.14.17.4 and AOS, were selected for qRT-PCR validation. The results manifested that there was a good agreement between RNA-Seq data and qRT-PCR data (*r^2^=*0.9753), proving the reliability of the relevant gene expression levels (Fig4E).

**Figure 4:** Statistics of transcriptome sequencing results of *Pinellia ternata* bulbils at different growth stages on the Illumina platform. A. The number of annotations of the spliced unigene sequences in the NR, Swiss-Prot, KEGG, KOG, eggNOG, GO, and Pfam databases. B. The number of DEGs in different comparison groups. C. Venn diagram of a single gene in each group. D. Species distribution of the identified transcripts in the NR database. E. Correlation analysis of the qRT-PCR and RNA sequencing (RNA-Seq) results for 9 genes.

**KEGG: pathway enrichment analysis of DEGs**

To uncover the dynamic characteristics of the growth and development of *P. ternata* bulbils at different stages, the transcripts of the bulbils at five stages were compared. KEGG analysis showed that the gene expression pathways and biosynthesis pathways of *P. ternata* bulbils differed at different growth stages. It was also found that 12 biological expression pathways, namely, photosynthesis (ko00195), photosynthetic-antenna proteins (ko00196), α-linolenic acid metabolism (ko00592), phenylpropanoid biosynthesis (ko00940), flavonoid biosynthesis (ko00941), plant hormone signal transduction (ko04075), pentose and glucuronic acid interconversion (ko00040), cutin (ko00073), suberin and wax biosynthesis, starch and sucrose metabolism (ko00500), biotin metabolism (ko00780), brassinosteroid biosynthesis (ko00905), and plant-pathogen interaction (ko04626) were evidently enriched in the bulbils, indicating that these 12 pathways play an important role in the whole-life process of bulbils including formation, growth and development. *P. ternata* bulbils, formed and attached to the underground petioles, are vital "reservoir" organs of plants in the soil responsible for storing nutrients. Photosynthesis exerts a crucial effect on the yield and quality of bulbils. Two biological pathways, photosynthesis (ko00195) and photosynthetic-antenna proteins (ko00196), were significantly enriched in the bulbils at different growth stages, indicating that the bulbils may be a key site for the regulation of photosynthesis of plants, and antenna proteins are integral parts for light-harvesting complex to obtain light energy in photosynthesis. Remarkable enrichment of the biosynthetic pathways of cutin (ko00073), suberin, and wax could be observed at ZY_1, ZY_2 and ZY_3, which indicated that more wax and lignin are synthesized and accumulated in the bulbils during the immature period (Figs5A and 5B). The enrichment results of pentose and glucuronic acid interconversion (ko00040), starch and sucrose metabolism (ko00500), α-linolenic acid metabolism, phenylpropanoid biosynthesis (ko00940), flavonoid biosynthesis (ko00941), plant hormone signal transduction (ko04075) and brassinosteroid biosynthesis (ko00905) demonstrated that primary and secondary metabolites were continuously synthesized and accumulated in bulbils at different growth stages (Figs 5A-5D).

**Figure 5:** KEGG enrichment analysis of gene expression pathways in *Pinellia ternata* bulbils at different growth stages.

**DEGs related to sucrose and starch metabolic pathways**

*P. ternata* bulbils are "reservoir" organs, where sucrose and starch are their vital storage substances, laying a solid material foundation in the morphogenesis process of the bulbils. For further exploration of the dynamic variation characteristics and metabolic mechanisms of sucrose and starch synthesis and accumulation in the bulbils at different growth and development stages, their biological pathways and related gene expression levels were subjected to visualized analysis. According to KEGG data, there were 12 key DEGs in the sucrose and starch metabolic pathways in the bulbils at different growth stages. As illustrated in Fig6, variations in the key DEGs related to sucrose and starch metabolism (INV, scrK, E2.4.1.14, SPP, SUS, UGP2, pgm, glgC, GPI, glgA, WAXY and GBE1), at different growth stages revealed that these genes were expressed at different levels in the heat maps at different stages. Except for the high expression levels of INV at ZY_1, ZY_2, ZY_3 and ZY_4, the expression levels of the other 11 genes were up-regulated at ZY_2, ZY_3 and ZY_4, especially at ZY_2 and ZY_4, indicating that sucrose and starch metabolism are more active at ZY_2 and ZY_4. In the present study, sucrose was the main substrate for the synthesis and accumulation of starch in the bulbils. Sucrose underwent a series of transformation and metabolism to produce uridine diphosphate glucose and D-glucose 6-phosphate. Then the same intermediate α-glucose-1p generated under the action of two key genes, UGP2 and pgm, was finally produced into starch under the action of key genes, glgC, glgA, WAXY and GBE1. The expression of these key enzyme genes was significantly up-regulated in ZY_2 and ZY_4 periods, which was consistent with the change of starch content.

**Figure 6:** Heat maps of DEGs related to sucrose and starch synthesis in the bulbils at different growth stages plotted by log2 transformation of FPKM values.

**DEGs related to the endogenous hormone metabolic pathway**

As trace and efficient organic compounds synthesized in plants, endogenous hormones are crucial for plant organogenesis, morphogenesis and other growth and development processes. For further exploration of the metabolic mechanisms and dynamic variation characteristics of endogenous hormones in *P. ternata* bulbils at different growth and development stages, the metabolic pathways of four endogenous hormones and the expression levels of related genes were examined. The heat maps (Fig7) illustrated variations in ABA, GA, IAA, JA and other related enzymes in the metabolic pathways at different growth stages, and revealed that these genes were differentially expressed at different growth stages. According to the KEGG enrichment analysis results, it was found that four enzymes [15-cis phytoene synthase (crtB), ζ-carotene desaturase (ZDS), β-carotene hydroxylase (LUT5) and 9-cis-epoxycarotenoid dioxygenase (NCED)] encoded by eight highly expressed DEGs were involved in the metabolic pathway of ABA. Firstly, geranylgeranyl pyrophosphate was converted into prephytoene diphosphate and then into crtB, whose expression level was the highest at ZY_2 and ZY_4. Secondly, ZDS and NCEDwere involved in the production of lycopene and 2-cis,4-trans xanthosine, exhibited the highest expression levels at ZY_2, ZY_3 and ZY_4, while LUT5 in the production of zeaxanthin was mainly up-regulated at ZY_2 and ZY_4(Fig7A).The downstream enzyme of GA, namely, gibberellin 2-oxidase (GA2ox), played a key role in the metabolic pathway of GA, involving the transformation of GA20 to GA29, GA9 to GA51, GA4 to GA34, and GA1 to GA8, as well as the catabolic reactions of four GA isoforms, GA29, GA51, GA34 and GA8 (Fig7B). Results indicated that the expression levels of GA2ox was up-regulated at ZY_2, ZY_4 and ZY_5, implying obvious GA synthesis and metabolism at these stages. According to the KEGG analysis, four key enzymes encoded by 11 DEGs were mainly involved in the IAA metabolic pathway. In this pathway tryptophan, the substrate, generated tryptamine and indolepyruvate under the action of DOPA decarboxylase (DDC) and l-tryptophan-pyruvate aminotransferase (TAA1), which took part in the subsequent biochemical reactions and then were catalyzed to IAA, respectively, under the action of downstream enzymes, acetaldehyde dehydrogenase (ALDH) and indole-3-pyruvate monooxygenase (YUCCA). Generally speaking, the four enzymes (DDC, TAA1, ALDH and YUCCA) were highly expressed at ZY_2 and ZY_4, and their expression levels at ZY_3 were lower than that of other stages (Fig7C). At different growth stages, the JA metabolic pathway in the bulbils mainly involved five enzymes encoded by 16 DEGs, and these enzymes showed differences in heat maps at different stages. The substrate lecithin produced α-linolenic acid under the action of secreted phospholipase A2 (PLA2), and then reacted with a series of enzymes such as lipoxygenase 2S (LOX2S), allene oxide synthase (AOS), 3-hydroxyacyl-CoA dehydrogenase (MFP2) and acetyl-CoA acyltransferase (fadA) to produce JA. The expression levels of DEGs revealed that the expressions of most genes were mainly up-regulated at ZY_2, ZY_3 and ZY_4 (Fig7D).

**Figure 7:** Heat maps of DEGs related to endogenous hormone metabolism in the bulbils at different growth stages plotted by log2 transformation of FPKM values. A. ABA. B. GA. C.IAA. D.JA.

**Transcription Factors**

At different growth stages, TF coding genes of 5 samples were differentially expressed. According to statistics, there were 155 and 293 TF coding DEGs in the two comparison groups, ZY_2 vs. ZY_1 and ZY_5 vs. ZY_4, respectively, all involving 27 families. In addition, there were 223 and 186 TF coding DEGs in the two comparison groups, ZY_3 vs. ZY_2 and ZY_4 vs. ZY_3, respectively, all involving 25 families. AP2/ERF-ERF, bHLH, MYB-related, NAC, WRKY, C3H, C2H2, and MYB families were eight TF families with the largest number of DEGs (Fig 8A). The expressions of most of AP2/ERF-ERF, bHLH, WRKY and MYB coding genes were up-regulated at ZY_3 and ZY_5, which were markedly higher than those at the other three stages. Additionally, the expressions of NAC and C3H coding genes were raised at ZY_4, while other genes were up-regulated at ZY_3 and ZY_5. Moreover, the expressions of C2H2 coding genes were up-regulated at ZY_2, ZY_3 and ZY_5, and those of MYB-related family coding genes were up-regulated at five stages, but the expressions of most genes were up-regulated mainly at ZY_3 and ZY_5 (Fig8B).

**Figure 8:** DEGs related to the TF family in the bulbils at different growth stages.A. The number of DEGs at different growth stages. B. Heat maps of DEGs at each stage.

**Plant hormone signal transduction**

Plant endogenous hormones are able to regulate the growth and development of plants by binding to specific protein receptors, and play a small but efficient regulatory role in plant morphogenesis and biological metabolism. Among them, IAA was directly involved in the regulation process in the nucleus, while the other five hormones played a regulatory role in the nucleus after signal transduction in the cytoplasm (Fig 9A). In this study, the differential expressions of protease genes that can affect DNA expressions were analyzed. In the IAA signal transduction pathway, ARF gene was up-regulated at ZY_1, ZY_2, ZY_3, and ZY_4 and markedly down-regulated at ZY_5. A-ARR, synthesized by transcription and translation, played a physiological role in the nucleus. Its coding genes were mainly raised at ZY_1, ZY_3 and ZY_5, which were notably higher than those at the other two stages. In the process of GA signal transduction, TFs were dissociated by DELLA to inhibit DNA expressions, and most genes were up-regulated at ZY_3 and ZY_5. ABF entered the nucleus from the cytoplasm and acted directly on DNA. Its coding genes were mainly up-regulated at ZY_2 and ZY_4, and the up-regulation of some genes appeared at ZY_3. Furthermore, EIN3 coding genes were highly expressed at ZY_3 and ZY_5, while MYC2 in the JA signal transduction pathway was notably up-regulated at ZY_3 (Fig9B).

**Figure 9:** DEGs related to plant hormone signal transduction in the bulbils at different growth stages. A. Plant hormone signal transduction pathway B. Heat maps of DEGs at different growth stages.

**Weighted gene co-expression network analysis (WGCNA)**

**Construction of co-expression network modules**

The synergistic relationship between the content of endogenous metabolites of *P. ternata* bulbils and the obtained transcript expression values at different growth and development stages were investigated, and the transcripts closely related to endogenous metabolites were speculated. To this end, 15 samples, with a total of 68,386 transcripts, were selected to construct the whole-gene co-expression network. After the genes with low variation in expression (standard deviation ≤0.5) were excluded, the remaining 39,074 genes were finally used to construct the co-expression network. Through hierarchical clustering of dissTOM matrix (Fig10A), a total of 18 co-expression clustering modules were obtained (Fig10B). Among these modules, the grey module (Grey) was a set of genes that could not be assigned to any module and showed no reference significance. The blue2 module (Fig10C) and the dark olive green module (Fig10D) were selected to display the transcript expression profiles constructed by the co-expression network, respectively, under the co-expression clustering modules.

**Figure 10:** Gene co-expression network analysis of 15 samples of bulbils at different growth stages. A. TOM correlation heat maps for all transcripts. B. Co-expression network modules constructed by transcripts. Different colors represent the network modules constructed, and the phylogenetic tree represents the hierarchical clustering of different samples, where each color corresponds to one module. C. Transcript expression profiles in the co-expressed blue2 module. D. Transcript expression profiles in the co-expressed dark olive green module. In the histogram, the x-axis represents all samples, and the y-axis represents the expression profile of the transcripts in the module.

**Screening of co-expressed genes of endogenous metabolites in the bulbils at different growth stages**

Among the 18 co-expression clustering modules obtained, antique white 2、blue 2、dark olive greenand honey dew modules displayed significantly positive correlations with the content of endogenous metabolites in the bulbils (Fig11A). Close correlations of the antique white 2 module with the content of IAA (R=0.92，*p＜*0.001) and ABA (R=0.92，*p＜*0.001) were observed. The blue2 module, showed extremely marked positive correlations with JA (R=0.77，*p＜*0.001) and ABA（R=0.79，*p＜*0.001）. The dark olive green module was positively correlated with starch (R=0.86，*p＜*0.001), and the honeydew module displayed the closest correlation with sucrose（R=0.96，*p＜*0.001）、SPS（R=0.94，*p＜*0.001）、SuSy（R=0.84，*p＜*0.001）、SS（R=0.90，*p＜*0.001）、JA（R=0.84，*p＜*0.001）、ABA（R=0.82，*p＜*0.001）.

Finally, Cytoscape was employed to visualize the module network for the screening of the core genes of each module. It was found that the core gene was TRINITY_DN18564_c0_g1_i2_5 in the antiquewhite 2 module, TRINITY_DN13478_c0_g1_i1_3 in the blue 2 module, TRINITY_DN27378_c0_g1_i3_2 in the darkolivegreen module, and TRINITY_DN24626_c0_g1_i2_3 in the honeydew module (Figs11B-11E). These genes are annotated as hypothetical proteins in the NR database. Further comparison of their RNA sequences with NCBI Reference Sequence indicates that the structure and function of the proteins encoded by these genes have not been completely resolved. These results suggest that the analysis of the structure and function of unknown proteins may be a key way to further understand the regulatory network of genes related to the characteristics of bulbils development.

**Figure 11:**Core transcripts that were significantly associated with changes in endogenous metabolite content of sucrose, starch, SPS, SuSy, SS, IAA, JA, GA and ABA were screened in the co-expression module. A. Correlation heat maps of the identified co-expression module with the content of endogenous metabolites in the bulbils. B. Gene co-expression network of the identified antiquewhite2 module related to the content of IAA and ABA. C. Gene co-expression network of the identified blue2 module related to the content JA and ABA. D. Gene co-expression network of the identified darkolivegreen module related to the content of starch. E. Gene co-expression network of the identified honeydew module related to the content of sucrose, JA, ABA and SPS, SuSy, SS.

**Discussion**

**Sucrose and starch metabolism**

Bulbils are vital storage organs and reproductive organs of *P. ternata*. Sucrose and starch are the end products of photosynthesis, which are responsible for the transportation and storage of carbohydrates in higher plants, respectively, and they are also signaling molecules that coordinate the relationship between source and sink of plants [36,37]. More and more evidence shows that sucrose is able to regulate intracellular metabolism at the level of gene expression [38-40], and wide attention has been paid to its vital role in plant growth and development and assimilate distribution schemes[41]. In the processes of growth and development, plants often adapt to the environment through the spatiotemporal gene expression, and show corresponding botanical characteristics. SPS is a key rate-limiting enzyme during sucrose synthesis[42,43], and SS is a key enzyme that decomposes sucrose and serves as a substrate for starch synthesis [44]. SPS is involved in the carbon allocation between sucrose and starch[45], and with high activity, it increases the sucrose content and decreases starch synthesis [46]. In our study, SPS activity showed a decreasing trend except for the significant increase in the ZY_4 period. Compared with the measured sucrose and starch contents, it was found that the sucrose content increased significantly in the ZY_4 period, while the starch content decreased compared with that in the ZY_2 period. In conclusion, the content of sucrose and starch increased significantly in ZY_2 and ZY_4 stages during the five growth stages, which was consistent with the changes of SuSy, SPS and SS enzymes.Among the metabolic pathways of sucrose and starch of concern, the carbon required by bulbils was mainly obtained by the transportation of the mother stem at the early stage and the photosynthesis of leaves after emergence. At the first four stages, INV coding genes were continuously highly expressed. They transformed the carbon source transported from the mother stem to the bulbils and the assimilates produced by photosynthesis into D-glucose 6-phosphate and fructose, which were finally synthesized into starch under the action of downstream genes. Among them, the coding genes of eight enzymes, namely, E2.4.1.14, SUS, UGP2, pgm, glgC, glgA, WAXY and GBE1, were evidently up-regulated at ZY_2 and ZY_4 in terms of expression, which was identical to the variation trend of the starch content. This result indicated that ZY_2 and ZY_4 are crucial stages of starch accumulation during the growth and development of bulbils.

**Endogenous hormone metabolism**

Plant hormones are chemical messengers, which can regulate the growth and development of plants and increase their biomasses[47,48]. As plant hormones, ETH and JA exhibit defensive functions, which can respond to external stimuli and improve the immunity of plants[49].In this study, the content of jasmonic acid generally increased from ZY_1 to ZY_4 stage, and was significantly lower in ZY_5 stage than in other stages, suggesting that jasmonic acid enhanced the adaptability of bulbils to the environment during the transition from tender tissue to mature tissue. These results support the results of related enzyme gene expression levels. Among GAs, GA_1_, GA_3_, GA_4_ and GA_7_ have been proven to have a wide range of biological activities[50]. GA2ox was differentially expressed in the GA metabolic pathway at different stages, involving GA_1_, GA_4_, GA_9_, GA_20_, GA_29_, GA_34_ and GA_51_.There was no significant difference in gibberellin content between ZY_1 and ZY_4 stages, and the content of gibberellin remained at a low level, indicating that the low concentration of GA in the natural state was conducive to the normal growth of the bulbils. IAA regulates the growth and development of plants and the stress response to external environmental stimuli, and it often plays a coordinated role with other hormones[51]. In this study, the content of IAA in bulbils decreased during the whole growth period, and auxin content was highest only at the initial formation of bulbils (ZY_1 stage) and when new bulbils was formed from the stalk of germination of bulbils (ZY_4 stage), while the content was lower in other periods. Therefore, it is speculated that IAA may have a strong regulation effect during the formation of bulbils. The regulation was weakened in the growth stage of bulbils, which was in good agreement with the expression levels of related genes in the IAA metabolic pathway. ABA is a type of growth-inhibiting hormone involved in regulating various physiological processes[52]. ABA content showed an increasing trend before the ZY_4 stage, and significantly increased in the ZY_4 stage. Alongwith the whole expansion and growth stage of the bulbils, the variation characteristics of ABA content might be related to the regulation of the distribution and accumulation of carbohydrates in *P. ternata*.

**Transcription Factors**

The AP2/ERF family, as one of the largest TF families in plants, is widely involved in plant growth and development, biosynthesis and various stress responses. AP2/ERF-ERF is one of its subfamilies (ETH response factor). Many ERF subfamilies have been proven to promote plant growth and development, and improve tolerance to hormone and abiotic stress in maize, *Arabidopsis thaliana*, wheat and tobacco[53]. In addition, some other large TF families were also found in our research, such as bHLH, MYB-related, NAC, WRKY, C3H, C2H2 and MYB families. The coding genes of these TFs were differentially expressed in the heat maps at different stages, especially at ZY_3 and ZY_5, during which their expressions were evidently up-regulated. Interestingly, a common feature is shared by these TFs, i.e., they are large families comprising a large number of members, which can participate in plant growth and development and biological metabolism, and respond to external environmental stress. For example, TFs in the bHLH family are generally involved in plant growth and metabolism, photomorphogenesis, light signal transduction and secondary metabolism, and can improve the resistance of plants to environmental stress[54,55]. In the MYB-related family, LlMYB3 protein is overexpressed in transgenic Arabidopsis thaliana, which confirms ABA hypersensitivity and indicates the enhanced tolerance to cold, drought and salt stresses. LlMYB3 may also be involved in the anthocyanin synthesis pathway [56]. Some uncommon TFs, such as C3H and C2H2, are essential in plant stress responses[57,58], although the regulatory network is still unclear. The number and function of TFs are often quite different in different plants, and there is little research on TFs in *P. ternata*. The results of this study manifested that ZY_3 and ZY_5 were two distinct growth and development stages of the bulbils, when many TFs were up-regulated intensively.

**Conclusions**

*P. ternata* bulbils is a vital medicinal plant resource, understanding the growth and development pattern as well as the molecular mechanism of the bulbils is of great significance to the protection and development of its medicinal resource. This study found that the ZY_2 and ZY_4 stages are the critical periods for bulbils to accumulate sucrose and starch, and the changes in sucrose and starch contents are basically consistent with the activities of related enzymes and the differential expression characteristics of enzyme genes in their metabolic pathways. Endogenous hormones are essential for the development of bulbils, and the differential expression characteristics of key enzyme genes in their metabolic pathways well support the measurement results of their contents. Eight TF families were significantly up-regulated in the ZY_3 and ZY_5 stages. Four core transcripts involved in the development process of bulbils have been screened by WGCNA. The structures and functions of proteins encoded by these transcripts have not been fully analyzed yet, indicating that analyzing the structures and functions of these unknown proteins may be a key approach to gain a deeper understanding of the gene regulatory network related to bulbils development. Finally, the reliability of transcriptome results was verified by qRT-PCR analysis. This study provides an information source for analyzing the molecular mechanism of bulbils growth and development, and also helps to solve the problem of lack of genetic information in non-model plant species.

**Acknowledgements**

Thank Gansu University of Chinese Medicine for providing the research experiment platform, and also thank all the authors for their contributions to this research.

**References**

1. Noushahi H A, Zhu Z, Khan A H, Ahmed U, Haroon M, Asad M, et al. Rhizosphere microbial diversity in rhizosphere of *P. ternata* intercropped with maize. *3 Biotech*, 2021;11(11): 469. DOI: 10.1007/s13205-021-03011-3.
2. Moon B C, Kim W J, Ji Y, Lee Y M, Kang Y M, Choi G. Molecular identification of the traditional herbal medicines, Arisaematis Rhizoma and Pinelliae Tuber, and common adulterants via universal DNA barcode sequences. *Genetics and molecular research : GMR*. 2016;15(1): 10.4238/gmr.15017064 DOI: 10.4238/gmr.15017064.
3. Kong X, Yang M, Abbas H, Wu J, Li M, Dong W. Antimicrobial genes from Allium sativum and *P. ternata* revealed by a Bacillus subtilis expression system. *Scientific reports*. 2018;8(1): 14514. DOI: 10.1038/s41598-018-32852-x.
4. Wei S, Peng Z. Survey of *P. ternata* and its adulterants. *Journal of Chinese medicinal materials*. 2003;26(11): 828-832.
5. Peng W, Li N, Jiang E, Zhang C, Huang Y, Tan L,et al. A review of traditional and current processing methods used to decrease the toxicity of the rhizome of *P. ternata* in traditional Chinese medicine. *Journal of ethnopharmacology*. 2022;299:115696.DOI: 10.1016/j.jep.2022.115696.
6. An S J, Liang J, Liu X C, Liang J C, Chen H H, Liu X M. Quality control status and predictive analysis of quality markers of Pinellia ternate. *Chinese Archives of Traditional Chinese Medicine*. 2023; 42(03):168-172. DOI:10.13193/j.issn.1673-7717.2023.03.036.
7. Mao R, He Z. *P. ternata* (Thunb.) Breit: A review of its germplasm resources, genetic diversity and active components. *Journal of ethnopharmacology*. 2020; 263:113252. DOI: 10.1016/j.jep.2020.113252.
8. Bai J, Qi J, Yang L, Wang Z, Wang R, Shi Y. A comprehensive review on ethnopharmacological, phytochemical, pharmacological and toxicological evaluation, and quality control of *P. ternata* (Thunb.) Breit. *Journal of ethnopharmacology*. 2022; 298: 115650. DOI: 10.1016/j.jep.2022.115650.
9. Hao E W, Su Z X, Gong Y L, Du Z C, Yang X, Huang C T, et al. Analysis on application law of dampness-removing traditional Chinese medicines in treatment of coronavirus disease 2019. *Chinese herbal medicines*. 2021;13(4): 518-524. DOI: 10.1016/j.chmed.2021.09.011.
10. Hao C L, Hu F R,Yang P P. Research Progress on Plant Bulbil Formation. *Molecular Plant Breeding*. 2021; 1-10. <https://kns.cnki.net/kcms/detail/46.1068.s.20210722.1406.012>.
11. Xia L S. The role of Auxin signaling pathway related genes in the development of bulbil for two species of plant. master's thesis, Gui Zhou University. 2019.
12. Gao M, Zhang S, Luo C, He X, Wei S, Jiang W, etal. Transcriptome analysis of starch and sucrose metabolism across bulb development in Sagittaria sagittifolia. *Gene*. 2018; 649: 99-112. DOI: 10.1016/j.gene.2018.01.075.
13. Zhang K, Wu Z, Tang D, Luo K, Lu H, Liu Y, etal. Comparative Transcriptome Analysis Reveals Critical Function of Sucrose Metabolism Related-Enzymes in Starch Accumulation in the Storage Root of Sweet Potato. *Frontiers in plant science*. 2017; 8: 914. DOI: 10.3389/fpls.2017.00914.
14. Angeles-Núñez J G, Tiessen A. Arabidopsis sucrose synthase 2 and 3 modulate metabolic homeostasis and direct carbon towards starch synthesis in developing seeds. *Planta*. 2010; 232(3): 701-718. DOI: 10.1007/s00425-010-1207-9.
15. Wang Z, Xu Y, Chen T, Zhang H, Yang J, Zhang J. Abscisic acid and the key enzymes and genes in sucrose-to-starch conversion in rice spikelets in response to soil drying during grain filling. *Planta*. 2015; 241(5): 1091-1107. DOI: 10.1007/s00425-015-2245-0.
16. Li X, Wang C, Cheng J, Zhang J, da Silva J A, Liu X, etal. Transcriptome analysis of carbohydrate metabolism during bulblet formation and development in Lilium davidii var. unicolor. *BMC plant biology*. 2014; 14: 358. DOI: 10.1186/s12870-014-0358-4.
17. Xu X, van Lammeren AA, Vermeer E, Vreugdenhil D. The role of gibberellin, abscisic acid, and sucrose in the regulation of potato tuber formation in vitro. *Plant physiology*. 1998; 117(2): 575-584. DOI: 10.1104/pp.117.2.575.
18. Navarro C,Cruz-Oró E,Prat S. Conserved function of FLOWERING LOCUS T (FT) homologues as signals for storage organ differentiation. *Current opinion in plant biology*. 2015; 23: 45-53. DOI: 10.1016/j.pbi.2014.10.008.
19. He G, Yang P, Tang Y, Cao Y, Qi X, Xu L, etal. Mechanism of exogenous cytokinins inducing bulbil formation in Lilium lancifolium in vitro. *Plant cell reports*. 2020; 39(7): 861-872. DOI: 10.1007/s00299-020-02535-x.
20. Xia L. The role of auxin signaling pathway-related genes in bulbil development of two plant species. (Guizhou: Guizhou university). 2019.
21. Muller D and Leyser O. Auxin, cytokinin and the control of shoot branching. Ann. Bot. 2011;107, 1203–1212. doi: 10.1093/aob/mcr069.
22. Miyamoto K, Kotake T, Boncela A J, Saniewski M and Ueda J. Hormonal regulation of gummosis and composition of gums from bulbs of hyacinth (*hyacinthus orientalis*). J. Plant Physiol. 2015;174, 1–4. doi: 10.1016/j.jplph.2014.10.007.
23. Shu F, Wang D, Sarsaiya S, Jin L, Liu K,Zhao M, et al. Bulbil initiation: a comprehensivereview on resources, development, andutilisation, with emphasis on molecularmechanisms, advanced technologies, and future prospects.Front. Plant Sci. 2024;15:1343222.doi: 10.3389/fpls.2024.1343222.
24. He G, Yang P, Tang Y, Cao Y, Qi X, Xu L, et al. Mechanism of exogenous cytokinins inducing bulbil initiation in *lilium lancifolium* in vitro. Plant Cell Rep. 2020; 39 (7), 861–872. doi: 10.1007/s00299-020-02535-x.
25. Prasad R. Cytokinin and its key role to enrich the plant nutrients and growth under adverse conditions-an update. Front. Genet. 2022; 13. doi: 10.3389/fgene.2022.883924.
26. Zhang Y, MAO G and Deng, L. Effect of gibberellin treatment on agronomic and quality traits under shade conditions (Modern Agricultural Science and Technology), 2018; 71–72.
27. Guo C, Li J, Li M, Xu X, Chen Y, Chu J, et al. Regulation mechanism of exogenous brassinolide on bulbil initiation and development in *pinellia ternata*. Front. Plant Sci. 2021; 12. doi: 10.3389/fpls.2021.809769.
28. Mou L, Zhang L, Qiu Y, Liu M, Wu L, Mo X, et al. Endogenous Hormone Levels and Transcriptomic Analysis Reveal the Mechanisms of Bulbil Initiation in Pinellia ternata. Int.J. Mol. Sci. 2024; 25, 6149. <https://doi.org/10.3390/ijms25116149>.
29. Guo C, Zhang Y, Wu D, Wang M, Du Y, Chu J, et al. Principal component analysis to assess the changes of yield and quality in pinellia ternata at different stages after brassinolide treatments. Int. J. Mol. Sci. 2022; 23(23), 15375. doi: 10.3390/ijms232315375.
30. Goryachev A B and Mallo M. Patterning and morphogenesis from cells to organisms: progress, common principles and new challenges. Front. Cell. Dev. Biol. 2020; 8.doi: 10.3389/fcell.2020.6024830.1007/s11103-016-0439-x.
31. Yang P, Xu L, Xu H, Tang Y, He G, Cao Y, et al. Histological and transcriptomic analysis during bulbil initiation in lilium lancifolium. Front. Plant Sci. 2017; 8, 1508. doi: 10.3389/fpls.2017.01508.
32. Bolger A M, Lohse M, Usadel B. Trimmomatic: a flexible trimmer for Illumina sequence data. *Bioinformatics (Oxford, England)*. 2014; 30(15): 2114–2120. DOI: 10.1093/bioinformatics/btu170.
33. Grabherr M G, Haas B J, Yassour M, Levin J Z, Thompson D A, Amit I, etal. Full-length transcriptome assembly from RNA-Seq data without a reference genome. *Nature biotechnology*. 2011; 29(7): 644–652. DOI: 10.1038/nbt.1883.
34. Langmead B, Salzberg S L. Fast gapped-read alignment with Bowtie 2. *Nature methods*,.2012; 9(4): 357-359. DOI: 10.1038/nmeth.1923.
35. Roberts A, Pachter L. Streaming fragment assignment for real-time analysis of sequencing experiments. *Nature methods*. 2013; 10(1): 71-73. DOI: 10.1038/nmeth.2251.
36. Stein O, Granot D. An Overview of Sucrose Synthases in Plants. *Frontiers in plant science*. 2019; 10: 95. DOI: 10.3389/fpls.2019.00095.
37. Zhang W Q, Wang Y L, Zhu D F,Chen H Z, Xiang J, Zhang Y K, etal. Response of Grain Starch and Sucrose Metabolism-related Enzyme Activities to Night Temperature Rise in Conventional Indica Rice Huanghuazhan. *Current Biotechnology*. 2019;9(03): 283-289. DOI:10.19586/j.2095-2341.2019.0010.
38. Gibson S I. Plant sugar-response pathways. Part of a complex regulatory web. *Plant physiology*. 2000; 124(4): 1532-1539. DOI: 10.1104/pp.124.4.1532.
39. Koch K E, Ying Z, Wu Y, Avigne W T. Multiple paths of sugar-sensing and a sugar/oxygen overlap for genes of sucrose and ethanol metabolism. *Journal of experimental botany*. 2000; 417-427. DOI: 10.1093/jexbot/51.suppl_1.417.
40. Daloso D M, Dos Anjos L, Fernie A R. Roles of sucrose in guard cell regulation. *The New phytologist*. 2016; 211(3): 809–818. DOI: 10.1111/nph.13950.
41. Lunn J E, Furbank R T. Localisation of sucrose-phosphate synthase and starch in leaves of C4 plants. *Planta*. 1997; 202(1): 106-111. DOI: 10.1007/s004250050108.
42. Champigny M L. Integration of photosynthetic carbon and nitrogen metabolism in higher plants. *Photosynthesis research*. 1995; 46(1-2): 117–127. DOI: 10.1007/BF00020422.
43. Anur R M, Mufithah N, Sawitri W D, Sakakibara H, Sugiharto B. Overexpression of Sucrose Phosphate Synthase Enhanced Sucrose Content and Biomass Production in Transgenic Sugarcane. *Plants (Basel, Switzerland)*. 2020; 9: 200. DOI: 10.3390/plants9020200.
44. Bilska-Kos A, Mytych J, Suski S, Magoń J, Ochodzki P, Zebrowski J. Sucrose phosphate synthase (SPS), sucrose synthase (SUS) and their products in the leaves of Miscanthus × giganteus and Zea mays at low temperature. *Planta*. 2020; 252: 23. DOI:10.1007/s00425-020-03421-2.
45. Rufty T W, Huber S C. Changes in Starch Formation and Activities of Sucrose Phosphate Synthase and Cytoplasmic Fructose-1,6-bisphosphatase in Response to Source-Sink Alterations. *Plant physiology*. 1983; 72(2): 474-480. DOI: 10.1104/pp.72.2.474.
46. Keeling P L, Wood J R, Tyson R H, Bridges I G. Starch Biosynthesis in Developing Wheat Grain : Evidence against the Direct Involvement of Triose Phosphates in the Metabolic Pathway. *Plant physiology*. 1988; 87(2): 311-319 DOI: 10.1104/pp.87.2.311.
47. Duan N, Jia Y, Xu J, Chen H, Sun P. Research Progress on Plant Endogenous Hormones. *Chinese Agricultural Science Bulletin*. 2015; 31(02): 159-165.
48. Ali S, Baloch A M. Overview of Sustainable Plant Growth and Differentiation and the Role of Hormones in Controlling Growth and Development of Plants Under Various Stresses. *Recent patents on food, nutrition & agriculture*. 2020; 11(2): 105-114. DOI: 10.2174/2212798410666190619104712.
49. Gonzalez N, Beemster G T, Inzé D. David and Goliath: what can the tiny weed Arabidopsis teach us to improve biomass production in crops?. Current opinion in plant biology.2009; 12(2): 157-164. DOI: 10.1016/j.pbi.2008.11.003.
50. Davière J M, Achard P. Gibberellin signaling in plants. *Development (Cambridge, England)*. 2013; 140(6): 1147–1151. DOI: 10.1242/dev.087650.
51. Mazzoni-Putman S M, Brumos J, Zhao C, Alonso J M, Stepanova A N. Auxin Interactions with Other Hormones in Plant Development. *Cold Spring Harbor perspectives in biology*. 2021; 13(10): a039990. DOI: 10.1101/cshperspect.a039990.
52. Chen K, Li G J, Bressan R A, Song C P, Zhu J K, Zhao Y. Abscisic acid dynamics, signaling, and functions in plants. *Journal of integrative plant biology*. 2020;62(1): 25–54. DOI: 10.1111/jipb.12899.
53. Hao L, Shi S, Guo H, Li M, Hu P, Wei Y, etal. Genome-wide identification and expression profiles of ERF subfamily transcription factors in Zea mays. *PeerJ*, 2020; 8: e9551. DOI: 10.7717/peerj.9551.
54. Sun X, Wang Y, Sui N. Transcriptional regulation of bHLH during plant response to stress. *Biochemical and biophysical research communications*. 2018; 503(2): 397-401. DOI: 10.1016/j.bbrc.2018.07.123.
55. Guo J, Sun B, He H, Zhang Y, Tian H, Wang B. Current Understanding of bHLH Transcription Factors in Plant Abiotic Stress Tolerance. *International journal of molecular sciences*. 2021; 22(9): 4921. DOI: 10.3390/ijms22094921.
56. Yong Y, Zhang Y, Lyu Y. A MYB-Related Transcription Factor from Lilium lancifolium L. (LlMYB3) Is Involved in Anthocyanin Biosynthesis Pathway and Enhances Multiple Abiotic Stress Tolerance in Arabidopsis thaliana. *International journal of molecular sciences*. 2019; 20(13): 3195. DOI: 10.3390/ijms20133195.
57. Liu C, Xu X, Kan J, Cheng Z M, Chang Y, Lin J, etal. Genome-wide analysis of the C3H zinc finger family reveals its functions in salt stress responses of Pyrus betulaefolia. *PeerJ*. 2020;8: e9328. DOI: 10.7717/peerj.9328.
58. Han G, Lu C, Guo J, Qiao Z, Sui N, Qiu N, etal. C2H2 Zinc Finger Proteins: Master Regulators of Abiotic Stress Responses in Plants. *Frontiers in plant science*. 2020; 11: 115. DOI: 10.3389/fpls.2020.00115.
