# Supplementary material for: Transcriptomic and biochemical insights into key gene networks driving bulbil development of Pinellia ternata (Thunb.) Breit
Source: PLoS One. 2025 Feb 11;20(2):e0314396. doi: 10.1371/journal.pone.0314396 (PMC11813136; doi:10.1371/journal.pone.0314396)
Supplement: Attachment — Submitted filename: Response to Reviewers.docx [file pone.0314396.s019.docx]

**Response to Reviewers**

[October 17th]

Emily Chenette

Editor-In-Chief

*PLOS ONE*

Dear Ph.D Arun Kumar Shanker:

We wish to re-submit the manuscript titled “**Transcriptomic and Biochemical Insights into Key Gene Networks Driving Bulbil Development of *Pinellia ternata* （Thunb.）Breit**.” The manuscript ID is PONE-D-24-28450R1.

Funding Statement: This work was supported by the Science and Technology Key R&D Program in Gansu Province (21YF5NA130) and the Special Foundation for Construction of National Traditional Chinese Medicine Industry Technology System in China "Supported by the earmarked fund for CARS-21".There was no additional external funding received for this study. The funders had no role in study design, data collection and analysis, decision to publish, or preparation of the manuscript

Based on your and the reviewers' suggestions, we have rechecked the manuscript and revised it appropriately. At the same time, we checked all references in the manuscript to make sure that it is complete and correct, and responded to and revised each of the review comments, as detailed below

**Review Comments to the Author：**

**Reviewer #1:**

1. The manuscript is improved much. All the recommendations have been updated. However, would it be possible to add gene sequences within supplimentry 2 file. Yes you have mentioned ids of genes but hand on placement of gene sequences within manuscript material would make it more elegant.

**Authors’ Response:** In response to your suggestion, we have added sequence information for the genes in Supplementary file 2.

**Reviewer #3:**

1. The term "irregular observation" in the Materials section can be changed to "frequent observation" for better accuracy and clarity.

**Authors’ Response:** We have changed the term “irregular observation” to “regular observation” in the revised manuscript.

2. The quality of Figure 11 can be improved to ensure it is visually clear and easy to interpret.

**Authors’ Response:** In response to your helpful suggestion, we have made appropriate adjustments to the Figure 11.
